# Review of the genus *Sparasion* Latreille, 1802 (Hymenoptera: Platygastroidea: Sparasionidae) of the Oriental region with descriptions of new species from India

**DOI:** 10.1186/s40850-023-00169-6

**Published:** 2023-08-25

**Authors:** Kamalanathan Veenakumari, Andrew Polaszek, Roberto Poggi, Kolla Sreedevi, Prashanth Mohanraj, Farmanur Rahman Khan, Gundappa Baradevanal

**Affiliations:** 1 ICAR-National Bureau of Agricultural Insect Resources, P.B. No. 2491, Hebbal, Bengaluru, 560 024 India; 2 Department of Life Sciences, Insects Division, Natural History Museum, Cromwell Road, London, SW7 5BD Great Britain; 3 Museo Civico di Storia Naturale ‘Giacomo Doria’, Genoa, 16121 Italy; 4 Department of Biology, Deanship of Educational Services, Qassim University, Buraidah, Al Qassim Saudi Arabia

**Keywords:** Egg parasitoids, Hymenoptera, New species, Oriental region, Platygastroidea, *Sparasion*, Taxonomy, Tettigoniidae

## Abstract

**Background:**

The genus *Sparasion*, endoparasitoids of Tettigoniidae, occur in the Nearctic, Palearctic, Afrotropical and Oriental regions. It is absent in the Neotropics and Australasia. Of the thirteen species found in the Oriental region only a single species is from India.

**Results:**

Two new species groups - *Sparasion bilahari* species group and *Sparasion manavati* species group - are proposed for species from the Oriental region. Thirty-six species are described and illustrated of which twenty-four are new: *Sparasion albopilosellus* Cameron, 1906 (Pakistan); *S. bhairavi* Veenakumari, **sp. n**. (India); *S*. *bhupali* Veenakumari, **sp. n**. (India); *S*. *bihagi* Veenakumari, **sp. n**. (India); *S*. *bilahari* Veenakumari, **sp. n**. (India); *S. cellularis* Strand, 1913 (Taiwan); *S. coconcu*s Kozlov and Lê, 2000 (Vietnam); *S. coeruleus* Kieffer, 1905 (Sumatra); *S. cullaris* Kozlov and Lê, 2000 (Vietnam); *S*. *darbari* Veenakumari, **sp. n**. (India); *S*. *deepaki* Veenakumari, **sp. n**. (India); *S. domes* Kozlov and Lê, 2000 (Vietnam); *S*. *elbakyanae* Veenakumari, **sp. n**. (India); *S. formosus* Kieffer, 1910 (Taiwan); *S*. *hindoli* Veenakumari, **sp. n**. (India); *S*. *kalyani* Veenakumari, **sp. n**. (India); *S*. *kanakangi* Veenakumari, **sp. n**. (India); *S*. *karivadana* Veenakumari, **sp. n**. (India); *S. lividus* Johnson, Masner & Musetti, 2008 (Philippines); *S*. *manavati* Veenakumari, **sp. n**. (India); *S*. *meghmalhari* Veenakumari, **sp. n**. (India); *S. micromerus* Kozlov and Lê, 2000 (Vietnam); *S*. *pahadi* Veenakumari, **sp. n**. (India); *S. philippinensis* Kieffer, 1913 (Philippines); *S*. *ratnangi* Veenakumari, **sp. n**. (India); *S*. *rupavati* Veenakumari, **sp. n**. (India); *S*. *salagami* Veenakumari, **sp. n**. (India); *S*. *shulini* Veenakumari, **sp. n**. (India); *S. sinensis* Walker, 1852 (China); *S*. *sivaranjini* Veenakumari, **sp. n**. (India); *S*. *syamalangi* Veenakumari, **sp. n**. (India); *S*. *todi* Veenakumari, **sp. n**. (India); *S. travancoricus* Mani and Sharma, 1981 (India); *S*. *vanaspati* Veenakumari, **sp. n**. (India); *S*. *visvambari* Veenakumari, **sp. n**. (India) and *S*. *zeelafi* Veenakumari, **sp. n**. (India). Keys to Oriental species of *Sparasion* are furnished. Intrasexual colour morphs among females of *Sparasion* is reported. Lectotype is designated for *Sparasion cellularis* Strand.

**Conclusions:**

Twenty-four new species are added to the Indian fauna of *Sparasion*. Previously described species of Oriental *Sparasion* are redescribed and illustrated. Keys are furnished for all Oriental species.

## Background

Hymenoptera, the most speciose order of insects constituting ten per cent of all known species on earth consists of three groups: aculeate wasps, sawflies, and parasitic Hymenoptera. Platygastroidea, the third largest of the parasitic hymenopteran superfamilies after Ichneumonoidea and Chalcidoidea, are found in almost all habitats except the polar regions. They are relatively more speciose in wet tropical and subtropical forests than in other habitats [1]. The sheer magnitude of their diversity poses a hindrance to their easy documentation on a worldwide basis – this being more so in the tropics. It was in the context of this overwhelming diversity as well as for reasons like the lack of exhaustive regional faunas and poorly defined species that are often indistinguishable based on their original descriptions that Johnson et al. [2] suggested it would be more pragmatic to undertake this task in a piecemeal or incremental manner with the documentation of the world fauna being the final goal. In their view this approach should include the review of the primary types in existence in museums around the world, ‘the addition of targeted, newly collected material’, and the demarcation and inclusion of as many stable character states as possible.

During the course of our studies on the Platygastroidea of the Indian region, it became amply evident that the concerns expressed by Johnson et al. [2] applied in no small measure to the genus *Sparasion* of the Oriental region. Our collections over many years from across India revealed the presence of a surprisingly rich and undocumented fauna of this genus. Of the thirteen species of *Sparasion* currently known from the Oriental region, only one is from India. The remaining are from Southeast Asia, with one each from Pakistan, China and Taiwan. Most were scantily described and poorly illustrated, and some specimens are in such poor condition that even the ‘photographic catalogues of the primary types of the species are of limited use in their recognition’ [2]. In this paper we seek to address these issues and ameliorate the situation with respect to the Oriental *Sparasion* fauna.

The genus *Sparasion* was proposed 220 years ago by Latreille, with *S. cephalotes* Latreille, 1802 as the type species [3]. In their review of the tribe Sparasionini Dahlbom, 1858 (Platygastroidea: Sparasionidae) Johnson et al. [4] recognised five genera, viz. *Electroteleia* Brues, 1940, *Sceliomorpha* Ashmead, 1893, *Sparasion* Latreille, 1802, *Listron* Musetti & Johnson, 2008 and *Mexon* Masner & Johnson, 2008; furnished detailed diagnostic characters for each of these genera; and presented a generic key. In a recent integrated phylogenetic study however, the classification of Platygastroidea was revised and eight families, viz. Geoscelionidae, Janzenellidae, Neuroscelionidae, Nixoniidae, Platygastridae, Proterosceliopsidae, Scelionidae and Sparasionidae, were recognized [5]. The genus *Archaeoteleia* Masner, 1968 (earlier included in the tribe Sparasionini by Masner [6]) was considered a part of the family Sparasionidae in this study in addition to the five genera previously included in the tribe Sparasionini sensu Johnson et al. [4]. With 141 valid species *Sparasion* is by far the most speciose of these six genera [4, 7, 8].

*Sparasion* is represented in the Nearctic, Palearctic, Afrotropical and Oriental regions. It is absent in the Neotropics and Australasia [4]. Two species are known from Baltic amber (Palearctic region) [9]. With 120 species described from the region [5, 6, 10–16] *Sparasion* is many times more speciose in the Palearctic (constituting 86 per cent of all known species) than in the Oriental region (represented by a mere 9 per cent of the described species) [5, 6, 10–16].

Thirteen species of *Sparasion* constitute the presently known Oriental fauna (gender of the species studied indicated in parentheses): *S. albopilosellus* Cameron (Pakistan) (**♂**), *S. cellularis* Strand (Taiwan) (**♂**,**♀**), *S. coconcu*s Kozlov and Lê (Vietnam) (♀), *S. coeruleus* Kieffer (Sumatra) (**♂**,**♀**), *S. cullaris* Kozlov and Lê (Vietnam) (**♀**), *S. domes* Kozlov and Lê (Vietnam) (**♂**), *S. formosus* Kieffer (Taiwan) (**♂**), *S. lividus* Johnson, Masner & Musetti (Philippines) (**♂**), *S. micromerus* Kozlov and Lê (Vietnam) (**♂**), *S. parcepunctatus* Kieffer (Philippines) (**♀**), *S. philippinensis* Kieffer (Philippines) (**♀**), *S. sinensis* Walker (China) (**♀**), and *S. travancoricus* Mani and Sharma (India) (**♀**) [4, 7, 8, 11, 17–21].

The description of 24 new species from India in this study has not only resulted in a threefold increase in the Oriental fauna but also indicates that the hitherto perceived poverty of species here [10] may be an artifact - the result of poor collection - rather than a reflection of the true species diversity of the genus in the region.

All the 24 newly discovered species of *Sparasion* from India are described and illustrated. As this paper examines the Oriental fauna as a whole, all species known from the region are redescribed, and images of the types of all these species (with the exception of *S. parcepunctatus*) are furnished along with keys to the known females and males of all Oriental species of *Sparasion*.

## Results

### *Sparasion* Latreille

*Sparasion* Latreille, 1802: 316. Type: *Sparasion cephalotes* Latreille, by monotypy.

*Oxyurus* Lamarck, 1817: 128. Type: *Sparasion frontalis* Latreille, designated by Muesebeck and Walkley (1951). Preoccupied by *Oxyurus* Rafinesque 1810 (Pisces).

*Bebelus* Gistel, 1848: x. Type: *Sparasion frontalis* Latreille, by substitution of *Bebelus* for *Oxyurus* Lamarck. Replacement name for *Oxyurus* Lamarck.

*Prosparasion* Kieffer, 1913: 190. Type: *Prosparasion coeruleum* Kieffer, by monotypy and original designation. Synonymized by Masner (1976).

In this paper we do not deal with the generic concept and diagnosis of the genus as these have been dealt with comprehensively by Johnson et al. [4].

### New species groups

We propose two new species groups - the *Sparasion bilahari* species group and the *Sparasion manavati* species group - based on distinctive morphological characters enabling the inclusion of all but one of the currently known Oriental species in one of these groups.

### *Sparasion bilahari* species group (e.g. Figs. 1, 2, 3 and 4)

**Fig. 1:**
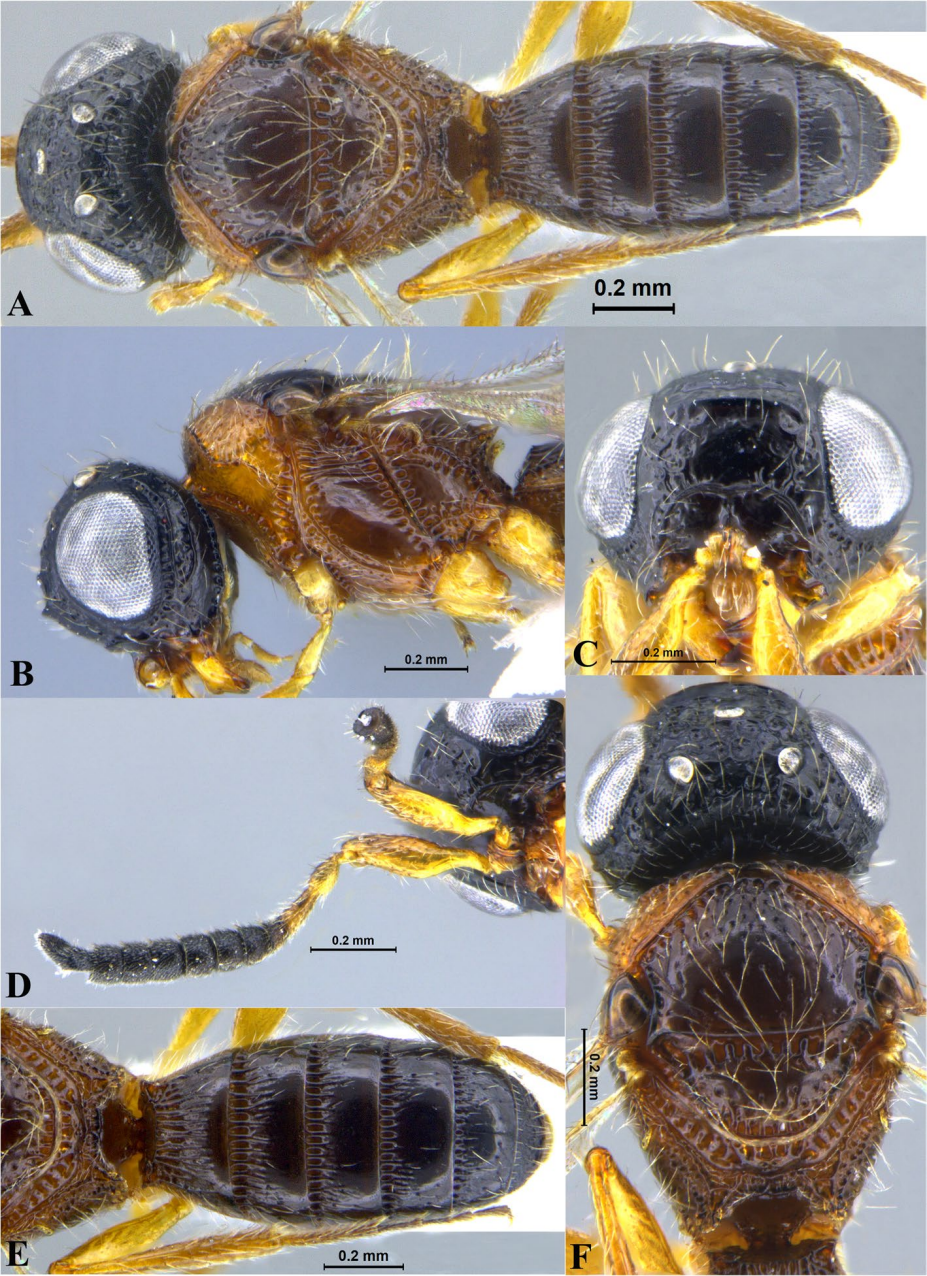
*Sparasion bhairavi ***sp. n**., female holotype. **A** Habitus, dorsal view. **B** Head and pleuron. **C** Frons. **D** Antennae. **E** Metasoma. **F** Head and mesonotum

**Fig. 2:**
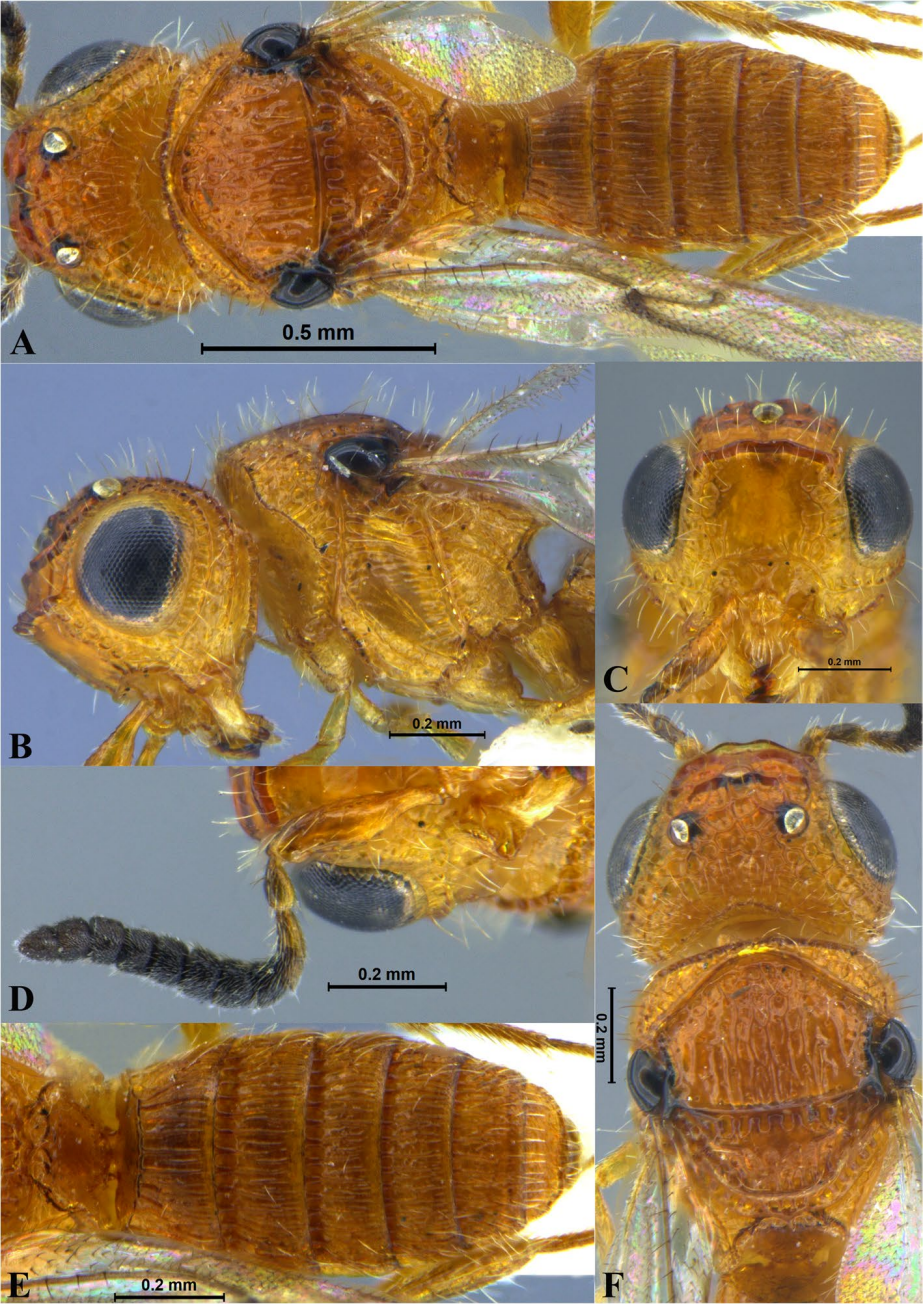
*Sparasion bhupali ***sp. n**., female holotype. **A** Habitus, dorsal view. **B** Head and pleuron. **C** Frons. **D** Antenna. **E** Metasoma. **F** Head and mesonotum

**Fig. 3:**
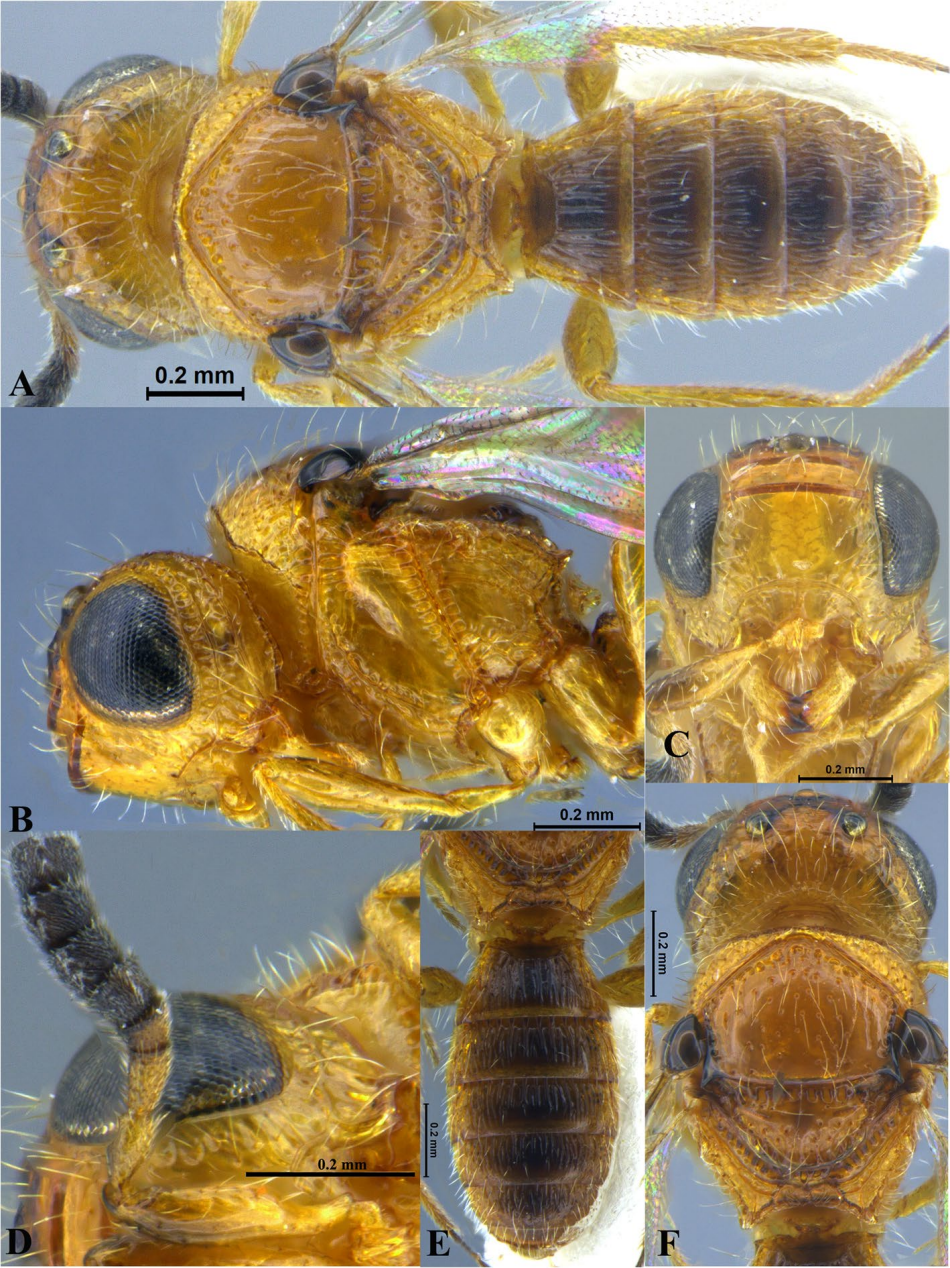
*Sparasion bihagi ***sp. n**., female holotype. **A** Habitus, dorsal view. **B** Head and pleuron. **C** Frons. **D** Antenna. **E** Metasoma. **F** Head and mesonotum

**Fig. 4:**
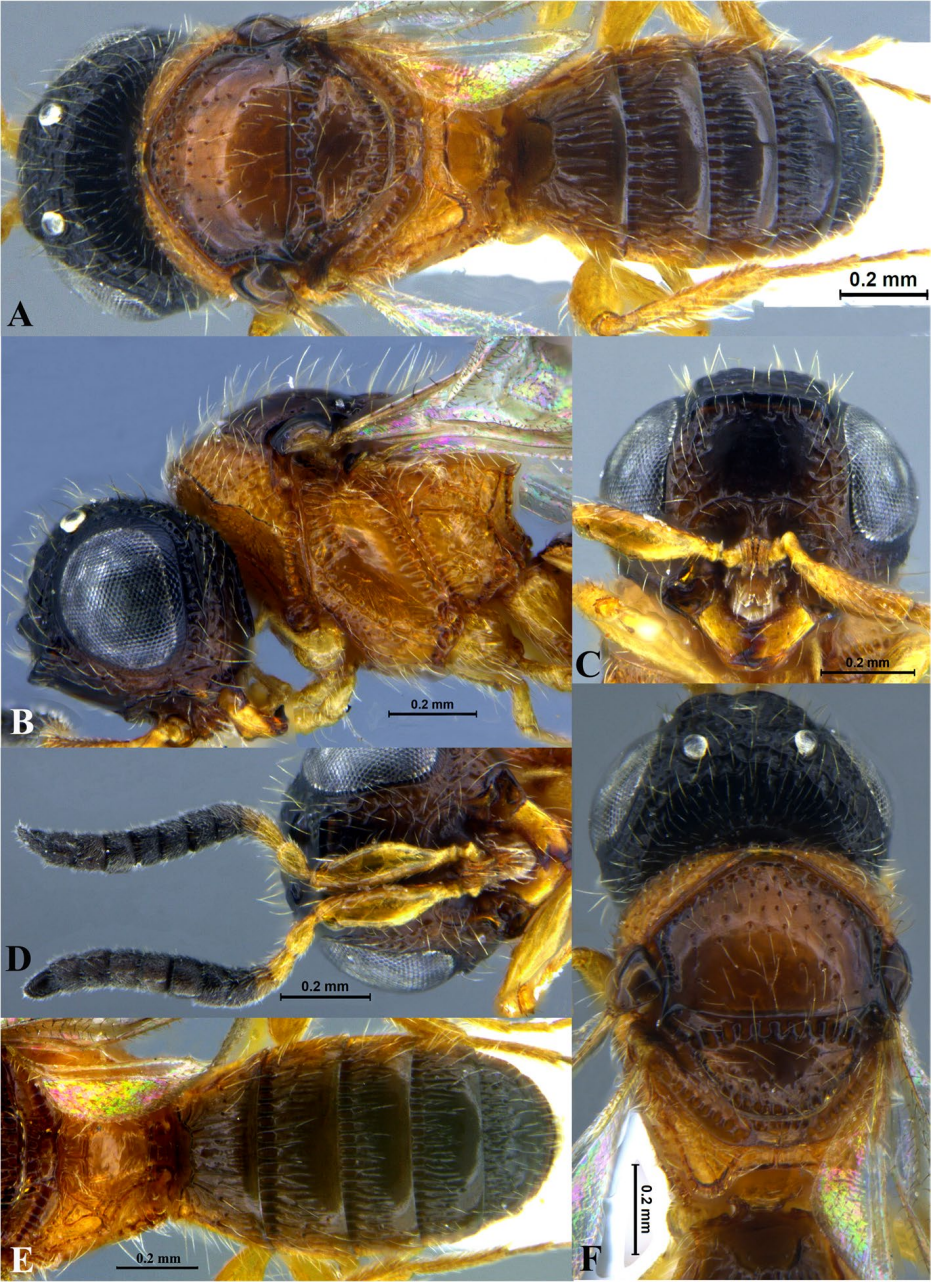
*Sparasion bilahari ***sp. n**., female holotype. **A** Habitus, dorsal view. **B** Head and pleuron. **C** Frons. **D** Antennae. **E** Metasoma. **F** Head and mesonotum

#### Diagnosis

Two to three transverse ledges present on upper frons; A1 smooth with sparse setae; A3 short, at most 1.4 × the length of A2 (in females); genal carina present; body colour either yellow, orange-brown or black; all legs xanthic; radialis curving upwards distally.

### Description

#### General

Body size: medium. Body length: 2.1–3.5 mm. Colour: yellow, orange-brown or black, with xanthic mandibles, A1–A3 and legs. Wings: weakly infuscate.

#### Head

Setation on head: sparse. Lateral ocellus: away from orbit. Anterior margin of frons: arcuate, rarely sinuous. Number of transverse ledges on upper frons: 2–3. Sculpture of upper frons: smooth or with polygonal cells. Sculpture of lower frons: either smooth with polygonal cells or entirely with polygonal cells. Sculpture on vertex: with sparse or dense polygonal cells. Sculpture of posterior orbital furrow: with foveae and depressions. Genal carina: present. Sculpture on A1: smooth with sparse setae. Length of A3: short, at most 1.4 × the length of A2 (in females).

#### Mesosoma

Sculpture of dorsal pronotum: sculptured with dense setae. Sculpture of mesoscutum: either setigerous punctate or foveate or longitudinally carinate. Notaulus: present or absent. Sculpture of notaulus: foveate or with depressions. Mesoscutal humeral sulcus: foveate. Mesoscutal suprahumeral sulcus: foveate. Parapsidal line: indicated as a furrow. Scutoscutellar sulcus: foveate. Sculpture of mesoscutellum: partially smooth with polygonal cells. Sculpture of dorsellum: anteriorly foveate and posteriorly smooth. Posterior propodeal projection: rounded or projecting. Epomial carina: present. Netrion: absent. Speculum of mesopleuron: transversely carinate. Episternal sulcus: not foveate. Sculpture of femoral depression: smooth. Mesopleural pit: present. Wings: weakly infuscate. Radialis: curving upwards distally.

#### Metasoma

Anterior margin of T1: weakly convex or straight. Sculpture of T1–T5: longitudinally costate or smooth or both.

### *Sparasion manavati* species group (e.g. Figs. 5, 6, 7, 8, 9, 10, 11, 12, 13, 14 and 15)

**Fig. 5:**
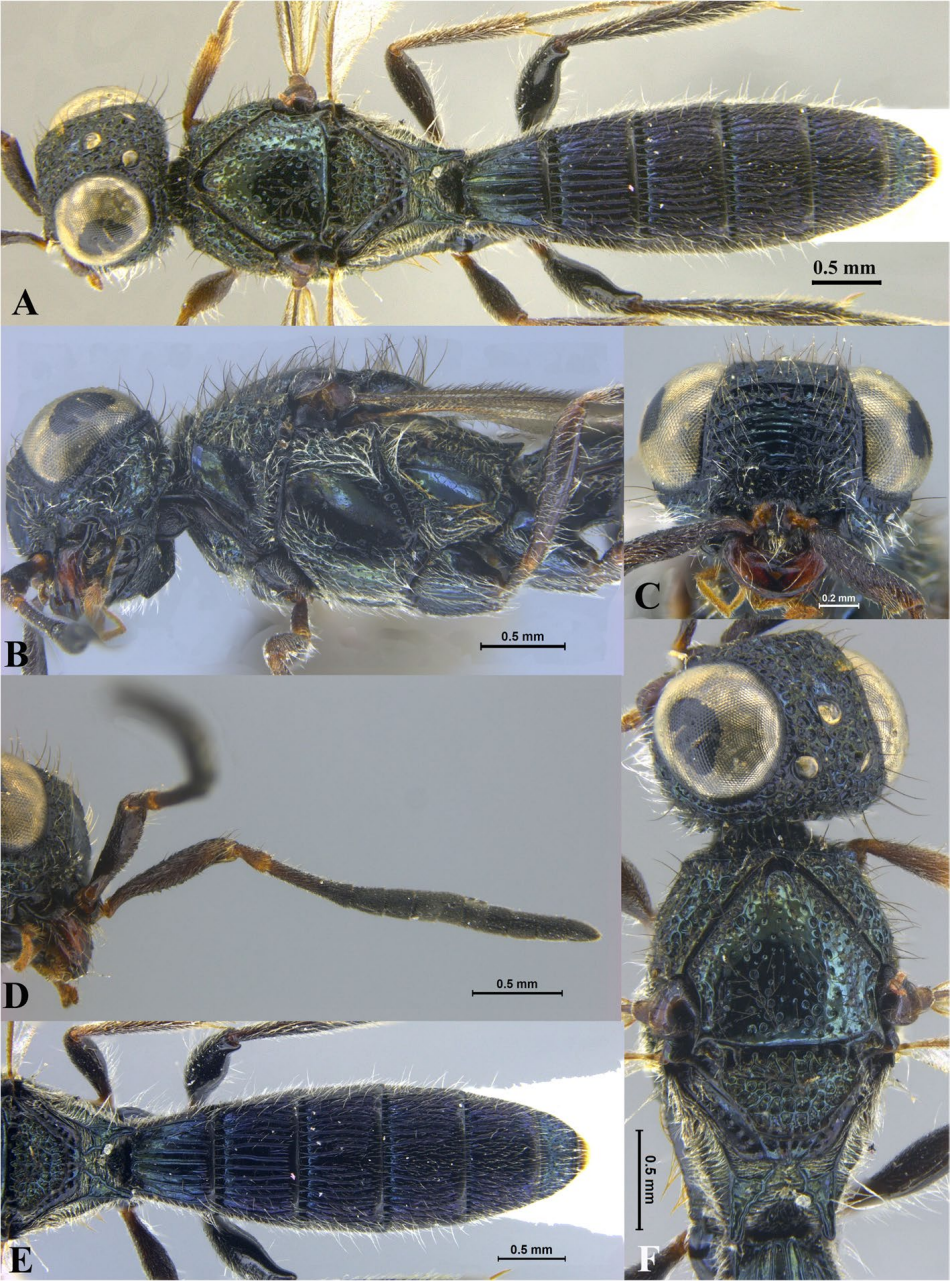
*Sparasion pahadi ***sp. n**., female holotype. **A** Habitus, dorsal view. **B** Head and pleuron. **C** Frons. **D** Antennae. **E** Metasoma. **F** Head and mesonotum

**Fig. 6:**
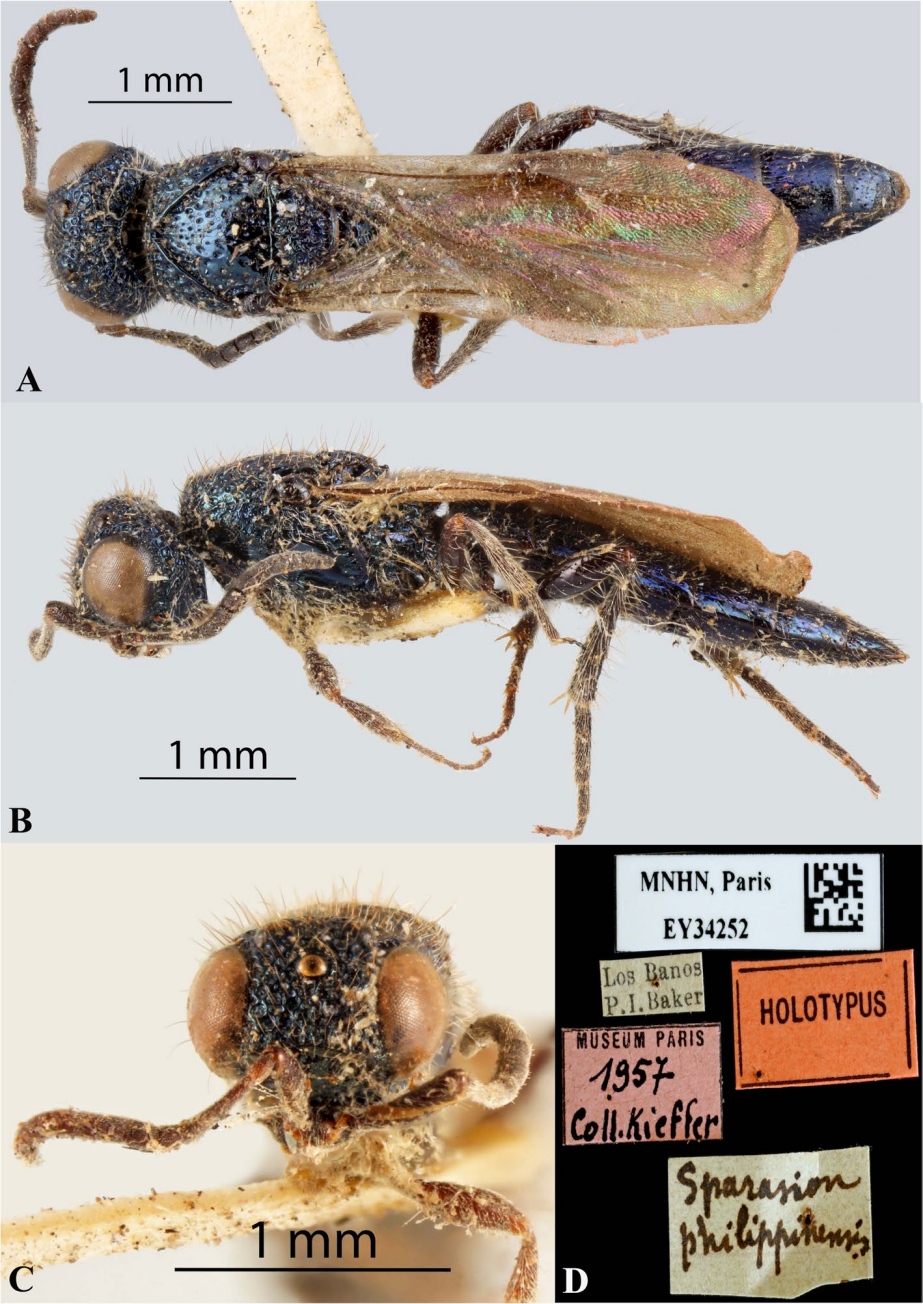
*Sparasion philippinensis* Kieffer*,* female holotype. **A** Habitus, dorsal view. **B** Habitus, lateral view. **C** Frons. **D** Type labels (Photos: Dr. Agnièle Touret-Alby ©, MNHN)

**Fig. 7:**
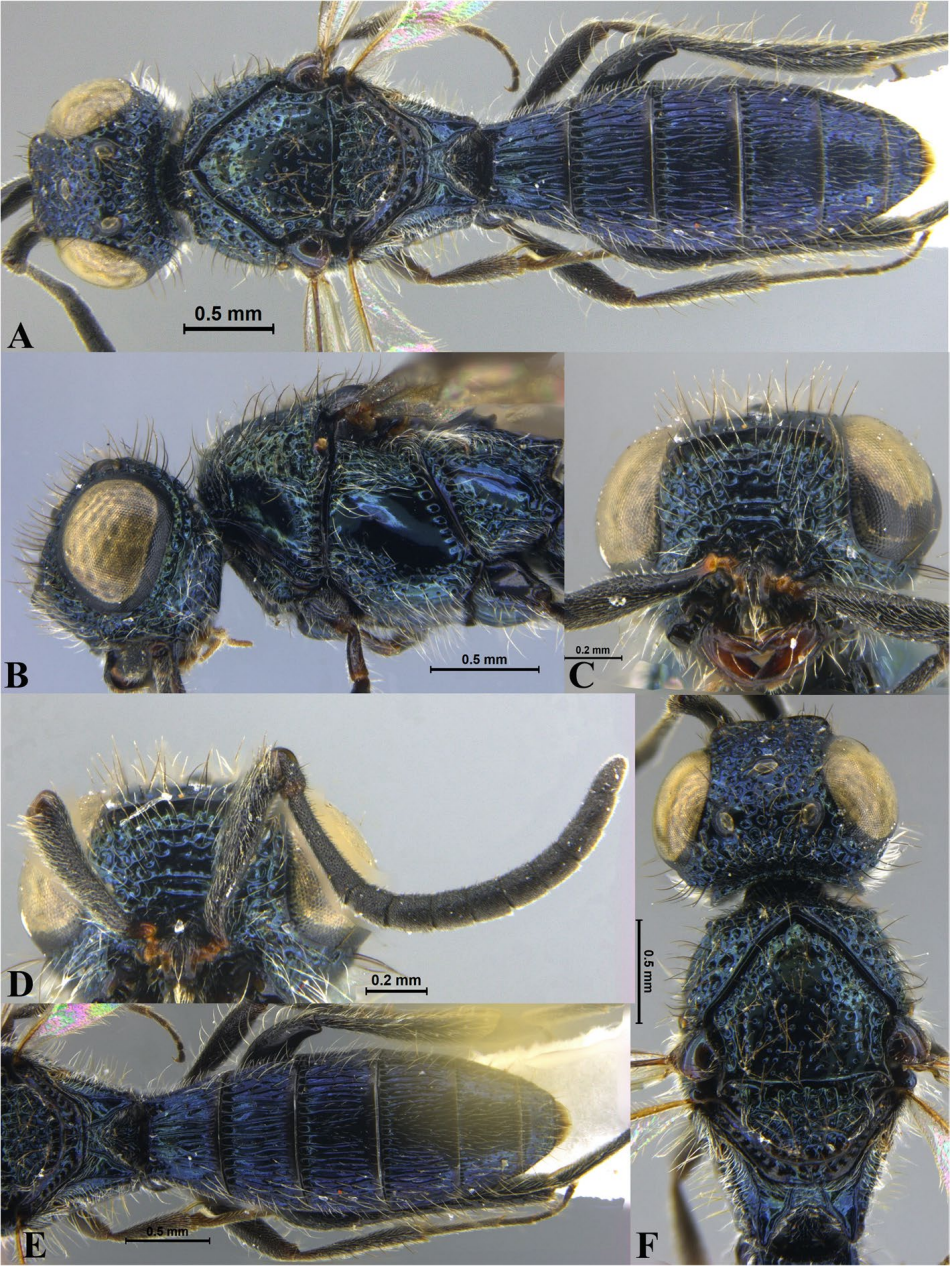
*Sparasion ratnangi ***sp. n**., female holotype. **A** Habitus, dorsal view. **B** Head and pleuron. **C** Frons. **D** Antenna. **E** Metasoma. **F** Head and mesonotum

**Fig. 8:**
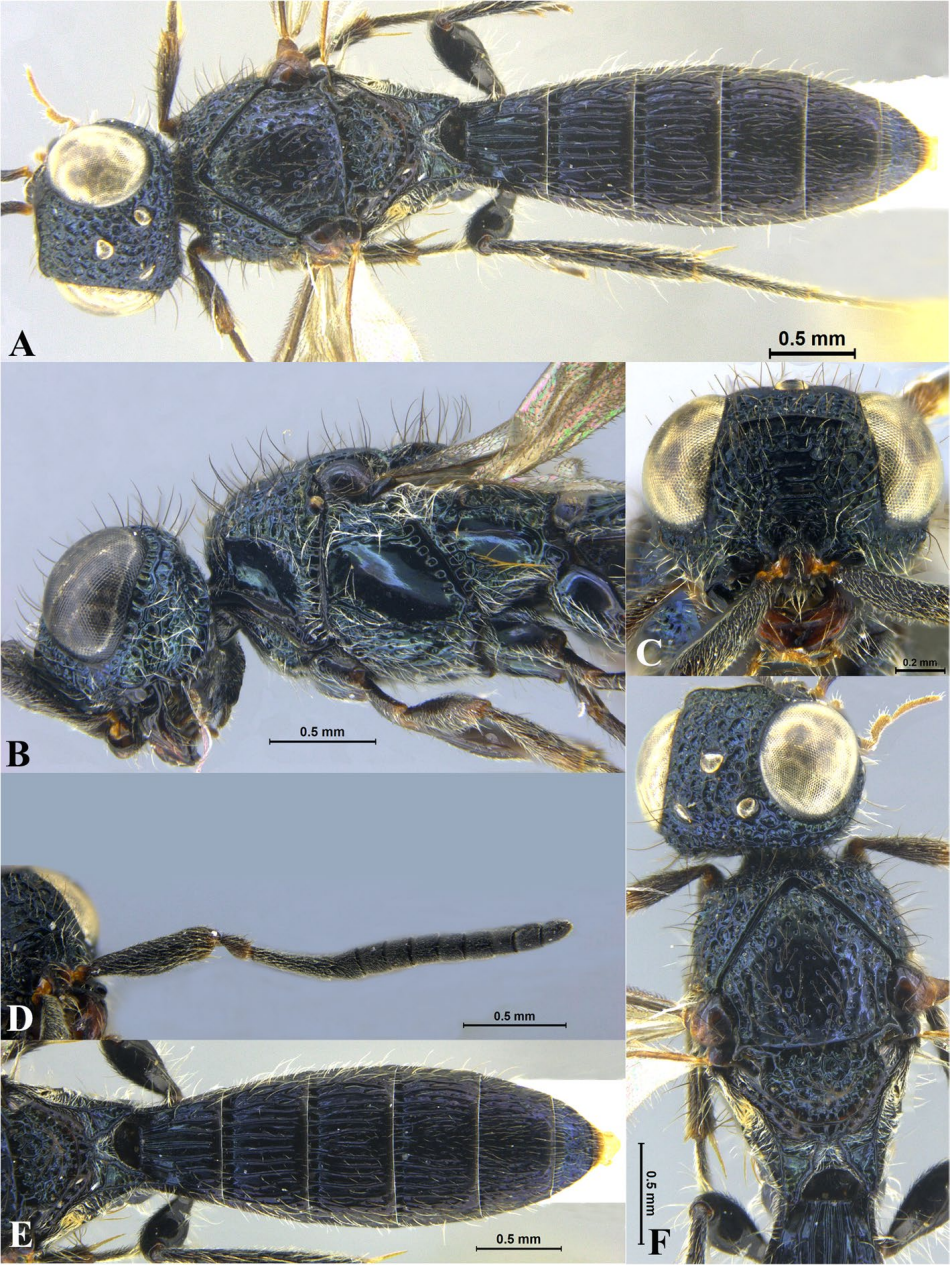
*Sparasion rupavati ***sp. n**., female holotype. **A** Habitus, dorsal view. **B** Head and pleuron. **C** Frons. **D** Antenna. **E** Metasoma. **F** Head and mesonotum

**Fig. 9:**
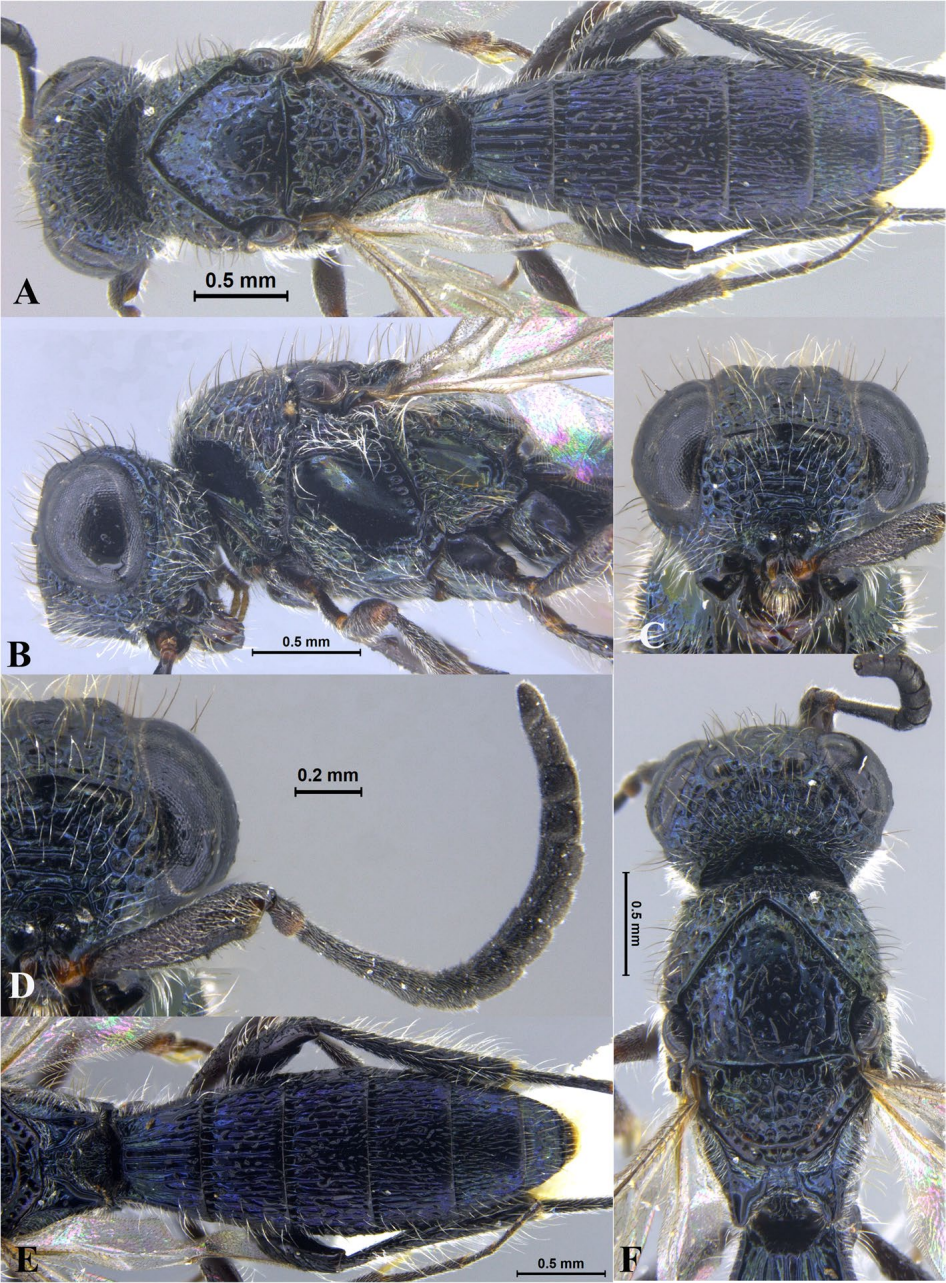
*Sparasion salagami ***sp. n**., female holotype. **A** Habitus, dorsal view. **B** Head and pleuron. **C** Frons. **D** Frons and antenna. **E** Metasoma. **F** Head and mesonotum

**Fig. 10:**
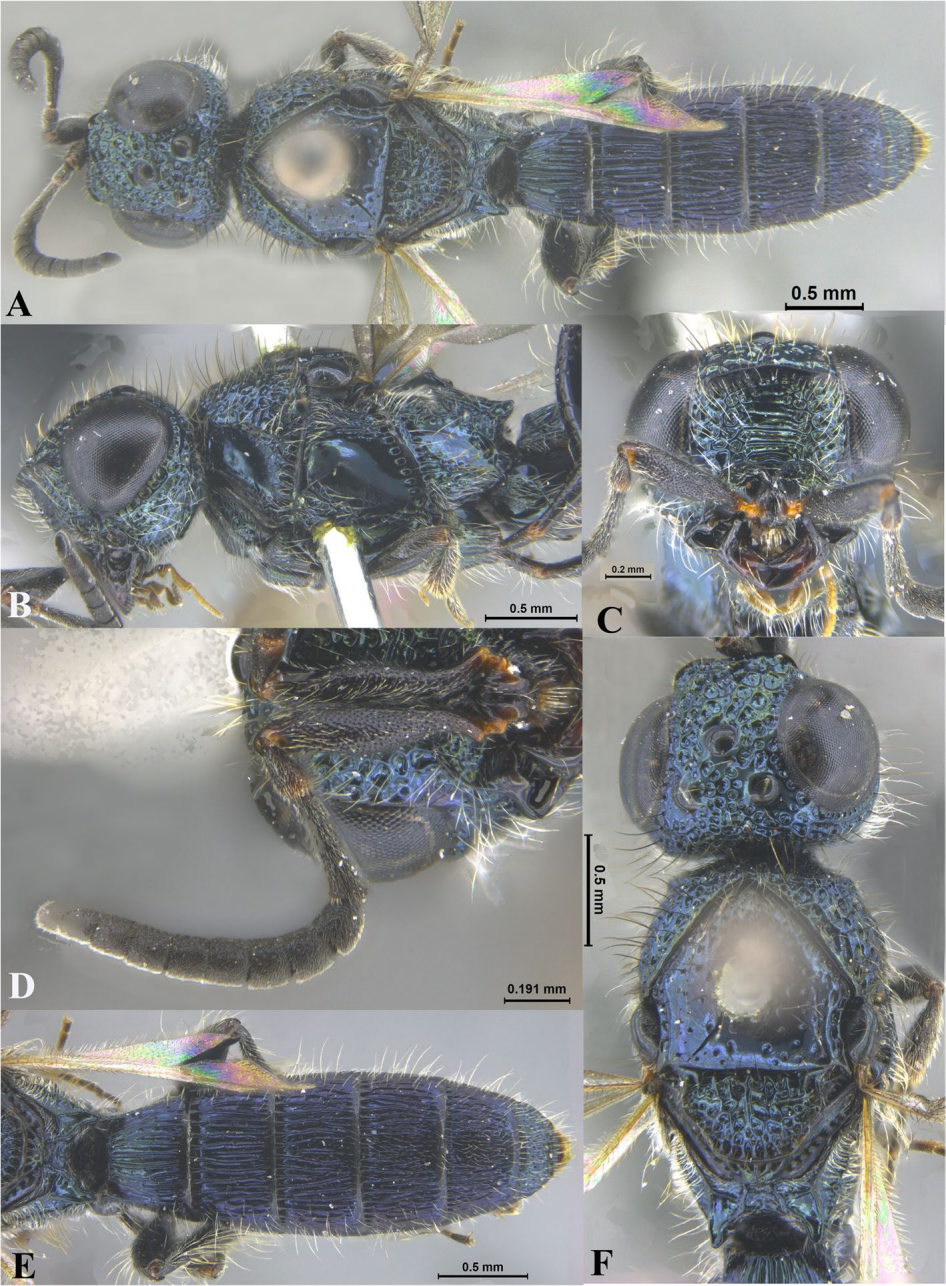
*Sparasion shulini ***sp. n**., female holotype. **A** Habitus, dorsal view. **B** Head and pleuron. **C** Frons. **D** Frons and antenna. **E** Metasoma. **F** Head and mesonotum

**Fig. 11:**
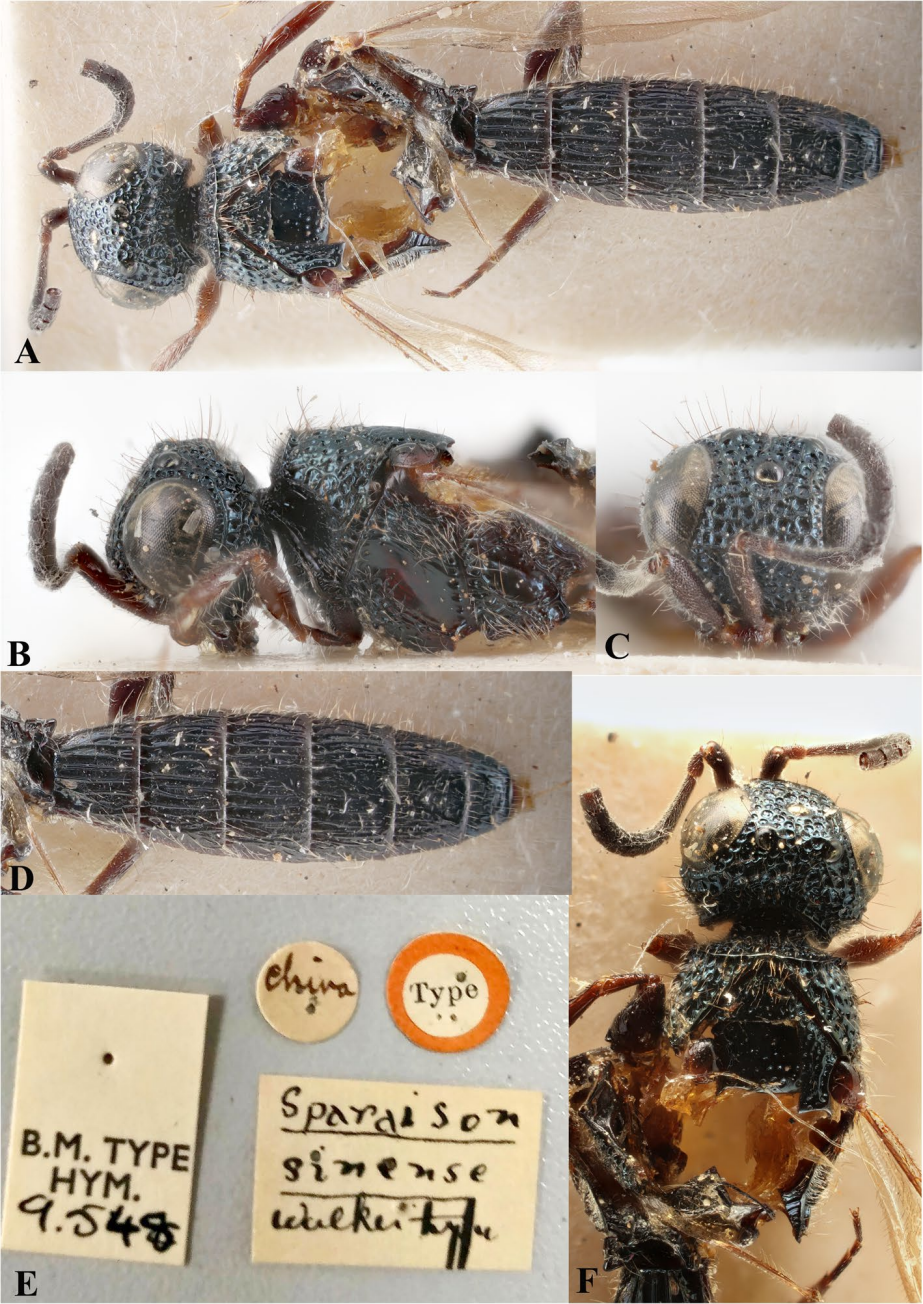
*Sparasion sinensis* Walker, female holotype. **A** Habitus, dorsal view. **B** Head and pleuron. **C** Frons and antenna. **D** Metasoma. **E** Type labels. **F** Head, antennae and mesonotum (Photos: Dr. Andrew Polaszek © NHMUK)

**Fig. 12:**
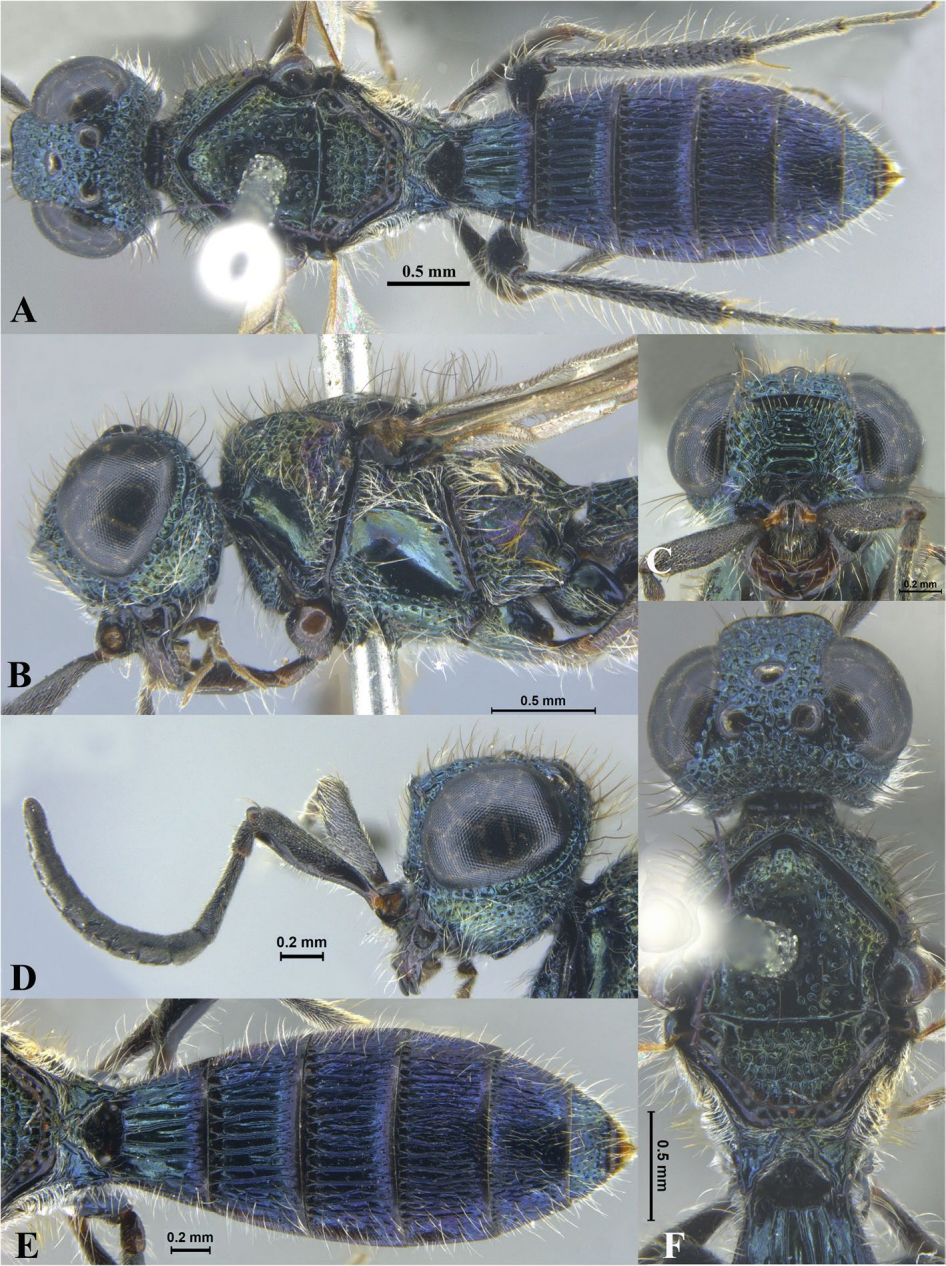
*Sparasion sivaranjini ***sp. n**., female holotype. **A** Habitus, dorsal view. **B** Head and pleuron. **C** Frons. **D** Antenna. **E** Metasoma. **F** Head and mesonotum

**Fig. 13:**
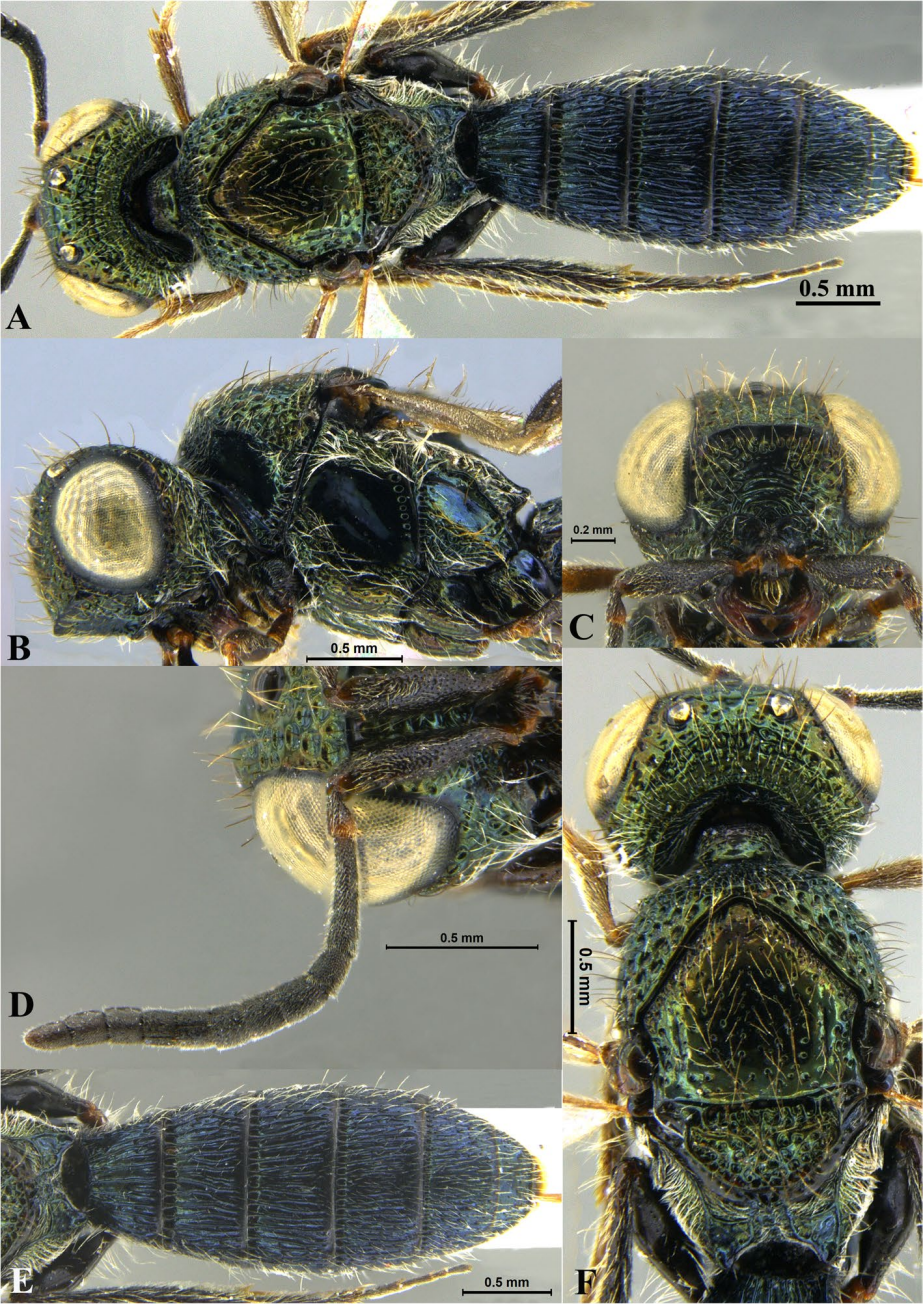
*Sparasion syamalangi ***sp. n**., female holotype. **A** Habitus, dorsal view. **B** Head and pleuron. **C** Frons. **D** Antenna. **E** Metasoma. **F** Head and mesonotum

**Fig. 14:**
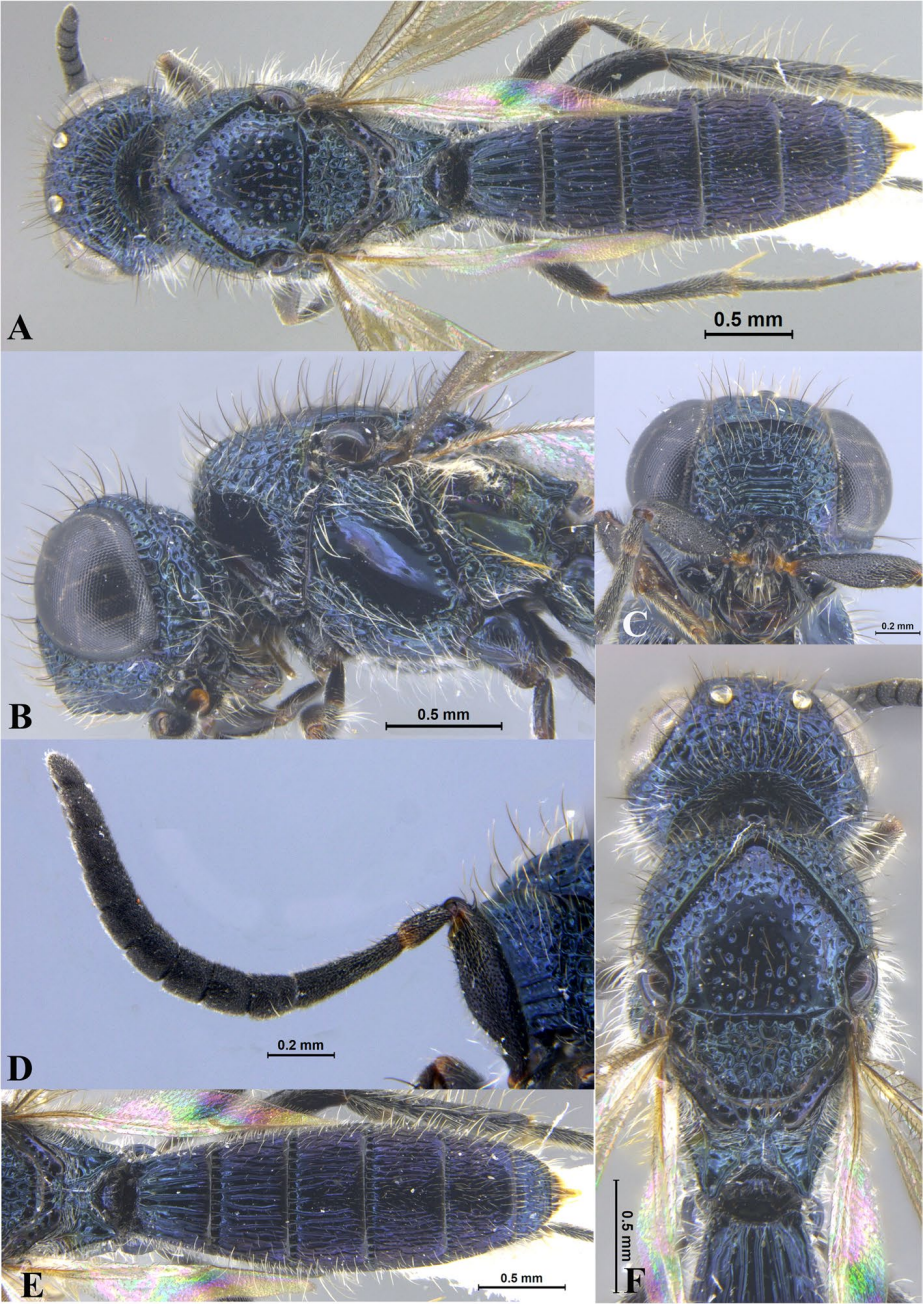
*Sparasion todi ***sp. n**., female holotype. **A** Habitus, dorsal view. **B** Head and pleuron. **C** Frons. **D** Antenna. **E** Metasoma. **F** Head and mesonotum

**Fig. 15:**
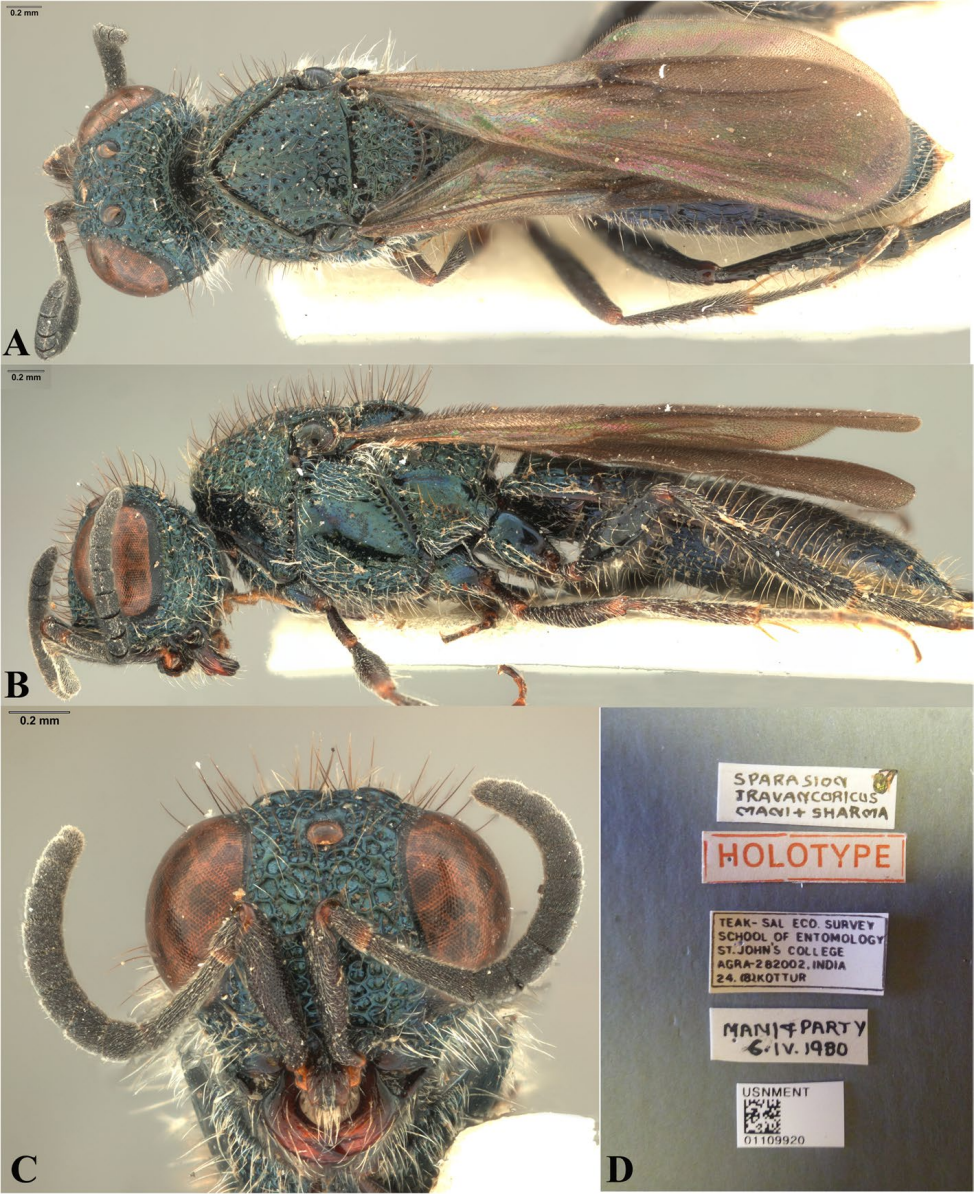
*Sparasion travancoricus* Mani and Sharma, female holotype. **A** Habitus, dorsal view. **B** Habitus, lateral view. **C** Frons and antennae. **D** Type labels (Photos: Dr. N. F. Johnson © Ohio State University)

#### Diagnosis

A single transverse ledge present on upper frons; A1 with dense setigerous punctae; A3 elongate, > 2.2 × the length of A2 (in females); genal carina absent; body colour steel blue or green, all legs black-brown; radialis almost straight.

#### General

Body size: large and robust. Body length: 5.2–9 mm. Colour: steel blue or green, with black-brown legs. Wings: strongly infuscate.

#### Head

Setation on head: dense. Lateral ocellus: away from orbit. Anterior margin of frons: arcuate with or without medial indentation. Number of transverse ledges on upper frons: one. Sculpture of upper frons: with polygonal cells or foveae, with smooth interstices. Sculpture of lower frons: with or without medial transverse carinae surrounded by polygonal cells. Sculpture on vertex: with polygonal cells. Sculpture of posterior orbital furrow: with foveae and depressions. Genal carina: absent. Sculpture on A1: densely setigerous punctate. Length of A3: elongate, > 2.2 × the length of A2 (in females).

#### Mesosoma

Sculpture of dorsal pronotum: highly sculptured with dense setae. Sculpture of mesoscutum: either setigerous punctate or foveate. Notaulus: present or absent. Sculpture of notaulus: foveate. Mesoscutal humeral sulcus: foveate. Mesoscutal suprahumeral sulcus: foveate. Parapsidal line: indicated as a furrow. Scutoscutellar sulcus: foveate. Sculpture of mesoscutellum: with compact polygonal cells bearing setae. Sculpture of dorsellum: anteriorly foveate and posteriorly smooth. Posterior propodeal projection: rounded or projecting. Epomial carina: present. Netrion: absent. Speculum of mesopleuron: transversely carinate or foveate or both. Episternal sulcus: not foveate. Sculpture of femoral depression: smooth. Mesopleural pit: present. Wings: highly infuscate; narrow and elongate. Radialis: almost straight.

#### Metasoma

Anterior margin of T1: weakly convex or straight. Sculpture of T1–T5: with longitudinal costae or smooth or both.

## Description of species

### *Sparasion albopilosellus* Cameron (Figs. 16A–F, 17A, 18A, 19)

**Fig. 16:**
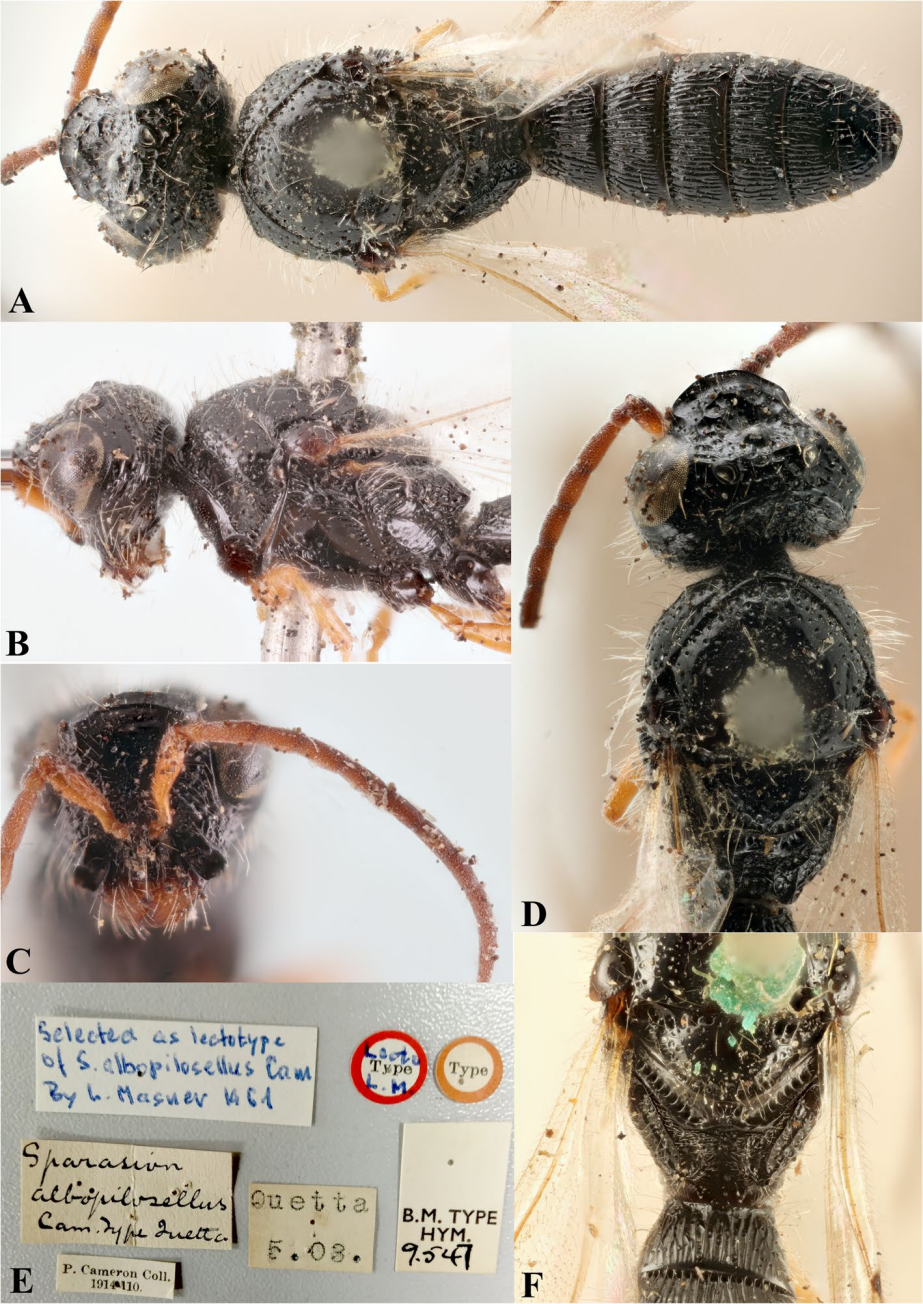
*Sparasion albopilosellus* Cameron, male holotype. **A** Habitus, dorsal view. **B** Head and pleuron. **C** Frons and antennae. **D** Head and mesonotum. **E** Type labels. **F** Mesoscutellum, dorsellum and T1 (Photos: Dr. Andrew Polaszek © NHMUK)

**Fig. 17:**
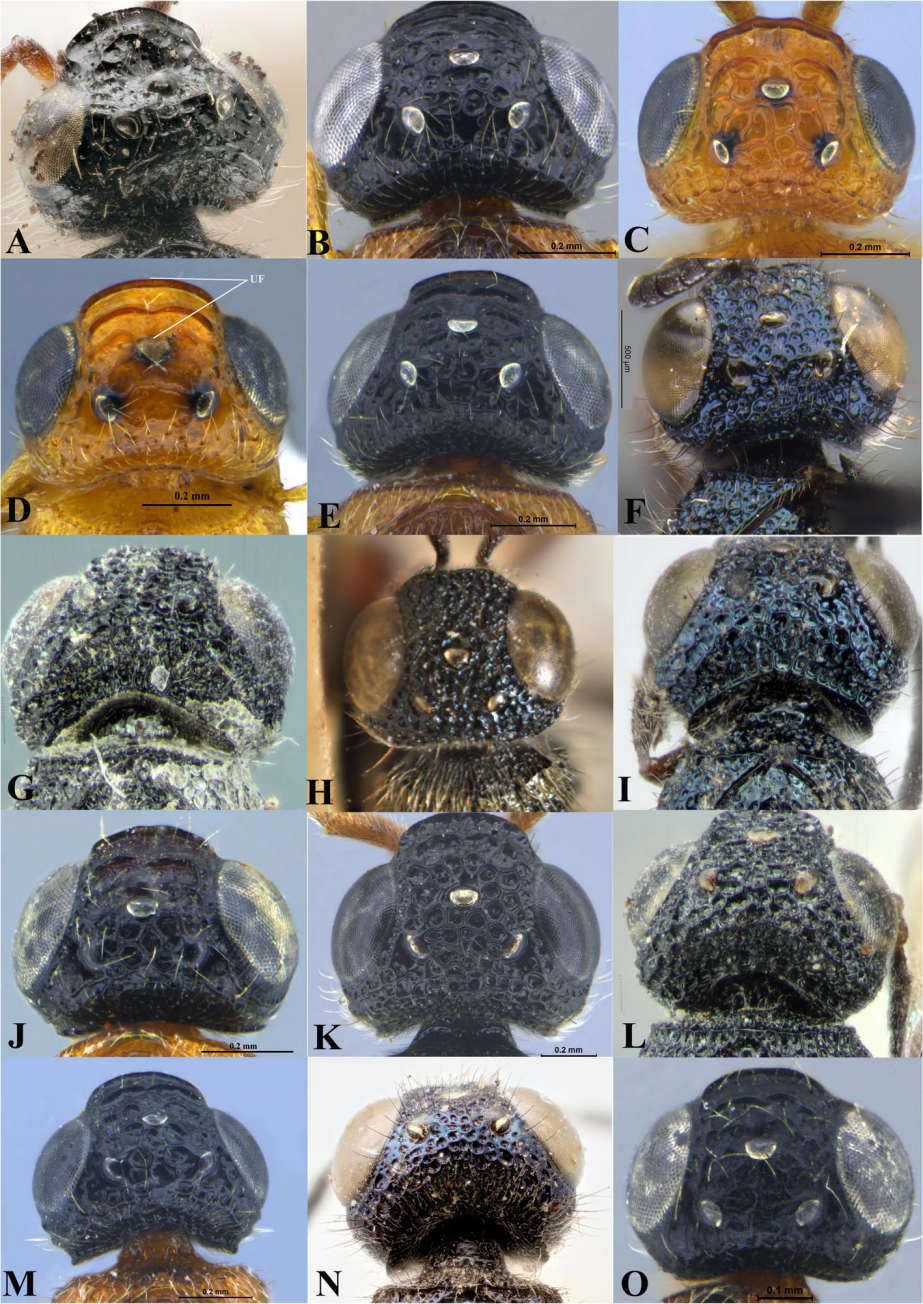
Head, dorsal view. **A ***Sparasion albopilosellus*. **B ***S. bhairavi*. **C ***S. bhupali*. **D ***S. bihagi*. **E ***S. bilahari*. **F ***S. cellularis*. **G ***S. coconcus. ***H ***S. coeruleus*. **I ***S. cullaris*. **J ***S. darbari. ***K ***S. deepaki. ***L ***S. domes. ***M ***S. elbakyanae. ***N ***S. formosus*. **O ***S. hindoli.* UF-upper frons

**Fig. 18:**
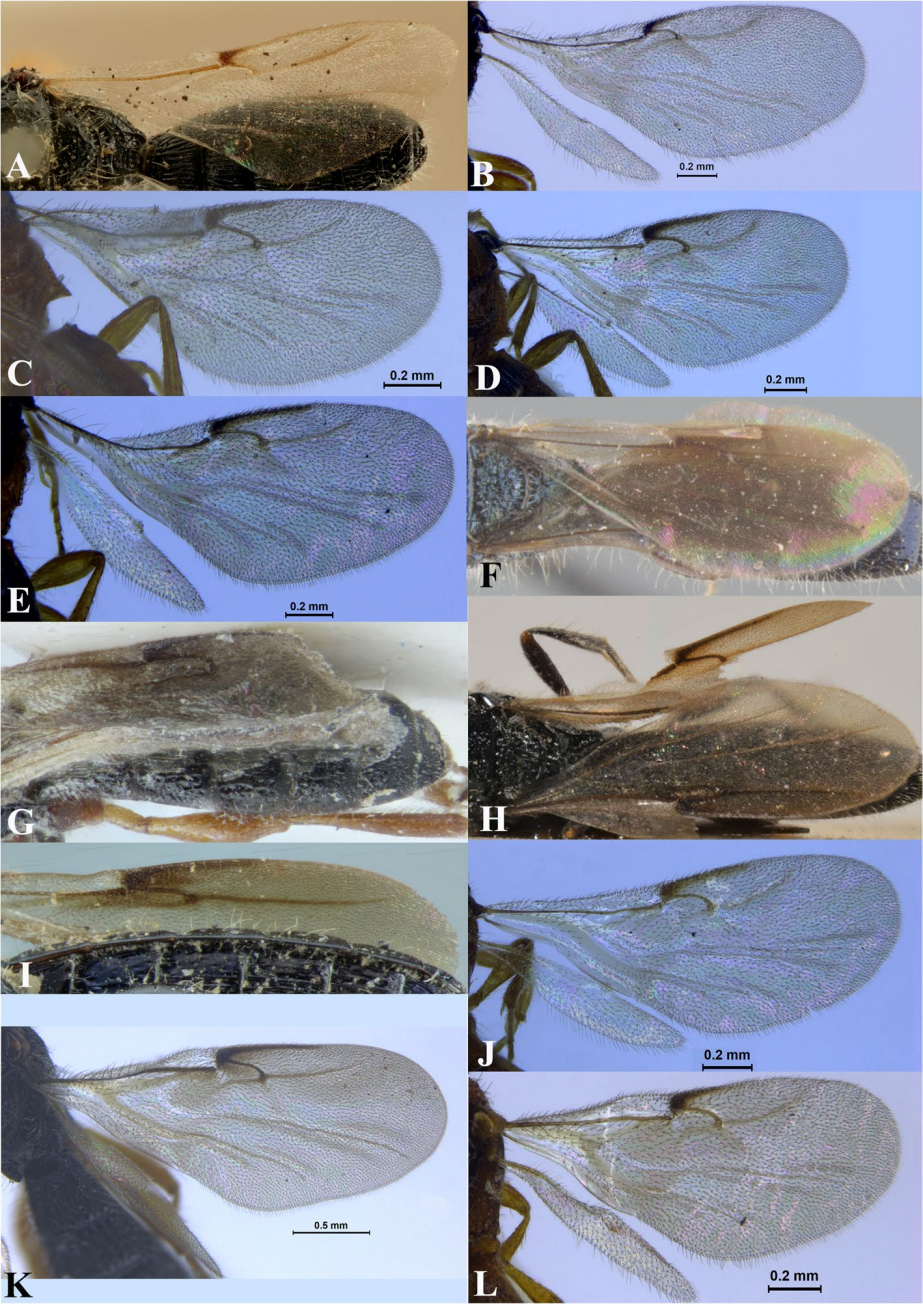
Wings. **A*** Sparasion albopilosellus ***B ***S. bhairavi*. **C ***S. bhupali*. **D ***S. bihagi*. **E ***S. bilahari*. **F ***S. cellularis*. **G ***S. coconcus. ***H ***S. coeruleus*. **I ***S. cullaris*. **J ***S. darbari. ***K ***S. deepaki. ***L ***S. elbakyanae*

**Fig. 19:**
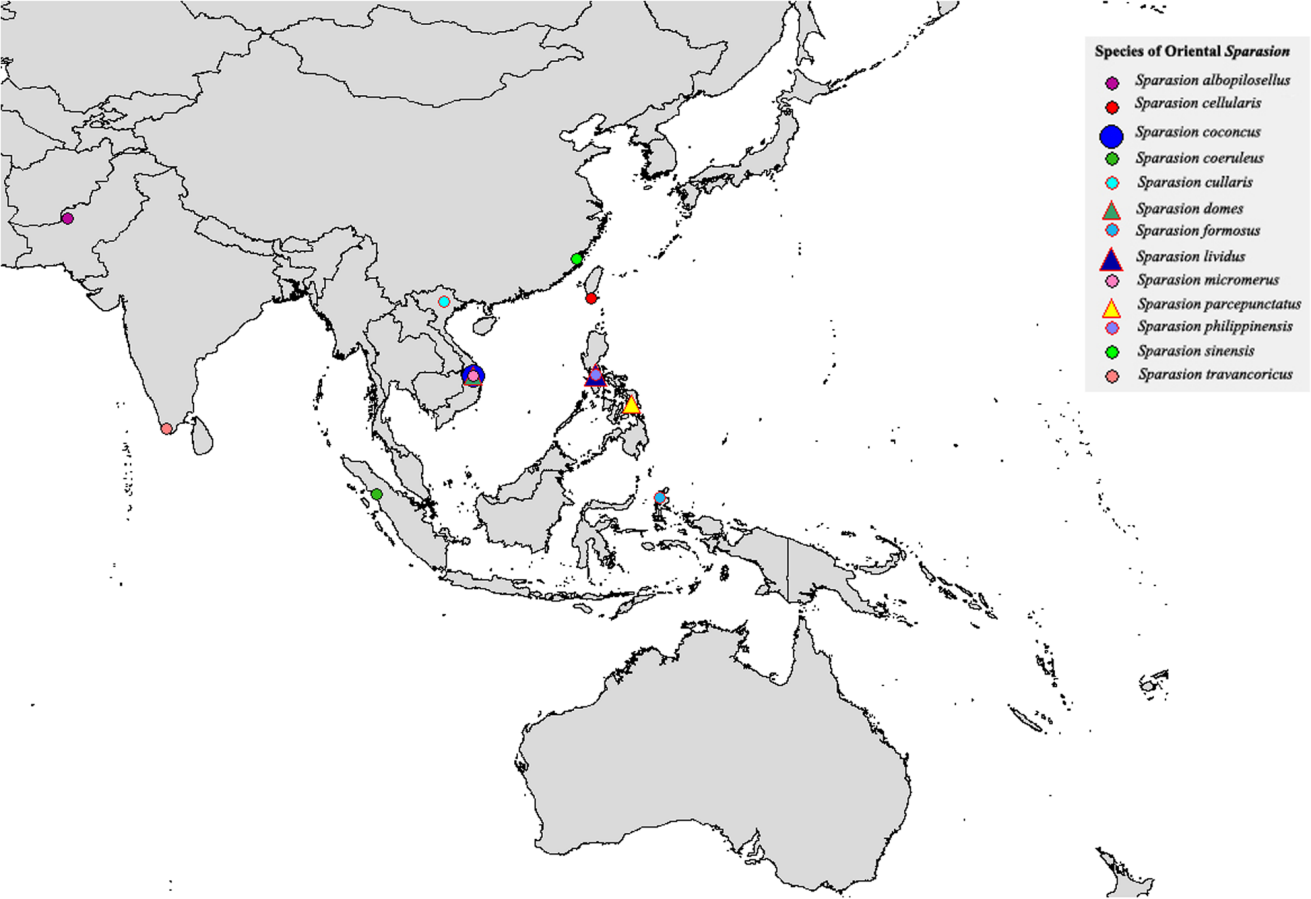
Map of previously recorded localities of *Sparasion* in the Oriental region

*Sparasion albo-pilosellus* Cameron, 1906: 98.

*Sparasion albopilosellus*: Kieffer, 1926:283, 292. Description, keyed

*Sparasion albopilosellus*: Masner, 1965a: 96. Lectotype designation

#### Diagnosis

Upper frons with oblique carinae anterior to lateral ocellus in addition to a transverse carina between lateral ocelli; orbital carina absent; dorsal and ventral metapleural area transversely carinate: distinctive to this species among Oriental *Sparasion*.

#### Material examined

Lectotype: Male, (B. M. Type, HYM 9.547), **PAKISTAN**: Baluchistan, Quetta, 3.V.1914; leg. P. Cameron.

### Description

Male body length = 4.5 mm.

#### Colour

Head, mesosoma and metasoma black; tegula black with brown patches; fore coxa orange-brown, mid- and hind- coxae black-brown, remainder of leg orange-brown; entire antenna orange-brown; mandibles orange-brown with apical margins of teeth brown.

#### Head

Setation on head: sparse. Setation of compound eye: sparsely setose. Anterior margin of frons: arcuate. Distance from level of anterior margin of compound eyes to anterior extension of frons in dorsal view: 1.8 × MOD. Number of transverse ledges on upper frons: three. Sculpture of upper frons: anteriorly smooth followed by a transverse row of shallow polygonal cells and a semicircular smooth area with sparse punctae. Sculpture of lower frons: smooth with polygonal cells laterally. Transverse carina above interantennal process: medially notched. Area ventral to transverse carina above interantennal process: smooth with setigerous punctae. Sculpture on vertex: obliquely carinate on anterolateral margin of lateral ocellus, with a transverse carina and shallow polygonal cells between lateral ocelli, followed by uneven transverse carinae interspersed with setigerous punctae posteriorly; smooth area present around anterior ocellus with sparse oblique carinae posteriorly; smooth area with sparse setigerous punctae and oblique carinae present posterior to lateral ocellus. Sculpture of posterior orbital furrow: smooth setigerous punctae. Genal carina: absent. Sculpture of gena: smooth with sparse punctae and longitudinal carinae. Sculpture of A1: smooth with sparse setae. A1: 3.9 × as long as wide. A3: 0.6 × length of A1 and 2 × length of A2.

#### Mesosoma

Sculpture of dorsal pronotum: smooth with sparse punctae. L: W of mesoscutum: 75:80. Sculpture of mesoscutum: smooth with sparse punctae. Notaulus: absent. Mesoscutal humeral sulcus: punctate. Mesoscutal suprahumeral sulcus: foveate. Parapsidal line: indicated as furrow. Scutoscutellar sulcus: foveate. L: W of mesoscutellum: 26:45. Sculpture of mesoscutellum: smooth with sparse punctae. Sculpture of dorsellum: anteriorly foveate, posteriorly smooth, posterior margin almost straight. Sculpture of outer lateral propodeal area: with shallow depressions, sparsely setose; Sculpture of inner lateral propodeal area: smooth, with sparse pilosity anteriorly, posterior half divided into three shallow depressions. Lateral propodeal carina: curving inwards medially. Posterior propodeal projection: rounded, not extending to anterior margin of T1. Sculpture of metasomal depression: transversely carinate with intricate sculpture in between. Plical area: anteriorly setose and posteriorly with depressions. Sculpture of propleuron: smooth. Sculpture of lateral pronotal area: smooth, with sparse longitudinal carinae anteriorly. Posterior pronotal sulcus: foveate. Pronotal cervical sulcus: foveate. Speculum of mesopleuron: transversely carinate interspersed with sparse punctae, sparsely setose. Prespecular sulcus: foveate. Mesepimeral sulcus: foveate. Mesepimeral area: smooth, wider than mesepimeral sulcus. Mesopleural carina: present. Sculpture of femoral depression: smooth. Mesopleural pit: present. Sculpture of ventral mesopleuron: smooth with sparse setigerous punctae. Sculpture of metapleuron: dorsal metapleural area transversely carinate interspersed with punctae and a row of long setae on anterior margin; ventral metapleural area dorsally smooth, anteroventrally foveate-punctate interspersed with transverse carinae and posteroventrally transversely carinate. Metapleural sulcus: weakly foveate. Paracoxal sulcus: with wide foveae. Metapleural epicoxal sulcus: foveate.

#### Fore wing

L: W: 310:113. Transparency: hyaline. Lengths of R: R1: r-rs in ratio of 133:107:28. Anterior margin of fore wing: weakly downcurved prior to R1.

#### Metasoma

L: W of metasoma: 188:85. Ratio of length of T1: T2: T3: T4: T5: 32:32:33:30:28. Anterior margin of T1: straight. Spine on anteromedial margin of T1: present. Sculpture of T1: entirely longitudinally costate except for smooth area with sparse punctae laterally and posterior smooth patch. Sculpture of T2: basal foveae present, followed by longitudinal costae except for smooth area with punctae laterally and posterior smooth patch. Sculpture of T3: same as T2, with larger basal foveae. Sculpture of T4: same as T2. Sculpture of T5: same as T2 with a semicircular smooth area posteriorly. Sculpture of T6: entirely smooth with sparse setigerous punctae except for irregular carinae sublaterally. Sculpture of T7: smooth with sparse setigerous punctae.

#### Female

Unknown.

#### Remarks

Lectotype preserved in good condition in NHM, London.

### *Sparasion bhairavi* Veenakumari sp. n. (Figs. 1A–F, 17B, 18B, 20A–B, 21A)

**Fig. 20:**
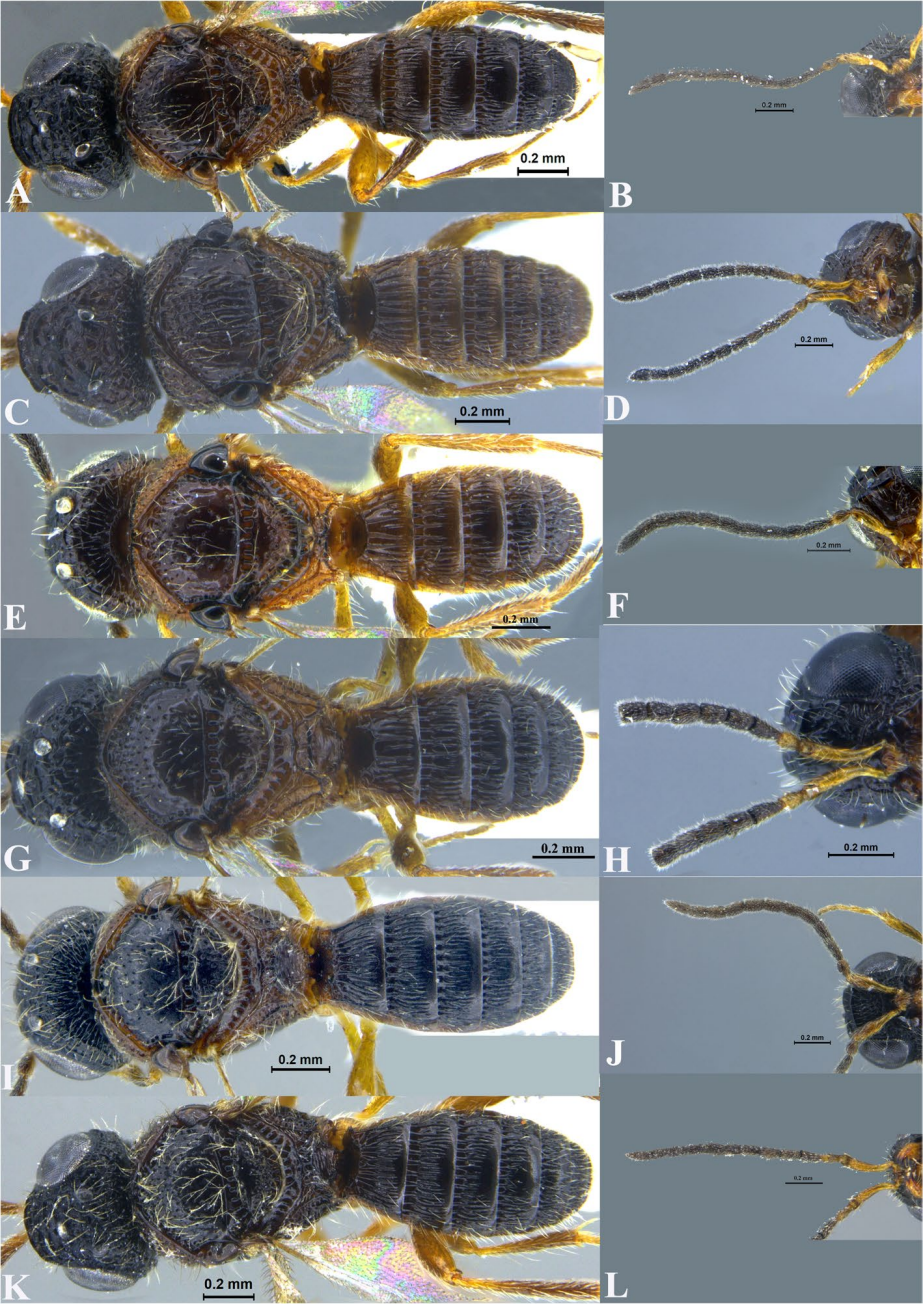
Males: *Sparasion bhairavi* - **A** Habitus. **B** Antenna; *S. bhupali* - **C** Habitus. **D** Antennae; *S. bihagi* - **E** Habitus. **F** Antenna; *S. bilahari* - **G** Habitus. **H** Antennae; *S. hindoli* - **I ***Habitus*. **J** Antenna; *S. kanakangi* - **K** Habitus*. ***L** Antenna

**Fig. 21:**
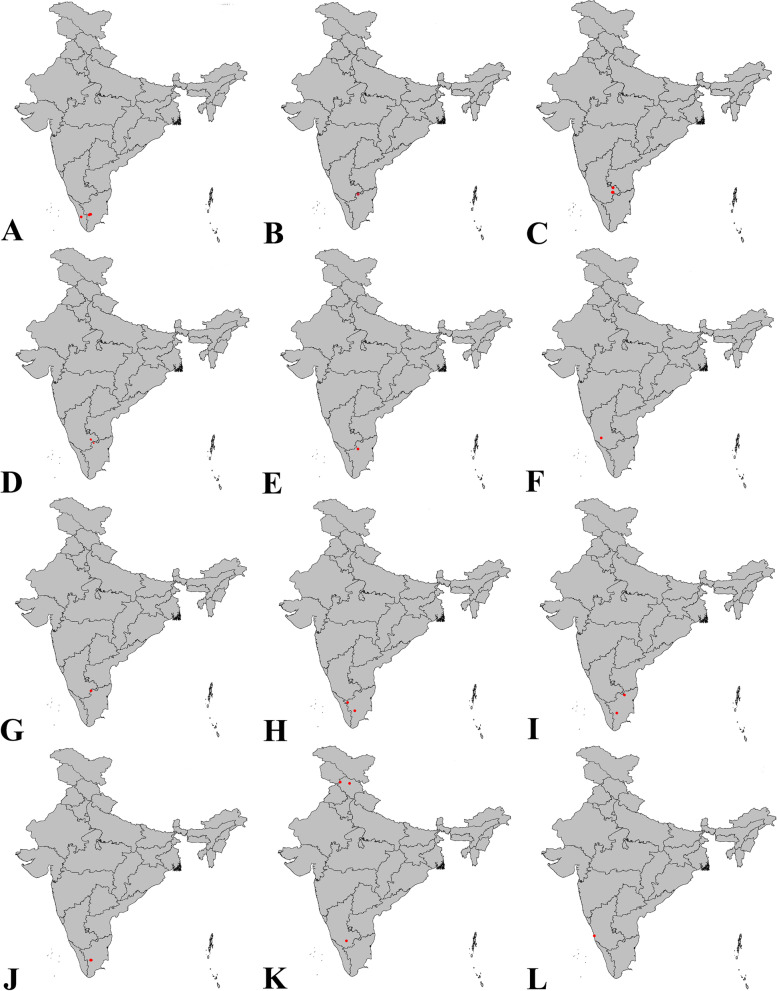
Distribution of new species of *Sparasion* in India: **A ***S. bhairavi ***sp. n**. **B ***S*. *bhupali ***sp. n**. **C ***S*. *bihagi ***sp. n**. **D ***S*. *bilahari ***sp. n**. **E ***S*. *darbari ***sp. n**. **F ***S*. *deepaki ***sp. n**. **G ***S*. *elbakyanae ***sp. n**. **H ***S*. *hindoli ***sp. n**. **I ***S*. *kalyani ***sp. n**. **J ***S*. *kanakangi ***sp. n**. **K ***S*. *karivadana ***sp. n**. **L ***S*. *manavati ***sp. n**

urn:lsid:zoobank.org:act:73A77B63-6971-461E-8BF2-EDB1758A4AD6

#### Diagnosis

*Sparasion bhairavi* sp. n. is close to *S. hindoli* sp. n. but differs from it in the following characters: in *S. bhairavi* sp. n. metasoma is narrow and elongate, foveae of scutoscutellar sulcus are incomplete and genal carina is basally curving towards orbital carina. Conversely, in *S. hindoli* sp. n. metasoma is short and wide, foveae of scutoscutellar sulcus are complete and genal carina is percurrent.

#### Material examined

Holotype: Female, (ICAR/NBAIR/P4675), **INDIA**: Tamil Nadu: Lower Pulney Hills, Thadiyankudisai, HRS, 10°17′58ʺN 77°42′42ʺE, 990 m, YPT in Citrus plot, 27.XI.2016. Paratypes: 1 female (ICAR/NBAIR/P4676), Tamil Nadu: Lower Pulney Hills, Thadiyankudisai, HRS, 10°17′58ʺN 77°42′42ʺE, 990 m, YPT in weeds, 30.I.2017; 1 female (ICAR/NBAIR/P4677), Tamil Nadu: Dindugul, Thandikudi, Regional Coffee Research Station (RCRS), 10°18′34ʺN 77°38′34ʺE, 1305 m, YPT in black pepper (*Piper nigrum*: Piperaceae) field, 27.XI.2016; 2 males (ICAR/NBAIR/P4678–P4679), Tamil Nadu: Lower Pulney Hills, Thadiyankudisai, HRS, 10°17′58ʺN 77°42′42ʺE, 990 m, YPT in grass, 26.XI.2016; 1 male (ICAR/NBAIR/P4680), Tamil Nadu: Lower Pulney Hills, Thadiyankudisai, HRS, 10°17′58ʺN 77°42′42ʺE, 990 m, YPT in weeds, 29.XI.2017; 3 males (ICAR/NBAIR/P4681–P4683), Tamil Nadu: Lower Pulney Hills, Thadiyankudisai, HRS, 10°17′58ʺN 77°42′42ʺE, 990 m, YPT in Citrus plot, 27.XI.2016; 2 males (ICAR/NBAIR/P4684–P4685), Tamil Nadu: Lower Pulney Hills, Thadiyankudisai, HRS, 10°17′58ʺN 77°42′42ʺE, 990 m, YPT, 25.XI.2016; 2 males (ICAR/NBAIR/P4686–P4687), Tamil Nadu: Kodaikanal, Shenbaganur, 10°14′01ʺN 77°30′47ʺE, 1865 m, YPT, 02.IV.2014; 1 male (ICAR/NBAIR/P4688), Tamil Nadu: Dindugul, Thandikudi, RCRS, 10°18′34ʺN 77°38′34ʺ, 1305 m, YPT, 28.XII.2016; 4 females (ICAR/NBAIR/P4711–P4714), Kerala: Palakkad, Mayiladumpara, 9°58′24ʺN 76°31′27ʺE, 99 m, YPT in weeds, 25.III.2017; 2 males (ICAR/NBAIR/P4715–P4716), Kerala: Palakkad, Mayiladumpara, 9°58′24ʺN 76°31′27ʺE, 99 m, YPT in weeds, 25.III.2017.

### Description

Female body length = 2.38–2.53 mm (*n* = 7); male body length = 2.41–2.62 mm (*n* = 8).

#### Colour

Head black; mesoscutum, mesoscutellum and lateral propodeal area brown-black; dorsellum, metanotal trough, meso and metapleuron honey brown; dorsal and lateral pronotum yellow-brown; transverse pronotal carina black; metasoma black-brown; tegula light brown with uneven black patches; legs yellow-brown; radicle, A1–A3 yellow with uneven black patches, remaining antennomeres black; mandibles yellow with teeth dark brown.

#### Head

1.3 × as wide as high, 1.1 × as high as long. Setation on head: sparse. IOS: 0.5 × head width, 0.9 × eye length. POL > LOL > OOL: 17.2:12.1:5.1. OOL: 0.8 × MOD. Compound eye: (L: W = 35.1:28.2). Setation of compound eye: glabrous. Anterior margin of frons: arcuate. Distance from the level of anterior margin of compound eyes to anterior extension of frons: 1.3 × MOD. Number of transverse ledges on upper frons: three. Sculpture of upper frons: anteriorly smooth, followed by a row of effaced polygonal cells and a row of polygonal cells in front of anterior ocellus. Sculpture of lower frons: dorsally smooth followed by a row of polygonal cells, medially smooth and with two vertical rows of polygonal cells and smooth area laterad. Interantennal process: 1.2 × as long as wide, smooth. Transverse carina above interantennal process: without medial notch. Area ventral to transverse carina above interantennal process: smooth, with setigerous foveae laterally. Sculpture on vertex: anteriorly with large polygonal cells, followed by smaller polygonal cells and an uneven transverse carina, posteriorly smooth with setigerous punctae. Sculpture of posterior orbital furrow: dorsally foveate and ventrally rectangular cells. Genal carina: present, not percurrent, curving basally towards orbital carina. Sculpture of gena: anterodorsally smooth, setigerous foveate medially, posteriorly smooth with very sparse setigerous punctae. Sculpture on A1: smooth with sparse setae. A1: 3.8 × as long as wide. Length of A3: 0.3 × A1 and subequal to A2.

#### Mesosoma

Sculpture of dorsal pronotum: smooth with sparse setigerous punctae. L: W of mesoscutum: 34.7:48.4. Sculpture of mesoscutum: smooth with setigerous punctae, punctae sparse posteriorly, setae long. Notaulus: absent. Mesoscutal humeral sulcus: foveate. Mesoscutal suprahumeral sulcus: with oblong cells. Parapsidal line: indicated as furrow. Scutoscutellar sulcus: foveate. L: W of mesoscutellum: 23.2:36.8. Sculpture of mesoscutellum: smooth with sparse setigerous foveae, setae long with a discontinuous furrow anteriorly. Sculpture of dorsellum: anteriorly foveate, sparsely foveate posteromedially, posterior margin upcurved. Sculpture of outer lateral propodeal area: densely foveate. Sculpture of inner lateral propodeal area: anteriorly sparsely setose, posteriorly smooth. Lateral propodeal carina: sinuous. Posterior propodeal projection: rounded, not extending to anterior margin of T1. Sculpture of metasomal depression: smooth with a medial vertical carina and sparse pilosity. Plical area: anteriorly setose, posteriorly smooth with setigerous punctae. Sculpture of propleuron: smooth. Sculpture of lateral pronotal area: smooth. Posterior pronotal sulcus: with oblong cells. Pronotal cervical sulcus: foveate. Speculum of mesopleuron: transversely carinate with sparse setae. Postacetabular sulcus: foveate. Prespecular sulcus: foveate. Mesepimeral sulcus: foveate. Posterior mesepimeral area: smooth, narrower than mesepimeral sulcus. Mesopleural carina: percurrent, with a row of uneven foveae dorsally. Sculpture of femoral depression: smooth. Mesopleural pit: present. Sculpture of ventral mesopleuron: anteriorly and dorsally with polygonal cells, remainder smooth with setigerous punctae. Sculpture of metapleuron: dorsal metapleural area narrow and smooth with long setae on anterior margin; ventral metapleural area dorsally smooth and ventrally with sparse foveae. Metapleural sulcus: foveate, medially indicated as a furrow. Paracoxal sulcus: with oblong cells. Metapleural epicoxal sulcus: with uneven depressions.

#### Fore wing

L: W: 189.2:76.2. Transparency: weakly infuscate. Lengths of R: R1: r-rs in ratio of 77:24:19. R: basally closer and gradually distant from anterior margin of wing. Anterior margin of fore wing: upcurved basally and with no downcurve prior to R1.

#### Metasoma

L: W of metasoma: 101.2:52.1. Ratio of length of T1: T2: T3: T4: T5: 18.3:19.7:18.8:18.8:16.4. Anterior margin of T1: weakly convex Sculpture of T1: basal foveae present, followed medially by longitudinal costae with depressions between them anteriorly and weak foveae posteriorly; anterosublaterally with punctae and depressions, posteriorly smooth, and laterally smooth with dense setae. Sculpture of T2: basal foveae present, followed by short longitudinal costae, extending 0.5 × the length of tergite; laterally smooth with setae, posteriorly smooth. Sculpture of T3: same as T2, costae shorter and present submedially. Sculpture of T4: same as T3. Sculpture of T5: basal foveae present, remainder smooth with sparse setigerous punctae. Sculpture of T6: smooth with setigerous punctae.

#### Male

Similar to female except for the following characters: anterior margin of mesoscutum crenulate; setigerous punctae on mesoscutum and mesoscutellum dense; longitudinal costae on metasomal tergites long.

#### Etymology

This species is named ‘Bhairavi’ after a *raga*, or melodic structure, in Hindustani music, one of the two major traditions of Indian classical music; usually sung at dawn and called the queen of the morning melodies.

### *Sparasion bhupali* Veenakumari sp. n. (Figs. 2A–F, 17C, 18C, 20C–D, 21B)

urn:lsid:zoobank.org:act:FA24D744-CC07-49D5-A266-85EFBACBC27D

#### Diagnosis

*Sparasion bhupali* sp. n. is distinct in having mesoscutum with longitudinal carinae interspersed with setigerous foveae.

#### Material examined

Holotype: Female, (ICAR/NBAIR/P4651), **INDIA**: Tamil Nadu: Hosur, Uddanapalli, 12°37′28ʺN 77°55′29ʺE, 758 m, YPT, 29.XI.2014. Paratypes: 1 female (ICAR/NBAIR/P4652), Tamil Nadu: Hosur, Uddanapalli, 12°37′28ʺN 77°55′29ʺE, 758 m, YPT, 29.XI.2014; 1 male (ICAR/NBAIR/P4653), Tamil Nadu: Hosur, Uddanapalli, 12°37′28ʺN 77°55′29ʺE, 758 m, YPT, 02.XII.2014; 1 male (ICAR/NBAIR/P4654), Tamil Nadu: Hosur, Uddanapalli, 12°37′28ʺN 77°55′29ʺE, 758 m, MT, 11.VI.2015; 1 male (ICAR/NBAIR/P4655) Tamil Nadu: Hosur, Uddanapalli, 12°37′28ʺN 77°55′29ʺE, 758 m, YPT, 29.XI.2014; 1 male (ICAR/NBAIR/P4656), Tamil Nadu: Hosur, Uddanapalli, 12°37′28ʺN 77°55′29ʺE, 758 m, MT, 27.III.2015.

### Description

Female body length = 2.33–2.41 mm (*n* = 2); male body length: 2.28–2.39 mm (*n* = 4).

#### Colour

Head orange-yellow; black patches present on inner margins of ocelli; ledges on upper frons red-brown; mesoscutum and mesoscutellum orange-brown; metanotal trough and lateral propodeal area brown-yellow; lateral margins of mesoscutum and mesoscutal flange black; transverse pronotal carina, lateral propodeal carina brown; metasoma brown-yellow; pronotum and pleuron yellow-brown; tegula black; legs yellow-brown; radicle, A1 red-brown, A2–A3 yellow-black, remaining antennomeres black; mandibles yellow with teeth dark brown.

#### Head

1.2 × as wide as high, 1.1 × as high as long. Setation on head: sparse. IOS: 0.5 × head width, 1.1 × eye length. POL > LOL > OOL: 20.6:14.7:5.0. OOL: 0.8 × MOD. Compound eye: (L: W = 29.7:25.7). Setation of compound eye: glabrous. Anterior margin of frons: sinuous. Distance from level of anterior margin of compound eyes to anterior extension of frons in dorsal view: 1.2 × MOD. Number of transverse ledges on upper frons: two. Sculpture of upper frons: anteriorly with shallow rectangular cells, followed by three polygonal cells on either side of anterior ocellus. Sculpture of lower frons: smooth with polygonal cells bearing setae laterally and a row of long setae dorsally. Interantennal process: 1.3 × as long as wide, smooth with medial furrow. Transverse carina above interantennal process: with a medial notch and an additional transverse carina above. Area ventral to transverse carina above interantennal process: smooth, with shallow cells laterally. Sculpture on vertex: anteriorly with a raised carina posterior to anterior ocellus; with large polygonal cells, followed by a row of smaller polygonal cells and an uneven transverse carina, posteriorly smooth with setigerous punctae and uneven short transverse carinae; anterior ocellus with a narrow smooth area around; lateral ocellus with an irregular smooth area posteriorly. Sculpture of posterior orbital furrow: with rectangular cells. Genal carina: present. Sculpture of gena: anteriorly with weak impressions of setigerous polygonal cells and posteriorly smooth, sparsely setose. Sculpture on A1: smooth with sparse setigerous foveae. A1: 3.8 × as long as wide. Length of A3: 0.3 × A1 and 0.9 × A2.

#### Mesosoma

Sculpture of dorsal pronotum: setigerous depressions. L: W of mesoscutum: 35.0:46.5. Sculpture of mesoscutum: with longitudinal carinae interspersed with sparse setigerous foveae. Notaulus: absent. Mesoscutal humeral sulcus: with shallow depressions. Mesoscutal suprahumeral sulcus: with polygonal cells bearing setae. Parapsidal line: indicated as a furrow. Scutoscutellar sulcus: foveate. L: W of mesoscutellum: 19.0:30.0. Sculpture of mesoscutellum: anteriorly smooth, posteriorly with weak longitudinal carinae interspersed with uneven depressions. Sculpture of dorsellum: anteriorly foveate, posteriorly smooth with posterior margin upcurved medially. Sculpture of outer lateral propodeal area: anteriorly with shallow depressions, sparsely setose. Sculpture of inner lateral propodeal area: anteriorly sparsely setose, with an oblique carina medially, posteriorly smooth. Lateral propodeal carina: arched. Posterior propodeal projection: rounded, not extending to anterior margin of T1. Sculpture of metasomal depression: with weak depressions, sparsely setose. Plical area: anteriorly sparsely setose, posteriorly weakly rugose. Sculpture of propleuron: smooth. Sculpture of lateral pronotal area: smooth. Posterior pronotal sulcus: with irregular cells. Pronotal cervical sulcus: foveate. Speculum of mesopleuron: transversely carinate. Postacetabular sulcus: foveate. Prespecular sulcus: with incomplete foveae. Mesepimeral sulcus: foveate. Posterior mesepimeral area: smooth, narrower than mesepimeral sulcus. Mesopleural carina: percurrent, with shallow depressions on anterior margin. Sculpture of femoral depression: smooth with transverse carinae posteriorly. Mesopleural pit: present. Sculpture of ventral mesopleuron: with transverse rows of depressions, sparsely setose. Sculpture of metapleuron: dorsal metapleural area smooth with long setae on anterior margin; ventral metapleural area dorsally rugose and ventrally with weak impressions of polygonal cells. Metapleural sulcus: foveate, medially indicated as a furrow. Paracoxal sulcus: with shallow foveae. Metapleural epicoxal sulcus: with shallow depressions.

#### Fore wing

L: W: 153.0:70.2. Transparency: weakly infuscate. Lengths of R: R1: r-rs in ratio of 69:27:20. R: curving away from anterior margin of wing. Anterior margin of fore wing: no downcurve prior to R1.

#### Metasoma

L: W of metasoma: 80.9:45.2. Ratio of length of T1: T2: T3: T4: T5: 13.6:16.7:14.2:12.6:13.3. Anterior margin of T1: convex. Sculpture of T1: longitudinally costate except for smooth area laterally with setae, posteriorly smooth. Sculpture of T2: basal foveae present, followed by longitudinal costae; laterally smooth with setigerous punctae, posteriorly smooth. Sculpture of T3: same as T2. Sculpture of T4: same as T2. Sculpture of T5: anteriorly with short longitudinal costae, posterior half smooth with setigerous punctae. Sculpture of T6: rugose.

#### Male

Similar to female except for following characters. Head and mesonotum black-brown and metasoma brown-black; legs yellow-brown; mesoscutum anteromedially with depressions and posteromedially longitudinally costate and laterally smooth; posterior propodeal projections wide and laminar.

#### Etymology

This species is named ‘Bhupali’ after a *raga*, or melodic structure, in the Hindustani tradition of Indian classical music usually performed at dusk.

### *Sparasion bihagi* Veenakumari sp. n. (Figs. 3A–F, 17D, 18D, 20E–F, 21C)

urn:lsid:zoobank.org:act:74F22B5B-1319-4878-B7EA-0EF799D0C41C

#### Diagnosis

*Sparasion bihagi* sp. n. is distinct in the *S. bilahari* species group in having the first frontal ledge placed lower at 2/3 level along ventral margin of eye and in having weak reticulations on medial frons.

#### Material examined

Holotype: Female, (ICAR/NBAIR/P4737), INDIA: Karnataka: Chikkaballapur, Nandi Hills, 13°37′02ʺN 77°41′34ʺE, 1448 m, YPT, 01.V.2015. Paratypes: 1 female (ICAR/NBAIR/P4738), Karnataka: Chikkaballapur, Nandi Hills, 13°37′02ʺN 77°41′34ʺE, 1448 m, SN, 08.IX.2010; 1 male (ICAR/NBAIR/P4739), Karnataka: Chikkaballapur, Nandi Hills, 13°37′02ʺN 77°41′34ʺE, 1448 m, SN, 26.VIII.2010; 1 male (ICAR/NBAIR/P4740), Karnataka: Bengaluru, Hebbal, 13°02′08ʺN 77°35′49ʺE, 906 m, SN, 29.VII.2010; 1 male (ICAR/NBAIR/P4741), Karnataka: Tumkuru: Kunigal, Ranganathaswamy Betta, 13°02′02ʺN, 76°58′18ʺ E, 901 m, SN, 20.XI. 2011; 1 male (ICAR/NBAIR/P4742) Karnataka: Chikkaballapur, Nandi Hills, 13°37′02ʺN 77°41′34ʺE, 1448 m, SN, 21.X.2010.

### Description

Female body length = 2.32–2.41 mm (*n* = 2); male body length = 2.15–2.31 mm (*n* = 4).

#### Colour

Frons yellow with red-brown ledges; black patches surrounding posterior ocelli; vertex, mesoscutum, mesoscutellum, dorsellum honey brown; dorsal pronotum, lateral propodeal area yellow–brown; pleuron yellow; metasoma black-brown medially, laterally yellow-brown and posterior margins of each tergite brown; legs yellow; tegula black with sparse brown patches; radicle, basal half of A1 yellow, remainder of A1 and A2–A3 yellow-brown, A4 brown, remaining antennomeres black; mandibles yellow-brown with teeth dark brown.

#### Head

1.1 × as wide as high, 1.2 × as high as long. Setation on head: sparse. IOS: 0.5 × head width, 1.1 × eye length. POL > LOL > OOL: 18.0:12.5:5.0. OOL: 0.7 × MOD. Compound eye: (L: W = 30.3:24.9). Setation of compound eye: sparsely setose. Anterior margin of frons: arcuate. Distance from level of anterior margin of compound eyes to anterior extension of frons in dorsal view: 1.2 × MOD. Number of transverse ledges on upper frons: three, first ledge placed 2/3 level from ventral eye margin. Sculpture of upper frons: smooth with sparse setae. Sculpture of lower frons: medially weakly reticulate with a smooth patch followed by effaced polygonal cells bearing setae on either side. Interantennal process: 1.2 × as long as wide, smooth. Transverse carina above interantennal process: without a medial notch. Area ventral to transverse carina above interantennal process: smooth, with weak longitudinal striae laterad. Sculpture on vertex: with three shallow polygonal cells on either side of anterior ocellus, followed by smooth area and a transverse carina between posterior ocelli, posteriorly smooth with sparse setigerous punctae; Sculpture of posterior orbital furrow: foveate. Genal carina: present: Sculpture of gena: anteriorly with shallow rectangular cells bearing setae, posteriorly smooth. Sculpture on A1: smooth with sparse setae. A1: 4.1 × as long as wide. Length of A3: 0.3 × A1 and subequal to A2.

#### Mesosoma

Sculpture of dorsal pronotum: with setigerous foveae. L: W of mesoscutum: 36.6:46.2. Sculpture of mesoscutum: smooth with sparse setigerous foveae. Notaulus: absent. Mesoscutal humeral sulcus: foveate. Mesoscutal suprahumeral sulcus: with circular cells. Parapsidal line: indicated as furrow. Scutoscutellar sulcus: foveate. L: W of mesoscutellum: 20.6:34.4. Sculpture of mesoscutellum: anteriorly smooth with an incomplete carina, posteriorly with setigerous foveae. Sculpture of dorsellum: anteriorly foveate, posteriorly smooth, posterior margin weakly upcurved. Sculpture of outer lateral propodeal area: with shallow depressions, sparsely setose. Sculpture of inner lateral propodeal area: anteriorly sparsely setose, posteriorly smooth. Lateral propodeal carina: anteriorly arched and posteriorly oblique. Posterior propodeal projection: rounded, not extending to anterior margin of T1. Sculpture of metasomal depression: smooth with a medial vertical carina and sparse pilosity. Plical area: anteriorly setose, posteriorly with shallow depressions with sparse setae. Sculpture of propleuron: smooth. Sculpture of lateral pronotal area: smooth. Posterior pronotal sulcus: dorsally with irregular cells and ventrally foveate. Pronotal cervical sulcus: not foveate. Speculum of mesopleuron: transversely carinate, sparsely setose. Postacetabular sulcus: indicated as a shallow furrow. Prespecular sulcus: foveate, foveae wide progressively decreasing in width ventrad. Mesepimeral sulcus: foveate. Posterior mesepimeral area: smooth, narrower than mesepimeral sulcus. Mesopleural carina: percurrent, with a row of irregular foveae dorsally. Sculpture of femoral depression: smooth. Mesopleural pit: present. Sculpture of ventral mesopleuron: with a row of irregular foveae anterodorsally, ventrally smooth with sparse setigerous punctae. Sculpture of metapleuron: dorsal metapleural area smooth with sparse long setae on anterior margin; ventral metapleural area dorsally smooth and ventrally with shallow polygonal cells. Metapleural sulcus: with shallow foveae, indicated medially as furrow. Paracoxal sulcus: foveate. Metapleural epicoxal sulcus: with shallow polygonal cells.

#### Fore wing

L: W: 165.7:72.9. Transparency: weakly infuscate. Lengths of R: R1: r-rs in ratio of 68:38:21. R: parallel to anterior margin of wing. Anterior margin of fore wing: with a downcurve prior to R1.

#### Metasoma

L: W of metasoma: 82.7:47.7. Ratio of length of T1: T2: T3: T4: T5: 15.9:16.8:15.0:14.1:12.3. Anterior margin of T1: convex. Sculpture of T1: longitudinally costate, laterally smooth with sparse setae, posteriorly smooth. Sculpture of T2: basal foveae present, followed by longitudinal costae; laterally smooth with setae, posteriorly smooth. Sculpture of T3: same as T2, anteromedially with shorter costae. Sculpture of T4: same as T3, anteromedially with shorter costae. Sculpture of T5: anteriorly with setigerous punctae and short longitudinal carinae, remainder weakly coriaceous reticulate with setigerous punctae except for smooth posterior margin; Sculpture of T6: smooth with setae.

#### Male

Similar to female except for the following characters: mesoscutum, mesoscutellum and metasoma dark brown; T4 with basal foveae, T5 with elongate depressions basally.

#### Etymology

This species is named ‘Bihag’ after a *raga*, or melodic structure, in classical Hindustani music, one of the two schools of Indian classical music, to be performed late at night.

### *Sparasion bilahari* Veenakumari sp. n. (Figs. 4A–F, 17E, 18E, 20G–H, 21D)

urn:lsid:zoobank.org:act:3C3BB511-DD5A-4453-B96A-FA2B0B71E8ED

#### Diagnosis

*Sparasion bilahari* sp. n. is close to *S. darbari* sp. n. but differs from it in the following characters: in *S. bilahari* sp. n. mesoscutum is smooth with sparse punctae, mesoscutellum also smooth with sparse foveae posteriorly, and posterior propodeal projection is rounded. Conversely, in *S. darbari* sp. n. mesoscutum is weakly rugose with sparse punctae, mesoscutellum is with longitudinal carinae posteriorly and posterior propodeal projection is angular.

#### Material examined

Holotype: Female, (ICAR/NBAIR/P4657), **INDIA**: Tamil Nadu: Hosur, Uddanapalli, 12°37′28ʺN 77°55′29ʺE, 758 m, YPT, 03.II.2016. Paratypes: 1 female (ICAR/NBAIR/P4658), Karnataka: Bengaluru, Attur, 13°05′48ʺN 77°33′59ʺE, 936 m, MT, 12.VIII.2012; 1 female (ICAR/NBAIR/P4659), Karnataka: Bengaluru, Attur, 13°05′48ʺN 77°33′59ʺE, 936 m, YPT, 26.XII.2013; 1 female (ICAR/NBAIR/P4660), Karnataka: Bengaluru, Attur, 13°05′48ʺN 77°33′59ʺE, 936 m, YPT, 20.XII.2013; 2 females (ICAR/NBAIR/P4661–P4662), Karnataka: Bengaluru, Jarakabande Kaval, 13°05′41ʺN 77°32′35ʺE, 921 m, MT, 18.VIII.2015; 1 female (ICAR/NBAIR/P4663), Karnataka: Bengaluru, Jarakabande Kaval, 13°05′41ʺN 77°32′35ʺE, 921 m, MT, 03.XII.2013; 2 females (ICAR/NBAIR/P4664–P4665), Karnataka: Bengaluru, Jarakabande Kaval, 13°05′41ʺN 77°32′35ʺE, 921 m, MT, 09.IX.2014; 1 female (ICAR/NBAIR/P4666), Karnataka: Bengaluru, Jarakabande Kaval, 13°05′41ʺN 77°32′35ʺE, 921 m, MT, 29.II.2013; 1 female (ICAR/NBAIR/P4667), Karnataka: Bengaluru, Hebbal, National Bureau of Agricultural Insect Resources (NBAIR), 13°01′38ʺN 77°35′03ʺE, 927 m, YPT, 18.VIII.2015; 1 male (ICAR/NBAIR/P4668) Karnataka: Bengaluru, Attur, 13°05′48ʺN 77°33′59ʺE, 936 m, MT, 05.VIII.2013; 1 male (ICAR/NBAIR/P4669), Karnataka: Bengaluru, Attur, 13°05′48ʺN 77°33′59ʺE, 936 m, MT, 12.VIII.2013; 1 male (ICAR/NBAIR/P4670), Karnataka: Bengaluru, Attur, 13°05′48ʺN 77°33′59ʺE, 936 m, SN, 25.IX.2010; 1 male (ICAR/NBAIR/P4671), Tamil Nadu: Hosur, Uddanapalli, 12°37′28ʺN 77°55′29ʺE, 758 m, YPT, 03.II.2016; 1 male (ICAR/NBAIR/P4672), Karnataka: Bengaluru, Jarakabande Kaval, 13°05′41ʺN 77°32′35ʺE, 921 m, MT, 14.XI.2014.

### Description

Female body length = 2.31–2.48 mm (*n* = 10); male body length: 2.12–2.23 mm (*n* = 5).

#### Colour

Head black; mesoscutum medially brown and laterally brown-black; mesoscutellum, dorsellum, metanotal trough yellow-brown; pronotum, pleuron, lateral propodeal area yellow-brown; transverse pronotal carina black; T1–T3 brown-black and remaining tergites black; tegula dark brown with uneven light brown patches; legs yellow-brown; radicle, A1–A3 yellow-brown, A4 brown, remaining antennomeres black; mandibles yellow with teeth dark brown.

#### Head

1.2 × as wide as high, 1.1 × as high as long. Setation on head: sparse. IOS: 0.5 × head width, 1.1 × eye length. POL > LOL > OOL: 19.5:11.9:5.7. OOL: 0.8 × MOD. Compound eye: (L: W = 37.5:31.3). Setation of compound eye: sparsely setose. Anterior margin of frons: arcuate. Distance from level of anterior margin of compound eyes to anterior extension of frons in dorsal view: 1.3 × MOD. Number of transverse ledges on upper frons: three. Sculpture of upper frons: anteriorly smooth, followed by a row of effaced polygonal cells and two polygonal cells on either side of anterior ocellus. Sculpture of lower frons: smooth with 1 row dorsally and two rows of polygonal cells laterally bearing setae. Interantennal process: 1.5 × as long as wide, smooth with medial indentation. Transverse carina above interantennal process: with a medial notch. Area ventral to transverse carina above interantennal process: smooth, with setigerous foveae dorsally and laterally. Sculpture on vertex: anteriorly with large polygonal cells, followed by a row of smaller polygonal cells and a sinuous transverse carina, posteriorly smooth with setigerous punctae; anterior ocellus with no smooth area around; posterior ocellus with an elongate triangular smooth area posteriorly. Sculpture of posterior orbital furrow: dorsally foveate and ventrally with rectangular cells. Genal carina: present. Sculpture of gena: anteriorly with foveae and posteriorly smooth, setae sparse. Sculpture on A1: smooth with sparse setae. A1: 3.7 × as long as wide. Length of A3: 0.2 × A1 and 0.7 × A2.

#### Mesosoma

Sculpture of dorsal pronotum: setigerous depressions. L: W of mesoscutum: 37.3:50.4. Sculpture of mesoscutum: smooth with sparse setigerous punctae. Notaulus: absent. Mesoscutal humeral sulcus: foveate. Mesoscutal suprahumeral sulcus: with circular cells. Parapsidal line: indicated as furrow. Scutoscutellar sulcus: foveate. L: W of mesoscutellum: 23.0:36.7. Sculpture of mesoscutellum: smooth with sparse setigerous foveae and a discontinuous furrow anteriorly. Sculpture of dorsellum: anteriorly foveate, posteriorly smooth with a medial vertical carina and posterior margin straight. Sculpture of outer lateral propodeal area: smooth, sparsely setose. Sculpture of inner lateral propodeal area: with an oblique medial carina, anteriorly sparsely setose, posteriorly smooth. Lateral propodeal carina: oblique. Posterior propodeal projection: rounded, not extending to anterior margin of T1. Sculpture of metasomal depression: smooth with a medial longitudinal carina. Plical area: anteriorly setose, posteriorly smooth, with sparse setae. Sculpture of propleuron: smooth. Sculpture of lateral pronotal area: reticulate. Posterior pronotal sulcus: foveate. Pronotal cervical sulcus: not foveate. Speculum of mesopleuron: transversely carinate, sparsely setose. Postacetabular sulcus: foveate. Prespecular sulcus: with wide shallow depressions. Mesepimeral sulcus: foveate, progressively increasing in width ventrad. Posterior mesepimeral area: smooth, narrower mesepimeral sulcus. Mesopleural carina: percurrent, with a row of shallow depressions dorsally. Sculpture of femoral depression: smooth. Mesopleural pit: present. Sculpture of ventral mesopleuron: with transverse rows of depressions, sparsely setose. Sculpture of metapleuron: dorsal metapleural area smooth with long setae on anterior margin; ventral metapleural area dorsally smooth and ventrally with sparse setigerous polygonal cells in addition to transverse carinae on anterior margin. Metapleural sulcus: foveate, medially indicated as a furrow. Paracoxal sulcus: foveate, interspersed with transverse carinae ventrally. Metapleural epicoxal sulcus: foveate.

#### Fore wing

L: W: 180.8:75.2. Transparency: weakly infuscate. Lengths of R: R1: r-rs in ratio of 78:42:24. R: basally closer, gradually distant from anterior margin of wing. Anterior margin of fore wing: with no downcurve prior to R1.

#### Metasoma

L: W of metasoma: 88.6:53.3. Ratio of length of T1: T2: T3: T4: T5: 16.3:20.7:18.1:18.1:15.0. Anterior margin of T1: convex. Sculpture of T1: medially longitudinally costate, laterally with short costae anteriorly and posteriorly smooth with a row of setigerous punctae. Sculpture of T2: basal foveae present, followed by short longitudinal costae; laterally smooth with dense setae, posteriorly smooth. Sculpture of T3: same as T2. Sculpture of T4: same as T2. Sculpture of T5: anteriorly with short longitudinal depressions interspersed with setigerous punctae, posteriorly smooth. Sculpture of T6: smooth.

#### Male

Similar to female except for following characters. Dorsal pronotum dark brown, mesoscutum and mesoscutellum black-brown, metanotum and propodeum brown-black, metasoma black. Setigerous punctae on mesoscutum dense, mesoscutellum anteriorly smooth, posteriorly foveate, costae on tergites long extending 0.8 × length of tergite, propodeal carinae more pronounced.

#### Etymology

This species is named ‘Bilahari’ after the *ragam*, or melodic structure, rendered in the morning in Carnatic music, the South Indian classical music tradition.

### *Sparasion cellularis* Strand (Figs. 17F, 18F: Taeger [22], 19, 22A–I, 23A–G)

**Fig. 22:**
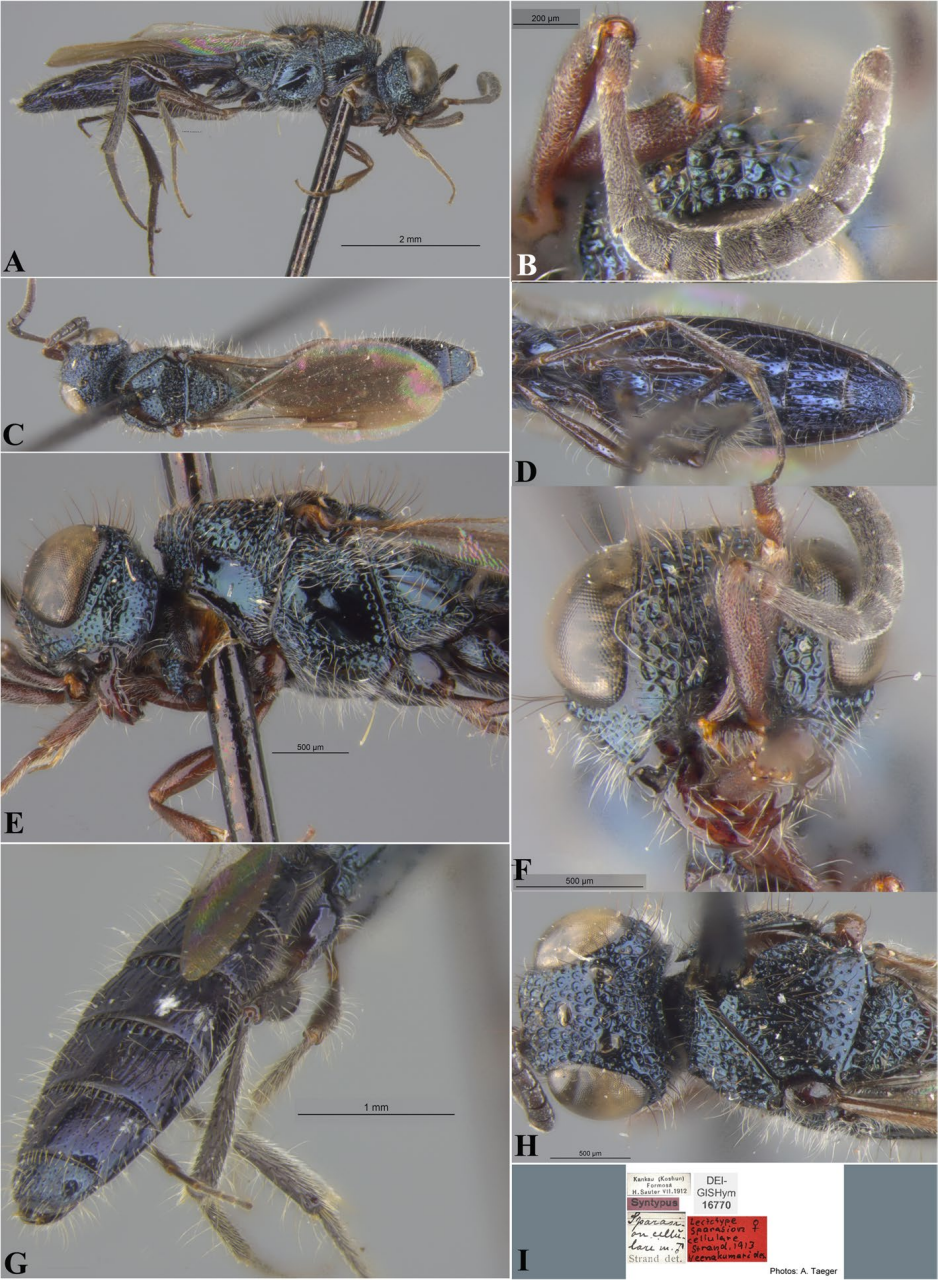
*Sparasion cellularis* Strand, female lectotype, **A** Habitus, lateral view. **B** Antenna. **C** Habitus, dorsal view. **D** Sternites. **E** Head and pleuron. **F** Frons. **G** Metasoma, dorsolateral view. **H** Head and mesonotum. **I** Type labels. (Photos: Dr. Andreas Taeger © SDEI)

**Fig. 23:**
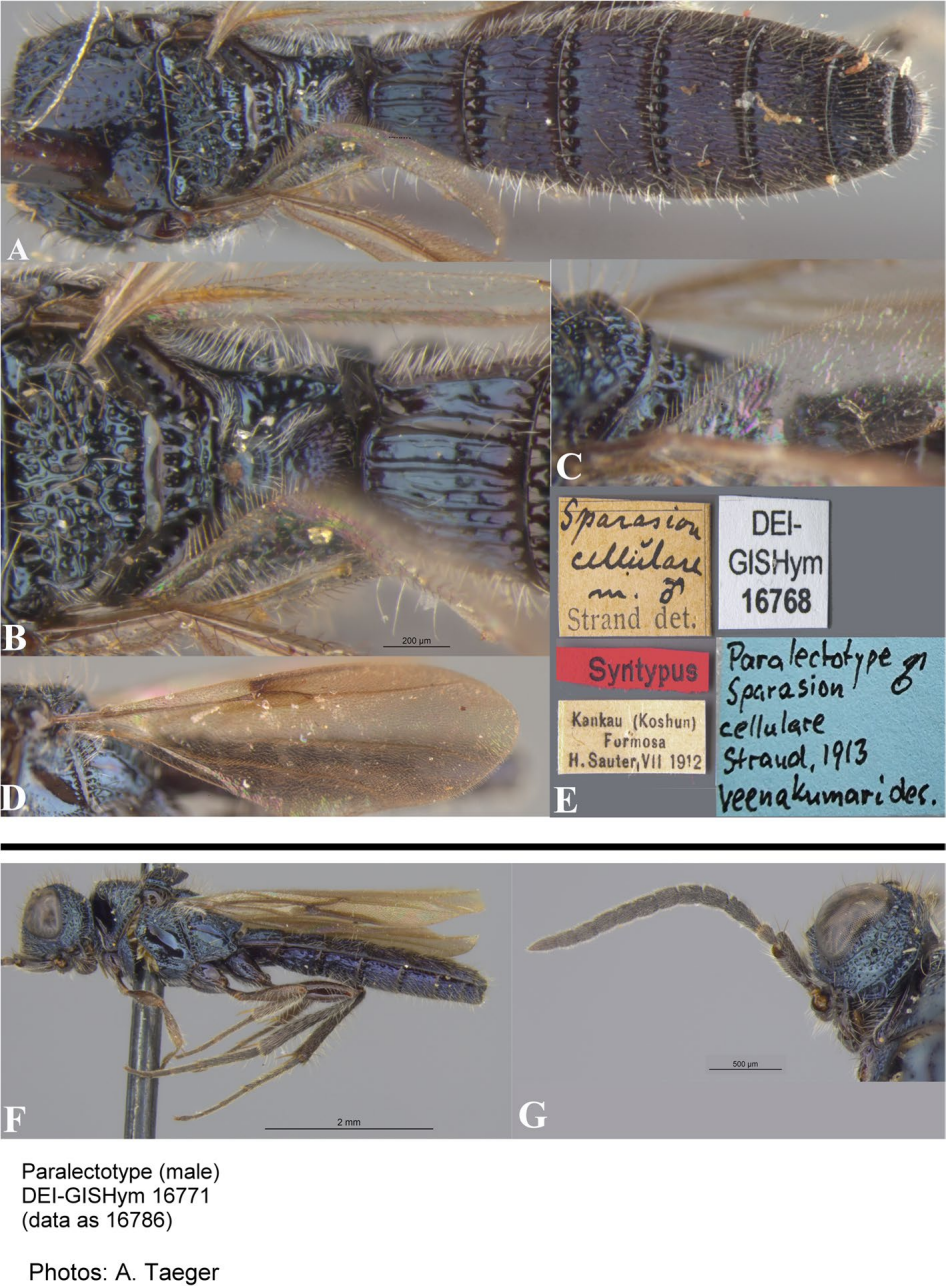
*Sparasion cellularis* Strand, male paralectotype, DEI-GISHym 16,768, **A**–**E**: **A** Mesonotum and metasoma. **B** Mesoscutellum, metasomal depression and T1. **C** Mesoscutellum, lateral view. **D** Fore wing. **E** Type labels. Male paralectotype, DEI-GISHym 16,786. **F**–**G**: **F** Habitus lateral view. **G** Head and antenna. (Photos: Dr. Andreas Taeger © SDEI)

*Sparasion cellulare* Strand, 1913: 211

*Sparasion cellularis*: Kieffer, 1926: 284, 295. Description, emendation, keyed.

*Sparasion cellulare*: Oehlke and Szabó, 1984: 129. Type information.

#### Diagnosis

*Sparasion cellularis* is close to *S. cullaris*, *S. meghmalhari* sp. n. and *S. travancoricus* but differs from them in following combination of character states: in *S. cellularis* speculum of mesopleuron is setigerous foveate and notaulus is absent; conversely, in the latter three species speculum of mesopleuron is setigerous foveate interspersed with transverse carinae; notaulus is present.

#### Material examined

Female, lectotype (by present designation) (DEI-GISHym 16670), **TAIWAN**: Kankau (Koshun) (present name: Hengchun), VII.1912, leg. H. Sauter. Paralectotype (by present designation) (DEI-GISHym 16768), 1 male, Kankau (Koshun) (present name: Hengchun), VII.1912, leg. H. Sauter; paralectotype (by present designation) (DEI-GISHym 16771), 1 male, Kankau (Koshun), VII.1912, leg. H. Sauter.

### Description

Female body length = 6 mm.

#### Colour

Head, mesosoma and metasoma steel blue; tegula brown; fore coxa brown, mid- and hind- coxae steel blue, remainder of legs brown-black; radicle, A1–A2 red-brown, remaining antennomeres brown-black; mandibles red-brown.

#### Head

Setation on head: dense. Setation of compound eye: glabrous. Anterior margin of frons: arcuate with weak indentation medially. Distance from the level of anterior margin of compound eyes to anterior extension of frons in dorsal view: 0.5 × MOD. Number of transverse ledges on upper frons: one. Sculpture of upper frons: with circular cells bearing setae. Sculpture of lower frons: with closely packed polygonal cells bearing setae. Transverse carina above interantennal process: with medial notch. Area ventral to transverse carina above interantennal process: with setigerous punctae. Sculpture on vertex: anteriorly with circular cells and posteriorly with closely packed polygonal cells bearing setae followed by an uneven transverse carina and posteriorly smooth with setigerous punctae; a smooth area present around anterior ocellus; a smooth triangular area present posterior to lateral ocellus. Sculpture of posterior orbital furrow: with depressions. Genal carina: absent. Sculpture of gena: smooth with setigerous foveae and punctae, sparsely setose. Sculpture on A1: densely setigerous punctate.

#### Mesosoma

Sculpture of dorsal pronotum: with setigerous foveae. Sculpture of mesoscutum: smooth with setigerous foveae with punctae. Notaulus: absent. Mesoscutal humeral sulcus: with depressions. Mesoscutal suprahumeral sulcus: with depressions. Parapsidal line: present as a furrow. Scutoscutellar sulcus: foveate. Sculpture of mesoscutellum: with compact polygonal cells, and an incomplete transverse furrow anteriorly. Sculpture of dorsellum: anteriorly foveate, posteriorly smooth with weak longitudinal furrows. Lateral propodeal carina: straight. Posterior propodeal projection: rounded. Sculpture of metasomal depression: sparsely setose. Plical area: anteriorly sparsely setose, posteriorly with large shallow depressions. Sculpture of propleuron: with a shallow furrow. Sculpture of lateral pronotal area: smooth with setigerous punctae posterad. Posterior pronotal sulcus: foveate. Pronotal cervical sulcus: posteriorly with sparse foveae. Speculum of mesopleuron: foveate-punctate, densely setose. Postacetabular sulcus: hidden by dense setae. Prespecular sulcus: foveate. Mesepimeral sulcus: foveate. Mesepimeral area: smooth, narrower than mesepimeral sulcus. Mesopleural carina: indicated anteriorly with foveae on dorsal margin. Sculpture of femoral depression: smooth. Mesopleural pit: present. Sculpture of ventral mesopleuron: smooth with foveae, setae dense. Sculpture of metapleuron: dorsal metapleural area smooth with dense long setae on anterior margin; ventral metapleural area dorsally smooth and ventrally with polygonal cells bearing brown and white setae. Metapleural sulcus: foveate, medially indicated as a furrow. Paracoxal sulcus: with depressions. Metapleural epicoxal sulcus: densely setose.

#### Fore wing

L: W: 324:117. Transparency: strongly infuscate. Lengths of R: R1: r-rs in ratio of 46:24:12. R: distant from anterior margin of wing. Anterior margin of wing: no downcurve prior to R1.

#### Metasoma

L: W of metasoma: 260:90. Sculpture of T1: longitudinally costate, laterally smooth with setigerous punctae and posteriorly smooth. Sculpture of T2–T4: basal foveae present, longitudinally costate, laterally smooth with setigerous punctae and posteriorly smooth; Sculpture of T5: entirely smooth with setigerous punctae except for short longitudinal costae sublaterally on anterior margin. Sculpture of T6: smooth with setigerous punctae.

#### Male

Similar to female.

#### Remarks

DEI-GISHym 16670 is a female, incorrectly mentioned as a male by Strand (1913). DEI-GISHym 16768 is without head. Lectotype and paralectotypes are preserved in good condition in SDEI.

### *Sparasion coconcu*s Kozlov & Lê (Figs. 17G, 18G, 19, 24A–G)

**Fig. 24:**
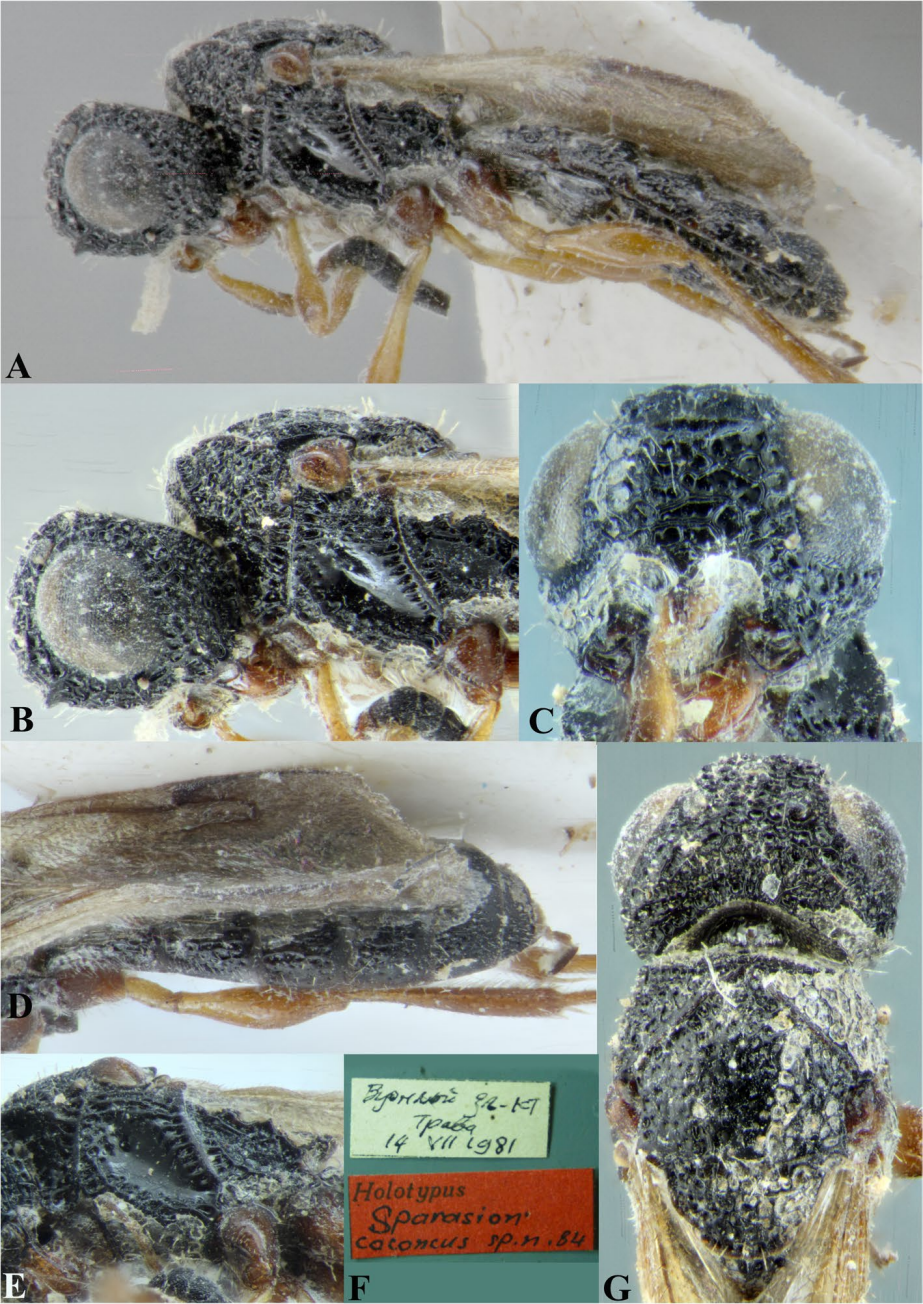
*Sparasion coconcu*s Kozlov and Lê, female holotype. **A** Habitus, lateral view. **B** Head and pleuron. **C** Frons. **D** Wings and metasoma. **E** Pleuron, lateroventral view. **F** Type labels **G** Head and mesonotum (Photos: Drs. N. F. Johnson © Ohio State University and E. J. Talamas, Florida Department of Agriculture and Consumer Services)

*Sparasion coconcus* Kozlov & Lê, 2000: 203, 354. Original description, keyed.

#### Diagnosis

*Sparasion coconcus* is close to *S. deepaki* sp. n. but differs from it in the following combination of character states: in *S. coconcus* frons is with polygonal cells and with two short transverse carinae medially; posterior pronotal sulcus indicated with narrow transverse cells and T1 without spine anteromedially. Conversely, in *S. deepaki* sp. n. frons with several transverse carinae with polygonal cells between them; posterior pronotal sulcus indicated as large ovoid cells and T1 with a short spine anteromedially.

#### Material examined

Holotype, female, **VIETNAM**: Gia Lai: An Khe*:* Buon Luoi*,* 14.XII.1978, leg. Lê, X. H.

### Description

Female body length = 3.5 mm.

#### Colour

Head, mesosoma and metasoma black; tegula brown; all coxae brown, remainder of legs yellow-brown; radicle, A1 yellow-brown, A2–A3 brown, remaining antennomeres black-brown; mandibles brown.

#### Head

Setation on head: sparse. Setation of compound eye: glabrous. Anterior margin of frons: arcuate with a weak medial indentation. Distance from the level of anterior margin of compound eyes to anterior extension of frons in dorsal view: 1.2 × MOD. Number of transverse ledges on upper frons: three. Sculpture of upper frons: with polygonal cells bearing setae. Sculpture of lower frons: dorsally smooth, remainder with polygonal cells bearing setae and two short ribbed costae posteromedially. Sculpture on vertex: anteriorly with polygonal cells, followed by small setigerous foveae; irregular smooth area present on posterior margin of lateral ocellus. Sculpture of posterior orbital furrow: with polygonal cells. Genal carina: present. Sculpture of gena: anteriorly with polygonal cells, posteriorly smooth. Sculpture on A1: smooth with sparse long setae.

#### Mesosoma

Sculpture of dorsal pronotum: with setigerous depressions. Sculpture of mesoscutum: smooth with foveae. Notaulus: present. Sculpture of notaulus: foveate. Mesoscutal humeral sulcus: foveate. Mesoscutal suprahumeral sulcus: foveate. Scutoscutellar sulcus: foveate. Sculpture of mesoscutellum: with closely packed foveae. Sculpture of lateral pronotal area: smooth. Posterior pronotal sulcus: indicated as narrow transverse foveae. Pronotal cervical sulcus: foveate posteriorly. Speculum of mesopleuron: transversely carinate. Prespecular sulcus: with wide foveae. Mesepimeral sulcus: foveate. Mesepimeral area: smooth, narrower than mesepimeral sulcus. Mesopleural carina: present with a row of shallow depressions anteriorly. Sculpture of femoral depression: smooth. Mesopleural pit: present. Sculpture of metapleuron: dorsal metapleural area not distinct; ventral metapleural area dorsally smooth and ventrally with sparse foveae. Metapleural sulcus: foveate, indicated medially as a furrow. Paracoxal sulcus: with wide foveae.

#### Fore wing

Transparency: weakly infuscate. Length of R1: 1.6 × as long as r-rs. R: gradually curving away from anterior margin of wing towards bulla.

#### Metasoma

L: W of metasoma: 2.5 × as long as wide. Sculpture of T1–T5: longitudinally costate, laterally smooth with sparse depressions and punctae, posteriorly smooth. Sculpture of T6: smooth with sparse punctae.

#### Male

Briefly described by Kozlov and Lê [19]. Not examined in this study.

#### Remarks

The holotype is present in IEBR, Vietnam.

### *Sparasion coeruleus* Kieffer (Figs. 17H, 18H, 19, 25A–G, 26A–F)

**Fig. 25:**
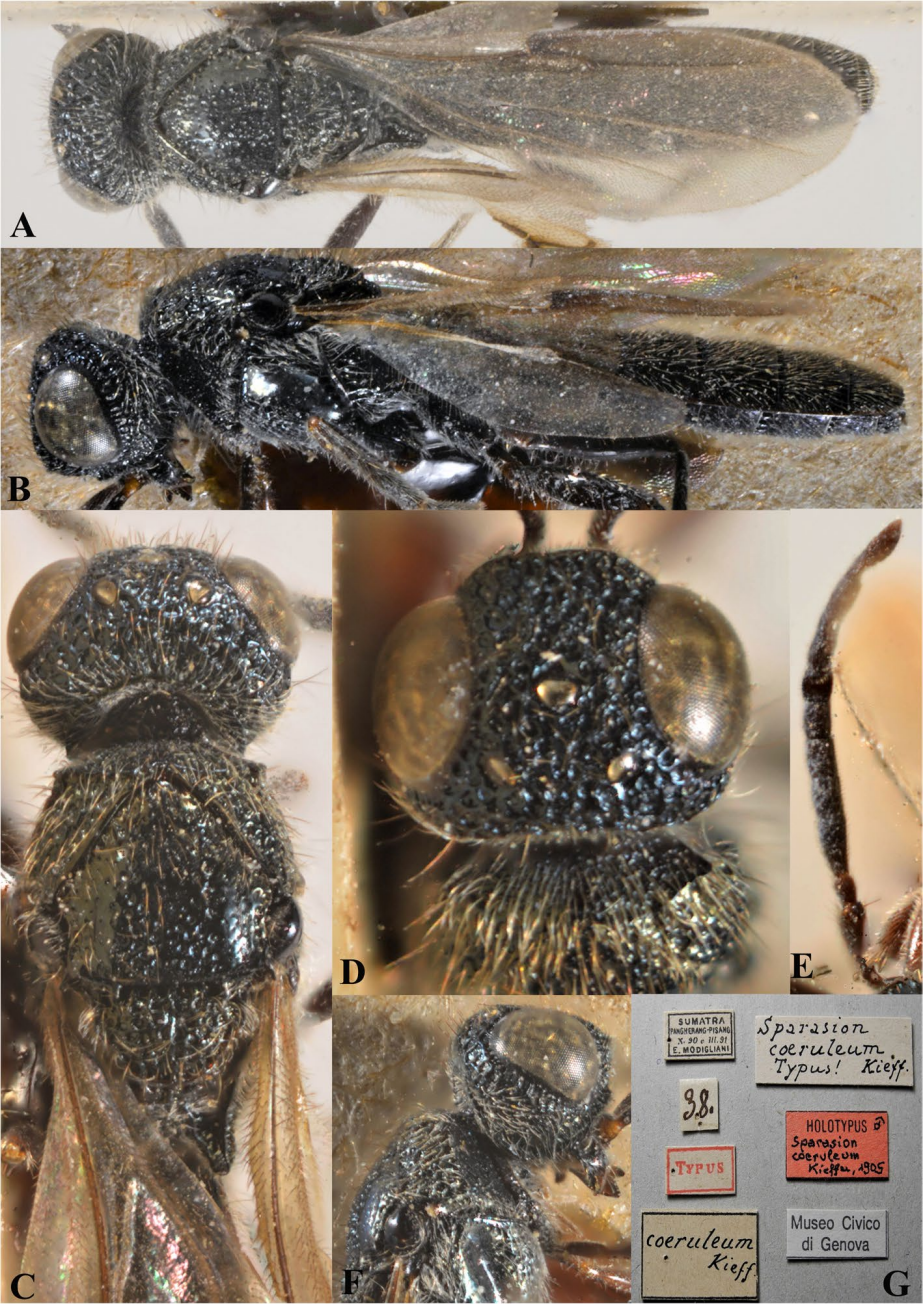
*Sparasion coeruleus* Kieffer, male holotype. **A** Habitus, dorsal view. **B** Habitus, lateral view. **C** Head and mesonotum. **D** Anterior frons, vertex and pronotum. **E** Antenna. **F** Head and pronotal area, lateral view. **G** Type labels (Photos: Dr. Roberto Poggi © MCSN)

**Fig. 26:**
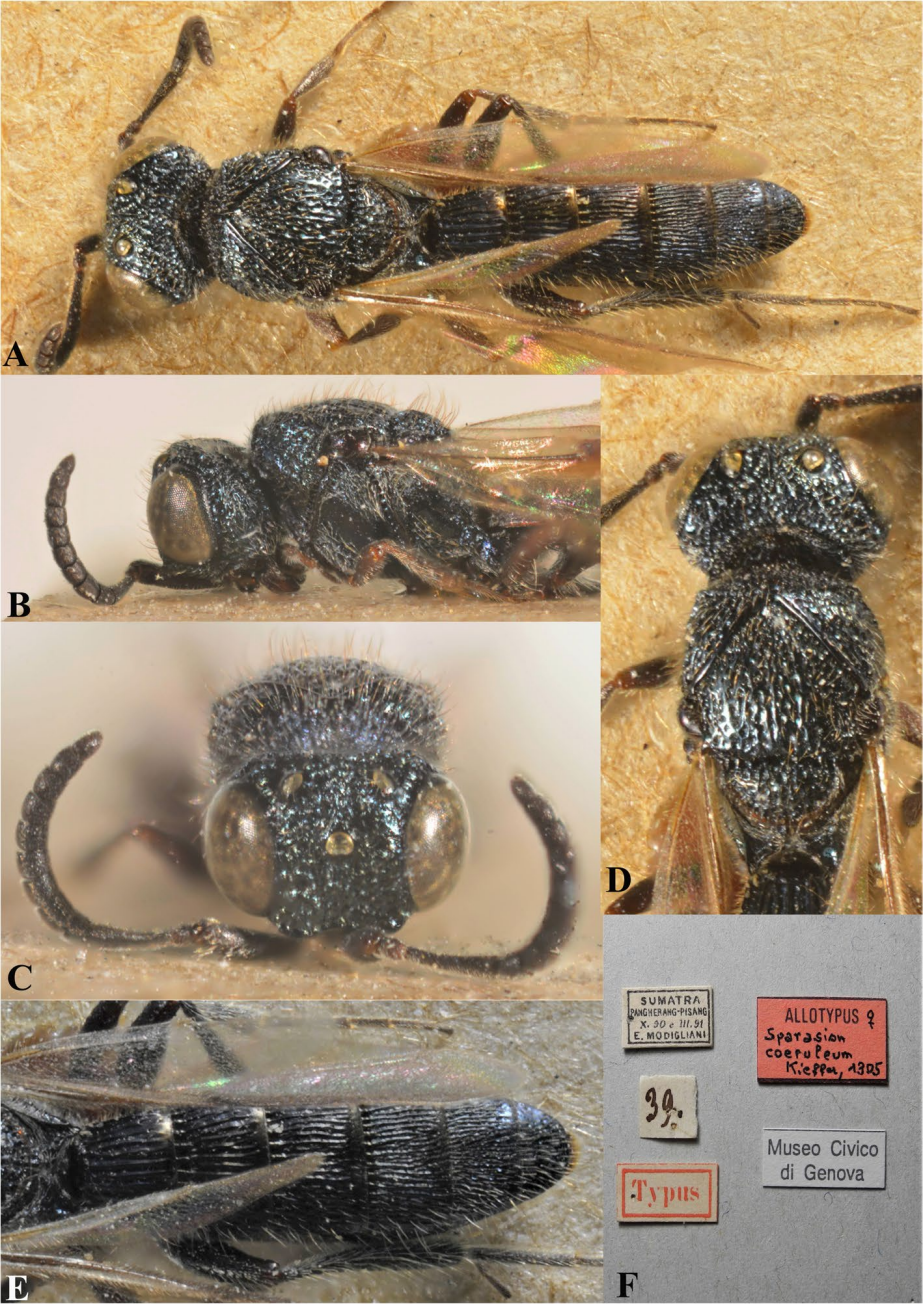
*Sparasion coeruleus* Kieffer, female paratype. **A** Habitus, dorsal view. **B** Head and pleuron. **C** Frons and antennae. **D** Head and mesonotum. **E** Metasoma. **F** Type labels (Photos: Dr. Roberto Poggi © MCSN)

*Sparasion coeruleum* Kieffer, 1905: 20.

*Sparasion coeruleus*: Kieffer, 1926: 284, 295. Description, emendation, keyed.

*Sparasion coeruleus*: Bin, 1974: 459. Type information

#### Diagnosis

*Sparasion coeruleus* is distinct in having longitudinal carinae along with foveae on mesoscutum.

#### Material examined

Holotype, male, (38), **SUMATRA**: Pangherang-Pisang, X.1890 to III.1891, leg. E. Modigliani. Paratype (Allotype): 1 female, Pangherang-Pisang, X.1890 to III.1891, leg. E. Modigliani.

### Description

Male body length = 5.5 mm.

#### Colour

Head and mesosoma and metasoma steel blue-black; tegula brown-black; all legs brown-black; radicle and basal A1 orange-brown, remaining antennomeres brown-black; mandibles brown-black.

#### Head

Setation on head: dense. Setation of compound eye: glabrous. Anterior margin of frons: arcuate. Distance from the level of anterior margin of compound eyes to anterior extension of frons in dorsal view: subequal to MOD. Number of transverse ledges on upper frons: one. Sculpture of upper frons: with a network of cells bearing setae. Sculpture of lower frons: with closely packed circular cells bearing setae. Sculpture on vertex: with closely packed circular cells bearing setae between lateral ocelli; oblique carinae present on temples; a smooth area present anterior to anterior ocellus; a small smooth area present posterior to lateral ocellus. Sculpture of posterior orbital furrow: with depressions. Genal carina: absent. Sculpture of gena: smooth with dense setigerous punctae. Sculpture on A1: densely setigerous punctate.

#### Mesosoma

Sculpture of dorsal pronotum: with setigerous depressions and foveae and laterally extending as blunt spines. Sculpture of mesoscutum: smooth with sparse setigerous foveae with punctae. Notaulus: present. Sculpture of notaulus: foveate. Mesoscutal humeral sulcus: with depressions. Mesoscutal suprahumeral sulcus: with depressions. Parapsidal line: present as a furrow. Scutoscutellar sulcus: foveate. Sculpture of mesoscutellum: with closely packed polygonal cells. Sculpture of dorsellum: anteriorly foveate, posteriorly smooth. Sculpture of outer lateral propodeal area: anteriorly densely setose concealing the sculpture and posteriorly foveate. Sculpture of inner lateral propodeal area: anteriorly smooth, medial transverse carina present and posteriorly smooth. Lateral propodeal carina: sinuous. Posterior propodeal projection: rounded, extending onto anterior margin of T1. Sculpture of metasomal depression: densely setose. Sculpture of lateral pronotal area: smooth. Posterior pronotal sulcus: with large depressions. Speculum of mesopleuron: densely setose. Prespecular sulcus: foveate. Mesepimeral sulcus: foveate. Mesepimeral area: smooth, narrower than mesepimeral sulcus. Sculpture of femoral depression: smooth. Sculpture of ventral mesopleuron: densely setose. Sculpture of metapleuron: dorsal metapleural smooth with long setae on anterior margin; ventral metapleural area dorsally smooth and ventrally with sparse foveae and setae. Metapleural sulcus: foveate, indicated medially as a furrow. Paracoxal sulcus: with wide foveae.

#### Fore wing

L: W: 336:110. Transparency: strongly infuscate. Lengths of R: R1: r-rs in ratio of 71:44:21. R: proximally closer and gradually distant from anterior margin of wing. Anterior margin of wing: with a gradual downcurve prior to R1.

#### Metasoma

L: W of metasoma: 270:74. T1–T6 subequal in length, densely setose; T1–T5 longitudinally costate, T6–T7 smooth with punctae.

#### Female

Body length = 5.0 mm. Similar to male except for following character states. Anterior margin of frons arcuate with medial indentation. Mesonotum densely sculptured with depressions and longitudinal costae. Lateral propodeal carina oblique and straight, not sinuous. Lateral propodeal area short and wide. Metasoma with sparse setae. T2–T4 with basal foveae. T1–T3 with thick longitudinal costae and posteriorly with a narrow smooth area. T4 with closely spaced thin longitudinal costae. T5 densely setigerous punctate medially, flanked on either side with thin dense longitudinal costae, posteriorly smooth. T6 smooth sparse setigerous punctae.

#### Remarks

The holotype and paratype (allotype) present in MCSN, are in good condition.

Note: The labels are not by Kieffer. He merely attached tickets with numbers to the pins of the specimens he studied. The specimens he returned to the museum were accompanied by a letter to the Director of the museum stating that the male labelled ‘38’ is ‘*Sparasion coeruleum* K’ and that ‘39 = 38’ with ‘39’ being the female of the species.

The labels with the names of the new species were attached by Gestro, Vice Director, MCSN in 1905. More recently the red labels of the holotype and allotype were written and affixed by Dr. Roberto Poggi. (personal communication, Dr. Roberto Poggi).

### *Sparasion cullaris* Kozlov & Lê (Figs. 17I, 18I, 19, 27A–G)

**Fig. 27:**
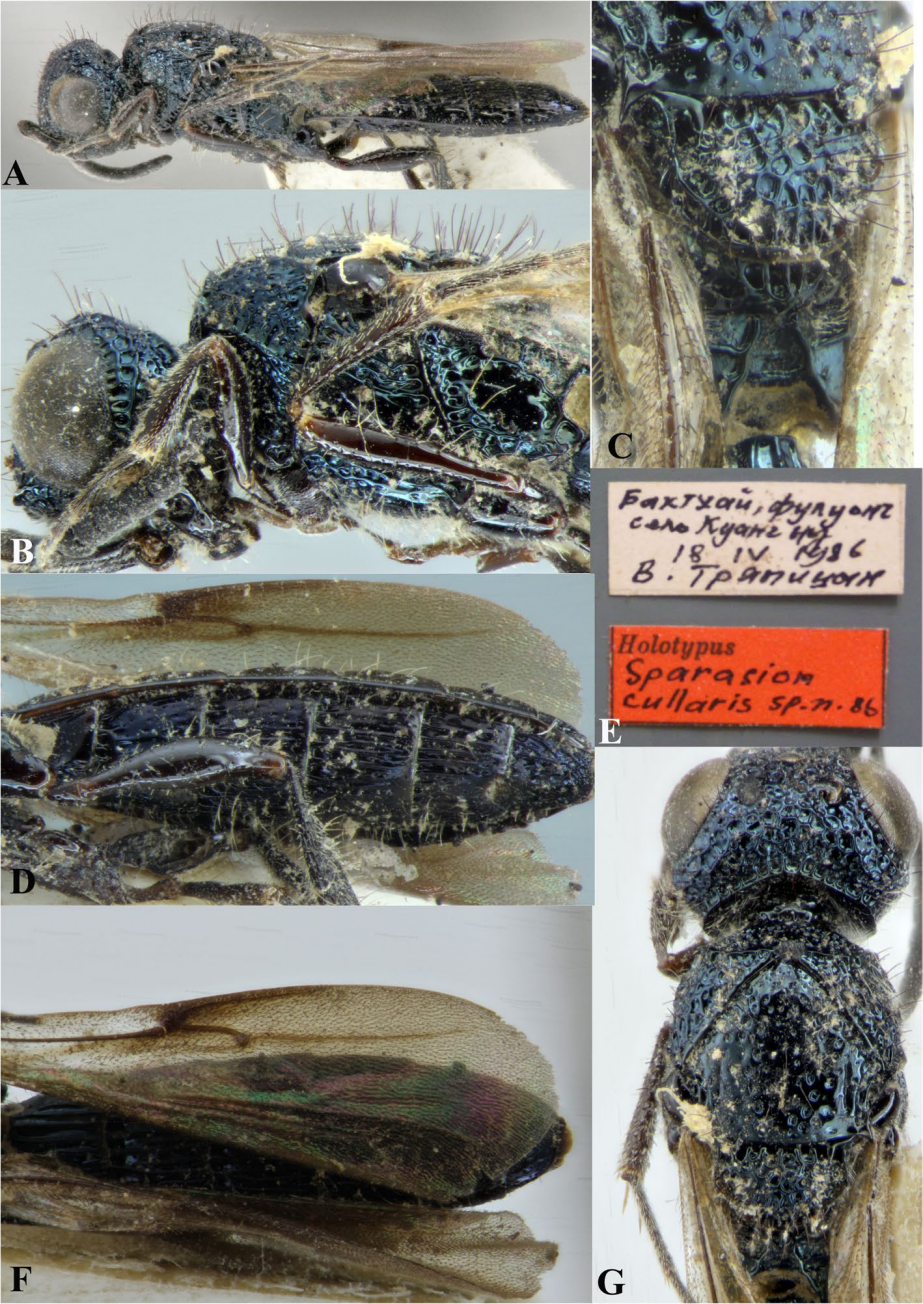
*Sparasion cullaris* Kozlov and Lê, female holotype. **A** Habitus, lateral view. **B** Head and pleuron. **C** Mesoscutellum and lateral propodeal area. **D** Wing and sternites. **E** Type labels. **F** Metasoma and wings. **G** Head and mesonotum (Photos: Drs. N. F. Johnson © Ohio State University and E. J. Talamas, Florida Department of Agriculture and Consumer Services)

*Sparasion cullaris* Kozlov & Lê, 2000: 202, 203, 204, 355. Original description, keyed.

#### Diagnosis

*Sparasion cullaris* is close to *S. travancoricus* and *S. meghmalhari* sp. n. but differs from them in the following characters: in *S. cullaris* speculum of mesopleuron is with transverse carinae and sparse setae; ventral mesopleuron is foveate, interspersed with transverse carinae. Conversely, in *S. travancoricus* speculum of mesopleuron is transversely carinate interspersed with dense setigerous punctae; ventral mesopleuron is densely foveate. In *S. meghmalhari* sp. n. speculum of mesopleuron is transversely carinate interspersed with dense setigerous foveae; ventral mesopleuron anteriorly with dense polygonal cells and posteriorly smooth with setigerous punctae.

#### Material examined

Holotype, female, **VIETNAM**: Thai Nguyen: Phu Luong*,* 18.IV.1986, leg. V. Triapitxưn.

### Description

Female body length = 4.7–5.2 mm.

#### Colour

Head, mesosoma and metasoma steel blue; tegula black-brown; all legs black-brown; antenna black-brown; mandible black.

#### Head

Setation on head: dense. Setation of compound eye: glabrous. Number of transverse ledges on upper frons: one. Sculpture on vertex: anteriorly with polygonal cells; a smooth area present posterior to lateral ocellus. Sculpture of posterior orbital furrow: with rectangular depressions. Sculpture on gena: dorsally smooth with punctae. Sculpture on A1: densely setigerous punctate.

#### Mesosoma

Sculpture of dorsal pronotum: with setigerous depressions. L: W of mesoscutum: 85:87. Sculpture of mesoscutum: smooth with sparse setigerous foveae and punctae; a ligula present anteromedially. Notaulus: present. Sculpture of notaulus: foveate. Mesoscutal humeral sulcus: with depressions. Mesoscutal suprahumeral sulcus: with depressions. Parapsidal line: indicated as deep furrow. Scutoscutellar sulcus: foveate. L: W of mesoscutellum: 42:57. Sculpture of mesoscutellum: with compact polygonal cells, and an incomplete transverse furrow anteriorly. Sculpture of dorsellum: entirely foveate except for smooth area posterolaterally, posterior margin projecting medially. Sculpture of inner lateral propodeal area: smooth, with medial transverse carina and dense anterior pilosity. Lateral propodeal carina: sinuous. Posterior propodeal projection: rounded, extending on to anterior margin of T1. Sculpture of metasomal depression: smooth with sparse pilosity and two pairs of transverse carinae. Plical area: anteriorly densely setose and posteriorly with depressions. Sculpture of lateral pronotal area: smooth. Posterior pronotal sulcus: foveate. Speculum of mesopleuron: transversely carinate, not setose. Prespecular sulcus: foveate. Mesepimeral sulcus: foveate. Mesepimeral area: smooth, narrower than mesepimeral sulcus. Sculpture of femoral depression: smooth. Mesopleural pit: present. Sculpture of ventral mesopleuron: foveate interspersed with transverse carinae and dense setae. Sculpture of metapleuron: dorsal metapleural area smooth; ventral metapleural area dorsally smooth and ventrally with polygonal cells bearing brown setae. Metapleural sulcus: foveate. Paracoxal sulcus: with polygonal cells.

#### Fore wing

Transparency: strongly infuscate. Lengths of R1: 2 × r-rs. R: basally closer and remainder distant from anterior margin of wing. Anterior margin of wing: downcurved prior to R1.

#### Metasoma

L: W of metasoma: 3 × as long as wide. Sculpture of T1–T5: longitudinally costate with smooth area laterally with sparse setigerous punctae and posteriorly smooth. Sculpture of T6: smooth with setigerous punctae.

#### Male

Briefly described by Kozlov and Lê [19]. Not examined in this study.

#### Remarks

The holotype is present in IEBR, Vietnam.

### *Sparasion darbari* Veenakumari sp. n. (Figs. 17J, 18J, 21E, 28A–F)

**Fig. 28:**
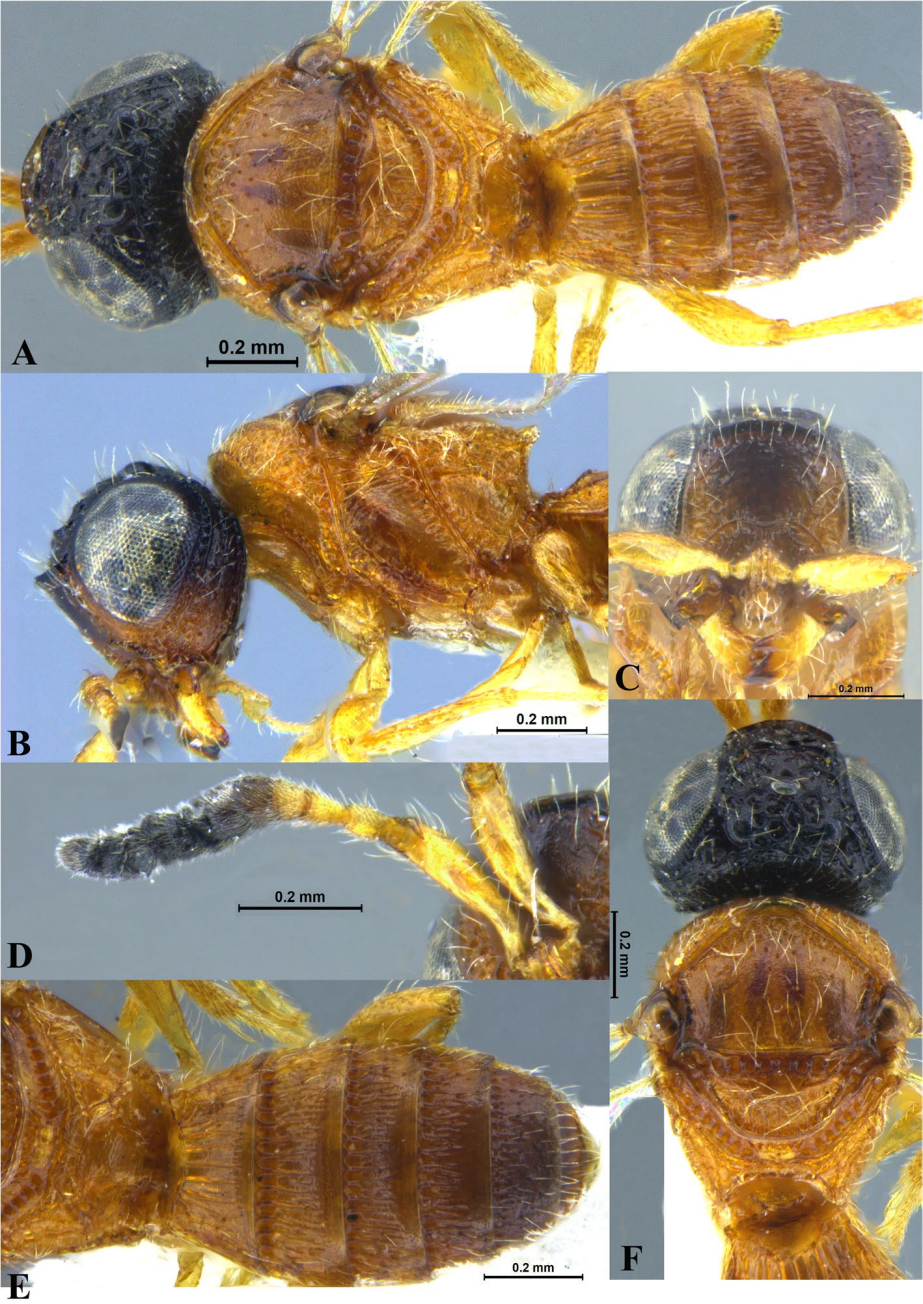
*Sparasion darbari ***sp. n**., female holotype. **A** Habitus, dorsal view. **B** Head and pleuron. **C** Frons. **D** Antenna. **E** Metasoma. **F** Head and mesonotum

urn:lsid:zoobank.org:act:4810DF12-D1A0-4C17-970A-89848B8A7AC4

#### Diagnosis

*Sparasion darbari* sp. n. is close to *S. bilahari* sp. n. The distinguishing characters are given above under the latter species.

#### Material examined

Holotype: Female, (ICAR/NBAIR/P4736), INDIA: Tamil Nadu: Yercaud, HRS, 11°47′44ʺN 78°12′42ʺE, 1399 m, YPT, 06.VIII.2014.

### Description

Female body length = 2.2 mm (*n* = 1).

#### Colour

Head dorsally black, mesoscutum yellow-brown with brown patches; frons and lower gena brown; mesoscutellum, dorsellum and lateral propodeal area yellow, metanotum brown-yellow; T1–T2 yellow-brown, remaining tergites brown; legs yellow-brown; radicle, A1–A4 yellow, A5 brown, remaining antennomeres black; mandibles yellow with teeth dark brown.

#### Head

1.3 × as wide as high, 1.1 × as high as long. Setation on head: sparse. IOS: 0.6 × head width, 0.8 × eye length. POL > LOL > OOL: 14.0:9.0:4.3. OOL: 0.6 × MOD. Compound eye: (L: W = 35.3:29.2). Setation of compound eye: glabrous. Anterior margin of frons: weakly sinuate. Distance from the level of anterior margin of compound eyes to anterior extension of frons in dorsal view: 1.1 × MOD. Number of transverse ledges on upper frons: three. Sculpture of upper frons: anteriorly smooth, followed by a row of effaced polygonal cells and a row of polygonal cells in front of anterior ocellus, all bearing setae. Sculpture of lower frons: dorsally with a row of setigerous foveae, medially smooth and with two rows of polygonal cells, laterally bearing setae; sparse short longitudinal carinae present above transverse carina above interantennal process. Interantennal process: 1.2 × as long as wide, smooth. Transverse carina above interantennal process: without a medial notch. Area ventral to transverse carina above interantennal process: smooth, with setigerous foveae dorsally and laterally. Sculpture on vertex: anteriorly with large polygonal cells followed by irregular transverse carinae, posteriorly smooth with setigerous punctae; anterior ocellus circumscribed by a narrow smooth area; lateral ocellus with a large triangular smooth area posteriorly. Sculpture of posterior orbital furrow: foveate. Genal carina: present. Sculpture of gena: anteriorly with sparse weak setigerous foveae and posteriorly smooth with very sparse setae. Sculpture on A1: smooth with sparse setae. A1: 3.6 × as long as wide. Length of A3: 0.3 × A1 and subequal to A2.

#### Mesosoma

Sculpture of dorsal pronotum: smooth with sparse setigerous punctae. L: W of mesoscutum: 35.2:47.4. Sculpture of mesoscutum: weakly rugose with sparse setigerous punctae. Mesoscutal humeral sulcus: foveate. Mesoscutal suprahumeral sulcus: with irregular cells. Parapsidal line: indicated as furrow. Scutoscutellar sulcus: foveate. L: W of mesoscutellum: 21.3:35.7. Sculpture of mesoscutellum: anteriorly smooth with an incomplete carina posteriorly longitudinally costate with foveae between costae. Sculpture of dorsellum: anteriorly foveate, posteriorly smooth, posterior margin with weak upcurve medially. Sculpture of outer lateral propodeal area: with depressions, sparsely setose. Sculpture of inner lateral propodeal area: anteriorly sparsely setose, posteriorly smooth and with a transverse medial carina. Lateral propodeal carina: arched. Posterior propodeal projection: rounded, not extending to anterior margin of T1. Sculpture of metasomal depression: smooth with a medial vertical carina and sparse pilosity. Plical area: anteriorly setose, posteriorly weakly rugose interspersed with striae, sparsely setose. Sculpture of propleuron: anteriorly smooth, posteriorly rugose. Sculpture of lateral pronotal area: dorsally smooth, followed by intricate sculpture. Posterior pronotal sulcus: with weak depressions. Pronotal cervical sulcus: foveate. Speculum of mesopleuron: transversely carinate, sparsely setose. Postacetabular sulcus: foveate. Prespecular sulcus: foveate, foveae wide and shallow. Mesepimeral sulcus: foveate. Posterior mesepimeral area: smooth, narrower mesepimeral sulcus. Mesopleural carina: percurrent, with a row of incomplete rectangular cells dorsally. Sculpture of femoral depression: smooth with sparse oblique striae anterodorsally. Mesopleural pit: present. Sculpture of ventral mesopleuron: dorsally with a row of rectangular cells, posteroventrally smooth with setigerous punctae. Sculpture of metapleuron: dorsal metapleural area narrow and rugose with sparse setae on anterior margin; ventral metapleural area dorsally weakly rugose and ventrally with impressions of rectangular cells. Metapleural sulcus: indicated as shallow depressions, medially present as a furrow. Paracoxal sulcus: with shallow foveae. Metapleural epicoxal sulcus: indicated as a furrow.

#### Fore wing

L: W: 171.8:68.2. Transparency: weakly infuscate. Lengths of R: R1: r-rs in ratio of 73:40:22. R: gradually distant from anterior margin of wing. Anterior margin of fore wing: with no downcurve prior to R1.

#### Metasoma

L: W of metasoma: 84.9:51.1. Ratio of length of T1: T2: T3: T4: T5: 14.4:17.7:15.3:14.8:14.6. Anterior margin of T1: weakly convex. Sculpture of T1: longitudinally costate, anterolaterally with short oblique carinae, laterally smooth with setae, posteriorly smooth. Sculpture of T2: basal foveae present, followed by short longitudinal costae, with intricate sculpture between costae; laterally smooth with sparse setae, posteriorly smooth. Sculpture of T3: same as T2, with costae shorter medially. Sculpture of T4: same as T3, with costae shorter medially. Sculpture of T5: same as T3, smooth with setigerous punctae with sparse costae sublaterally on anterior margin. Sculpture of T6: smooth with sparse setae.

#### Male

Unknown.

#### Etymology

This species is named ‘Darbari’ after a *raga*, or melodic structure, in Hindustani music (an Indian classical music tradition), derived from ‘durbar’ meaning ‘royal court’ in Persian and considered by some cognoscenti to be the ‘emperor of *ragas* and the *raga* of emperors’.

### *Sparasion deepaki* Veenakumari sp. n. (Figs. 17K, 18K, 21F, 29A–F)

**Fig. 29:**
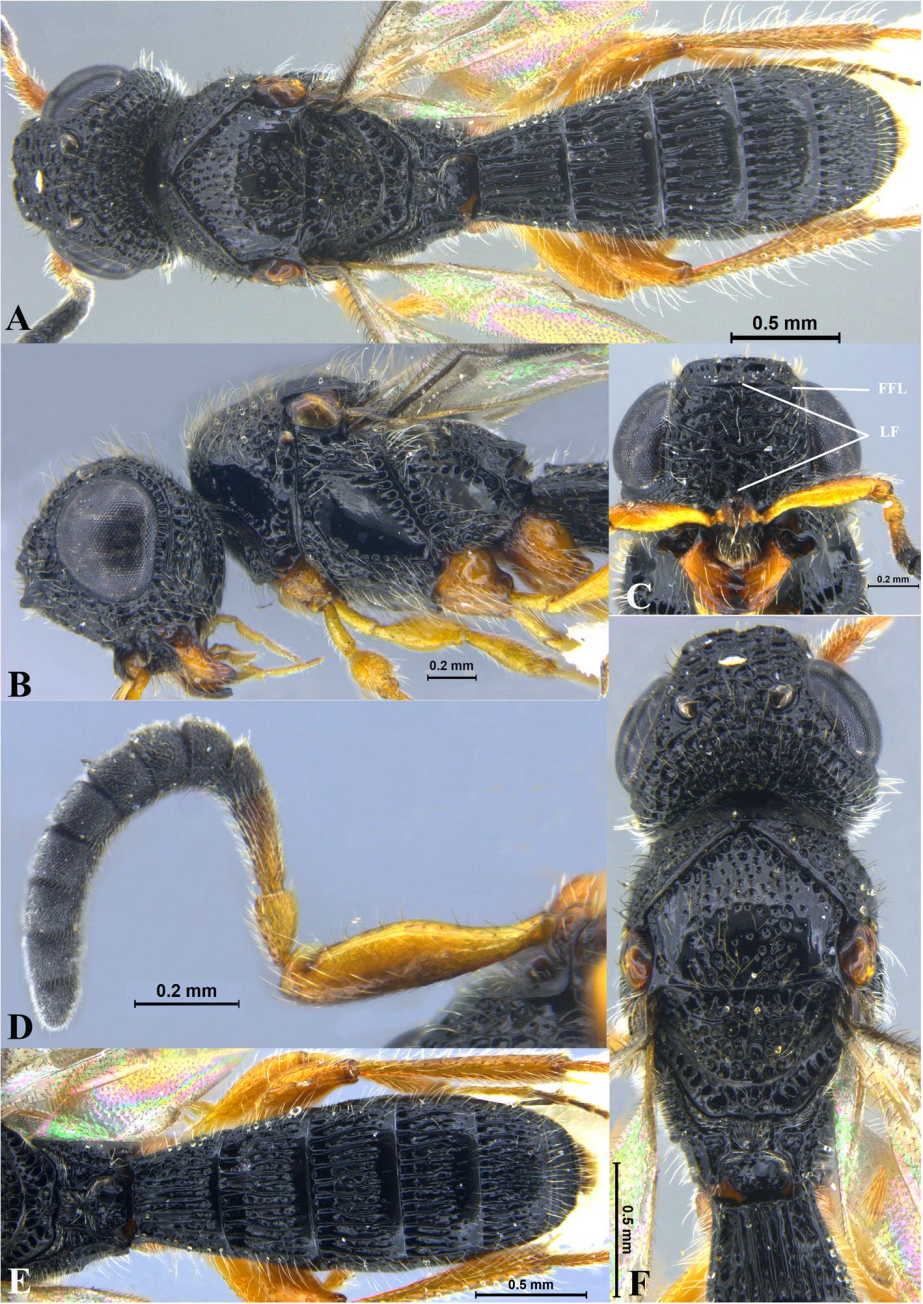
*Sparasion deepaki ***sp. n**., female holotype. **A** Habitus, dorsal view. **B** Head and pleuron. **C** Frons. **D** Antenna. **E** Metasoma. **F** Head and mesonotum. FFL-first frontal ledge; LF- lower frons

urn:lsid:zoobank.org:act:FB061713-FB9A-4FF3-A5D1-42CA63E0E410

#### Diagnosis

*Sparasion deepaki* sp. n. is close to *S. coconcus*. The distinguishing characters are given above under the latter species.

#### Material examined

Holotype: Female, (ICAR/NBAIR/P4582), **INDIA**: Karnataka: Mudigere, College of Horticulture, 13°06′54ʺN 75°37′57ʺE, 976 m, SN, 30.IX.2015.

### Description

Female body length = 4.72 mm (*n* = 1).

#### Colour

Head, mesosoma and metasoma black; tegula black with brown patches; coxa orange- brown, remainder of leg yellow-brown; radicle, A1–A2 and basal half of A3 yellow-brown; distal half of A3 brown-black, remaining antennomeres black; mandibles orange-brown with teeth black.

#### Head

1.2 × as wide as high, as high as long. Setation on head: sparse. IOS: 0.5 × head width, subequal to eye length. POL > LOL > OOL: 27.6:17.4:4.3. OOL: 0.4 × MOD. Compound eye: (L: W = 52.7:43.6). Setation of compound eye: glabrous. Anterior margin of frons: arcuate. Distance from the level of anterior margin of compound eyes to anterior extension of frons in dorsal view: 1.8 × MOD. Number of transverse ledges on upper frons: three. Sculpture of upper frons: anteriorly and posteriorly with polygonal cells and medially with a row of larger polygonal cells, anterior cells shallow. Sculpture of lower frons: with uneven transverse ribbed carinae with rows of polygonal setigerous cells between. Interantennal process: subequal in length and width, longitudinally carinate with intricate sculpture in between. Transverse carina above interantennal process: medially notched and discontinuous. Area ventral to transverse carina above interantennal process: with polygonal cells and punctae except for smooth area on inner margin. Sculpture on vertex: anteriorly with large polygonal cells, followed by smaller polygonal cells and an uneven transverse carina and posteriorly smooth with setigerous punctae; smooth area absent around anterior ocellus; a smooth triangular area present posterior to lateral ocellus. Sculpture of posterior orbital furrow: foveate. Genal carina: present. Sculpture of gena: with polygonal cells except for smooth patch posteroventrally, sparsely setose. Sculpture of A1: smooth with sparse setae. A1: 4.1 × as long as wide. A3: 0.4 × the length of A1 and 1.4 × the length of A2.

#### Mesosoma

Sculpture of dorsal pronotum: with setigerous depressions. L: W of mesoscutum: 63.4:84.3. Sculpture of mesoscutum: smooth with dense foveae anteromedially and polygonal cells posteromedially. Notaulus: present. Sculpture of notaulus: foveate. Mesoscutal humeral sulcus: foveate. Mesoscutal suprahumeral sulcus: with depressions. Parapsidal line: indicated as furrow. Scutoscutellar sulcus: foveate. L: W of mesoscutellum: 37.0:56.0. Sculpture of mesoscutellum: with compact polygonal cells and an incomplete transverse furrow anteriorly. Sculpture of dorsellum: anteriorly foveate, posteriorly smooth with sparse punctae, posterior margin with an upcurve medially. Sculpture of outer lateral propodeal area: with depressions, sparsely setose. Sculpture of inner lateral propodeal area: anteriorly densely setose, posteriorly unevenly foveate. Lateral propodeal carina: anteriorly perpendicular and posteriorly arched. Posterior propodeal projection: rounded, not extending to anterior margin of T1. Sculpture of metasomal depression: anteriorly densely setose and posteriorly with intricate sculpture. Plical area: anteriorly sparsely setose, medially unevenly foveate, posteriorly smooth. Sculpture of propleuron: weakly rugose. Sculpture of lateral pronotal area: smooth. Posterior pronotal sulcus: with large ovoid cells. Pronotal cervical sulcus: foveate. Speculum of mesopleuron: transversely carinate interspersed with sparse foveae, sparsely setose. Postacetabular sulcus: foveate. Prespecular sulcus: foveate. Mesepimeral sulcus: foveate. Posterior mesepimeral area: smooth, narrower than mesepimeral sulcus. Mesopleural carina: indicated anteriorly. Sculpture of femoral depression: smooth. Mesopleural pit: present. Sculpture of ventral mesopleuron: with polygonal cells, densely setose. Sculpture of metapleuron: dorsal metapleural area smooth with long dense setae on anterior margin; ventral metapleural area dorsally smooth and ventrally with polygonal cells. Metapleural sulcus: with circular to ovoid cells. Paracoxal sulcus: with ovoid cells. Metapleural epicoxal sulcus: with polygonal cells.

#### Fore wing

L: W: 278.0:109.2. Transparency: weakly infuscate. Lengths of R: R1: r-rs in ratio of 80:66:29. R: curving towards anterior margin of wing at 0.6 × its length, later deflecting away from anterior margin towards bulla. Anterior margin of fore wing: downcurved prior to R1.

#### Metasoma

L: W of metasoma: 211.2:81.1. Ratio of length of T1: T2: T3: T4: T5: 39.6:39.6:39.6:36.5:33.0. Anterior margin of T1: straight with a small spine anteromedially. Sculpture of T1: medially longitudinally costate with uneven punctae between costae, laterally with intricate sculpture and posteromedially smooth. Sculpture of T2: basal foveae present, followed by longitudinal costae with depressions between costae; laterally smooth with setigerous punctae and posteriorly smooth. Sculpture of T3: same as T2, with smaller basal foveae. Sculpture of T4: same as T2 with smaller basal foveae. Sculpture of T5: basal foveae present, followed by dense setigerous punctae medially and remainder with longitudinal costae except for smooth area posteriorly. Sculpture of T6: smooth with basal foveae.

#### Male

Unknown.

#### Etymology

This species is named ‘Deepak’ after the melodic structure or *raga* in Hindustani music – an Indian classical music tradition - called the *raga* or melody of fire, and its consummate rendition on a rare occasion by the musician Tansen in the Mughal emperor Akbar’s court is said to have set the music hall on fire.

### *Sparasion domes* Kozlov & Lê (Figs. 17L, 19, 30A–F)

**Fig. 30:**
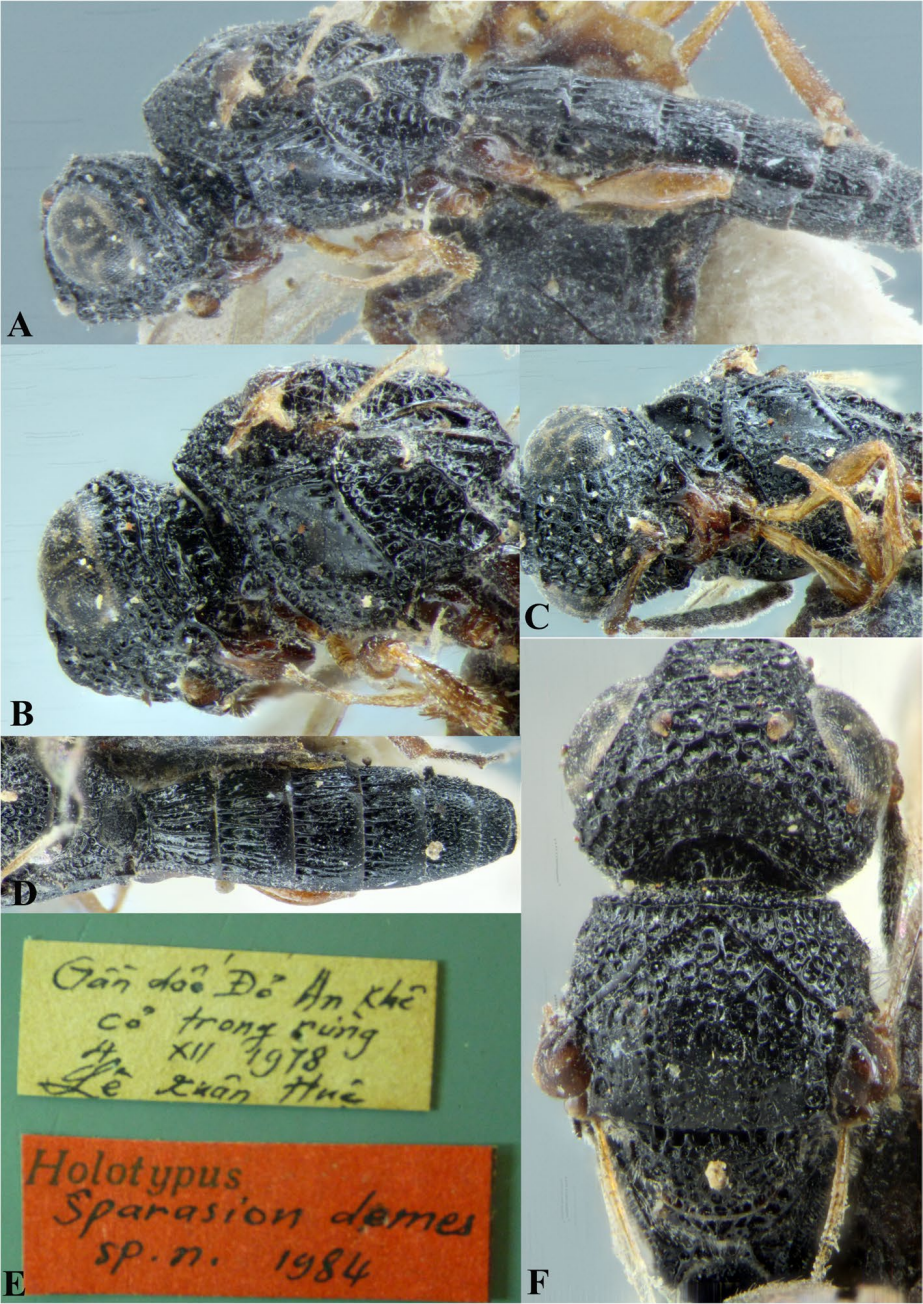
*Sparasion domes* Kozlov and Lê, male holotype. **A** Habitus, lateral view. **B** Head and pleuron. **C** Frons and pleuron, lateroventral view. **D** Metasoma. **E** Type labels. **F** Head and mesonotum (Photos: Drs. N. F. Johnson © Ohio State University and E. J. Talamas, Florida Department of Agriculture and Consumer Services)

*Sparasion domes* Kozlov & Lê, 2000: 203, 205, 356. Original description, keyed.

#### Diagnosis

*Sparasion domes* is close to *S. bhupali* sp. n. but differs from it in the following characters: in *S. domes* posterior mesoscutum is smooth with sparse punctae; notaulus is present; posterior propodeal projections are elongate and narrow. Conversely, in *S. bhupali* sp. n. mesoscutum is with longitudinal carinae posteromedially; notaulus is absent; posterior propodeal projections are short and wide.

#### Material examined

Holotype, male, **VIETNAM**: Gia Lai: An Khe*:* Buon Luoi, 4.XII.1978, leg. Lê, X. H.

### Description

Male body length: 3.4 mm.

#### Colour

Head, mesosoma and metasoma black; tegula brown-black; all coxae honey-brown, remainder of legs brown; radicle, A1–A2 honey-brown, remaining antennomeres black-brown; mandibles red-brown.

#### Head

Setation on head: sparse. Setation of compound eye: glabrous. OOL: < diameter of lateral ocellus. Anterior margin of frons: arcuate. Number of transverse ledges on upper frons: two. Sculpture of upper frons: with shallow polygonal cells. Sculpture of lower frons: entirely with polygonal cells. Transverse carina above interantennal process: with a medial notch. Area ventral to transverse carina above interantennal process: weakly rugose. Sculpture on vertex: anteriorly with polygonal cells, posteriorly smooth with setigerous punctae; irregular smooth area present on posterior margin of lateral ocellus. Sculpture of posterior orbital furrow: dorsally foveate, ventrally punctate. Genal carina: present. Sculpture of gena: anteriorly with polygonal cells and posteriorly smooth. Sculpture on A1: smooth with sparse long setae.

#### Mesosoma

Sculpture of dorsal pronotum: with dense depressions. L: W of mesoscutum: 39:46. Sculpture of mesoscutum: anteriorly with polygonal cells and posteriorly smooth with sparse punctae. Notaulus: present. Sculpture of notaulus: foveate. Mesoscutal humeral sulcus: with depressions. Mesoscutal suprahumeral sulcus: with depressions. Parapsidal line: indicated as furrow. Scutoscutellar sulcus: foveate. L: W of mesoscutellum: 20.0:40.0. Sculpture of mesoscutellum: closely packed foveae, with anterior incomplete transverse carinae. Sculpture of dorsellum: anteriorly foveate, posteriorly smooth with punctae, posterior margin rounded. Sculpture of outer lateral propodeal area: with uneven shallow depressions. Sculpture of inner lateral propodeal area: anteriorly sparsely setose followed a medial transverse carina, posteriorly uneven depressions. Lateral propodeal carina: weakly sinuous. Posterior propodeal projection: rounded, not extending to anterior margin of T1. Sculpture of metasomal depression: sparsely setose. Plical area: with uneven depressions. Sculpture of lateral pronotal area: smooth. Speculum of mesopleuron: with two transverse carinae. Postacetabular sulcus: foveate. Prespecular sulcus: weakly foveate. Mesepimeral sulcus: with shallow incomplete foveae. Mesepimeral area: smooth, narrower than mesepimeral sulcus. Mesopleural carina: present with a row of shallow depressions dorsally. Sculpture of femoral depression: smooth. Mesopleural pit: present. Sculpture of ventral mesopleuron: smooth with sparse foveae. Sculpture of metapleuron: dorsal metapleural area narrow and smooth; ventral metapleural area dorsally smooth and ventrally with irregular shallow cells followed by a rugose area. Metapleural sulcus: foveate. Paracoxal sulcus: foveate. Metapleural epicoxal sulcus: indicated as a furrow.

#### Fore wing

L: W: 2.5 × as long as wide. Transparency: weakly infuscate. Length of R1: 2.5 × r-rs.

#### Metasoma

L: W of metasoma: 78:28. Ratio of length of T1: T2: T3: T4: T5: 36:41:38:33:29. Anterior margin of T1: weakly convex. Sculpture of T1: medially longitudinally costate, posteriorly smooth and laterally with intricate sculpture. Sculpture of T2: basal foveae present, followed by longitudinal costae; laterally with intricate sculpture, posteriorly smooth. Sculpture of T3: same as T2 with sparse punctae posteriorly. Sculpture of T4: same as T3. Sculpture of T5: entirely punctate except for short longitudinal carinae sublaterally and smooth area posteriorly. Sculpture of T6: smooth with punctae.

#### Female

Unknown.

#### Remarks

The holotype is present in IEBR, Vietnam.

### *Sparasion elbakyanae* Veenakumari sp. n. (Figs. 17 M, 18L, 21G, 31A–F)

**Fig. 31:**
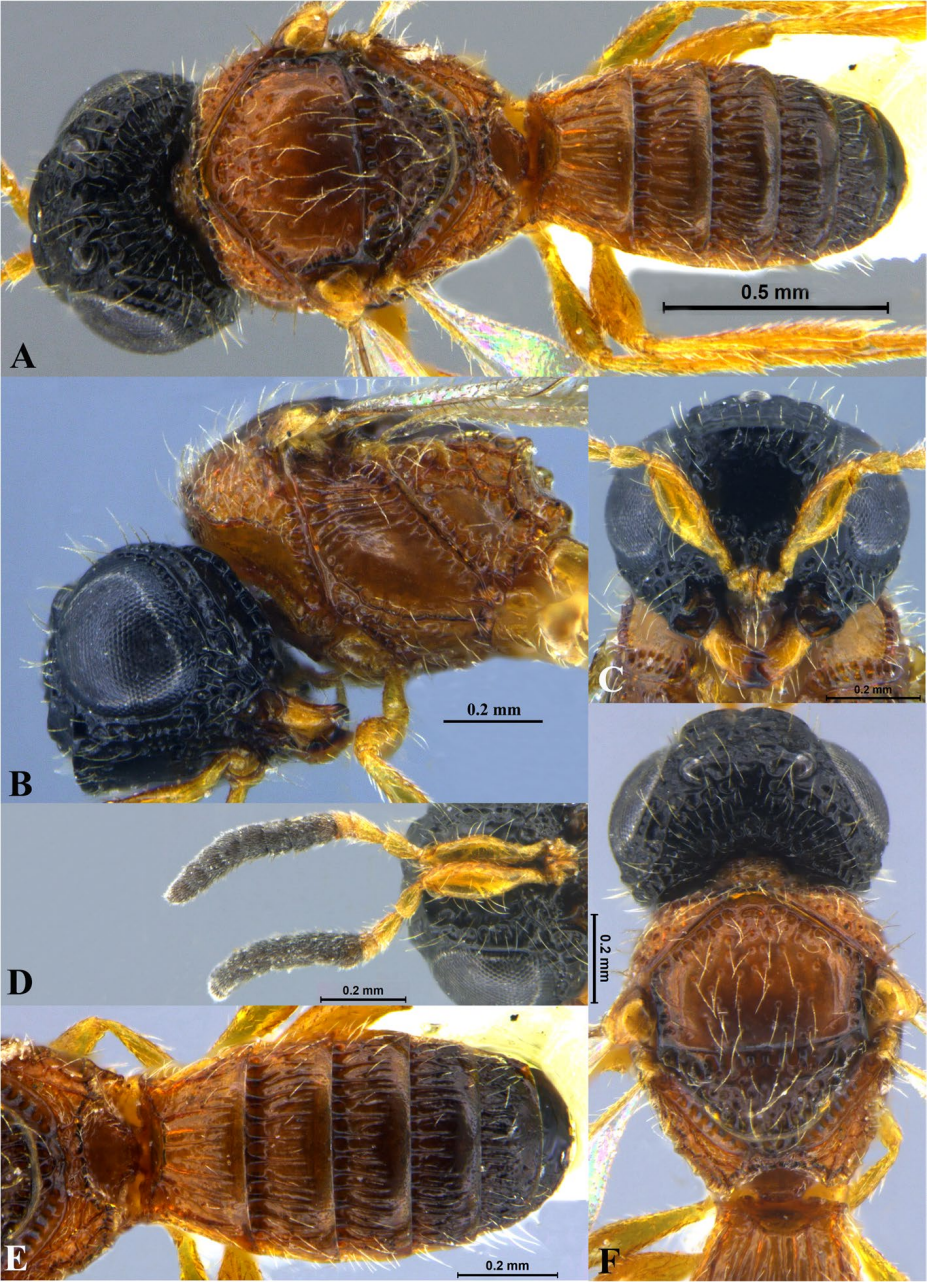
*Sparasion elbakyanae ***sp. n**., female holotype. **A** Habitus, dorsal view. **B** Head and pleuron. **C** Frons. **D** Antennae. **E** Metasoma. **F** Head and mesonotum

urn:lsid:zoobank.org:act:D44233E8-CB45-4363-8289-6FF6E4409B85

#### Diagnosis

*Sparasion elbakyanae* sp. n. is close to *S. kalyani* sp. n. and *S. kanakangi* sp. n. but differs from them in the following characters: in *S. elbakyanae* sp. n. outer lateral propodeal area is almost smooth and mesoscutellum predominantly setigerous foveate. Conversely, in the latter two species outer lateral propodeal area is punctate-foveate and mesoscutellum is predominantly smooth.

#### Material examined

Holotype: Female, (ICAR/NBAIR/P4629), **INDIA**: Tamil Nadu: Hosur, Uddanapalli, 12°37′28ʺN 77°55′29ʺE, 758 m, YPT, 02.XII.2014. Paratypes: 8 females (ICAR/NBAIR/P4630–P4637), Tamil Nadu: Hosur, Uddanapalli, 12°37′28ʺN 77°55′29ʺE, 758 m, YPT, 02.XII.2014; 4 females (ICAR/NBAIR/P4638–P4641), Tamil Nadu: Hosur, Uddanapalli, 12°37′28ʺN 77°55′29ʺE, 758 m, YPT, 29.XI.2014.

### Description

Female body length = 2.34–2.58 mm (*n* = 10).

#### Colour

Head black; mesoscutum, metanotal trough honey-brown; mesoscutellum and dorsellum brown-black; propodeum and T1 orange-brown; T2–T3 red-brown; remaining tergites brown-black; pronotum, pleuron honey-brown; tegula yellow-brown; legs yellow-brown; radicle, A1–A4 yellow, remaining antennomeres black-brown; mandibles orange-yellow with teeth dark brown.

#### Head

1.2 × as wide as high, 1.1 × as high as long. Setation on head: sparse. IOS: 0.5 × head width, subequal to eye length. POL > LOL > OOL: 20.5:13.0:4.6. OOL: 0.6 × MOD. Compound eye: (L: W = 35.7:32.8). Setation of compound eye: glabrous. Anterior margin of frons: arcuate. Distance from level of anterior margin of compound eyes to anterior extension of frons in dorsal view: 1.4 × MOD. Number of transverse ledges on upper frons: three. Sculpture of upper frons: anteriorly with two rows of effaced polygonal cells followed by one row of polygonal cells bearing setae. Sculpture of lower frons: medially smooth surrounded by polygonal cells bearing setae. Interantennal process: 1.5 × as long as wide, smooth with medial furrow. Transverse carina above interantennal process: with a medial notch. Area ventral to transverse carina above interantennal process: entirely setigerous punctate. Sculpture on vertex: anteriorly with large polygonal cells, followed by a row of smaller polygonal cells and an irregular transverse carina, posteriorly smooth with setigerous punctae; anterior ocellus with a narrow smooth area around; irregular smooth area present on anterior and posterior margin of lateral ocellus. Sculpture of posterior orbital furrow: dorsally foveate and ventrally with rectangular cells. Genal carina: present. Sculpture of gena: anteriorly with polygonal cells and posteriorly smooth, sparsely setose. Sculpture on A1: smooth with sparse long setae. A1: 3.1 × as long as wide. Length of A3: 0.3 × A1 and subequal to A2.

#### Mesosoma

Sculpture of dorsal pronotum: with setigerous punctae and depressions. L: W of mesoscutum: 37.1:50.7. Sculpture of mesoscutum: smooth with setigerous sparse foveae. Notaulus: absent. Mesoscutal humeral sulcus: with depressions. Mesoscutal suprahumeral sulcus: with ovoid cells. Parapsidal line: indicated as a furrow. Scutoscutellar sulcus: foveate. L: W of mesoscutellum: 20.0:36.6. Sculpture of mesoscutellum: setigerous foveate except for a smooth patch anteromedially. Sculpture of dorsellum: anteriorly foveate, posteriorly smooth, posterior margin sinuous. Sculpture of outer lateral propodeal area: anteriorly with depressions, posteriorly smooth, sparsely setose. Sculpture of inner lateral propodeal area: anteriorly sparsely setose, posteriorly smooth. Lateral propodeal carina: arched. Posterior propodeal projection: rounded, not extending to anterior margin of T1. Sculpture of metasomal depression: with a row of depressions and sparse pilosity. Plical area: anteriorly sparsely setose, posteriorly with smooth shallow depressions. Sculpture of propleuron: smooth. Sculpture of lateral pronotal area: smooth. Posterior pronotal sulcus: with ovoid cells. Pronotal cervical sulcus: foveate. Speculum of mesopleuron: transversely carinate, very sparsely setose. Postacetabular sulcus: foveate. Prespecular sulcus: foveate. Mesepimeral sulcus: foveate. Posterior mesepimeral area: smooth, narrower than mesepimeral sulcus. Mesopleural carina: percurrent, with a row of shallow depressions dorsally. Sculpture of femoral depression: smooth. Mesopleural pit: present. Sculpture of ventral mesopleuron: anteriorly with uneven setigerous depressions, posteriorly smooth with sparse setigerous punctae. Sculpture of metapleuron: dorsal metapleural area very narrow and smooth with sparse setae on anterior margin; ventral metapleural area dorsally smooth and ventrally with sparse foveae. Metapleural sulcus: foveate. Paracoxal sulcus: with ovoid cells. Metapleural epicoxal sulcus: with shallow depressions.

#### Fore wing

L: W: 153.0:67.9. Transparency: weakly infuscate. Lengths of R: R1: r-rs in ratio of 64:23:21. R: basally closer and gradually distant from anterior margin of wing. Anterior margin of fore wing: with a downcurve prior to R1.

#### Metasoma

L: W of metasoma: 81.7:47.0. Ratio of length of T1: T2: T3: T4: T5: 16.7:17.2:15.8:14.0:12.5. Anterior margin of T1: convex. Sculpture of T1: medially longitudinally costate, posteriorly smooth and anterolaterally smooth and posterolaterally with short longitudinal costae. Sculpture of T2: basal foveae present, followed by longitudinal costae; posterior 1/3 smooth with weak punctae. Sculpture of T3: same as T2. Sculpture of T4: same as T2. Sculpture of T5: anterior half longitudinally costate-punctate, posterior half smooth. Sculpture of T6: smooth.

#### Male

Unknown.

#### Etymology

This species is named in honour of Alexandra Elbakyan, the intrepid crusader from the Republic of Kazakhstan, who by founding the website Sci-Hub, took on the giants of the publishing industry in her quest to democratize knowledge by ‘removing all barriers in access to scientific knowledge’.

### *Sparasion formosus* Kieffer (Figs. 17N, 19, 32A–F, 33A)

**Fig. 32:**
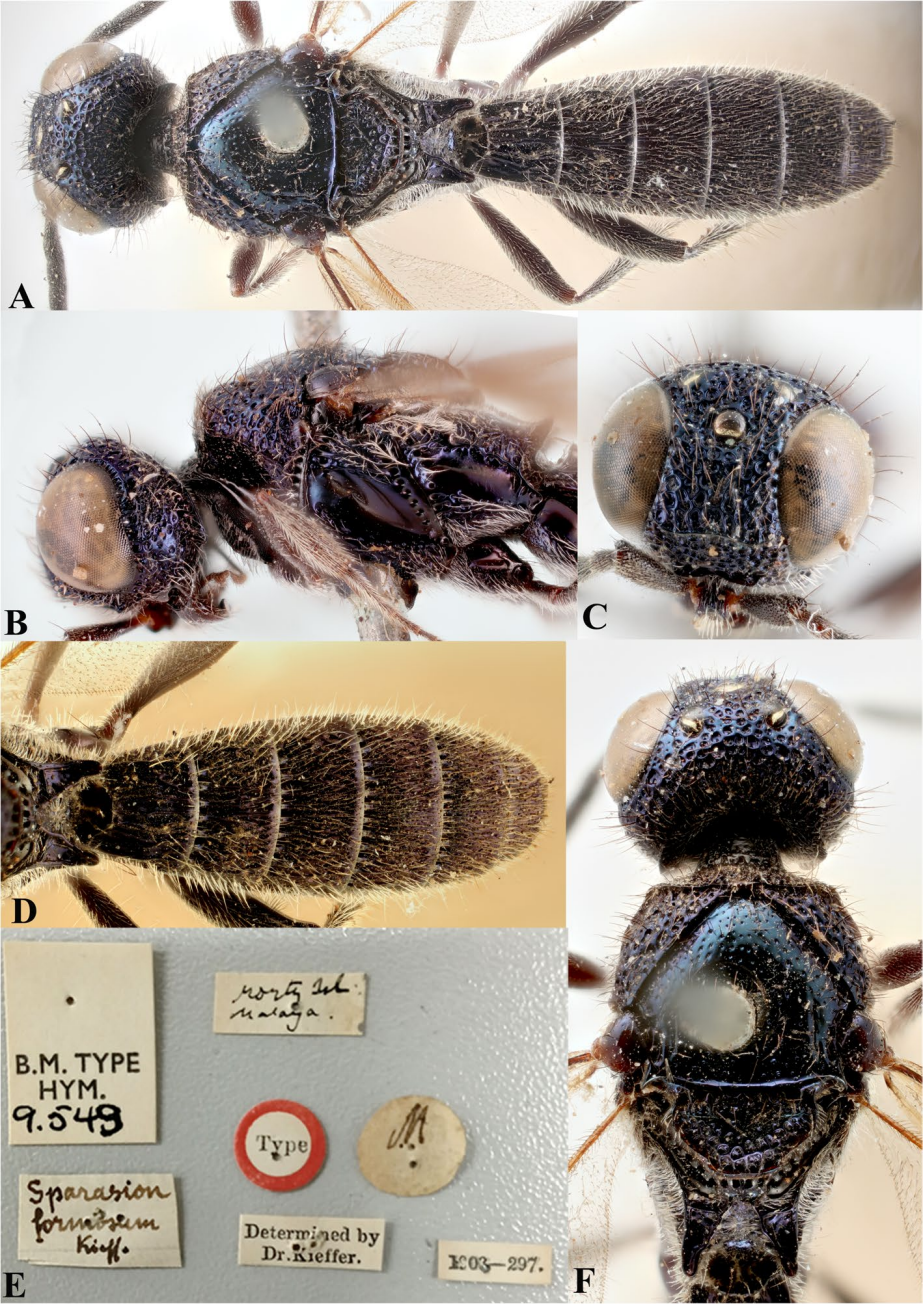
*Sparasion formosus* Kieffer, male holotype. **A** Habitus, dorsal view. **B** Head and pleuron. **C** Frons. **D** Metasoma. **E** Type labels. **F** Head and mesonotum (Photos: Dr. Andrew Polaszek © NHMUK)

**Fig. 33:**
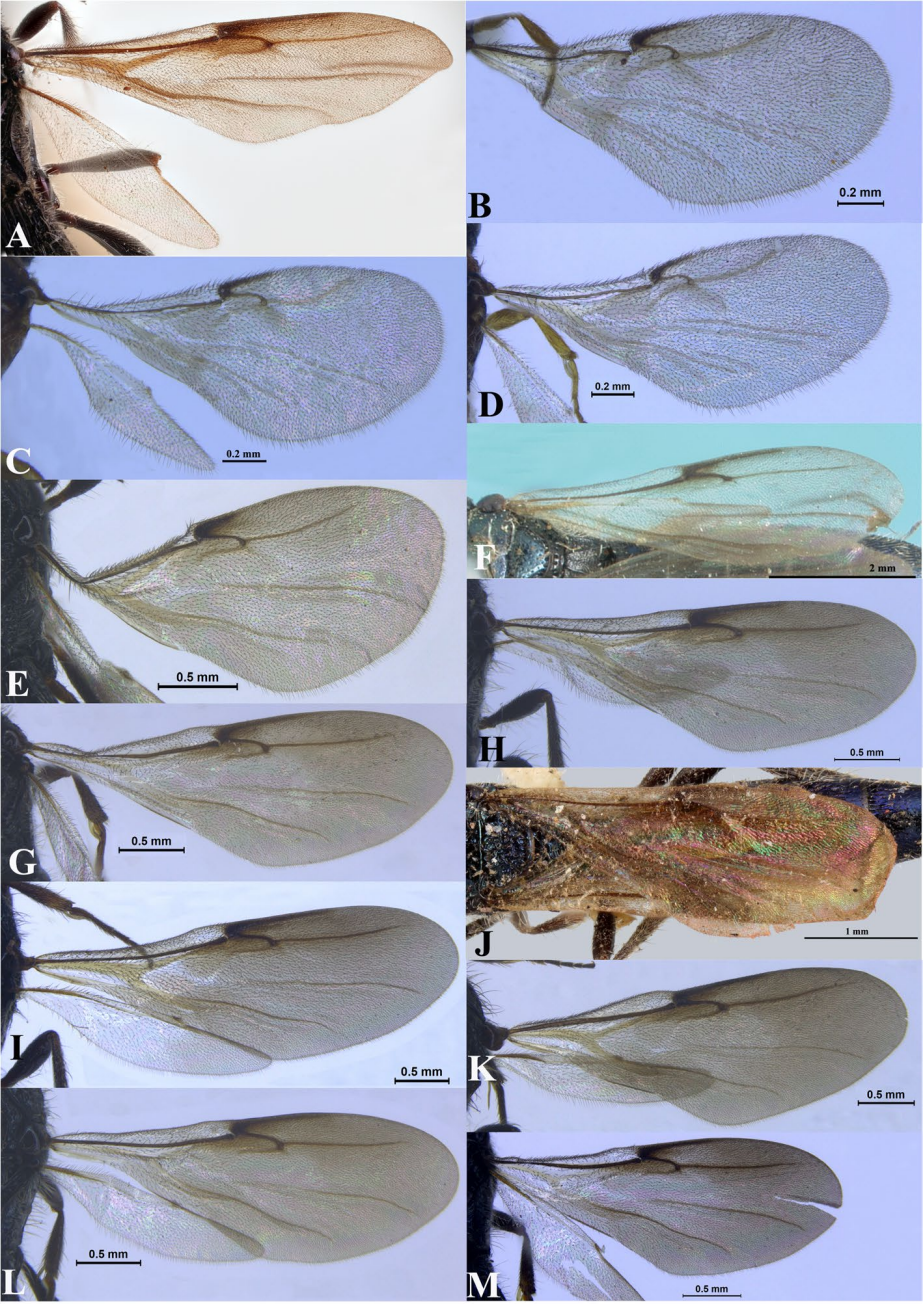
Wings. **A ***Sparasion formosus*. **B ***S. hindoli. ***C*** S. kalyani. ***D ***S. kanakangi*. **E ***S. karivadana*. **F ***S. lividus*. **G ***S. manavati*. **H ***S. meghmalhari*. **I ***S. pahadi*. **J ***S. philippinensis*. **K ***S. rupavati. ***L ***S. ratnangi. ***M ***S. salagami*

*Sparasion formosum* Kieffer, 1910: 311

*Sparasion formosus*: Kieffer, 1926:284, 294. Description, emendation, keyed.

*Sparasion formosus*: Masner, 1965: 97. Type information.

#### Diagnosis

This species is distinct in having very dense white setae on the metasoma and ventral metapleuron.

#### Material examined

Holotype: Male, (B.M.TYPE HYM. 9.549; K03-297), **INDONESIA**: Maluku Utara (North Moluccas), Morotai (mentioned as Morty Island, Malaya on label, Malakka in description).

### Description

Male body length = 7 mm.

#### Colour

Head, mesosoma and metasoma steel blue; tegula brown; all legs shining blue-brown; radicle, basal and apical A1 and A2 red-brown, remaining antennomeres black-brown; mandible brown-black.

#### Head

Setation on head: dense. Setation of compound eye: glabrous. Anterior margin of frons: arcuate with medial indentation. Distance from the level of anterior margin of compound eyes to anterior extension of frons in dorsal view: < MOD. Number of transverse ledges on upper frons: one. Sculpture of upper frons: with circular and polygonal cells bearing setae, with smooth interstices. Sculpture of lower frons: medially transversely carinate interspersed with polygonal cells. Transverse carina above interantennal process: with an acute medial notch. Sculpture on vertex: anteriorly with circular cells with setae and posteriorly smooth with setigerous punctae; a smooth area present around anterior ocellus; a smooth area present posterior to lateral ocellus. Sculpture of posterior orbital furrow: dorsally with small foveae and ventrally with large depressions. Genal carina: absent. Sculpture of gena: smooth with setigerous circular cells, sparsely setose. Sculpture on A1: densely setigerous punctate.

#### Mesosoma

Sculpture of dorsal pronotum: with setigerous depressions and foveae. L: W of mesoscutum: 75:80. Sculpture of mesoscutum: smooth with sparse setigerous punctae. Notaulus: absent. Mesoscutal humeral sulcus: with elongate depressions. Mesoscutal suprahumeral sulcus: dorsally smooth and posteriorly with depressions. Parapsidal line: indicated as furrow. Scutoscutellar sulcus: foveate. L: W of mesoscutellum: 34:52. Sculpture of mesoscutellum: anteromedially smooth with two rows of foveae on posterior margin. Sculpture of dorsellum: anteriorly foveate, posteriorly smooth, posterior margin almost straight. Sculpture of outer lateral propodeal area: densely setose concealing the sculpture. Sculpture of inner lateral propodeal area: smooth, with medial transverse carina and sparse pilosity anteriorly. Lateral propodeal carina: sinuous. Posterior propodeal projection: rounded, extending on to anterior margin of T1. Sculpture of metasomal depression: densely setose. Plical area: anteriorly densely setose and posteriorly with smooth shallow depressions. Sculpture of propleuron: smooth. Sculpture of lateral pronotal area: smooth. Posterior pronotal sulcus: foveate. Pronotal cervical sulcus: not foveate. Speculum of mesopleuron: transversely carinate, interspersed with foveae, densely setose. Mesepimeral sulcus: foveate. Mesepimeral area: smooth, wider than mesepimeral sulcus. Mesopleural carina: not distinct. Sculpture of femoral depression: smooth. Mesopleural pit: present. Sculpture of ventral mesopleuron: smooth with setigerous foveae. Sculpture of metapleuron: dorsal metapleural area narrow and smooth with long setae on anterior margin; ventral metapleural area dorsally smooth and ventrally with foveae and polygonal cells bearing dense white setae. Metapleural sulcus: foveate. Paracoxal sulcus: foveate. Metapleural epicoxal sulcus: hidden by dense setae.

#### Fore wing

L: W: 267:77. Transparency: weakly infuscate. Lengths of R: R1: r-rs in ratio of 121:80:33. R: basally closer and distally distant form anterior margin of wing. Anterior margin of wing: with a weak downcurve prior to R1.

#### Metasoma

L: W of metasoma: 260:98. Ratio of length of T1: T2: T3: T4: T5: 48:48:48:48:42. Anterior margin of T1: weakly convex. Sculpture of T1: medially with longitudinally ribbed costae, laterally smooth with dense setae, posteriorly smooth. Sculpture of T2: basal foveae present, followed by widely spaced longitudinal ribbed costae; laterally smooth with dense setae, and posteriorly smooth. Sculpture of T3: basal foveae present, entirely smooth with dense setae except for longitudinal costae sublaterally. Sculpture of T4: same as T3. Sculpture of T5: basal foveae present, densely setose. Sculpture of T6: same as T5.

#### Female

Unknown.

#### Remarks

Holotype preserved in good condition in NHM, London.

### *Sparasion hindoli* Veenakumari sp. n. (Figs. 17O, 20I–J, 21H, 33B, 34A–F)

**Fig. 34:**
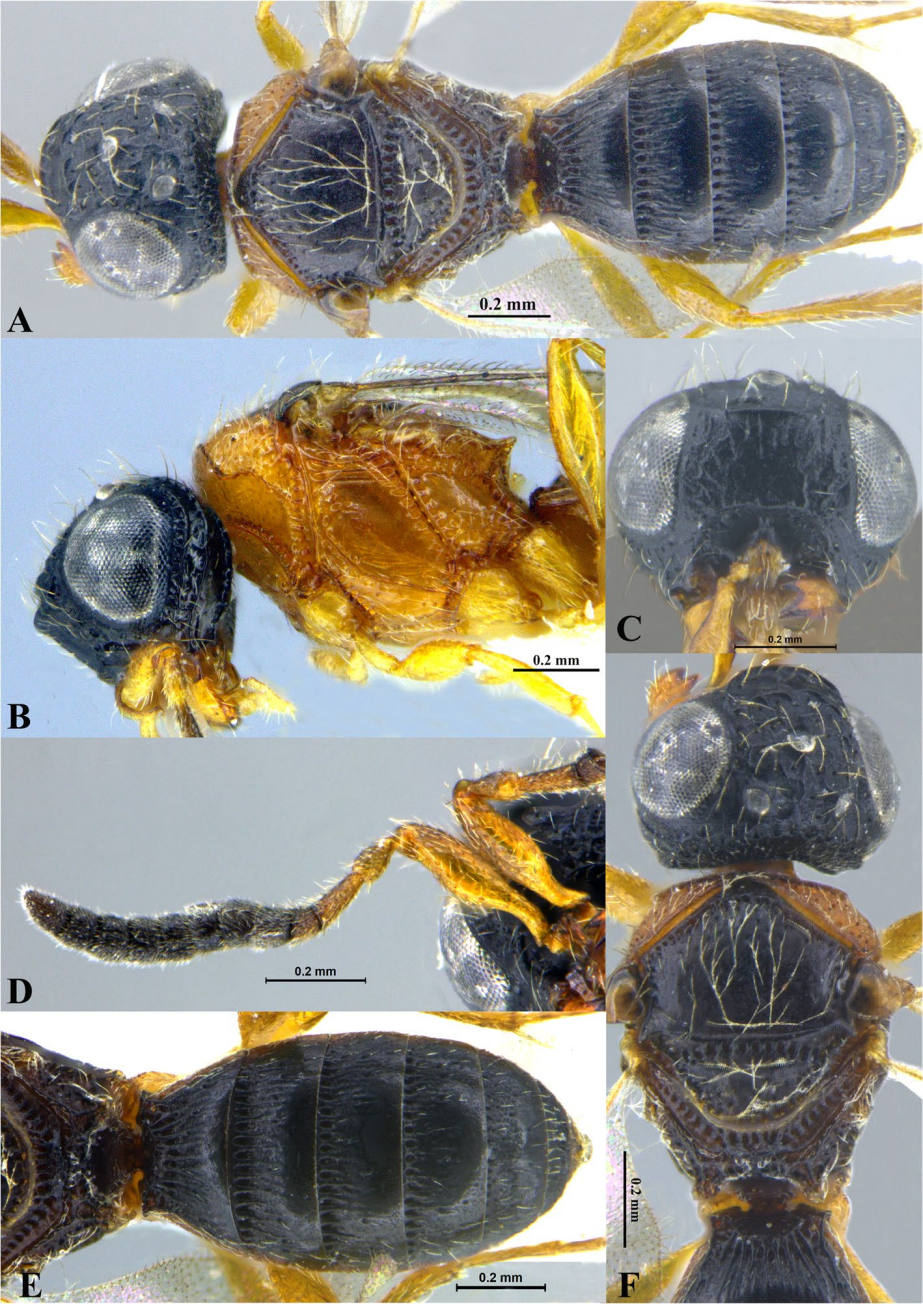
*Sparasion hindoli ***sp. n**., female holotype. **A** Habitus, dorsal view. **B** Head and pleuron. **C** Frons. **D** Antenna. **E** Metasoma. **F** Head and mesonotum

urn:lsid:zoobank.org:act:64053FF9-D93C-4122-955A-ED267CAD3606

#### Diagnosis

*Sparasion hindoli* sp. n. is close to *S. bhairavi* sp. n. The distinguishing characters are given above under the latter species.

#### Material examined

Holotype: Female, (ICAR/NBAIR/P4698), INDIA: Tamil Nadu: Nilgiris, Wellington, Indian Institute of Soil and Water Conservation (IISWC), 11°21′36ʺN 76°48′42ʺE, 1997 m, YPT, 18.XII.2016. Paratypes: 7 females (ICAR/NBAIR/P4699–P4705), Tamil Nadu: Nilgiris, Wellington, IISWC, 11°21′36ʺN 76°48′42ʺE, 1997 m, YPT, 18.XII.2016; 1 female (ICAR/NBAIR/P4706), Tamil Nadu: Lower Pulney Hills, Thadiyankudisai, HRS, 10°17′58ʺN 77°42′42ʺE, 990 m, YPT in banana plot, 28.XI.2016; 4 males (ICAR/NBAIR/P4707–P4710), Tamil Nadu: Nilgiris, Wellington, IISWC, 11°21′36ʺN 76°48′42ʺE, 1997 m, YPT, 18.XII.2016.

### Description

Female body length = 2.31–2.43 mm (*n* = 8); male body length = 2.24–2.33 mm (*n* = 4).

#### Colour

Head, mesonotum and metasoma black except for brown-black mesoscutellar rim and red-brown metanotal trough; dorsal and lateral pronotal area yellow–brown; meso and metapleuron honey-brown; transverse pronotal carina black-brown; tegula light brown with uneven black patches; legs yellow–brown; radicle, A1 yellow with uneven brown patches, A2–A3 yellow–brown, A4 brown, remaining antennomeres black; mandibles orange-brown with teeth dark brown.

#### Head

1.2 × as wide as high, 1.3 × as high as long. Setation on head: sparse. IOS: 0.6 × head width, subequal to eye length. POL > LOL > OOL: 13.2:10.4:4.6. OOL: 0.8 × MOD. Compound eye: (L: W = 31.7:27.9). Setation of compound eye: sparsely setose. Anterior margin of frons: arcuate. Distance from the level of anterior margin of compound eyes to anterior extension of frons in dorsal view: subequal to MOD. Number of transverse ledges on upper frons: three. Sculpture of upper frons: anteriorly smooth remainder with effaced polygonal cells bearing setae. Interantennal process: 1.6 × as long as wide, smooth. Sculpture of lower frons: medially smooth, laterally with sparse effaced polygonal cells and uneven longitudinal carinae dorsally. Interantennal process: 1.4 × as long as wide, smooth. Transverse carina above interantennal process: without medial notch. Area ventral to transverse carina above interantennal process: smooth, with setigerous foveae laterally. Sculpture on vertex: effaced polygonal cells followed by a transverse sinuous carina, posteriorly smooth with setigerous punctae. Sculpture of posterior orbital furrow: with rectangular cells. Genal carina: present. Sculpture of gena: anteriorly with shallow depressions, posteriorly smooth with sparse setae. Sculpture on A1: smooth with sparse setae. A1: 3.9 × as long as wide. Length of A3: 0.4 × A1 and 1.2 × A2.

#### Mesosoma

Sculpture of dorsal pronotum: smooth with sparse setigerous punctae. L: W of mesoscutum: 34.4:46.1. Sculpture of mesoscutum: smooth with setigerous punctae, punctae sparse posteriorly, setae long. Notaulus: absent. Mesoscutal humeral sulcus: foveate. Mesoscutal suprahumeral sulcus: foveate. Parapsidal line: indicated as a weak furrow. Scutoscutellar sulcus: foveate; foveae complete. L: W of mesoscutellum: 19.5:33.3. Sculpture of mesoscutellum: smooth with sparse setigerous foveae, setae long. Sculpture of dorsellum: anteriorly foveate, posterior margin almost straight. Sculpture of outer lateral propodeal area: punctate. Sculpture of inner lateral propodeal area: anteriorly sparsely setose, posteriorly smooth, a transverse medial carina present. Lateral propodeal carina: arched. Posterior propodeal projection: rounded, not extending to anterior margin of T1. Sculpture of metasomal depression: with foveae and sparse pilosity. Plical area: anteriorly densely setose, posteriorly smooth with sparse pilosity. Sculpture of propleuron: smooth. Sculpture of lateral pronotal area: smooth. Posterior pronotal sulcus: with oblong cells. Pronotal cervical sulcus: foveate. Speculum of mesopleuron: transversely carinate, sparsely setose. Postacetabular sulcus: foveate. Prespecular sulcus: foveate. Mesepimeral sulcus: foveate. Posterior mesepimeral area: smooth, narrower than mesepimeral sulcus. Mesopleural carina: percurrent, with a row of weak foveae anterodorsally. Sculpture of femoral depression: smooth. Mesopleural pit: present. Sculpture of ventral mesopleuron: anteriorly with a row of irregular depressions, remainder smooth with sparse setigerous punctae. Sculpture of metapleuron: dorsal metapleural area narrow and smooth with sparse setae on anterior margin; ventral metapleural area dorsally smooth and ventrally with shallow polygonal cells. Metapleural sulcus: foveate, indicated medially as furrow. Paracoxal sulcus: with shallow foveae. Metapleural epicoxal sulcus: with uneven depressions.

#### Fore wing

L: W: 181.1:89.4. Transparency: weakly infuscate. Lengths of R: R1: r-rs in ratio of 68:28:21. R: distal 2/3 curving towards anterior margin of wing. Anterior margin of fore wing: with no downcurve prior to R1.

#### Metasoma

L: W of metasoma: 98.7:54.0. Ratio of length of T1: T2: T3: T4: T5: 16.3:20.0:19.4:19.2:13.7. Anterior margin of T1: weakly convex. Sculpture of T1: basal foveae present, followed by longitudinal costae, laterally smooth with sparse setae and posteriorly weakly rugose. Sculpture of T2: basal foveae present, followed by short longitudinal costae; laterally smooth with sparse setae, posteriorly smooth with weak punctae. Sculpture of T3: same as T2, costae medially absent. Sculpture of T4: same as T2, smooth with weak punctae and longitudinal costae absent. Sculpture of T5: basal foveae present, anteriorly punctate and sparsely setose, posteriorly smooth. Sculpture of T6: smooth with sparse setigerous punctae.

#### Male

Similar to female except for the following characters: posterior vertex with dense setae, setigerous punctae on mesoscutum dense; mesoscutellum with denser setigerous foveae; longitudinal costae on metasomal tergites long and dense.

#### Etymology

This species is named ‘Hindol’ after one of the *ragas* or melodic structures, in North Indian (Hindustani) classical music associated with Spring and sung in the early hours of the day.

### *Sparasion kalyani* Veenakumari sp. n. (Figs. 21I, 33C, 35A–F, 36A)

**Fig. 35:**
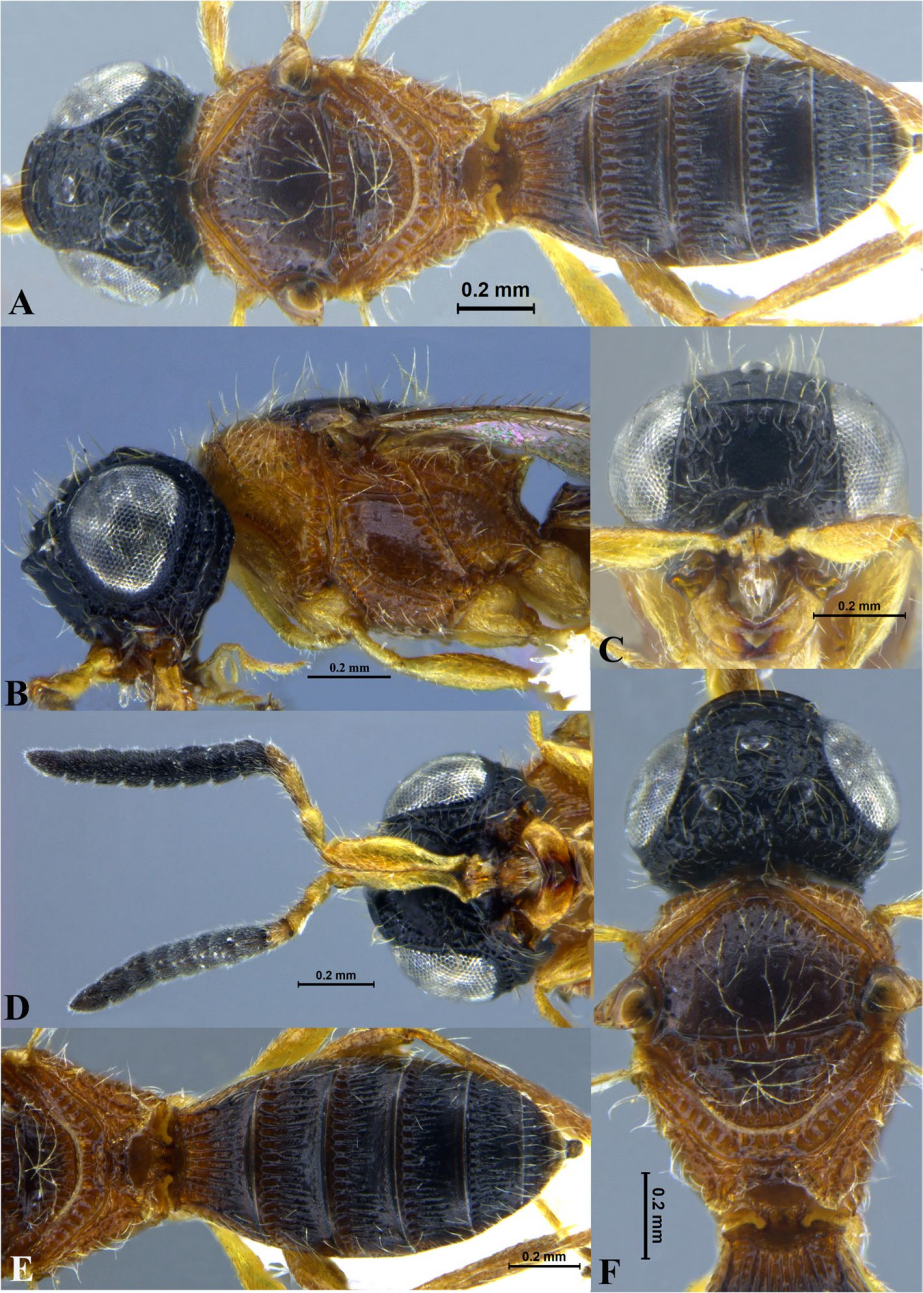
*Sparasion kalyani ***sp. n**., female holotype. **A** Habitus, dorsal view. **B** Head and pleuron. **C** Frons. **D** Antennae. **E** Metasoma. **F** Head and mesonotum

**Fig. 36:**
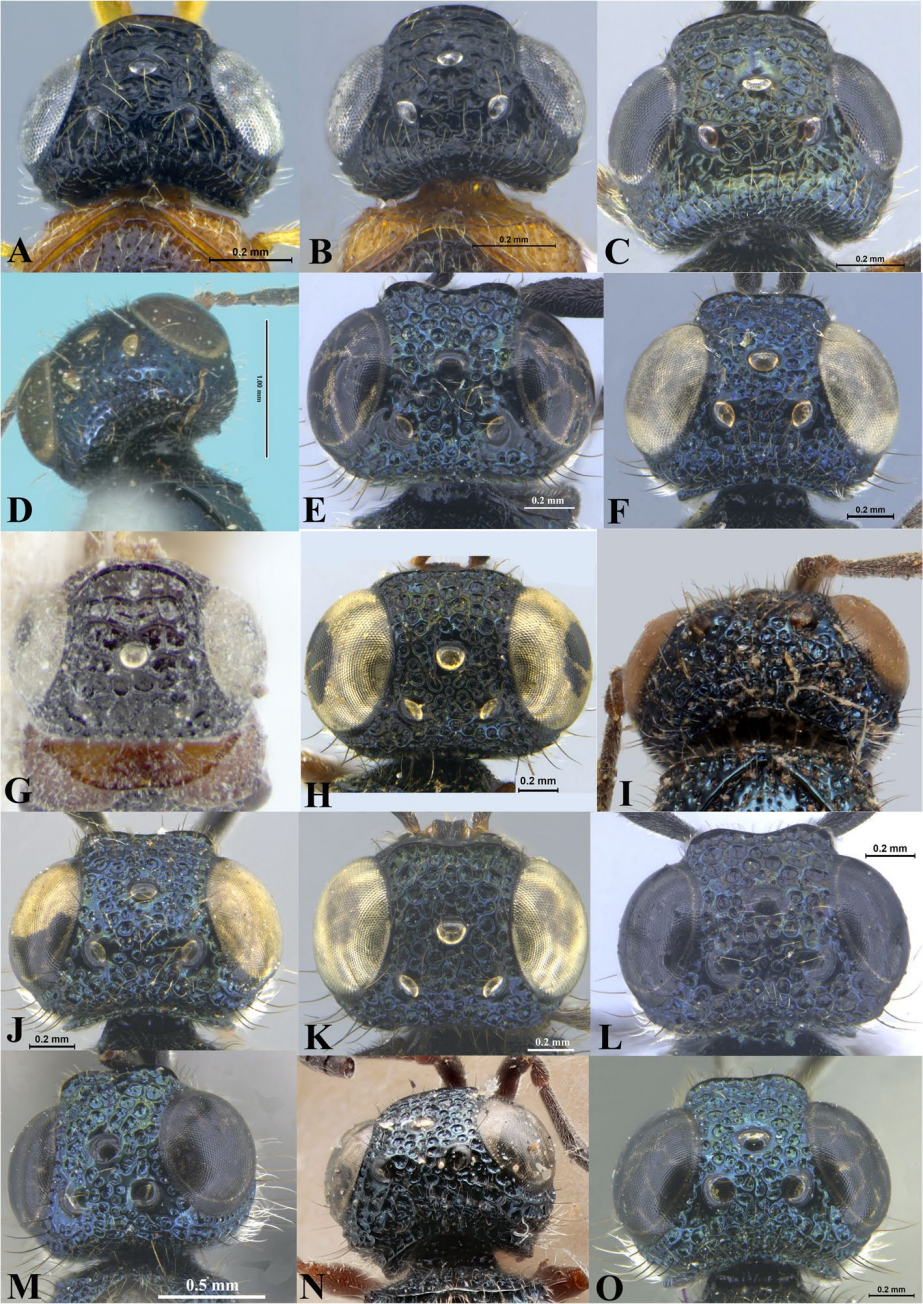
Head, dorsal view. **A*** Sparasion kalyani. ***B ***S. kanakangi*. **C ***S. karivadana*. **D ***S. lividus*. **E ***S. manavati*. **F ***S. meghmalhari*. **G ***S. micromerus. ***H ***S. pahadi*. **I ***S. philippinensis*. **J ***S. ratnangi. ***K ***S. rupavati. ***L ***S. salagami. ***M ***S. shulini. ***N ***S. sinensis*. **O ***S. sivaranjini*

urn:lsid:zoobank.org:act:A5FAD083-34B8-47E3-A29F-75A75AF4160D

#### Diagnosis

*Sparasion kalyani* sp. n. is close to *S. kanakangi* sp. n. but differs from it in the following characters: in *S. kalyani* sp. n. lateral propodeal area is sparsely foveate and posterior lateral propodeal area present as a wide lamella. Conversely, in *S. kanakangi* sp. n. lateral propodeal area is densely punctate-foveate and posterior lateral propodeal area is narrow and notched.

#### Material examined

Holotype: Female, (ICAR/NBAIR/P4673), **INDIA**: Tamil Nadu: Yelagiri, Thayalur, 12°34′43ʺN 78°39′46ʺE, 1111 m, YPT, 15.VI.2016. Paratype: 1 female (ICAR/NBAIR/P4674), Tamil Nadu: Lower Pulney Hills, Thadiyankudisai, HRS, 10°17′58ʺN 77°42′42ʺE, 990 m, YPT in bay leaf (*Laurus nobilis*: Lauraceae) plot, 28.XI.2016.

### Description

Female body length = 2.49–2.61 mm (*n* = 2).

#### Colour

Head black; mesoscutum brown-black; mesoscutellum brown-black except brown posterior mesoscutellar sulcus; pronotum, dorsellum, metanotal trough, lateral propodeal area yellow-brown; transverse pronotal carina black-brown; anterior T1 reddish-brown, remainder of metasoma black-brown; lateral pronotal area yellow-brown, mesopleuron and metapleuron honey-brown; tegula light brown with uneven black patches; legs yellow-brown; radicle, A1–A2 yellow, A3–A4 brown, remaining antennomeres black; mandibles yellow with teeth dark brown.

#### Head

1.2 × as wide as high, 1.3 × as high as long. Setation on head: sparse. IOS: 0.5 × head width, subequal eye length. POL > LOL > OOL: 16.6:11.5:5.1. OOL: 0.7 × MOD. Compound eye: (L: W = 33.6:30.5). Setation of compound eye: glabrous. Anterior margin of frons: arcuate. Distance from the level of anterior margin of compound eyes to anterior extension of frons in dorsal view: 1.2 × MOD. Number of transverse ledges on upper frons: three. Sculpture of upper frons: anteriorly smooth, followed by a row of effaced polygonal cells and a row of polygonal cells in front of anterior ocellus. Sculpture of lower frons: smooth with one row dorsally and two rows laterally of polygonal cells bearing setae. Interantennal process: 1.4 × as long as wide, smooth with medial furrow. Transverse carina above interantennal process: without medial notch. Area ventral to transverse carina above interantennal process: smooth, with setigerous foveae laterally. Sculpture on vertex: anteriorly with large polygonal cells, followed by smaller polygonal cells bearing setae, followed by a uneven transverse carina, posteriorly with smooth area with setigerous punctae; anterior ocellus without smooth area around; lateral ocellus with irregular smooth area posteriorly. Sculpture of posterior orbital furrow: foveate. Genal carina: present. Sculpture of gena: anterodorsally with depressions and anteroventrally foveate, posteriorly smooth, sparsely setose. Sculpture on A1: smooth with sparse setae. A1: 4 × as long as wide. Length of A3: 0.3 × A1 and 1.1 × A2.

#### Mesosoma

Sculpture of dorsal pronotum: setigerous punctate. L: W of mesoscutum: 37.5:51.9. Sculpture of mesoscutum: weakly reticulate with setigerous punctae, punctae sparse posteriorly; setae long, a short ligula present anteromedially. Notaulus: absent. Mesoscutal humeral sulcus: foveate. Mesoscutal suprahumeral sulcus: with circular cells. Parapsidal line: indicated as furrow. Scutoscutellar sulcus: foveate. L: W of mesoscutellum: 23.1:34.3. Sculpture of mesoscutellum: weakly reticulate with sparse setigerous foveae, setae long. Sculpture of dorsellum: anteriorly foveate, posteriorly with shallow impressions of foveae, posterior margin weakly sinuous. Sculpture of outer lateral propodeal area: with depressions and sparse long setae. Sculpture of inner lateral propodeal area: anteriorly sparsely setose followed by a transverse median carina; posteriorly smooth. Lateral propodeal carina: weakly sinuous. Posterior propodeal projection: rounded, not extending to anterior margin of T1. Sculpture of metasomal depression: with depressions bearing sparse setae along with a medial longitudinal carina. Plical area: anteriorly densely setose, posteriorly weakly rugose with sparse setae. Sculpture of propleuron: weakly rugose. Sculpture of lateral pronotal area: weakly rugose interspersed with uneven striae. Posterior pronotal sulcus: with irregular cells. Pronotal cervical sulcus: foveate. Speculum of mesopleuron: sparsely transversely carinate with dense setae. Postacetabular sulcus: foveate. Prespecular sulcus: foveate. Mesepimeral sulcus: foveate. Posterior mesepimeral area: smooth, narrower than mesepimeral sulcus. Mesopleural carina: percurrent, with a row of shallow foveae dorsally. Sculpture of femoral depression: smooth. Mesopleural pit: present. Sculpture of ventral mesopleuron: with a transverse row of setigerous foveae dorsally, remainder smooth with setigerous punctae. Sculpture of metapleuron: dorsal metapleural area narrow with sparse setae on anterior margin; ventral metapleural area dorsally weakly rugose and ventrally with two transverse rows of rectangular cells. Metapleural sulcus: foveate, medially indicated as a furrow. Paracoxal sulcus: foveate. Metapleural epicoxal sulcus: with shallow foveae.

#### Fore wing

L: W: 180.4:80.4. Transparency: weakly infuscate. Lengths of R: R1: r-rs in ratio of 80:25:21. R: basally closer and gradually diverging from anterior margin of wing. Anterior margin of fore wing: upcurved basally and with no downcurve prior to R1.

#### Metasoma

L: W of metasoma: 112.3:57.0. Ratio of length of T1: T2: T3: T4: T5: 19.2:20.3:20.0:19.7:16.4. Anterior margin of T1: weakly convex. Sculpture of T1: longitudinally costate, laterally smooth with setigerous foveae and punctae, posteriorly weakly rugose. Sculpture of T2: basal foveae present, followed by longitudinal costae, space between costae with intricate sculpture; laterally smooth with setigerous punctae, posteriorly weakly rugose. Sculpture of T3: same as T2, with shorter costae medially. Sculpture of T4: same as T3, with shorter costae medially. Sculpture of T5: basal foveae present, followed by setigerous punctae interspersed with short costae on anterior margin, posteriorly smooth to weakly punctate. Sculpture of T6: smooth with setigerous punctae.

#### Male

Unknown.

#### Etymology

This species is named ‘Kalyani’ after a *ragam* or melodic structure in South Indian (Carnatic) classical music meaning ‘the lady who is the harbinger of the auspicious’; performed often at South Indian weddings.

### *Sparasion kanakangi* Veenakumari sp. n. (Figs. 20K–L, 21J, 33D, 36B, 37A–F)

**Fig. 37:**
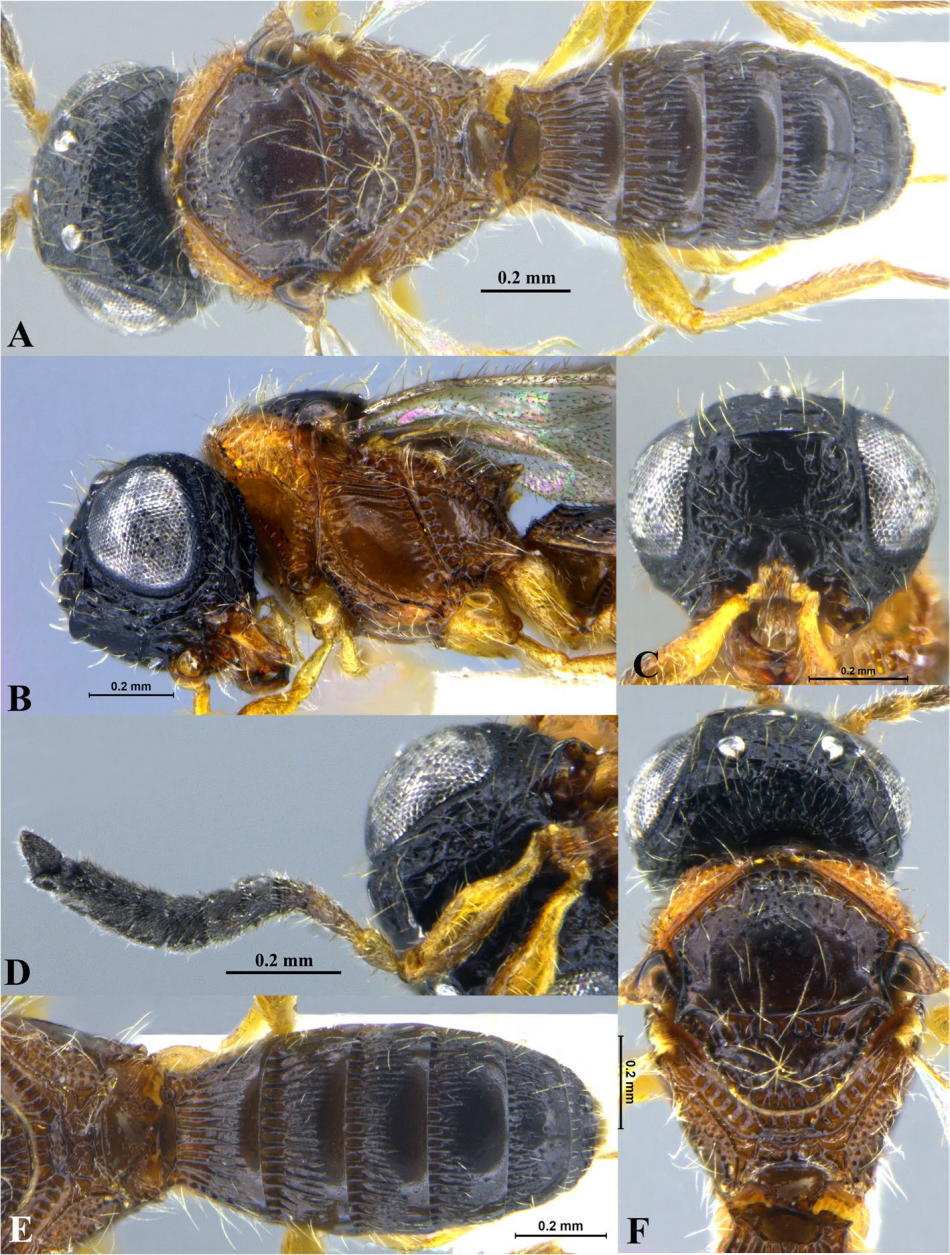
*Sparasion kanakangi ***sp. n**., female holotype. **A** Habitus, dorsal view. **B** Head and pleuron. **C** Frons. **D** Antenna. **E** Metasoma. **F** Head and mesonotum

urn:lsid:zoobank.org:act:059C729D-D44C-4747-8F39-9838D3921329

#### Diagnosis

*Sparasion kanakangi* sp. n. is close to *S. kalyani*. The distinguishing characters are given above under the latter species.

#### Material examined

Holotype: Female, (ICAR/NBAIR/P4689), **INDIA**: Tamil Nadu: Lower Pulney Hills, Thadiyankudisai, HRS, 10°17′58ʺN 77°42′42ʺE, 990 m, YPT, 25.XI.2016. Paratypes:1 female (ICAR/NBAIR/P4690), Tamil Nadu: Lower Pulney Hills, Thadiyankudisai, HRS, 10°17′58ʺN 77°42′42ʺE, 990 m, YPT, 07.XI.2014; 1 male (ICAR/NBAIR/P4691), Tamil Nadu: Lower Pulney Hills, Thadiyankudisai, HRS, 10°17′58ʺN 77°42′42ʺE, 990 m, YPT, 25.XI.2016; 1 male (ICAR/NBAIR/P4692), Tamil Nadu: Lower Pulney Hills, Thadiyankudisai, HRS, 10°17′58ʺN 77°42′42ʺE, 990 m, YPT, 26.VI.2015; 5 males (ICAR/NBAIR/P4693–P4697), Tamil Nadu: Dindugul, Thandikudi, RCRS, 10°18′34ʺN 77°38′34ʺE, 1305 m, YPT in black pepper (*Piper nigrum*: Piperaceae) plot, 27.XI.2016.

### Description

Female body length = 2.33–2.41 mm (*n* = 2); male body length = 2.49–2.61 mm (*n* = 7).

#### Colour

Head black; mesoscutum, mesoscutellum brown-black; dorsellum, metanotal trough, lateral propodeal area, meso and metapleuron honey-brown; dorsal and lateral pronotum yellow-brown; transverse pronotal carina black-brown; T1–T2 brown-black, remaining tergites black; tegula light brown with uneven black patches; legs yellow-brown; radicle, A1–A2 yellow with uneven black patches, A3 brown, remaining antennomeres black; mandibles yellow with teeth dark brown.

#### Head

1.2 × as wide as high, 1.2 × as high as long. Setation on head: sparse. IOS: 0.5 × head width, subequal to eye length. POL > LOL > OOL: 16.7:11.6:5.1. OOL: 0.8 × MOD. Compound eye: (L: W = 33.1:29.3). Setation of compound eye: sparsely setose. Anterior margin of frons: arcuate. Distance from the level of anterior margin of compound eyes to anterior extension of frons in dorsal view: 1.3 × MOD. Number of transverse ledges on upper frons: three. Sculpture of upper frons: anteriorly smooth, followed by a row of effaced polygonal cells and a row of polygonal cells in front of anterior ocellus. Sculpture of lower frons: medially smooth with sparse incomplete polygonal cells dorsally and with uneven longitudinal carinae and polygonal cells laterad. Interantennal process: 1.2 × as long as wide, smooth. Transverse carina above interantennal process: without medial notch. Area ventral to transverse carina above interantennal process: smooth, with setigerous foveae laterally. Sculpture on vertex: anteriorly with large polygonal cells, followed by smaller polygonal cells medially and an uneven transverse carina, posteriorly and laterally smooth with setigerous punctae. Sculpture of posterior orbital furrow: foveate. Genal carina: present. Sculpture of gena: smooth with sparse setae except for shallow polygonal cells anteroventrally, sparsely setose. Sculpture on A1: smooth with sparse setae. A1: 4.2 × as long as wide. Length of A3: 0.4 × A1 and 1.4 × A2.

#### Mesosoma

Sculpture of dorsal pronotum: with setigerous pit and depressions. L: W of mesoscutum: 31.4:48.6. Sculpture of mesoscutum: smooth with setigerous punctae, punctae sparse posteriorly, setae long. Notaulus: absent. Mesoscutal humeral sulcus: foveate. Mesoscutal suprahumeral sulcus: with circular and uneven cells. Parapsidal line: indicated as furrow. Scutoscutellar sulcus: foveate. L: W of mesoscutellum: 21.8:32.9. Sculpture of mesoscutellum: smooth with sparse setigerous foveae, setae long and with a discontinuous furrow anteriorly. Sculpture of dorsellum: anteriorly foveate, medially with a transverse carina, posteriorly foveate, posterior margin sinuous. Sculpture of outer lateral propodeal area: densely foveate, sparsely setose. Sculpture of inner lateral propodeal area: anteriorly sparsely setose, posteriorly smooth. Lateral propodeal carina: sinuous. Posterior propodeal projection: rounded, not extending to anterior margin of T1. Sculpture of metasomal depression: smooth with a medial vertical carina and sparse pilosity. Plical area: anteriorly densely setose, posteriorly smooth with setigerous punctae. Sculpture of propleuron: smooth. Sculpture of lateral pronotal area: smooth. Posterior pronotal sulcus: with oblong cells. Pronotal cervical sulcus: foveate. Speculum of mesopleuron: transversely carinate with sparse setae. Postacetabular sulcus: foveate. Prespecular sulcus: foveate. Mesepimeral sulcus: foveate. Posterior mesepimeral area: smooth, narrower than mesepimeral sulcus. Mesopleural carina: percurrent, with a row of uneven depressions dorsally. Sculpture of femoral depression: smooth. Mesopleural pit: present. Sculpture of ventral mesopleuron: anteriorly with a row of rectangular cells with setae and remainder smooth with sparse setigerous punctae. Sculpture of metapleuron: dorsal metapleural area narrow and smooth with sparse setae on anterior margin; ventral metapleural area dorsally smooth and ventrally with sparse foveae and polygonal cells. Metapleural sulcus: with elongate depressions, medially indicated as a furrow. Paracoxal sulcus: with oblong cells. Metapleural epicoxal sulcus: with depressions.

#### Fore wing

L: W: 187.3:78.0. Transparency: weakly infuscate. Lengths of R: R1: r-rs in ratio of 77:24:19. R: gradually distant from anterior margin of wing. Anterior margin of fore wing: with no downcurve prior to R1.

#### Metasoma

L: W of metasoma: 95.2:46.7. Ratio of length of T1: T2: T3: T4: T5: 17.9:18.8:18.4:17.5:13.9. Anterior margin of T1: weakly convex. Sculpture of T1: with elongate basal foveae followed by longitudinal costae (with intricate sculpture between costae), posteriorly smooth, laterally smooth with dense setae. Sculpture of T2: basal foveae present, followed by longitudinal costae extending 0.7 × the length of tergite, remainder smooth; laterally smooth with setae. Sculpture of T3: same as T2, except for shorter costae medially. Sculpture of T4: same as T2, with sparse shorter costae medially. Sculpture of T5: basal foveae present, with short costae sublaterally on anterior margin, remainder smooth with sparse setigerous punctae. Sculpture of T6: smooth with setigerous punctae.

#### Male

Similar to female except for the following characters: setigerous punctae on mesoscutum dense; mesoscutellum with polygonal cells posteriorly.

#### Etymology

This species is named ‘Kanakangi’ after a *ragam* or melodic structure in South Indian (Carnatic) classical music which means ‘the golden bodied one’.

### *Sparasion karivadana* Veenakumari sp. n. (Figs. 21K, 33E, 36C, 38A–F, 39A–C)

**Fig. 38:**
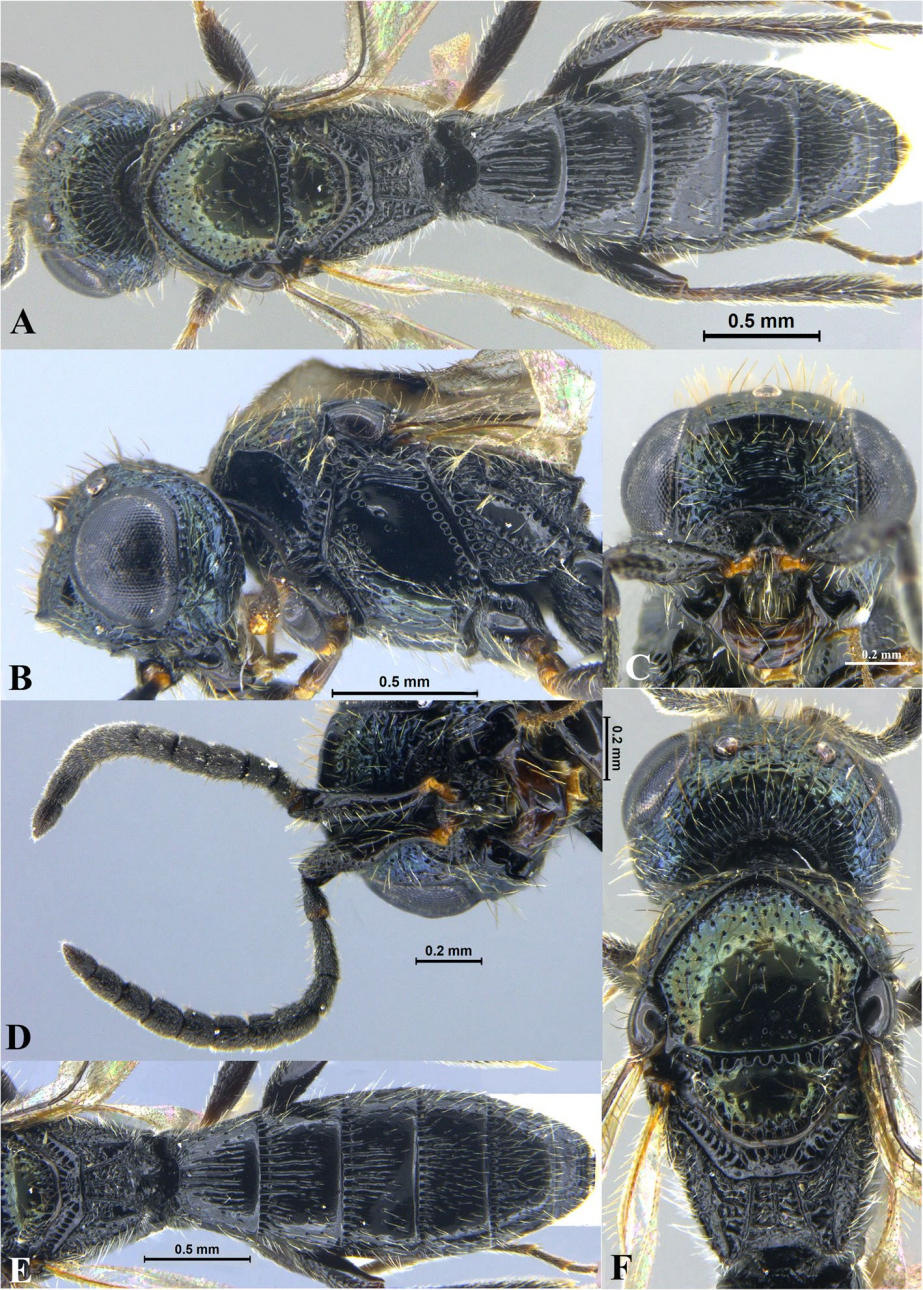
*Sparasion karivadana ***sp. n**., female holotype. **A** Habitus, dorsal view. **B** Head and pleuron. **C** Frons. **D** Antennae. **E** Metasoma. **F** Head and mesonotum

**Fig. 39:**
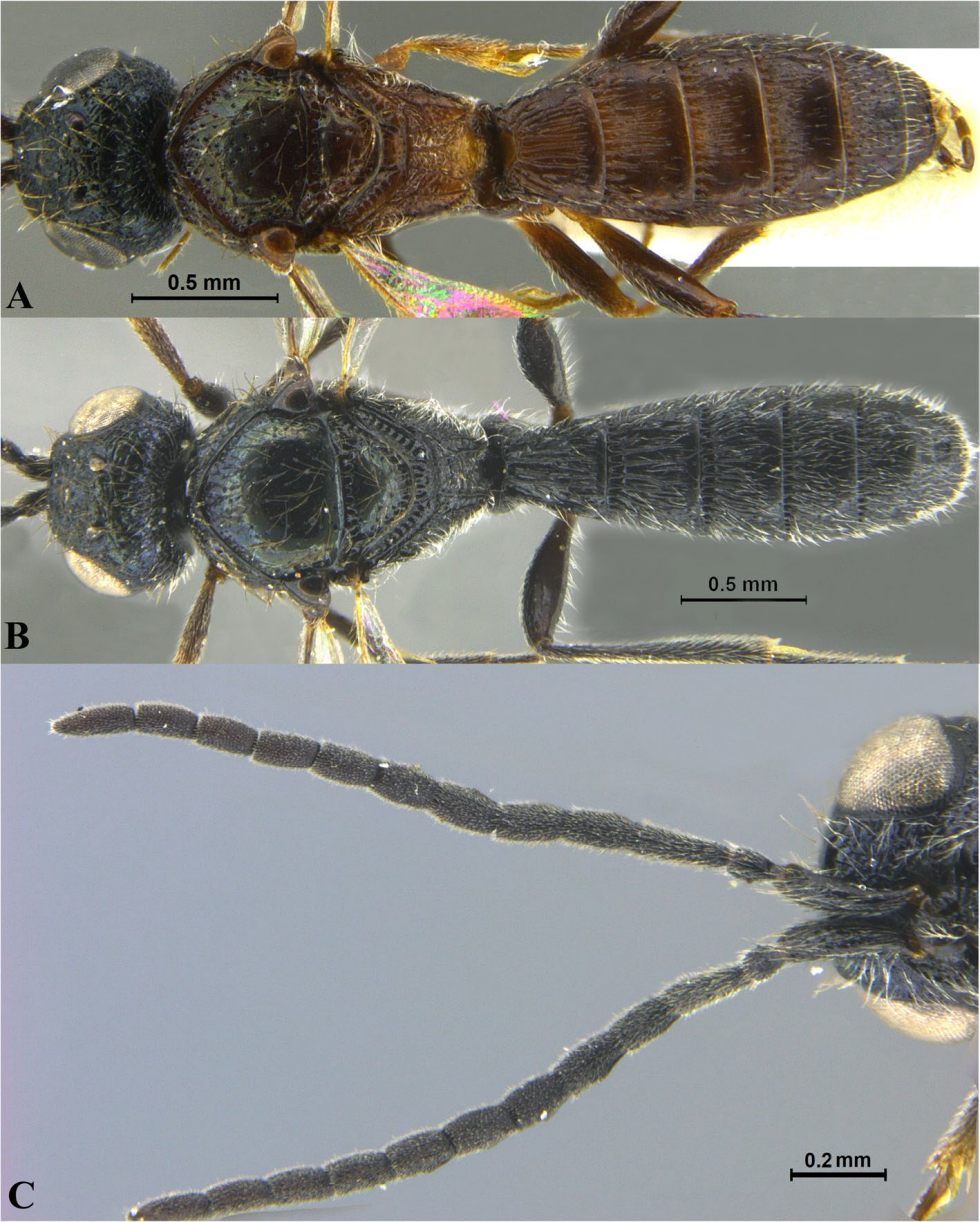
*Sparasion karivadana ***sp. n**., female and male paratypes. **A** Habitus, dorsal view of colour morph of female. **B** Habitus, dorsal view of male. **C** Male antennae

urn:lsid:zoobank.org:act:109E5E8C-FACE-496A-9A4E-4FBDF6F33F70

#### Diagnosis

Though *Sparasion karivadana* sp. n. has metallic blue-green head and mesonotum (green-black head and green mesoscutum in colour morph), it is distinct from those in *Sparasion manavati* species group in having a smooth A1 with sparse foveae and an upcurved radialis.

#### Material examined

Holotype: Female, (ICAR/NBAIR/P4583), **INDIA**: Himachal Pradesh: Dalhousie, 32°32′19ʺN 75°58′15ʺE, 2021 m, YPT, 24.VIII.2014. Paratypes: 2 females, (ICAR/NBAIR/P4584–P4585), Karnataka: Mandya, 12°33′51ʺN 76°44′01ʺE, 749 m, YPT, in finger millet (*Eleusine coracana*: Poaceae) field, 01.X.2012; 2 males (ICAR/NBAIR/P4586–P4587), Himachal Pradesh: Dhundi, 32°21′10ʺN 77°07′47ʺE, 2857 m, SN, 06.VIII.2014.

### Description

Female body length = 4.41–4.62 mm, (*n* = 3); male body length = 3.81–4.02 mm (*n* = 2).

#### Colour

Head, mesoscutum and mesoscutellum steel green; dorsellum, metanotal trough, lateral propodeal area, metasomal depression, pleuron, tegula and metasoma black; legs entirely black-brown except brown trochanter; radicle and basal A1 yellow–brown, remaining antennomeres black; mandibles red-brown with teeth dark brown. Brown colour morphs of males have been observed.

#### Head

1.3 × as wide as high, as high as long. Setation on head: dense. IOS: 0.6 × head width, subequal to eye length. POL > LOL > OOL: 24.9:15.1:7.1. OOL: 0.9 × MOD. Compound eye: (L: W = 48.5:45.6). Setation of compound eye: with short setae. Anterior margin of frons: arcuate. Distance from the level of anterior margin of compound eyes to anterior extension of frons in dorsal view: 1.6 × MOD. Number of transverse ledges on upper frons: one. Sculpture of upper frons: with polygonal cells bearing setae. Sculpture of lower frons: dorsally and laterally with polygonal cells bearing setae, medially smooth with sparse transverse carinae. Interantennal process: subequal in length and width, smooth. Transverse carina above interantennal process: without acute medial notch. Area ventral to transverse carina above interantennal process: setigerous punctate. Sculpture on vertex: anteriorly with large polygonal cells bearing setae, followed by an uneven transverse carina medially, posteriorly smooth with setigerous punctae; smooth area present anterior to anterior ocellus; a smooth triangular area present posterior to lateral ocellus. Sculpture of posterior orbital furrow: dorsally with depressions and ventrally foveate. Genal carina: absent. Sculpture of gena: entirely setigerous foveate interspersed with longitudinal carinae except for smooth area posteriorly and ventrally, sparsely setose. Sculpture on A1: sparsely setigerous foveate. A1: 3.7 × as long as wide. Length of A3: 0.4 × A1 and 1.2 × A2.

#### Mesosoma

Sculpture of dorsal pronotum: smooth with setigerous punctae. L: W of mesoscutum: 57.9:69.2. Sculpture of mesoscutum: smooth with sparse setigerous punctae. Notaulus: absent. Mesoscutal humeral sulcus: foveate. Mesoscutal suprahumeral sulcus: foveate. Parapsidal line: indicated as furrow. Scutoscutellar sulcus: foveate. L: W of mesoscutellum: 30.3:42.1. Sculpture of mesoscutellum: smooth with sparse setigerous foveae. Sculpture of dorsellum: anteriorly foveate, posteriorly smooth with sparse punctae, posterior margin straight. Sculpture of outer lateral propodeal area: with depressions, sparsely setose. Sculpture of inner lateral propodeal area: anteriorly setose and posteriorly with depressions between carinae. Lateral propodeal carina: oblique and almost straight. Posterior propodeal projection: pointed, not extending to anterior margin of T1. Sculpture of metasomal depression: entirely sparsely setose with several transverse carinae on either side of medial longitudinal carina, except for a smooth posteromedial area. Plical area: with uneven depressions and sparse setae. Sculpture of propleuron: smooth with a weak median transverse furrow anteriorly. Sculpture of lateral pronotal area: smooth. Posterior pronotal sulcus: with ovoid cells. Pronotal cervical sulcus: weakly foveate. Speculum of mesopleuron: transversely carinate, sparsely setose. Postacetabular sulcus: not foveate. Prespecular sulcus: foveate. Mesepimeral sulcus: foveate. Posterior mesepimeral area: smooth, narrower than mesepimeral sulcus. Mesopleural carina: indicated as a short carina anteriorly with foveae on dorsal margin. Sculpture of femoral depression: smooth. Mesopleural pit: present. Sculpture of ventral mesopleuron: anteriorly with polygonal cells bearing dense setae, remainder smooth with sparse setigerous punctae. Sculpture of metapleuron: dorsal metapleural area smooth, densely setose on anterior margin; ventral metapleural area dorsally smooth and ventrally with polygonal cells bearing sparse white setae. Metapleural sulcus: foveate, medially indicated as a furrow, posteriorly indicated as irregular cells. Paracoxal sulcus: irregular cells. Metapleural epicoxal sulcus: with polygonal cells and sparse setae.

#### Fore wing

L: W: 261.1:125.0. Transparency: strongly infuscate. Lengths of R: R1: r-rs in ratio of 113:29:29. R: closer to anterior margin of wing the entire length. Anterior margin of wing: with a weak downcurve prior to R1.

#### Metasoma

L: W of metasoma: 197.9:82.3 Ratio of length of T1: T2: T3: T4: T5: 38.5:38.5:35.4:33.3:33.3. Anterior margin of T1: convex. Sculpture of T1: medially with longitudinally ribbed costae, laterally smooth with setigerous punctae, posteriorly smooth. Sculpture of T2: basal foveae present, medially with longitudinally ribbed costae; laterally smooth with sparse setigerous punctae, posteriorly smooth. Sculpture of T3: same as T2. Sculpture of T4: basal foveae present, entirely smooth with setigerous punctae, setae short and dense medially whereas setae long and sparse laterally. Sculpture of T5: same as T4, medial setae longer than those on T4. Sculpture of T6: weakly rugose with sparse setigerous punctae.

#### Male

Same as female.

#### Colour morph

Head black; mesoscutum green with red-brown tinge; mesoscutellum, metascutellum, lateral propodeal area, metasoma and legs red-brown.

#### Etymology

This species is named ‘karivadana’, one of the many names of the elephant-headed Hindu God Ganesha.

### *Sparasion lividus* Johnson, Masner, Musetti, 2008 (Figs. 19, 33F, 36D, 40A–F)

**Fig. 40:**
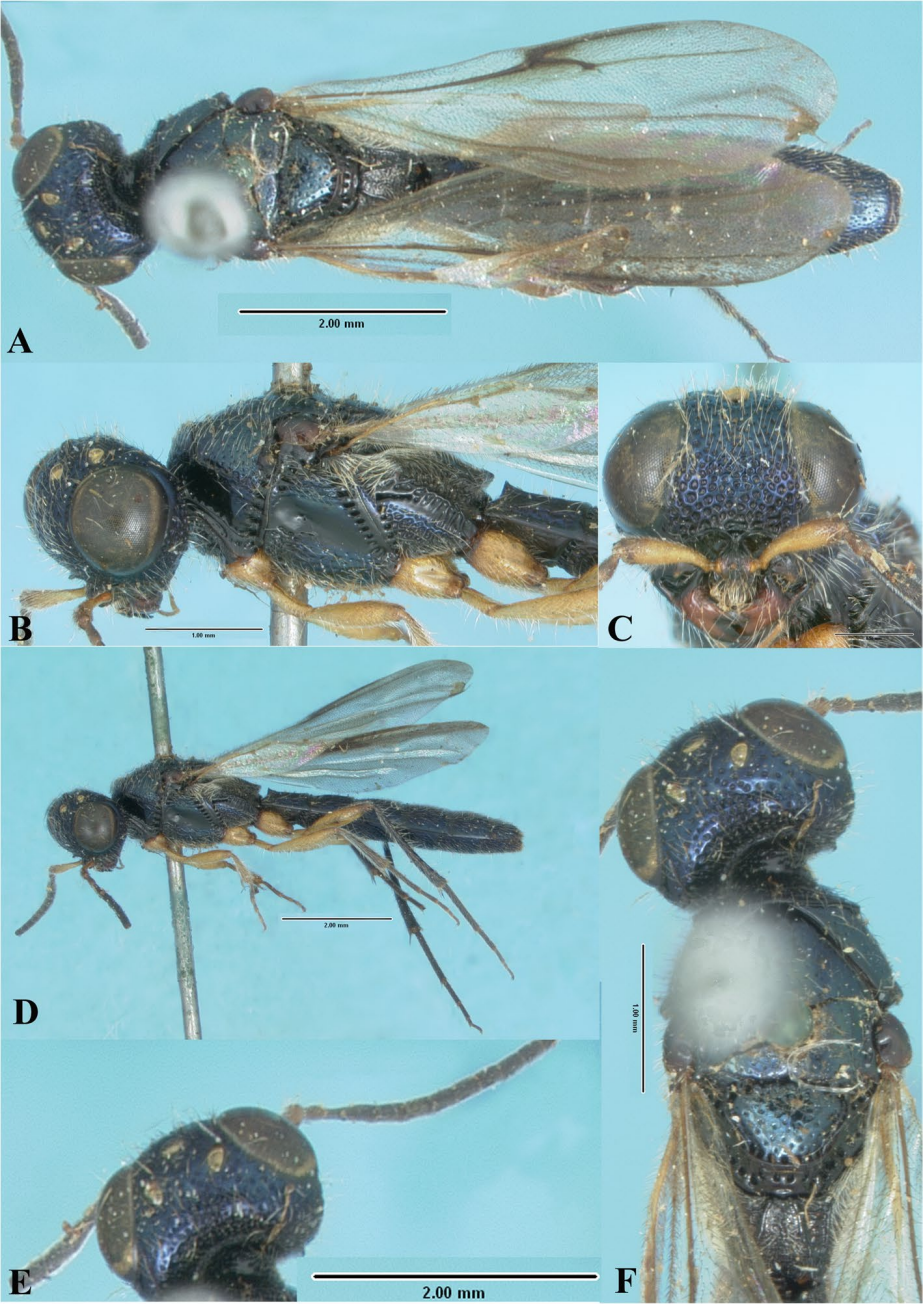
*Sparasion lividus* Johnson, Masner & Musetti, male holotype. **A** Habitus, dorsal view. **B** Head and pleuron. **C** Frons. **D** Habitus, lateral view. **E** Vertex and antenna. **F** Head and mesonotum (Photos: Dr. N. F. Johnson © Ohio State University)

*Prosparasion coeruleum* Kieffer, 1913: 190.

*Prosparasion coeruleum*: Kieffer, 1926: 299. Description.

*Prosparasion coeruleum*: Fouts, 1930: 6. Variation.

*Prosparasion coeruleum*: Kelner-Pillault, 1958: 151. Type information, error.

*Sparasion coeruleum*: Masner, 1976: 14. Generic transfer, description.

*Sparasion lividus*: Johnson, Masner, Musetti, 2008: 22. nom. nov.

#### Diagnosis

This is the only species in the genus *Sparasion* without the transverse ledge on the frons.

#### Material examined

Holotype, male, **PHILIPPINES**: Laguna, Los Baños, leg. M. Baker (cf. [4]).

### Description

Body length (male and female) = 6–9 mm.

#### Colour

Head, mesosoma and metasoma steel blue; tegula brown; all legs yellow-brown, except black-brown tarsi of all legs and tibiae of mid- and hind- legs; radicle, A1 yellow–brown, remaining antennomeres black-brown; mandible red-brown with margins of teeth black.

#### Head

Setation on head: dense. Setation of compound eye: glabrous. Number of transverse ledges on upper frons: None. Sculpture of upper frons: setigerous foveate with smooth interstices. Sculpture of lower frons: with dense circular setigerous polygonal cells. Transverse carina above interantennal process: with an acute medial notch. Area ventral to transverse carina above interantennal process: predominantly smooth. Sculpture on vertex: anteriorly smooth with sparse setigerous foveae, posteriorly with dense setigerous foveae. Sculpture of posterior orbital furrow: dorsally smooth, ventrally foveate. Genal carina: absent. Sculpture of gena: smooth with sparse foveae and setae. Sculpture on A1: densely setigerous punctate.

#### Mesosoma

Sculpture of dorsal pronotum: smooth with very sparse setigerous punctae. L: W of mesoscutum: 92:98. Sculpture of mesoscutum: smooth with sparse setigerous punctae; Notaulus: absent. Mesoscutal humeral sulcus: foveate. Mesoscutal suprahumeral sulcus: not foveate. Parapsidal line: indicated as furrow. Scutoscutellar sulcus: not foveate. L: W of mesoscutellum: 45:62. Sculpture of mesoscutellum: smooth with sparse setigerous and without an incomplete transverse furrow anteriorly. Sculpture of dorsellum: anteriorly foveate, posteriorly smooth, posterior margin with a medial upcurve. Sculpture of outer lateral propodeal area: densely setose. Sculpture of inner lateral propodeal area: smooth to weakly rugose, with medial transverse carina and dense anterior pilosity. Lateral propodeal carina: sinuous. Posterior propodeal projection: rounded, not extending on to anterior margin of T1. Sculpture of metasomal depression: rugose with setae. Plical area: anteriorly densely setose posteriorly with dense small depressions. Sculpture of propleuron: anteriorly indicated as a furrow and posteriorly with shallow foveae. Sculpture of lateral pronotal area: smooth. Posterior pronotal sulcus: with wide depressions. Pronotal cervical sulcus: weakly foveate. Speculum of mesopleuron: transversely carinate, interspersed with punctae, densely setose. Postacetabular sulcus: foveate. Prespecular sulcus: with wide depressions. Mesepimeral sulcus: foveate. Mesepimeral area: smooth, narrower than mesepimeral sulcus. Mesopleural carina: indicated anteriorly with foveae on dorsal margin. Sculpture of femoral depression: smooth. Mesopleural pit: present. Sculpture of ventral mesopleuron: anteriorly with polygonal cells, remainder smooth with sparse foveae, densely setose. Sculpture of metapleuron: dorsal metapleural area smooth with dense long setae on anterior margin; ventral metapleural area entirely with polygonal cells bearing white setae except for a narrow smooth patch posterodorsally. Metapleural sulcus: foveate, medially indicated as a furrow. Paracoxal sulcus: foveate. Metapleural epicoxal sulcus: foveate, densely setose.

#### Fore wing

L: W: 308.3:114. Transparency: weakly infuscate. Lengths of R: R1: r-rs in ratio of 143:88:37. R: distant from anterior margin of wing its entire length. Anterior margin of wing: downcurved basally and prior to R1.

#### Metasoma

L: W of metasoma: 252:71. Anterior margin of T1: weakly convex with a short medial spine. Sculpture of T1–T4: longitudinally costate. Sculpture of T5: longitudinally costate in anterior half sublaterally, remainder smooth with setigerous punctae; Sculpture of T6: smooth with setigerous punctae.

#### Female

Described in Kieffer [11, 23]. Not examined in this study.

#### Remarks

Holotype present at USNM. The specimen is in good condition.

### *Sparasion manavati* Veenakumari sp. n. (Figs. 21L, 33G, 36E, 41A–F, 42A)

**Fig. 41:**
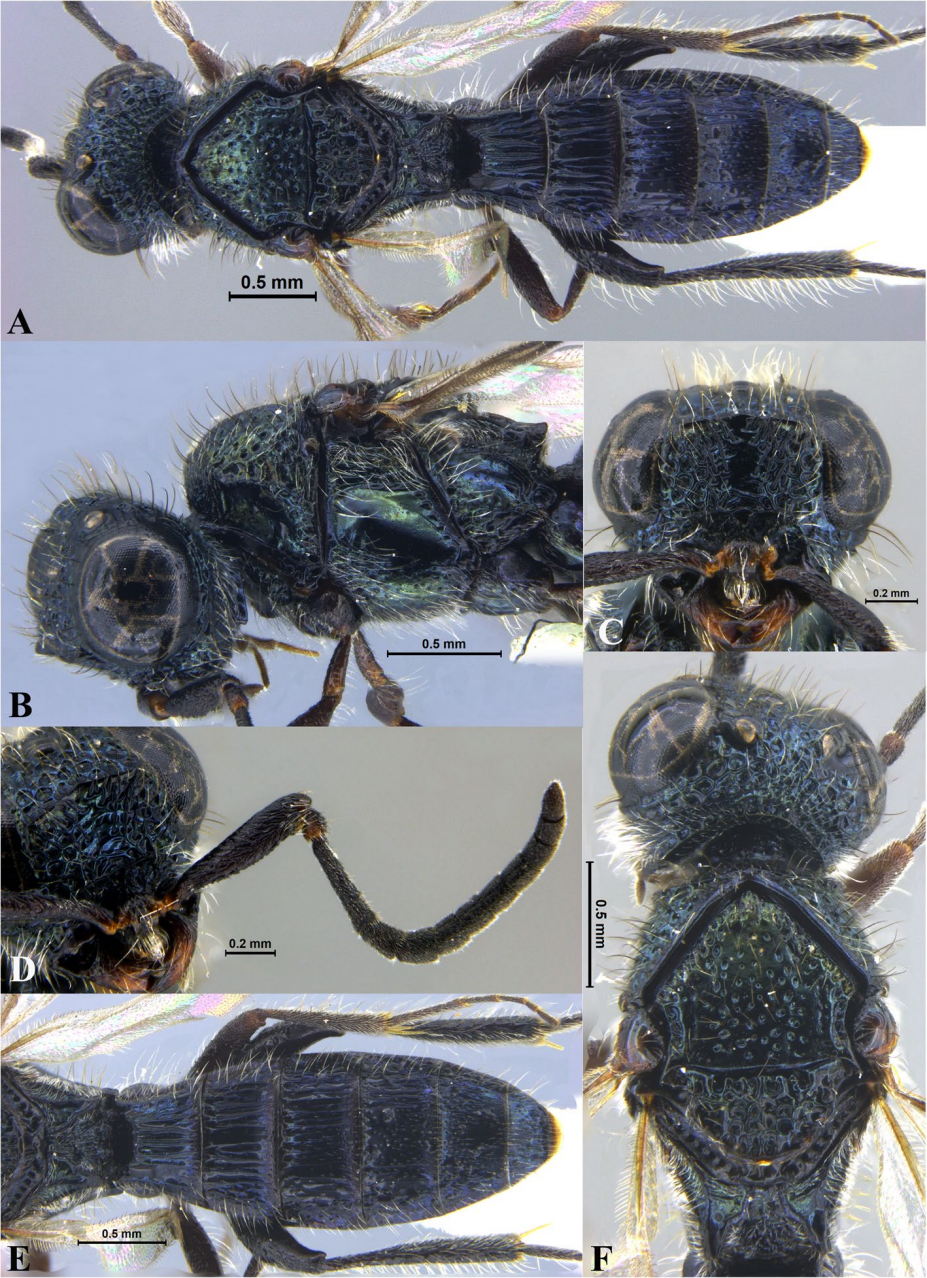
*Sparasion manavati ***sp. n**., female holotype. **A** Habitus, dorsal view. **B** Head and pleuron. **C** Frons. **D** Frons and antenna. **E** Metasoma. **F** Head and mesonotum

**Fig. 42:**
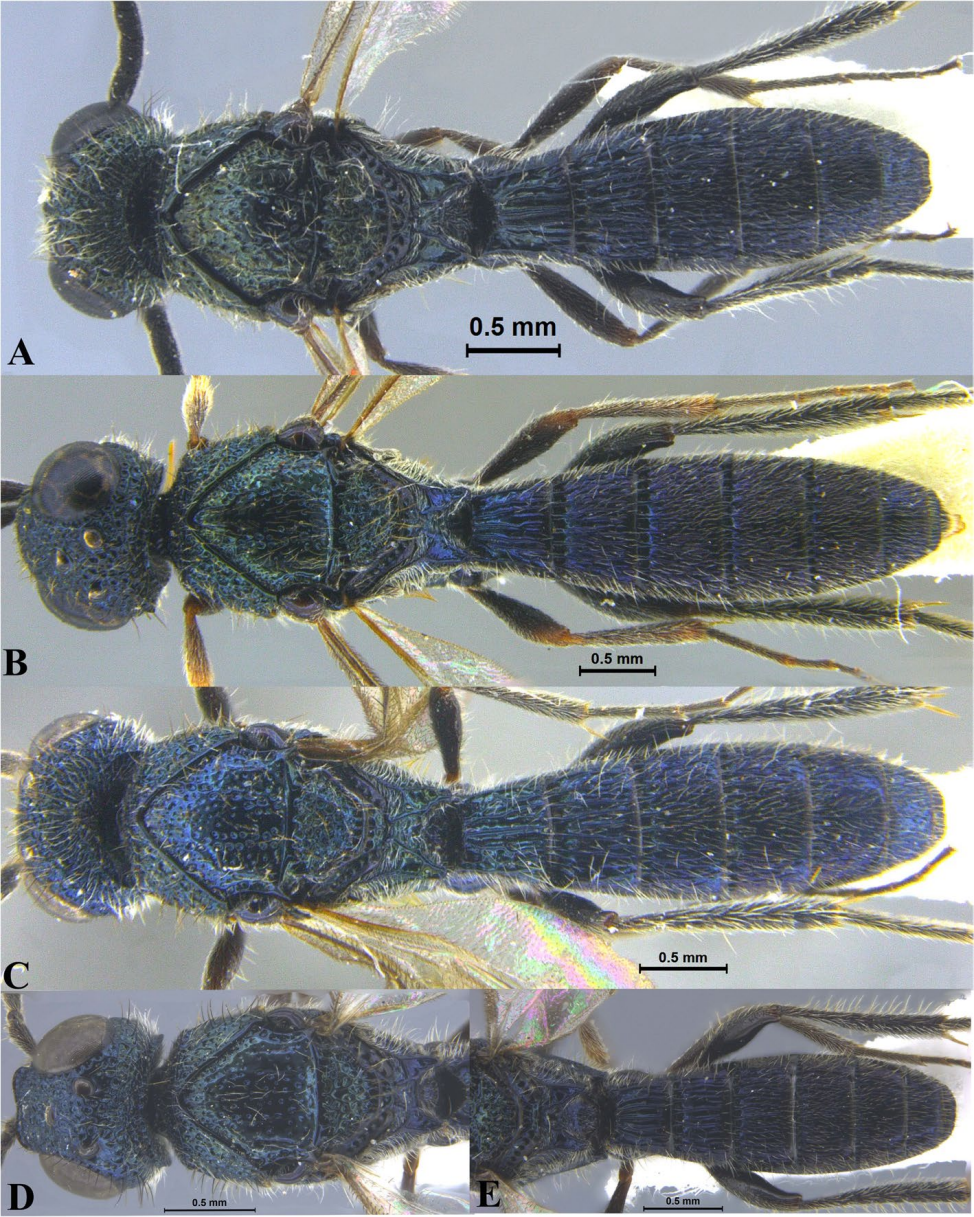
Males: **A** *Sparasion manavati. ***B ***S. pahadi*. **C ***S. rupavati. ***D ***S. ratnangi* (Head and mesosoma)*. ***E ***S. ratnangi* (Metasoma)

urn:lsid:zoobank.org:act:CE847A8E-7525-4AA5-B058-315F95A6A933

#### Diagnosis

*Sparasion manavati* sp. n. is distinct in having effaced longitudinal costate on medial T3; T4 with smooth area medially.

#### Material examined

Holotype: Female, (ICAR/NBAIR/P4626), **INDIA**: Karnataka: Udupi: Brahmavara, KVK, 13°25′51ʺN 74°44′43ʺE, 36 m, YPT, 28.V.2014. Paratype: 1 male (ICAR/NBAIR/P4627), Karnataka: Udupi: Brahmavara, KVK, 13°25′51ʺN 74°44′43ʺE, 36 m, YPT, 28.V.2014.

### Description

Female body length = 5.28 mm (*n* = 1); male body length = 4.91 mm (*n* = 1).

#### Colour

Head and metasoma steel blue; mesosoma steel green-blue; tegula red-brown; fore coxa black-brown, meso and meta coxae blue-brown, remainder of all legs black-brown; radicle, basal A1 and apical A2 red-brown; remainder of A1 and A2 and other antennomeres black-brown; mandibles red-brown with dark brown teeth.

#### Head

1.4 × as wide as high, as high as long. Setation on head: dense. IOS: 0.5 × head width, 0.8 × eye length. POL > LOL > OOL: 29.6:22.2:7.4. OOL: 0.6 × MOD. Compound eye: (L: W = 63.8:58.5). Setation of compound eye: sparsely setose. Anterior margin of frons: arcuate with deep medial indentation. Distance from level of anterior margin of compound eyes to anterior extension of frons: 0.7 × MOD. Number of transverse ledges on upper frons: one. Sculpture of upper frons: with polygonal and circular cells bearing setae with smooth interstices. Sculpture of lower frons: entirely with polygonal cells bearing setae except for a longitudinal smooth patch medially and sparse uneven carinae ventrally. Interantennal process: 1.2 × as long as wide, smooth. Transverse carina above interantennal process: with an acute medial notch. Area ventral to transverse carina above interantennal process: setigerous punctate. Sculpture on vertex: anteriorly with circular cells with smooth interstices, followed by polygonal cells bearing setae and an uneven transverse carina, and posteriorly smooth with setigerous punctae; smooth area around anterior ocellus present; an irregular semicircular area present in anterolateral region as well as posterior to lateral ocellus. Sculpture of posterior orbital furrow: dorsally foveate, ventrally with depressions. Genal carina: absent. Sculpture of gena: with dense white setigerous punctae and foveae. Sculpture on A1: densely setigerous punctate. A1: 4.4 × as long as wide. Length of A3: 0.6 × A1 and 3.7 × A2.

#### Mesosoma

Sculpture of dorsal pronotum: with setigerous depressions. L: W of mesoscutum: 75.0:78.4. Sculpture of mesoscutum: smooth with setigerous punctae and foveae, a short ligula present anteromedially. Notaulus: present. Sculpture of notaulus: foveate. Mesoscutal humeral sulcus: with depressions. Mesoscutal suprahumeral sulcus: with depressions. Parapsidal line: indicated as furrow. Scutoscutellar sulcus: foveate laterally. L: W of mesoscutellum: 39.6:60.3. Sculpture of mesoscutellum: with compact polygonal cells bearing setae and an incomplete transverse furrow anteriorly. Sculpture of dorsellum: anteriorly foveate, posteriorly smooth, posterior margin straight. Sculpture of outer lateral propodeal area: anteriorly sparsely setose and posteriorly smooth. Sculpture of inner lateral propodeal area: anteriorly densely setose, posteriorly smooth with a depression. Lateral propodeal carina: anteriorly oblique and posteriorly arched. Posterior propodeal projection: pointed, not extending to anterior margin of T1. Sculpture of metasomal depression: sparsely setose, posteriorly rugose. Plical area: anteriorly densely setose, posteriorly smooth with sparse punctae. Sculpture of propleuron: anteriorly smooth, posteriorly foveate. Sculpture of lateral pronotal area: smooth. Posterior pronotal sulcus: with irregular cells. Pronotal cervical sulcus: not foveate. Speculum of mesopleuron: transversely carinate, interspersed with punctae, densely setose. Postacetabular sulcus: densely setose, sculpture hidden. Prespecular sulcus: foveate. Mesepimeral sulcus: foveate. Posterior mesepimeral area: smooth, subequal to width of mesepimeral sulcus. Mesopleural carina: indicated anteriorly with foveae on dorsal margin. Sculpture of femoral depression: smooth. Mesopleural pit: present. Sculpture of ventral mesopleuron: anteriorly with polygonal cells bearing dense setae, remainder weakly reticulate with punctae, densely setose. Sculpture of metapleuron: dorsal metapleural area weakly rugose with dense setae on anterior margin; ventral metapleural area dorsally smooth, ventrally with sparse polygonal cells and posteroventrally with irregular transverse carinae. Metapleural sulcus: foveate, medially indicated by a furrow. Paracoxal sulcus: indicated by polygonal cells with setae. Metapleural epicoxal sulcus: with uneven sculpture and sparse setae.

#### Fore wing

L: W: 327.5:115.5. Transparency: strongly infuscate. Lengths of R: R1: r-rs in ratio of 150:74:36. R: basally closer and gradually distant from anterior margin of wing. Anterior margin of fore wing: upcurved basally and weakly downcurved prior to R1.

#### Metasoma

L: W of metasoma: 244.7:101.3. Ratio of length of T1: T2: T3: T4: T5: 38.3:43.6:45.7:43.1:39.4. Anterior margin of T1: convex. Sculpture of T1: longitudinally ribbed costate medially, laterally smooth with setigerous punctae. Sculpture of T2: basal foveae present, medially smooth, submedially and sublaterally with longitudinally ribbed costae; laterally smooth with setigerous punctae. Sculpture of T3: same as T2 except for a wider smooth area medially with effaced longitudinal costae and sparse setae. Sculpture of T4: same as T3 except for smooth area medially. Sculpture of T5: smooth with setigerous punctae and sparse short longitudinal costae sublaterally on anterior margin. Sculpture of T6: smooth with setigerous punctae.

#### Male

Similar to female.

#### Etymology

This species is named ‘Manavati’ after a *ragam* or melodic structure in South Indian (Carnatic) classical music which means ‘the bride’.

### *Sparasion meghmalhari* Veenakumari sp. n. (Figs. 33H, 36F, 43A–F, 44A)

**Fig. 43:**
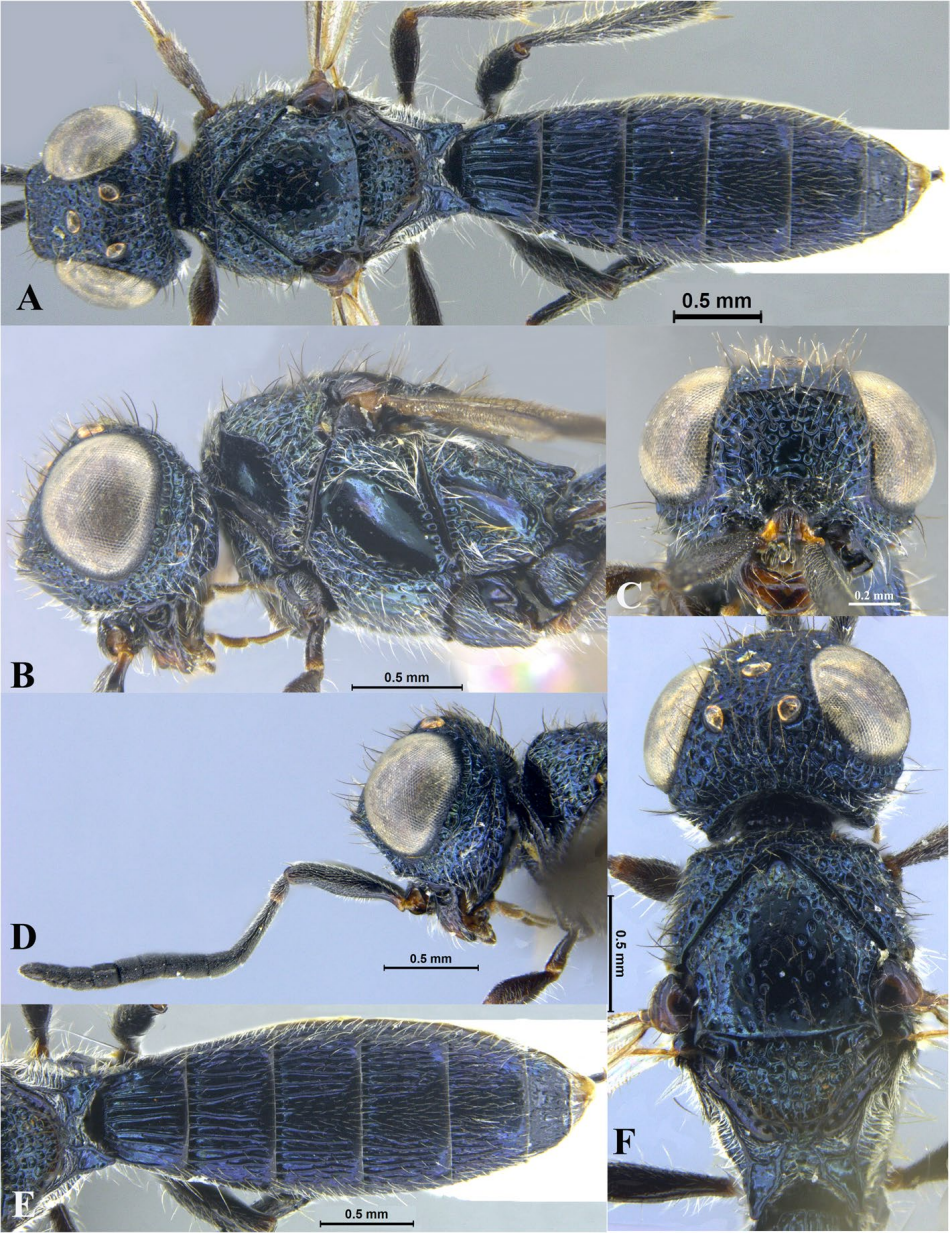
*Sparasion meghmalhari ***sp. n**., female holotype. **A** Habitus, dorsal view. **B** Head and pleuron. **C** Frons. **D** Head and antenna. **E** Metasoma. **F** Head and mesonotum

**Fig. 44:**
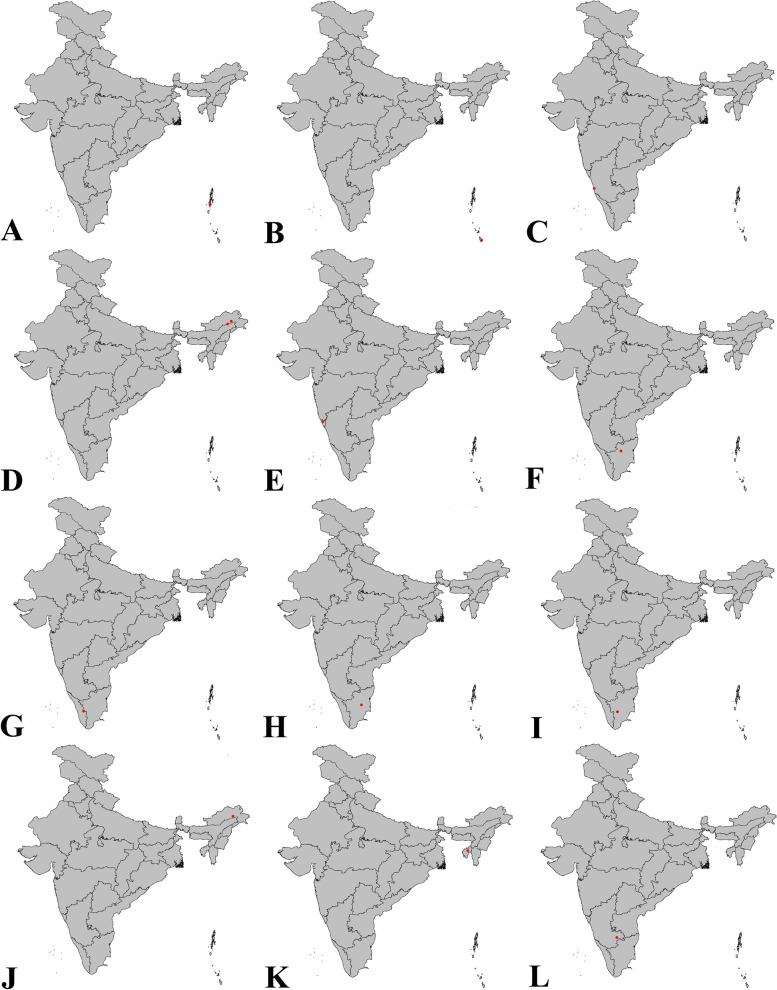
Distribution of new species of *Sparasion* in India: **A ***S*. *meghmalhari ***sp. n**. **B ***S*. *pahadi ***sp. n**. **C ***S*. *ratnangi ***sp. n**. **D ***S*. *rupavati ***sp. n**. **E ***S*. *salagami ***sp. n**. **F ***S*. *shulini ***sp. n**. **G ***S*. *sivaranjini ***sp. n**. **H ***S*. *syamalangi ***sp. n**. **I ***S*. *todi ***sp. n**. **J ***S*. *vanaspati ***sp. n**. **K ***S*. *visvambari ***sp. n**. **L ***S*. *zeelafi ***sp. n**

urn:lsid:zoobank.org:act:54F73C4C-8335-4D28-9649-FB34CE7F01BD

#### Diagnosis

*Sparasion meghmalhari* sp. n. is close to *S. travancoricus* but differs from it in the following characters: in *S. meghmalhari* sp. n. lower frons is with circular and polygonal cells with smooth interstices and gena is with polygonal cells. Conversely, in *S. travancoricus* lower frons is with a network of polygonal cells and gena is setigerous punctate.

#### Material examined

Holotype: Female, (ICAR/NBAIR/P4561), **INDIA**: Andaman and Nicobar Islands: South Andaman, Garacharma, Central Island Agricultural Research Institute, (CIARI), 11°36′21ʺN 92°42′21ʺE, 44 m, YPT, 22.II.2012. Paratypes: 6 females, (ICAR/NBAIR/P4562–P4567), Andaman and Nicobar Islands: South Andaman, Garacharma, CIARI, 11°36′21ʺN 92°42′21ʺE, 44 m, YPT, 22.II.2012; 5 females, (ICAR/NBAIR/P4568–P4572), Andaman and Nicobar Islands: South Andaman, Sippighat, Krishi Vigyan Kendra, 11°36′20ʺN 92°41′54ʺE, 8 m, YPT, 23.II.2012; 1 female, (ICAR/NBAIR/P4573), Andaman and Nicobar Islands: South Andaman, Garacharma, CIARI, 11°36′21ʺN 92°42′21ʺE, 44 m, YPT, 26.I.2013; 8 females, (ICAR/NBAIR/P4574–P4581), Andaman and Nicobar Islands: South Andaman, Sippighat, Krishi Vigyan Kendra, 11°36′20ʺN 92°41′54ʺE, 8 m, YPT, 22.III.2016.

### Description

Female body length = 5.52–5.84 mm (*n* = 12).

#### Colour

Head, mesosoma and metasoma steel blue; tegula black with brown patches; fore coxa black-brown, meso and meta coxae steel blue, remainder of all legs black-brown; radicle yellow–brown, remaining antennomeres black-brown; mandibles red-brown, with teeth dark brown.

#### Head

1.4 × as wide as high, 0.9 × as high as long. Setation on head: dense. IOS: 0.4 × head width, 0.7 × eye length. POL > LOL > OOL: 24.5:18.0:8.2. OOL: 0.5 × MOD. Compound eye: (L: W = 67.4:53.9). Setation of compound eye: glabrous. Anterior margin of frons: with a weak indentation medially. Distance from the level of anterior margin of compound eyes to anterior extension of frons in dorsal view: 1.2 × MOD. Number of transverse ledges on upper frons: one. Sculpture of upper frons: with polygonal cells bearing setae and with smooth interstices. Sculpture of lower frons: entirely with circular and polygonal cells bearing setae with smooth interstices. Interantennal process: 1.3 × as long as wide, smooth. Transverse carina above interantennal process: with an acute medial notch. Area ventral to transverse carina above interantennal process: with setigerous punctae except for smooth area on inner margin. Sculpture on vertex: anteriorly with polygonal cells with setae followed by an uneven transverse carina and posteriorly smooth with setigerous punctae; a smooth area present around anterior ocellus; an irregular smooth area present on anterior and posterior margins of lateral ocellus. Sculpture of posterior orbital furrow: with rectangular cells. Genal carina: absent. Sculpture of gena: with polygonal cells and foveae, and densely setose. Sculpture on A1: densely setigerous punctate. A1: 4.7 × as long as wide. Length of A3: 0.6 × A1 and 2.9 × A2.

#### Mesosoma

Sculpture of dorsal pronotum: with setigerous depressions. L: W of mesoscutum: 80.0:95.6. Sculpture of mesoscutum: anteromedially with a ligula, mesoscutum smooth with sparse setigerous punctae. Notaulus: present. Sculpture of notaulus: foveate. Mesoscutal humeral sulcus: foveate. Mesoscutal suprahumeral sulcus: foveate. Parapsidal line: indicated as furrow. Scutoscutellar sulcus: foveate. L: W of mesoscutellum: 37.0:59.6. Sculpture of mesoscutellum: with compact polygonal cells and an incomplete transverse furrow anteriorly. Sculpture of dorsellum: anteriorly foveate, medially with longitudinal carinae and posteriorly smooth, posterior margin weakly rounded. Sculpture of outer lateral propodeal area: anteriorly densely setose and posteriorly smooth. Sculpture of inner lateral propodeal area: anteriorly with dense pilosity, posteriorly smooth. Lateral propodeal carina: anterior half arched inwards and posterior half oblique. Posterior propodeal projection: rounded, extending on to anterior margin of T1. Sculpture of metasomal depression: densely setose with a medial transverse furrow. Plical area: anteriorly densely setose, posteriorly smooth with sparse setigerous punctae. Sculpture of propleuron: dorsally smooth, ventrally weakly foveate. Sculpture of lateral pronotal area: smooth. Posterior pronotal sulcus: with ovoid cells. Pronotal cervical sulcus: not foveate. Speculum of mesopleuron: foveate, interspersed with sparse transverse carinae, densely setose. Postacetabular sulcus: foveate. Prespecular sulcus: with circular cells. Mesepimeral sulcus: foveate. Posterior mesepimeral area: smooth, subequal in width of mesepimeral sulcus. Mesopleural carina: indicated anteriorly with foveae on dorsal margin. Sculpture of femoral depression: smooth. Mesopleural pit: present. Sculpture of ventral mesopleuron: anteriorly with polygonal cells, remainder smooth with sparse punctae, densely setose. Sculpture of metapleuron: dorsal metapleural area smooth with setae on anterior margin; ventral metapleural area dorsally smooth and in ventral half with a row of foveae bearing brown setae followed by dense polygonal cells bearing white setae. Metapleural sulcus: foveate, medially indicated by a furrow. Paracoxal sulcus: foveate. Metapleural epicoxal sulcus: with depressions, densely setose.

#### Fore wing

L: W: 319.7:112.8. Transparency: strongly infuscate. Lengths of R: R1: r-rs in ratio of 148:73:38. R: almost parallel to anterior margin of wing. Anterior margin of wing: with a weak downcurve prior to R1.

#### Metasoma

L: W of metasoma: 263.5:93.6. Ratio of length of T1: T2: T3: T4: T5: 44.8:46.8:45.8:46.8:42.7. Anterior margin of T1: straight. Sculpture of T1: medially longitudinally ribbed costate, laterally smooth. Sculpture of T2: basal foveae present, followed by longitudinal ribbed costae medially; laterally smooth with setigerous punctae and smooth area posteromedially. Sculpture of T3: same as T2 except for smooth patch medially with setae. Sculpture of T4: same as T3 except for larger smooth patch medially with setigerous punctae. Sculpture of T5: basal foveae present sublaterally, short longitudinal costae present submedially on anterior margin, remainder smooth with setigerous punctae. Sculpture of T6: smooth with setigerous punctae.

#### Male

Unknown.

#### Etymology

This species is named ‘Meghmalhar’ after a *raga*, or melodic structure, in North Indian (Hindustani) classical music (meaning clouds which bring rain) sung by the daughter of Tansen (the legendary musician in Mughal emperor Akbar’s court) to quench the fire resulting from the rendition of the *raga* Deepak (the melody of fire) by her father.

### *Sparasion micromerus* Kozlov & Lê (Figs. 19, 36G, 45A–F)

**Fig. 45:**
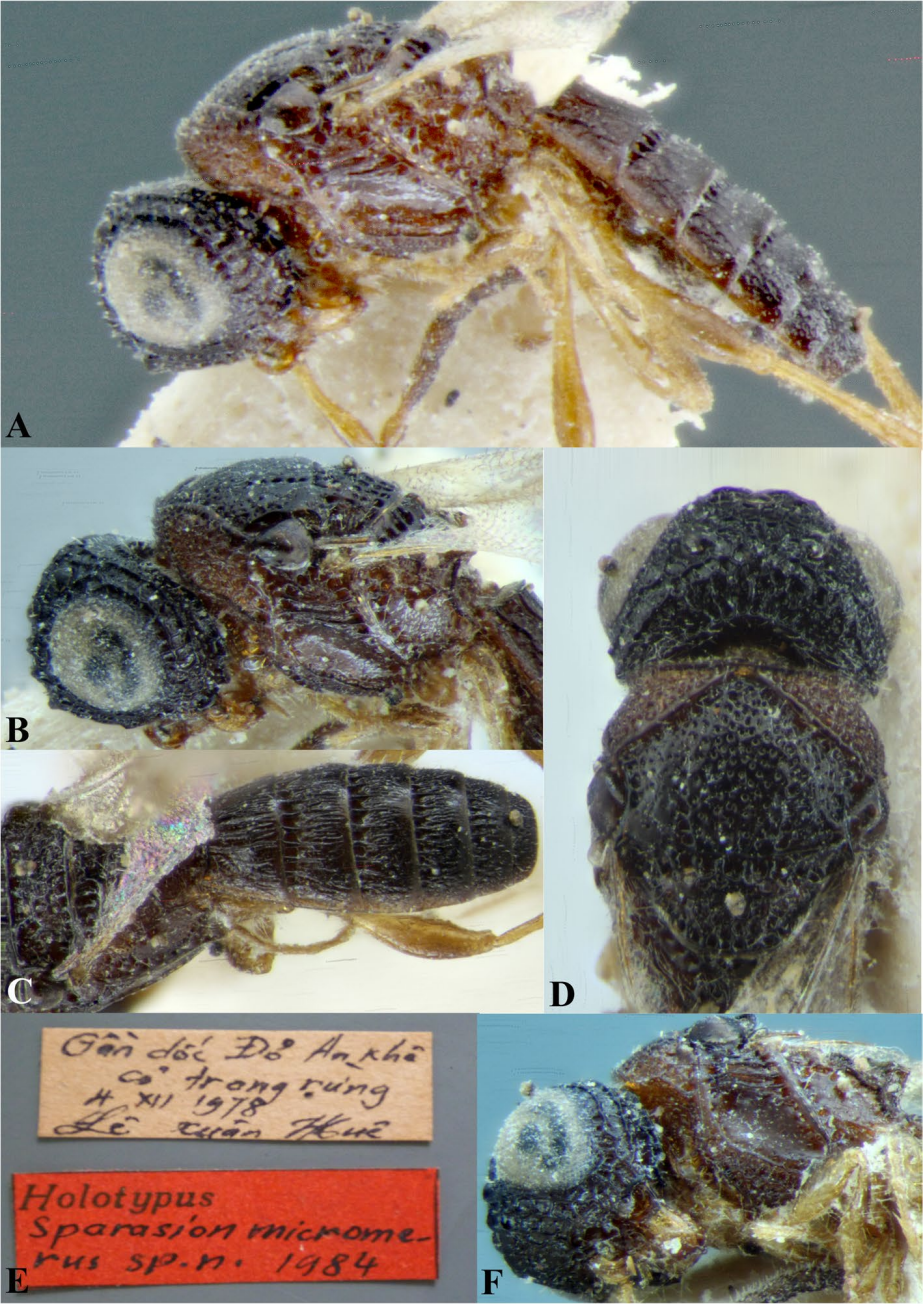
*Sparasion micromerus* Kozlov and Lê, male holotype. **A** Habitus, lateral view. **B** Head and pleuron. **C** Mesoscutellum and metasoma. **D** Head and mesonotum. **E** Type labels. **F** Frons and pleuron. (Photos: Drs. N. F. Johnson © Ohio State University and E. J. Talamas, Florida Department of Agriculture and Consumer Services)

*Sparasion micromerus* Kozlov and Lê, 2000: 203, 206, 356. Original description, keyed.

#### Diagnosis

*Sparasion micromerus* is close to *S. visvambari* sp. n. but differs from it in the following characters: in *S. micromerus* T2–T4 are entirely with dense longitudinal costae except for a narrow posterior margin; T5 is costate in anterior half and remainder smooth; mesoscutum is with closely spaced foveae. Conversely, in *S. visvambari* sp. n. T2–T4 are with sparse longitudinal costae in anterior half, remainder smooth; T5 is smooth with sparse setigerous punctae; foveae of mesoscutum widely spaced.

#### Material examined

Holotype, male, **VIETNAM**: Gia Lai: An Khe*:* Buon Luoi*,* 04.XII.1978, leg. Lê, X. H.

### Description

Male body length = 3 mm.

#### Colour

Head and mesonotum black, and metasoma black-brown; tegula black with brown patches; dorsal pronotum and pleuron honey-brown; all coxae honey brown, remainder of legs yellow–brown; radicle, A1–A2 yellow-brown, remaining antennomeres brown-black; mandibles yellow-brown.

#### Head

Setation on head: sparse. Setation of compound eye: glabrous. Anterior margin of frons: sinuous. Distance from level of anterior margin of compound eyes to anterior extension of frons in dorsal view: > MOD. Number of transverse ledges on upper frons: three. Sculpture of upper frons: anteriorly smooth, followed by a row of effaced large polygonal cells bearing setae and two rows of large polygonal cells anterior to anterior ocellus. Sculpture of lower frons: with polygonal cells bearing setae. Transverse carina above interantennal process: with a medial notch. Area ventral to transverse carina above interantennal process: weakly foveate. Sculpture on vertex: anteriorly with large polygonal cells, followed by two uneven transverse carinae with polygonal cells between them and posteriorly smooth with setigerous punctae; sparse longitudinal carinae extending onto vertex from medial occipital carina; anterior ocellus without smooth area around; irregular smooth area present on posterior margin of lateral ocellus. Sculpture of posterior orbital furrow: foveate. Genal carina: present. Sculpture of gena: anteriorly with polygonal cells and posteriorly smooth. Sculpture on A1: smooth with sparse long setae.

#### Mesosoma

Sculpture of dorsal pronotum: with dense setigerous depressions. L: W of mesoscutum: 38:50. Sculpture of mesoscutum: smooth with dense foveae. Notaulus: present. Sculpture of notaulus: foveate. Mesoscutal humeral sulcus: with depressions. Mesoscutal suprahumeral sulcus: with depressions. Parapsidal line: carinate. Scutoscutellar sulcus: foveate. L: W of mesoscutellum: 21:32. Sculpture of mesoscutellum: with closely packed polygonal cells except for a smooth patch anteromedially. Sculpture of outer lateral propodeal area: smooth. Sculpture of inner lateral propodeal area: anteriorly sparsely setose followed by a medial transverse carina, posteriorly smooth. Plical area: anteriorly setose and posteriorly with shallow depressions. Sculpture of lateral pronotal area: smooth. Posterior pronotal sulcus: foveate. Pronotal cervical sulcus: foveate. Speculum of mesopleuron: transversely carinate. Postacetabular sulcus: foveate. Prespecular sulcus: foveate. Mesepimeral sulcus: foveate. Mesepimeral area: smooth, subequal to mesepimeral sulcus in width. Mesopleural carina: present with a row of shallow depressions anteriorly. Sculpture of femoral depression: smooth. Mesopleural pit: present. Sculpture of ventral mesopleuron: smooth with setigerous punctae. Sculpture of metapleuron: dorsal metapleural area not distinct; ventral metapleural area dorsally smooth and ventrally with sparse foveae. Metapleural sulcus: foveate. Paracoxal sulcus: foveate.

#### Fore wing

L: W: 2.6 × as long as wide. Transparency: weakly infuscate. Lengths of R1: 1.5 × r-rs.

#### Metasoma

L: W of metasoma: 72:35. Ratio of length of T1: T2: T3: T4: T5: 16.8:15.4:14.0:11.9:9.1. Anterior margin of T1: convex. Sculpture of T1: entirely longitudinally costate, except for rugose area laterally. Sculpture of T2: basal foveae present, followed by longitudinal costae, area between these costae with short longitudinal costae; laterally rugose-punctate and posteriorly with a narrow smooth patch. Sculpture of T3: basal foveae present, followed by longitudinal costae, area between costae rugose and with short longitudinal carinae, laterally rugose-punctate and posteriorly smooth. Sculpture of T4: same as T3. Sculpture of T5: same as T4. Sculpture of T6: smooth with setigerous punctae.

#### Female

Unknown.

#### Remarks

The holotype is present in IEBR, Vietnam.

### *Sparasion pahadi* Veenakumari sp. n. (Figs. 5A–F, 33I, 36H, 42B, 44B)

urn:lsid:zoobank.org:act:95423CEB-F6FB-493B-A783-7BB482A0F25C

#### Diagnosis

*Sparasion pahadi* sp. n. shares the character state – elongate and narrow posterior propodeal projection which extends onto anterior margin of T1 and a sinuous lateral propodeal carina – with *S. sinensis*. It differs from *S. sinensis* in having foveate-punctate mesoscutum, presence of notaulus and mesoscutellum with densely packed polygonal cells. Conversely, in *S. sinensis*, mesoscutum is smooth with punctae, notaulus is absent and mesoscutellum is anteriorly smooth and posteriorly with two rows of foveae.

#### Material examined

Holotype: Female, (ICAR/NBAIR/P4601), **INDIA**: Andaman and Nicobar Islands: Great Nicobar I.: Campbell Bay, 7°00′27ʺN 93°54′17ʺE, 13 m, YPT, 22.III.2016. Paratypes: 8 females, (ICAR/NBAIR/P4602–4609), Andaman and Nicobar Islands: Great Nicobar I.: Campbell Bay, 7°00′27ʺN 93°54′17ʺE, 13 m, YPT, 22.III.2016; 1 male, (ICAR/NBAIR/P4610), Andaman and Nicobar Islands: Great Nicobar I.: Campbell Bay, 7°00′27ʺN 93°54′17ʺE, 13 m, YPT, 22.III.2016.

### Description

Female body length = 6.98–7.23 mm (*n* = 9); male body length = 6.95 mm (*n* = 1).

#### Colour

Head and mesosoma steel green–blue; metasoma steel blue, with T1 and T6 exhibiting a tinge of steel green; tegula brown-black; fore coxa black, meso and meta coxae steel blue, remainder of legs black-brown; radicle, basal and apical A1 red-brown, remainder of A1 brown-black, A2 orange-brown, remaining antennomeres black-brown; mandibles red-brown with teeth dark brown.

#### Head

1.5 × as wide as high, as high as long. Setation on head: dense. IOS: 0.4 × head width, 0.8 × eye length. POL > LOL > OOL: 28.9:22.1:9.3. OOL: 0.6 × MOD. Compound eye: (L: W = 77.4:68.9). Setation of compound eye: glabrous. Anterior margin of frons: weakly arcuate. Distance from the level of anterior margin of compound eyes to anterior extension of frons in dorsal view: 0.5 × MOD. Number of transverse ledges on upper frons: one. Sculpture of upper frons: with polygonal cells bearing setae and weakly rugose interstices. Sculpture of lower frons: dorsally and laterally with polygonal cells bearing setae, medially smooth with transverse carinae. Interantennal process: 1.3 × as long as wide, smooth. Transverse carina above interantennal process: with acute medial notch. Area ventral to transverse carina above interantennal process: with setigerous punctae. Sculpture on vertex: anteriorly with polygonal and circular cells bearing setae with smooth interstices, medially with compact polygonal cells, posteriorly smooth with setigerous punctae; smooth area present around anterior ocellus; narrow smooth area present on anterior and posterior margins of lateral ocellus. Sculpture of posterior orbital furrow: foveate, except for depressions medially. Genal carina: absent. Sculpture of gena: smooth with white setigerous foveae and punctae, setae dense. Sculpture on A1: densely setigerous punctate. A1: 4.6 × as long as wide. Length of A3: 0.6 × A1 and 2.2 × A2.

#### Mesosoma

L: W of mesoscutum: 95.9:100.0. Sculpture of dorsal pronotum: with setigerous depressions. Sculpture of mesoscutum: smooth with setigerous punctae and foveae, a short ligula present anteromedially. Notaulus: present. Sculpture of notaulus: foveate. Mesoscutal humeral sulcus: with elongate depressions. Mesoscutal suprahumeral sulcus: foveate. Parapsidal line: indicated as a furrow. Scutoscutellar sulcus: foveate. L: W of mesoscutellum: 47.9:83.6. Sculpture of mesoscutellum: with compact polygonal cells with setae and an incomplete transverse furrow anteriorly. Sculpture of dorsellum: anteriorly foveate, posteriorly smooth with a medial vertical carina, posterior margin with a weak medial spine. Sculpture of outer lateral propodeal area: anteriorly densely setose, posteriorly smooth. Sculpture of inner lateral propodeal area: anteriorly densely setose, posteriorly smooth. Lateral propodeal carina: sinuous. Posterior propodeal projection: rounded, extending beyond anterior margin of T1. Sculpture of metasomal depression: densely setose. Plical area: anteriorly densely setose, remainder with shallow depressions, sparsely setose. Sculpture of propleuron: smooth, with weak carinae posteriorly. Sculpture of lateral pronotal area: smooth with sparse setigerous punctae posteriorly. Posterior pronotal sulcus: with irregular cells. Pronotal cervical sulcus: not foveate. Speculum of mesopleuron: transversely carinate interspersed with setigerous punctae, densely setose. Postacetabular sulcus: foveate. Prespecular sulcus: foveate. Mesepimeral sulcus: foveate. Posterior mesepimeral area: smooth, subequal to width of mesepimeral sulcus. Mesopleural carina: indicated anteriorly with foveae on dorsal margin. Sculpture of femoral depression: smooth. Mesopleural pit: present. Sculpture of ventral mesopleuron: anteriorly with polygonal cells and dense setae, remainder smooth with sparse setigerous punctae. Sculpture of metapleuron: dorsal metapleural area smooth with dense setae on anterior margin; ventral metapleural area dorsally smooth and ventrally with a row of foveae bearing brown setae followed by dense polygonal cells bearing white setae. Metapleural sulcus: foveate, medially indicated as a furrow. Paracoxal sulcus: indicated as wide foveae. Metapleural epicoxal sulcus: dense setae concealing the sculpture.

#### Fore wing

L: W: 385.0:131.7. Transparency: strongly infuscate. Lengths of R: R1: r-rs in ratio of 181:100:45. R: distant from anterior margin of wing. Anterior margin of fore wing: with a slight downcurve prior to R1.

#### Metasoma

L: W of metasoma: 340.3:108.3. Ratio of length of T1: T2: T3: T4: T5: 62.5:62.5:62.5:62.5:55.5. Anterior margin of T1: straight with a small spine anteromedially. Sculpture of T1: longitudinally ribbed costae, laterally smooth with setigerous punctae, posteromedially smooth. Sculpture of T2: basal foveae present, followed by longitudinally ribbed costae; laterally smooth with setigerous punctae, and posteromedially smooth; Sculpture of T3: same as T2 except for narrow smooth patch with dense setae medially. Sculpture of T4: same as T3 except for a wider smooth patch with dense setae medially. Sculpture of T5: same as T4 with short longitudinal costae present submedially on anterior margin. Sculpture of T6: basal foveae present, remainder smooth with setigerous punctae.

#### Male

Similar to female.

#### Etymology

This species is named ‘Pahadi’ (meaning ‘of the mountains’) after a simple *raga* or melodic structure in North Indian (Hindustani) classical music derived from the folk music of the people of the Himalaya reflecting a romantic mood imbued with intense sadness.

### *Sparasion philippinensis* Kieffer (Figs. 6A–D, 19, 33J, 36I)

*Sparasion philippinense* Kieffer, 1913: 320.

*Sparasion philippinensis*: Kieffer, 1926: 284, 293. Description, keyed.

*Sparasion philippinensis*: Kelner-Pillault, 1958: 152. Type information.

#### Diagnosis

This species is distinct in having T5 and T6 very narrow and elongate and R is distant and parallel from anterior margin of wing along its entire length.

#### Material examined

Holotype, female (MNHN, Paris, EY34252), **PHILIPPINES**: Laguna, Los Baños, leg. P. I. Baker.

### Description

Female body length = 6 mm. Male body length = 5.5 mm.

#### Colour

Head, mesosoma and metasoma steel blue; tegula black blue-black; all legs black-brown; radicle yellow, A1–A3 red-brown, remaining antennomeres brown-black; mandible brown-black.

#### Head

Setation on head: dense. Setation of compound eye: glabrous. Anterior margin of frons: arcuate with deep medial indentation. Number of transverse ledges on upper frons: one. Sculpture of upper frons: with closely packed circular cells bearing setae. Sculpture of lower frons: head with network of polygonal cells with transverse carinae dorsally. Sculpture on vertex: with polygonal cells bearing setae, with sparse longitudinal carinae extending from medial occipital carina; a smooth area present around anterior ocellus; a smooth triangular area present posterior to lateral ocellus. Sculpture of posterior orbital furrow: dorsally with smaller and ventrally with large depressions. Genal carina: absent. Sculpture of gena: dorsally with polygonal cells and ventrally smooth, setose. Sculpture on A1: densely setigerous punctate.

#### Mesosoma

Sculpture of dorsal pronotum: with setigerous depressions with smooth interstices. L: W of mesoscutum: 76:86. Sculpture of mesoscutum: smooth with sparse foveae and punctae bearing setae. Notaulus: present. Sculpture of notaulus: foveate. Mesoscutal humeral sulcus: foveate. Mesoscutal suprahumeral sulcus: foveate. Parapsidal line: indicated as a shallow furrow. Scutoscutellar sulcus: foveate. L: W of mesoscutellum: 43:60. Sculpture of mesoscutellum: with compact polygonal cells, and an incomplete transverse furrow anteriorly. Sculpture of dorsellum: foveate. Sculpture of lateral pronotal area: smooth. Posterior pronotal sulcus: foveate. Pronotal cervical sulcus: not foveate. Speculum of mesopleuron: transversely carinate, interspersed with setigerous punctae. Prespecular sulcus: foveate. Mesepimeral sulcus: foveate. Mesepimeral area: smooth, narrower than mesepimeral sulcus. Sculpture of femoral depression: smooth. Sculpture of metapleuron: ventral metapleural area dorsally smooth and ventrally with polygonal cells bearing brown and white setae. Metapleural sulcus: foveate. Paracoxal sulcus: foveate.

#### Fore wing

L: W: 322:129. Transparency: strongly infuscate. Lengths of R: R1: r-rs in ratio of 157:60:40. R: distant from anterior margin of wing along its entire length. Anterior margin of wing: no downcurve prior to R1.

#### Metasoma

Sculpture of T1–T6: Basal foveae present on T2–T4; T1–T3 longitudinally costate, laterally smooth with sparse setigerous punctae; T4 longitudinal costate anteriorly and posteriorly smooth with sparse punctae; T5 with short, sparse longitudinal costae sublaterally on anterior margin, remainder smooth with sparse setigerous punctae; T6 smooth with sparse punctae.

#### Male

Described in Kieffer [11]. Not examined in this study.

#### Remarks

Holotype present at MNHN. The specimen is in good condition.

### *Sparasion ratnangi* Veenakumari sp. n. (Figs. 7A–F, 33L, 36J, 42D–E, 44C)

urn:lsid:zoobank.org:act:D7492903-0879-4780-9490-3E55D9D02884

#### Diagnosis

*Sparasion ratnangi* sp. n. is close to *S. sivaranjini* sp. n. and *S. rupavati* sp. n. but differs from them in the following characters: in *Sparasion ratnangi* sp. n. T5 is entirely punctate and lateral propodeal area posteriorly not extending onto anterior margin of T1. Conversely, in the latter two species T5 is longitudinally costate interspersed with punctae and lateral propodeal areas posteriorly extending onto anterior margin of T1.

#### Material examined

Holotype: Female, (ICAR/NBAIR/P4615), **INDIA**: Karnataka: Udupi: Brahmavara, KVK, 13°25′51ʺN 74°44′43ʺE, 36 m, YPT, 28.V.2014. Paratypes: 3 males (ICAR/NBAIR/P4623–P4625), Karnataka: Udupi: Brahmavara, KVK, 13°25′51ʺN 74°44′43ʺE, 36 m, YPT, 28.V.2014.

### Description

Female body length = 5.52 mm (*n* = 1); male body length = 5.23–5.38 mm (*n* = 3).

#### Colour

Head and metasoma steel blue; mesosoma steel green–blue; tegula black-brown; fore coxa black-brown, meso and meta coxae blue-brown, remainder of all legs black-brown; radicle, basal A1 red-brown; remainder of A1 and other antennomeres black-brown; mandibles red-brown with teeth dark brown.

#### Head

1.5 × as wide as high, 0.9 × as high as long. Setation on head: dense. IOS: 0.5 × head width, 0.9 × eye length. POL > LOL > OOL: 32.1:25.0:7.1. OOL: 0.6 × MOD. Compound eye: (L: W = 64.7:52.9). Setation of compound eye: glabrous. Anterior margin of frons: arcuate with medial indentation. Distance from level of anterior margin of compound eyes to anterior extension of frons: 0.8 × MOD. Number of transverse ledges on upper frons: one. Sculpture of upper frons: with circular cells bearing setae with smooth interstices. Sculpture of lower frons: entirely with polygonal cells bearing setae except for two short transverse carinae posteromedially. Interantennal process: 1.2 × as long as wide, smooth. Transverse carina above interantennal process: with an acute medial notch. Area ventral to transverse carina above interantennal process: setigerous punctate. Sculpture on vertex: anteriorly with circular cells with smooth interstices, followed by polygonal cells bearing setae and an uneven transverse carina, and posteriorly smooth with setigerous punctae; smooth area around anterior ocellus present; an irregular semicircular area present on anterior and posterior margins to lateral ocellus. Sculpture of posterior orbital furrow: foveate with irregular depressions medially. Genal carina: absent. Sculpture of gena: smooth with dense white setigerous punctae. Sculpture on A1: densely setigerous punctate. A1: 4.0 × as long as wide. Length of A3: 0.7 × A1 and 3.3 × A2.

#### Mesosoma

Sculpture of dorsal pronotum: with setigerous depressions and punctae. L: W of mesoscutum: 80.0:86.7. Sculpture of mesoscutum: smooth with setigerous punctae and foveae, a short ligula present anteromedially. Notaulus: absent. Mesoscutal humeral sulcus: with depression. Mesoscutal suprahumeral sulcus: with uneven depression. Parapsidal line: indicated as furrow. Scutoscutellar sulcus: foveate. L: W of mesoscutellum: 40.0:64.4. Sculpture of mesoscutellum: with compact polygonal cells bearing setae and an incomplete transverse furrow anteriorly. Sculpture of dorsellum: foveate with small smooth area posterolaterally, posterior margin almost straight. Sculpture of outer lateral propodeal area: smooth with sparse setae. Sculpture of inner lateral propodeal area: anteriorly densely setose, posteriorly smooth. Lateral propodeal carina: anteriorly oblique, posteriorly arched. Posterior propodeal projection: pointed, not extending to anterior margin of T1. Sculpture of metasomal depression: sparsely setose. Plical area: anteriorly densely setose, posteriorly smooth with shallow depressions. Sculpture of propleuron: anteriorly smooth, posteriorly foveate. Sculpture of lateral pronotal area: smooth. Posterior pronotal sulcus: with irregular cells. Pronotal cervical sulcus: foveate posteriorly. Speculum of mesopleuron: transversely carinate, interspersed with punctae, densely setose. Postacetabular sulcus: densely setose, sculpture not visible. Prespecular sulcus: foveate. Mesepimeral sulcus: foveate. Posterior mesepimeral area: smooth, subequal to width of mesepimeral sulcus. Mesopleural carina: indicated anteriorly, with foveae on dorsal margin. Sculpture of femoral depression: smooth. Mesopleural pit: present. Sculpture of ventral mesopleuron: anteriorly with polygonal cells bearing sparse setae, remainder with sparse setigerous punctae. Sculpture of metapleuron: dorsal metapleural area smooth with setae on anterior margin; ventral metapleural area dorsally smooth, and ventrally with a row of foveae bearing brown setae, followed by dense polygonal cells with white setae. Metapleural sulcus: foveate, medially indicated by a furrow. Paracoxal sulcus: foveate. Metapleural epicoxal sulcus: densely setose.

#### Fore wing

L: W: 320.5:117.1. Transparency: strongly infuscate. Lengths of R: R1: r-rs in ratio of 140:78:40. R: basally closer and gradually distant from anterior margin of wing. Anterior margin of fore wing: weakly downcurved prior to R1.

#### Metasoma

L: W of metasoma: 253.4:97.7. Ratio of length of T1: T2: T3: T4: T5: 42.1:45.5:50.0:47.4:37.5. Anterior margin of T1: straight. Sculpture of T1: longitudinally ribbed costate medially, laterally smooth with punctae and dense setae. Sculpture of T2: basal foveae present, laterally smooth with setigerous punctae, posteriorly smooth. Sculpture of T3: same as T2 except for a smooth area medially with sparse setae. Sculpture of T4: same as T3 except for dense setae on medial smooth area. Sculpture of T5: smooth with punctae and dense setae medially. Sculpture of T6: smooth with setigerous punctae.

#### Male

Similar to female.

#### Etymology

This species is named ‘Ratnangi’ after a *ragam* or melodic structure in South Indian (Carnatic) classical music meaning ‘one with gems for limbs’.

### *Sparasion rupavati* Veenakumari sp. n. (Figs. 8A–F, 33K, 36K, 42C, 44D)

urn:lsid:zoobank.org:act:D208311E-6C87-444B-A097-5CCC28749179

#### Diagnosis

*Sparasion rupavati* sp. n. is close to *S. sivaranjini* sp. n. but it differs from it in the following characters: in *S. rupavati* sp. n. upper frons is with polygonal cells without smooth interstices, OOL is at least 0.7 × MOD and metasoma is narrow and elongate. Conversely, in *S. sivaranjini* sp. n. upper frons is with circular cells with smooth interstices, OOL at most 0.3 × MOD and metasoma is short and wide.

#### Material examined

Holotype: Female, (ICAR/NBAIR/P4588), **INDIA**: Assam: Dhemaji: Simen Chapori, 27°43′19ʺN 94°52′05ʺE, 120 m, SN, 06.V.2014. Paratypes: 4 females, (ICAR/NBAIR/P4589–P4592), Arunachal Pradesh: Pasighat, College of Horticulture and Forestry (CHF), 28°04′28ʺN 95°19′28ʺE, 173 m, YPT, 05.V.2014; 1 female, (ICAR/NBAIR/P4593), Arunachal Pradesh: Pasighat, CHF, 28°04′28ʺN 95°19′28ʺE, 173 m, SN, 03.V.2014; 1 male, (ICAR/NBAIR/P4594), Assam: Dhemaji: Simen Chapori, 27°43′19ʺN 94°52′05ʺE, 120 m, SN, 06.V.2014; 1 male (ICAR/NBAIR/P4595), Arunachal Pradesh: Pasighat, CHF, YPT, 28°04′28ʺN 95°19′28ʺE, 173 m, YPT, 12.XI.2014.

### Description

Female body length = 5.73–6.02 mm (*n* = 6); male body length = 5.32–5.47 mm (*n* = 2).

#### Colour

Head, mesosoma and metasoma steel blue; tegula black-brown; fore coxa black-brown, meso- and metacoxae steel blue, remainder of legs black-brown; radicle and basal A1 yellow–brown, A2 brown, remaining antennomeres black-brown; mandibles red-brown with teeth dark brown.

#### Head

1.3 × as wide as high, 1.1 × as high as long. Setation on head: dense. IOS: 0.4 × head width, 0.8 × eye length. POL > LOL > OOL: 27.8:21.4:7.1. OOL: 0.7 × MOD. Compound eye: (L: W = 64.5:48.3). Setation of compound eye: glabrous. Anterior margin of frons: arcuate with medial indentation. Distance from the level of anterior margin of compound eyes to anterior extension of frons in dorsal view: 0.6 × MOD. Number of transverse ledges on upper frons: one. Sculpture of upper frons: with polygonal cells bearing setae without interstices. Sculpture of lower frons: dorsally and laterally with polygonal cells bearing setae, medially smooth with transverse carinae. Interantennal process: 1.3 × as long as wide, smooth with setigerous punctae medially. Transverse carina above interantennal process: with acute medial notch. Area ventral to transverse carina above interantennal process: with setigerous punctae. Sculpture on vertex: anteriorly with polygonal cells bearing setae, followed by an uneven transverse carina and posteriorly smooth with setigerous punctae; smooth area present around anterior ocellus; smooth area present on anterior and posterior margins of lateral ocellus. Sculpture of posterior orbital furrow: entirely foveate except for large depressions medially. Genal carina: absent. Sculpture of gena: with foveae and polygonal cells bearing dense white setae. Sculpture on A1: densely setigerous punctate. A1: 4.1 × as long as wide. Length of A3: 0.6 × A1 and 2.6 × A2.

#### Mesosoma

Sculpture of dorsal pronotum: with setigerous depressions. L: W of mesoscutum: 84.4:85.5. Sculpture of mesoscutum: smooth with setigerous punctae and foveae and a short ligula anteromedially. Notaulus: present. Sculpture of notaulus: foveate. Mesoscutal humeral sulcus: foveate. Mesoscutal suprahumeral sulcus: foveate. Parapsidal line: indicated as a furrow. Scutoscutellar sulcus: foveate. L: W of mesoscutellum: 40.0:63.3. Sculpture of mesoscutellum: with compact polygonal cells bearing setae and an incomplete transverse furrow anteriorly. Sculpture of dorsellum: anteriorly with large foveae, posteriorly smooth, posterior margin rounded. Sculpture of outer lateral propodeal area: anteriorly densely setose and posteriorly smooth. Sculpture of inner lateral propodeal area: anteriorly setose, posteriorly smooth. Lateral propodeal carina: anterior half arched inwards and posterior half oblique. Posterior propodeal projection: pointed, extending beyond anterior margin of T1. Sculpture of metasomal depression: setose with transverse furrows. Plical area: anteriorly densely setose posteriorly with uneven depressions with sparse setae. Sculpture of propleuron: anteriorly smooth, posteriorly foveate. Sculpture of lateral pronotal area: smooth. Posterior pronotal sulcus: with irregular cells. Pronotal cervical sulcus: foveae indicated anteriorly, remainder smooth. Speculum of mesopleuron: transversely carinate, interspersed foveae bearing dense setae. Postacetabular sulcus: foveate. Prespecular sulcus: foveate. Mesepimeral sulcus: foveate. Posterior mesepimeral area: smooth, narrower than mesepimeral sulcus. Mesopleural carina: indicated anteriorly with foveae on dorsal margin. Sculpture of femoral depression: smooth. Mesopleural pit: present. Sculpture of ventral mesopleuron: anteriorly with polygonal cells, remainder smooth with foveae, densely setose. Sculpture of metapleuron: dorsal metapleural area smooth with dense setae on anterior margin; ventral metapleural area dorsally smooth and ventrally with a row of foveae bearing brown setae followed by dense polygonal cells bearing white setae. Metapleural sulcus: foveate, medially indicated as a furrow. Paracoxal sulcus: indicated by large cells. Metapleural epicoxal sulcus: sculpture hidden by dense setae.

#### Fore wing

L: W: 359.2:131.4. Transparency: strongly infuscate. Lengths of R: R1: r-rs in ratio of 160:86:48. R: basally closer and distally distant from anterior margin of wing. Projection on anterior margin of fore wing: downcurved prior to R1.

#### Metasoma

L: W of metasoma: 272.9:98.8 Ratio of length of T1: T2: T3: T4: T5: 47.1:49.4:50.0:49.4:44.7. Anterior margin of T1: straight. Sculpture of T1: medially with longitudinally ribbed costae, laterally smooth with punctae bearing long setae and posteromedially smooth. Sculpture of T2: basal foveae present, followed by longitudinally ribbed costae medially; laterally longitudinally costate with sparse setigerous punctae, posteriorly smooth. Sculpture of T3: same as T2. Sculpture of T4: same as T2 except for medial smooth area with setigerous punctae. Sculpture of T5: smooth setigerous punctae interspersed with short longitudinal costae submedially on anterior margin. Sculpture of T6: smooth with setigerous punctae.

#### Male

Similar to male.

#### Etymology

This species is named ‘Rupavati’ after a *ragam* or melodic structure in South Indian (Carnatic) classical music meaning ‘the beautiful one’.

### *Sparasion salagami* Veenakumari sp. n. (Figs. 9A–F, 33M, 36L, 44E)

urn:lsid:zoobank.org:act:EDD84CDE-BDDE-46A9-A994-97333B55B574

#### Diagnosis

*Sparasion salagami* sp. n. is close to *S. syamalangi* sp. n. but differs from it in the following characters: in *S. salagami* sp. n. mesoscutum is sparsely foveate-punctate, scutoscutellar sulcus is foveate medially and outer lateral propodeal area is sparsely setose. Conversely, in *S. syamalangi* sp. n. mesoscutum is densely foveate-punctate, scutoscutellar sulcus is not foveate medially and outer lateral propodeal area is densely setose.

#### Material examined

Holotype: Female, (ICAR/NBAIR/P4615), **INDIA**: Goa: Carambolim: Central Coastal Agriculture Research Institute (CCARI), Krishi Vigyan Kendra (KVK), 15°29′53ʺN 73°55′25ʺE, 15 m, YPT, 15.VII.2015. Paratypes: 3 females, (ICAR/NBAIR/P4616–P4618), Goa: Carambolim, CCARI, KVK, 15°29′53ʺN 73°55′25ʺE, 15 m, YPT, 14–16.VII.2015.

### Description

Female body length = 5.54–5.81 mm (*n* = 4).

#### Colour

Head, mesosoma and metasoma steel blue; tegula black; fore coxa black-brown, meso and meta coxae blue-black, remainder of all legs black-brown; radicle, basal and apical A1 and apical A2 yellow–brown; remainder of A1 and A2 and other antennomeres black-brown; mandibles entirely black-brown.

#### Head

1.3 × as wide as high, 1.1 × as high as long. Setation on head: dense. IOS: 0.5 × head width, 0.9 × eye length. POL > LOL > OOL: 25.1:19.2:6.4. OOL: 0.5 × MOD. Compound eye: (L: W = 62.4:53.6). Setation of compound eye: sparsely setose. Anterior margin of frons: arcuate with deep indentation medially. Distance from level of anterior margin of compound eyes to anterior extension of frons: subequal to MOD. Number of transverse ledges on upper frons: one. Sculpture of upper frons: with circular cells bearing setae with smooth interstices. Sculpture of lower frons: entirely with polygonal cells except for transverse carinae posteromedially. Interantennal process: subequal in length and width, smooth. Transverse carina above interantennal process: with an acute medial notch. Area ventral to transverse carina above interantennal process: with setigerous punctae except for smooth area on inner margin. Sculpture on vertex: anteriorly with circular cells with smooth interstices, followed by polygonal cells bearing setae and an uneven transverse carina and posteriorly smooth with setigerous punctae; smooth area present around anterior ocellus; an irregular smooth area present posterior to lateral ocellus. Sculpture of posterior orbital furrow: foveate, except for depressions medially. Genal carina: absent. Sculpture of gena: with setigerous foveae and punctae, setae white, dense. Sculpture on A1: densely setigerous punctate. A1: 4.1 × as long as wide. Length of A3: 0.7 × A1 and 3.4 × A2.

#### Mesosoma

Sculpture of dorsal pronotum: with setigerous depressions. L: W of mesoscutum: 81.7:89.0. Sculpture of mesoscutum: smooth with sparse setigerous punctae and foveae. Notaulus: present. Sculpture of notaulus: foveate. Mesoscutal humeral sulcus: with large depressions. Mesoscutal suprahumeral sulcus: with large depressions. Parapsidal line: indicated as furrow. Scutoscutellar sulcus: foveate. L: W of mesoscutellum: 40.2:62.2. Sculpture of mesoscutellum: with compact polygonal cells bearing setae and an incomplete transverse furrow anteriorly. Sculpture of dorsellum: anteriorly foveate, with a pair of medial vertical carinae, posteriorly with depressions, posterior margin rounded. Sculpture of outer lateral propodeal area: anteriorly densely setose and posteriorly smooth. Sculpture of inner lateral propodeal area: anteriorly densely setose, posteriorly smooth with depressions. Lateral propodeal carina: anteriorly oblique and posteriorly arched. Posterior propodeal projection: rounded, not extending to anterior margin of T1. Sculpture of metasomal depression: sparsely setose. Plical area: anteriorly densely setose, posteriorly with smooth depressions. Sculpture of propleuron: anteriorly smooth, posteriorly foveate. Sculpture of lateral pronotal area: smooth. Posterior pronotal sulcus: with irregular cells. Pronotal cervical sulcus: foveate posteriorly. Speculum of mesopleuron: transversely carinate, interspersed with foveae bearing dense setae. Postacetabular sulcus: indicated as a furrow. Prespecular sulcus: foveate. Mesepimeral sulcus: foveate. Posterior mesepimeral area: smooth, narrower than mesepimeral sulcus. Mesopleural carina: indicated anteriorly with foveae on dorsal margin. Sculpture of femoral depression: smooth. Mesopleural pit: present. Sculpture of ventral mesopleuron: anteriorly with polygonal cells with dense setae, remainder smooth with sparse foveae, densely setose. Sculpture of metapleuron: dorsal metapleural area smooth with dense setae on anterior margin; ventral metapleural area dorsally smooth and ventrally with a row of foveae bearing brown setae followed by dense polygonal cells bearing white setae. Metapleural sulcus: foveate, medially indicated by a furrow. Paracoxal sulcus: indicated by polygonal cells with setae. Metapleural epicoxal sulcus: densely setose.

#### Fore wing

L: W: 314.1:115.4. Transparency: strongly infuscate. Lengths of R: R1: r-rs in ratio of 138:77:34. R: basally closer and distally distant from anterior margin of wing. Anterior margin of fore wing: downcurved both basally and prior to R1.

#### Metasoma

L: W of metasoma: 258.1:215.0. Ratio of length of T1: T2: T3: T4: T5: 44.1:46.5:47.6:47.6:41.2. Anterior margin of T1: weakly convex. Sculpture of T1: medially with longitudinally ribbed costae, laterally smooth with setigerous punctae. Sculpture of T2: basal foveae present, followed by longitudinal ribbed costae medially; laterally smooth with setigerous punctae, posteromedially smooth. Sculpture of T3: same as T2 except for posteromedial smooth patch with setigerous punctae. Sculpture of T4: same as T3 except for a wider smooth patch medially with setigerous punctae. Sculpture of T5: same as T4, except for shorter costae submedially on anterior margin. Sculpture of T6: smooth with setigerous punctae.

#### Male

Unknown.

#### Etymology

This species is named ‘Salagam’ after a simple melodic structure or *ragam* in Carnatic (South Indian) classical music.

### *Sparasion shulini* Veenakumari sp. n. (Figs. 10A–F, 36M, 44F, 46A)

**Fig. 46:**
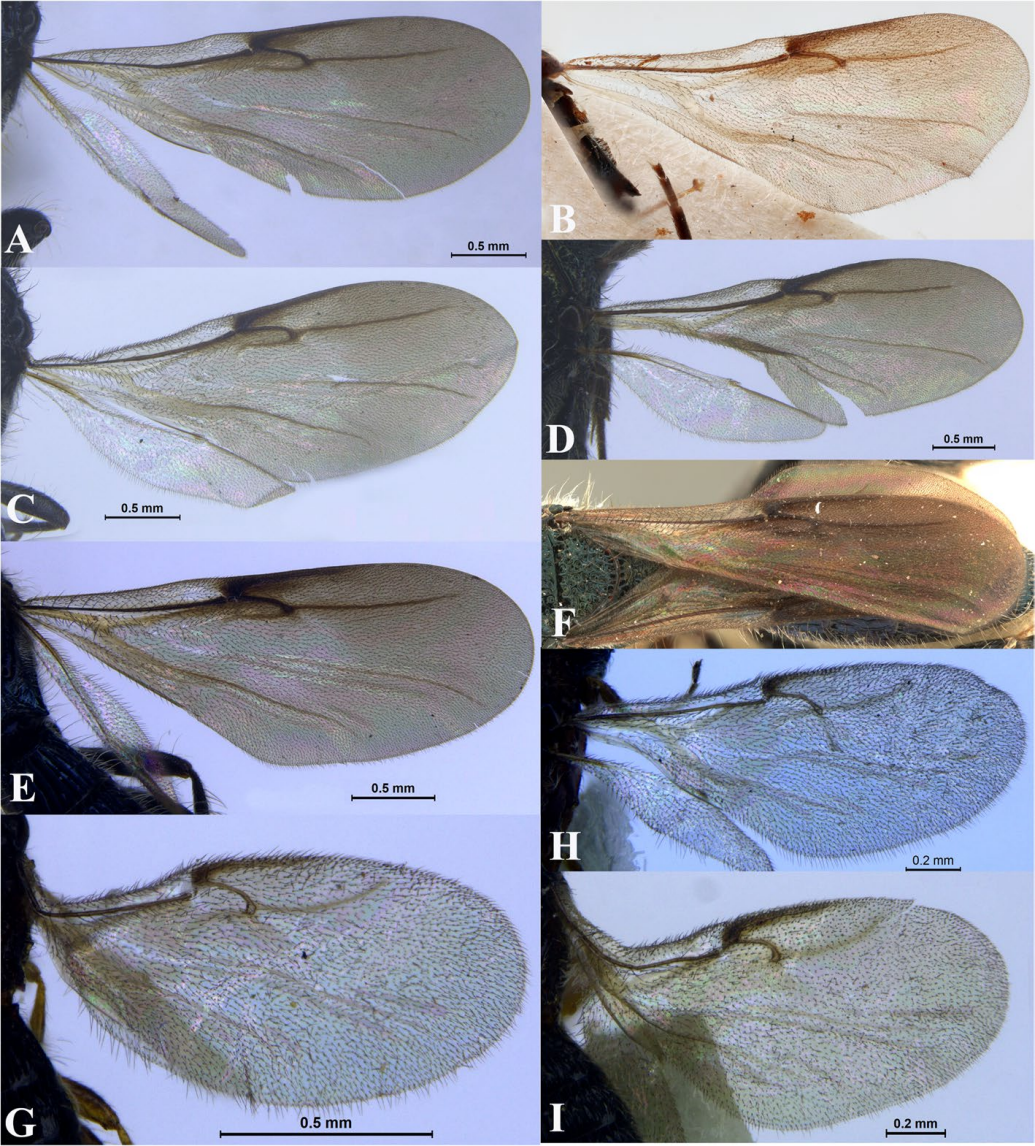
Wings. **A ***Sparasion shulini. ***B ***S. sinensis*. **C ***S. sivaranjini. ***D ***S. syamalangi. ***E ***S. todi*. **F ***S. travancoricus*. **G ***S. vanaspati*. **H ***S. visvambari*. **I ***S. zeelafi*

urn:lsid:zoobank.org:act:F86520CA-4A59-4AEF-B69A-3C7200D65B97

#### Diagnosis

*Sparasion shulini* sp. n. is close to *S. todi* sp. n. but differs from it in the following characters: in *S. shulini* mesoscutum is sparsely foveate-punctate, lower gena is smooth with setigerous punctae and setae on speculum of mesopleuron are short and sparse. Conversely, in *S. todi* sp. n. mesoscutum is densely foveate, lower gena with setigerous foveae and setae on speculum of mesopleuron are dense and elongate.

#### Material examined

Holotype: Female, (ICAR/NBAIR/P4599), **INDIA**: Tamil Nadu: Yercaud, Horticulture Research Station (HRS), 11°47′44ʺN 78°12′42ʺE, 1399 m, YPT, 06.VIII.2016.

### Description

Female body length = 5.93 mm (*n* = 1).

#### Colour

Head, mesosoma and metasoma steel blue; tegula black; fore coxa black, meso and meta coxae steel blue, remainder of legs black-brown; radicle and basal and apical A1 red-brown, remainder of A1 black, A2 apically red-brown, remainder black, remaining antennomeres black-brown; mandibles red-brown, with teeth dark brown.

#### Head

1.3 × as wide as high, 1.1 × as high as long. Setation on head: dense. IOS: 0.4 × head width, 0.9 × eye length. POL > LOL > OOL: 29.2:18.3:6.5. OOL: 0.4 × MOD. Compound eye: (L: W = 66.7:60.0). Setation of compound eye: glabrous. Anterior margin of frons: arcuate without medial indentation. Distance from level of anterior margin of compound eyes to anterior extension of frons in dorsal view: subequal to MOD. Number of transverse ledges on upper frons: one. Sculpture of upper frons: with large polygonal cells anteriorly without interstices and posteriorly with small circular cells with narrow interstices. Sculpture of lower frons: dorsally with setigerous foveae and laterally with polygonal cells bearing setae, medially smooth with transverse carinae. Interantennal process: 1.2 × as long as wide, smooth. Transverse carina above interantennal process: with acute medial notch. Area ventral to transverse carina above interantennal process: with setigerous punctae. Sculpture on vertex: anteriorly with small circular cells bearing setae with smooth interstices, posteriorly smooth with setigerous punctae; smooth area present around anterior ocellus; smooth area present on anterior and posterior margins of lateral ocellus. Sculpture of posterior orbital furrow: dorsally foveate and ventrally with depressions. Genal carina: absent. Sculpture of gena: smooth with setigerous foveae and punctae, setae dense. Sculpture on A1: densely setigerous punctate. A1: 4.6 × as long as wide. Length of A3: 0.5 × A1 and 2.2 × A2.

#### Mesosoma

Sculpture of dorsal pronotum: with setigerous depressions. L: W of mesoscutum: 85.1:91.4. Sculpture of mesoscutum: smooth with sparse setigerous punctae and foveae, a short ligula present anteromedially. Notaulus: present. Sculpture of notaulus: foveate. Mesoscutal humeral sulcus: foveate. Mesoscutal suprahumeral sulcus: foveate. Parapsidal line: indicated as a furrow. Scutoscutellar sulcus: foveate. L: W of mesoscutellum: 42.5:62.7. Sculpture of mesoscutellum: with compact polygonal cells bearing setae interspersed with incomplete longitudinal carinae and an incomplete transverse furrow anteriorly. Sculpture of dorsellum: anteriorly foveate, posteriorly almost smooth, posterior margin straight. Sculpture of outer lateral propodeal area: anteriorly densely setigerous punctate and posteriorly smooth with shallow depressions. Sculpture of inner lateral propodeal area: anteriorly pilose, posteriorly smooth. Lateral propodeal carina: anteriorly oblique and posteriorly arched. Posterior propodeal projection: pointed, extending on to anterior margin of T1. Sculpture of metasomal depression: setose with a medial transverse furrow. Plical area: anteriorly densely setose medially smooth and posteriorly with shallow depressions, sparsely setose. Sculpture of propleuron: anteriorly smooth, posteriorly with uneven carinae. Sculpture of lateral pronotal area: smooth with sparse setigerous punctae posteriorly. Posterior pronotal sulcus: with wide cells. Pronotal cervical sulcus: foveate posteriorly. Speculum of mesopleuron: transversely carinate, interspersed with foveae, sparsely setose. Postacetabular sulcus: foveate. Prespecular sulcus: foveate. Mesepimeral sulcus: foveate. Posterior mesepimeral area: smooth, narrower than mesepimeral sulcus. Mesopleural carina: indicated anteriorly with foveae on dorsal margin. Sculpture of femoral depression: smooth. Mesopleural pit: present. Sculpture of ventral mesopleuron: anteriorly with polygonal cells and dense setae, remainder smooth with sparse setigerous punctae. Sculpture of metapleuron: dorsal metapleural area smooth with sparse setae on anterior margin; ventral metapleural area dorsally smooth and ventrally with a row of foveae bearing brown setae followed by dense polygonal cells bearing white setae. Metapleural sulcus: foveate, medially indicated as a furrow. Paracoxal sulcus: indicated with wide depressions. Metapleural epicoxal sulcus: densely setose hiding the sculpture.

#### Fore wing

L: W: 332.5:121.3. Transparency: strongly infuscate. Lengths of R: R1: r-rs in ratio of 154:74:41. R: gradually distant from anterior margin of wing. Anterior margin of fore wing: with a slight downcurve prior to R1.

#### Metasoma

L: W of metasoma: 258.5:91.4. Ratio of length of T1: T2: T3: T4: T5: 45.7:47.9:47.9:46.8:42.5. Anterior margin of T1: straight. Sculpture of T1: medially with longitudinally ribbed costae, laterally smooth with shallow depressions bearing setigerous punctae and posteromedially smooth. Sculpture of T2: basal foveae present, followed by longitudinally ribbed costae medially; laterally smooth with setigerous foveae and posteriorly smooth. Sculpture of T3: same as T2. Sculpture of T4: same as T2 except for smooth area posteromedially with setigerous punctae. Sculpture of T5: same as T4. Sculpture of T6: smooth with setigerous punctae.

#### Male

Unknown.

#### Etymology

This species is named ‘Shulini’ after a *ragam* or melodic structure in South Indian (Carnatic) classical music, meaning ‘the spear wielding goddess’, referring to the Hindu goddess *Durga*.

### *Sparasion sinensis* Walker (Figs. 11A–F, 19, 36N, 46B)

*Sparasion Sinense* Walker, 1852: 46.

*Sparasion sinense*: Dodd, 1920: 343. Diagnosis.

*Sparasion sinense*: Kieffer, 1926: 293. Description.

#### Diagnosis

*Sparasion sinensis* sp. n. shares the character state – elongate and narrow posterior propodeal projection which extends onto anterior margin of T1 and a sinuous lateral propodeal carina – with *S. pahadi.* The distinguishing characters are given above under the latter species.

#### Material examined

Holotype, female (B.M.TYPE HYM. 9.548), **CHINA**: Fou-Chou-Fou (inferred by us to be Fuzhou, Fujian) (Walker, 1852).

### Description

Female body length = 8.8 mm.

#### Colour

Head, mesosoma and metasoma steel blue-black; tegula brown-black; all legs brown-black; radicle brown, remaining antennomeres red-brown; mandible brown-black.

#### Head

Setation on head: dense. Anterior margin of frons: arcuate. Distance from level of anterior margin of compound eyes to anterior extension of frons in dorsal view: < MOD. Number of transverse ledges on upper frons: one. Sculpture of upper frons: with circular cells bearing setae. Sculpture of lower frons: with polygonal and circular cells bearing setae. Sculpture on vertex: anteriorly with circular cells, followed by polygonal cells and posteriorly smooth with punctae; a smooth area present around anterior ocellus; a smooth triangular area present posterior to lateral ocellus. Sculpture of posterior orbital furrow: foveate. Genal carina: absent. Sculpture of gena: dorsally with polygonal cells and ventrally smooth, sparsely setose. Sculpture on A1: densely setigerous punctate.

#### Mesosoma

Sculpture of dorsal pronotum: smooth with foveae dorsally and ventrally with large irregular depressions (when viewed laterally). Sculpture of mesoscutum: smooth with sparse setigerous punctae and foveae. Mesoscutal humeral sulcus: indicated as a furrow. Mesoscutal suprahumeral sulcus: foveate. Parapsidal line: indicated as furrow. Sculpture of outer lateral propodeal area: densely setose and smooth posteriorly. Sculpture of inner lateral propodeal area: smooth, with two medial transverse carinae and anteriorly setose. Posterior propodeal projection: pointed, extending onto anterior margin of T1. Plical area: anteriorly densely setose and posteriorly with large shallow depressions. Sculpture of propleuron: anteriorly smooth, posteriorly foveate. Sculpture of lateral pronotal area: anteriorly smooth and posteriorly foveate-punctate. Posterior pronotal sulcus: with depressions. Pronotal cervical sulcus: anteriorly foveate. Speculum of mesopleuron: transversely carinate, interspersed with foveae, densely setose. Postacetabular sulcus: foveate. Prespecular sulcus: with wide depressions. Mesepimeral sulcus: foveate. Mesepimeral area: smooth, narrower than mesepimeral sulcus. Mesopleural carina: indicated anteriorly with shallow foveae on dorsal margin. Sculpture of femoral depression: smooth. Mesopleural pit: present. Sculpture of ventral mesopleuron: anteriorly with polygonal cells remainder smooth with sparse setigerous foveae. Sculpture of metapleuron: dorsal metapleural area smooth with dense long setae on anterior margin; ventral metapleural area dorsally smooth and ventrally with a row of foveae and remainder with large shallow polygonal cells bearing brown and white setae. Metapleural sulcus: foveate, medially indicated as a furrow. Paracoxal sulcus: foveate. Metapleural epicoxal sulcus: foveate, densely setose.

#### Fore wing

L: W: 303:121. Transparency: weakly infuscate. Lengths of R: R1: r-rs in ratio of 146:80:35. R: distant from anterior margin of wing. Anterior margin of wing: with downcurve prior to R1.

#### Metasoma

L: W of metasoma: 238:77. Ratio of length of T1: T2: T3: T4: T5: 44:44:44:44:39. Anterior margin of T1: straight. Sculpture of T1: longitudinally ribbed costate, laterally with setigerous punctae and posteriorly smooth. Sculpture of T2: basal foveae present, followed by longitudinal ribbed costae; laterally smooth with setigerous punctae and posteriorly smooth. Sculpture of T3: same as T2, with a small smooth area with setae posteromedially. Sculpture of T4: same as T3 except for a larger smooth area with setae posteromedially. Sculpture of T5: basal foveae present, with shorter longitudinal costae sublaterally, remainder smooth with setigerous punctae. Sculpture of T6: smooth with setigerous punctae.

#### Male

Unknown.

#### Remarks

Holotype present at NHM. *Sparasion* is misspelt as ‘*Sparaison*’ on the type label. Mesosoma is broken. The holotype is a female, incorrectly mentioned as a male [11, 21].

### *Sparasion sivaranjini* Veenakumari sp. n. (Figs. 12A–F, 36O, 44G, 46C)

urn:lsid:zoobank.org:act:90446A25-6FEB-4F59-B044-394E134AB84A

#### Diagnosis

*Sparasion sivaranjini* sp. n. is close to *S. rupavati* sp. n. The distinguishing characters are given above under the latter species.

#### Material examined

Holotype: Female, (ICAR/NBAIR/P4551), **INDIA**: Tamil Nadu: Coimbatore: Valparai: Urulikkal, 10°19′47ʺN 76°53′32ʺE, 1068 m, YPT, 04.V.2014. Paratypes: 9 females, (ICAR/NBAIR/P4552–P4560), Tamil Nadu: Coimbatore: Valparai: Urulikkal, 10°19′47ʺN 76°53′32ʺE, 1068 m, YPT, 04.V.2014.

### Description

Female body length = 6.03–6.46 mm (*n* = 10).

#### Colour

Head and mesosoma steel blue-green, T1 steel blue-green, remaining tergites steel blue; tegula blue-black; fore coxa brown, meso and meta coxae steel blue, remainder of all legs black-brown; radicle, basal A1 red-brown, remaining antennomeres black-brown; mandible brown-black.

#### Head

1.4 × as wide as high, 0.9 × as high as long. Setation on head: dense. IOS: 0.4 × head width, 0.7 × eye length. POL > LOL > OOL: 20.8:15.1:3.8. OOL: 0.3 × MOD. Compound eye: (L: W = 75.0:60.0). Setation of compound eye: sparsely setose. Anterior margin of frons: arcuate with medial indentation. Distance from level of anterior margin of compound eyes to anterior extension of frons in dorsal view: 0.8 × MOD. Number of transverse ledges on upper frons: one. Sculpture of upper frons: with circular cells bearing setae, with smooth interstices. Sculpture of lower frons: medially transversely carinate with ellipsoidal depressions between the carinae which progressively diminishes in width anteriad; remainder with polygonal cells bearing setae. Interantennal process: subequal in length and width, smooth. Transverse carina above interantennal process: with an acute medial notch. Area ventral to transverse carina above interantennal process: with setigerous punctae except for smooth area on inner margin. Sculpture on vertex: anteriorly with polygonal cells followed by an uneven transverse carina and posteriorly smooth with setigerous punctae; a smooth area present around anterior ocellus; a smooth triangular area present posterior to lateral ocellus. Sculpture of posterior orbital furrow: dorsally foveate and ventrally with polygonal cells. Genal carina: absent. Sculpture of gena: smooth with setigerous foveae and punctae, densely setose. Sculpture on A1: densely setigerous punctate. A1: 4.8 × as long as wide. Length of A3: 0.5 × A1 and 2.8 × A2.

#### Mesosoma

Sculpture of dorsal pronotum: with setigerous depressions. L: W of mesoscutum: 86.0:102.2. Sculpture of mesoscutum: weakly rugulose with dense punctae anteromedially, remainder with sparse setigerous foveae with punctae; a ligula present anteromedially. Notaulus: present. Sculpture of notaulus: foveate. Mesoscutal humeral sulcus: foveate. Mesoscutal suprahumeral sulcus: foveate. Parapsidal line: indicated as furrow. Scutoscutellar sulcus: foveate. L: W of mesoscutellum: 40.2:64.3. Sculpture of mesoscutellum: with compact polygonal cells, and an incomplete transverse furrow anteriorly. Sculpture of dorsellum: anteriorly foveate, posteriorly smooth with small uneven foveae, posterior margin almost straight. Sculpture of outer lateral propodeal area: anteriorly densely setose posteriorly smooth. Sculpture of inner lateral propodeal area: anteriorly setose and posteriorly almost smooth. Lateral propodeal carina: sinuous. Posterior propodeal projection: pointed, extending onto anterior margin of T1. Sculpture of metasomal depression: densely setose. Plical area: anteriorly densely setose concealing the sculpture and posteriorly almost smooth. Sculpture of propleuron: anteriorly smooth, posteriorly foveate. Sculpture of lateral pronotal area: weakly rugulose. Posterior pronotal sulcus: with ovoid cells. Pronotal cervical sulcus: not foveate. Speculum of mesopleuron: transversely carinate, interspersed with foveae, densely setose. Postacetabular sulcus: foveate. Prespecular sulcus: foveate. Mesepimeral sulcus: foveate. Posterior mesepimeral area: smooth, narrower than mesepimeral sulcus. Mesopleural carina: indicated anteriorly with polygonal cells on dorsal margin. Sculpture of femoral depression: smooth. Mesopleural pit: present. Sculpture of ventral mesopleuron: anteriorly with polygonal cells with dense setae, remainder smooth with sparse setigerous foveae. Sculpture of metapleuron: dorsal metapleural area rugulose with dense long setae on anterior margin; ventral metapleural area dorsally rugulose and ventrally with a row of foveae bearing brown setae followed by dense polygonal cells bearing white setae. Metapleural sulcus: foveate, medially indicated as a furrow. Paracoxal sulcus: with depressions. Metapleural epicoxal sulcus: densely setose hiding the sculpture.

#### Fore wing

L: W: 313.3:125.2. Transparency: strongly infuscate. Lengths of R: R1: r-rs in ratio of 149:85:40. R: basally closer and gradually distant from anterior margin of wing. Anterior margin of wing: upcurved basally and a weak downcurve prior to R1.

#### Metasoma

L: W of metasoma: 259.4:112.4. Ratio of length of T1: T2: T3: T4: T5: 43.2:48.3:48.6:48.6:42.1. Anterior margin of T1: weakly convex. Sculpture of T1: medially longitudinally ribbed costate, laterally smooth with setigerous punctae and posteriorly smooth. Sculpture of T2: basal foveae present, followed medially by longitudinal ribbed costae; laterally smooth with setigerous punctae and posteriorly smooth with sparse punctae. Sculpture of T3: same as T2. Sculpture of T4: same as T2 except for smooth area with setae posteromedially. Sculpture of T5: basal foveae present, sparsely costate with setigerous punctae. Sculpture of T6: smooth with setigerous punctae.

#### Male

Unknown.

#### Etymology

This species is named ‘Sivaranjini’ after a *ragam* or melodic structure in both South Indian and North Indian classical music which is sung in the late evenings to propitiate *Siva*, the fearsome Hindu god of destruction.

### *Sparasion syamalangi* Veenakumari sp. n. (Figs. 13A–F, 44H, 46D, 47A)

**Fig. 47:**
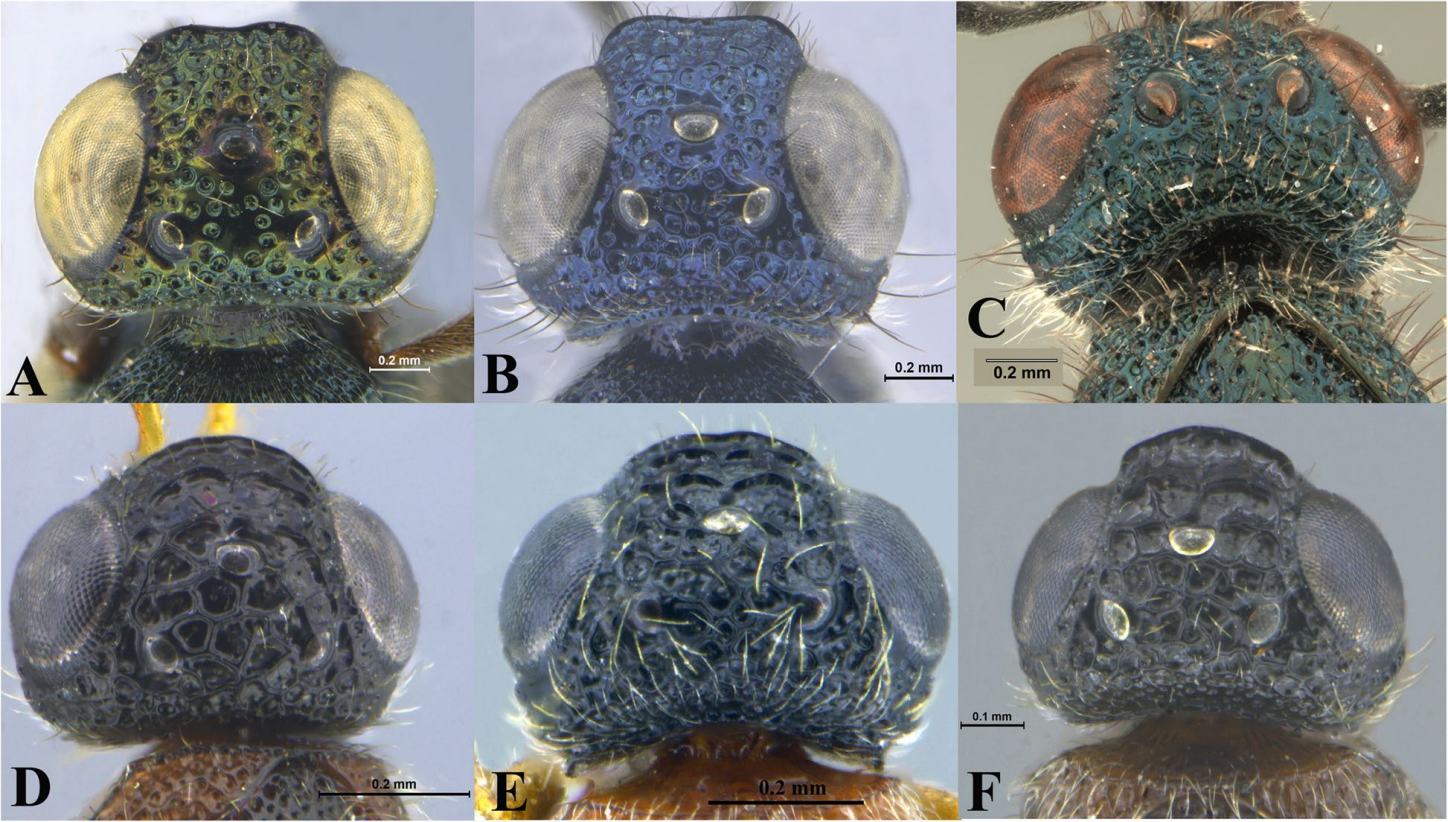
Head, dorsal view. **A*** Sparasion syamalangi. ***B ***S. todi*. **C ***S. travancoricus*. **D ***S. vanaspati*. **E ***S. visvambari*. **F ***S. zeelafi*

urn:lsid:zoobank.org:act:B97FC63E-548E-47AD-88A3-D9445F423491

#### Diagnosis

*Sparasion syamalangi* sp. n. is close to *S. salagami* sp. n. The distinguishing characters are given above under the latter species.

#### Material examined

Holotype: Female, (ICAR/NBAIR/P4600), **INDIA**: Tamil Nadu: Tiruchirappalli, Kannapadi, 11°07′02ʺN 78°42′55ʺE, 120 m, YPT, 23.III.2013.

### Description

Female body length = 6.23 mm, (*n* = 1).

#### Colour

Head and mesosoma steel green, metasoma steel blue; tegula brown; fore coxa black-brown, meso and meta coxae steel green–blue, remainder of legs black-brown; radicle, basal and apical A1, apical A2 red-brown, remainder of A1 and A2 dark brown, remaining antennomeres black-brown; mandibles red-brown with teeth dark brown.

#### Head

1.3 × as wide as high, as high as long. Setation on head: dense. IOS: 0.4 × head width, 0.8 × eye length. POL > LOL > OOL: 36.7:29.1:6.7. OOL: 0.4 × MOD. Compound eye: (L: W = 78.1:58.6). Setation of compound eye: sparsely setose. Anterior margin of frons: arcuate with a medial indentation. Distance from level of anterior margin of compound eyes to anterior extension of frons: 1.1 × MOD. Number of transverse ledges on upper frons: one. Sculpture of upper frons: with circular cells bearing setae and with smooth interstices. Sculpture of lower frons: dorsally and laterally with circular cells bearing setae, medially smooth with convex carinae ventrad. Interantennal process: 1.1 × as long as wide, smooth. Transverse carina above interantennal process: with acute medial notch. Area ventral to transverse carina above interantennal process: with setigerous foveae. Sculpture on vertex: anteriorly smooth with sparse circular cells, followed by an uneven transverse carina and a row of irregular cells bearing setae, posteriorly smooth with setigerous punctae; smooth area present around anterior ocellus; smooth area present around lateral ocellus. Sculpture of posterior orbital furrow: foveate. Genal carina: absent. Sculpture of gena: setigerous foveate, setae dense and white. Sculpture on A1: densely setigerous punctate. A1: 4.1 × as long as wide. Length of A3: 0.6 × A1 and 2.5 × A2.

#### Mesosoma

Sculpture of dorsal pronotum: with setigerous foveae and depressions. L: W of mesoscutum: 90.0:95.5. Sculpture of mesoscutum: smooth with punctae and foveae and a ligula present anteromedially. Notaulus: present. Sculpture of notaulus: foveate. Mesoscutal humeral sulcus: foveate. Mesoscutal suprahumeral sulcus: foveate; Parapsidal line: indicated as a furrow. Scutoscutellar sulcus: foveate only laterally. L: W of mesoscutellum: 38.8:67.7. Sculpture of mesoscutellum: with compact polygonal cells with setae and an incomplete transverse furrow anteriorly. Sculpture of dorsellum: foveate, posterior margin weakly projecting medially. Sculpture of outer lateral propodeal area: densely setose. Sculpture of inner lateral propodeal area: anteriorly densely foveate, posteriorly with shallow depressions. Lateral propodeal carina: anteriorly almost straight and posteriorly arched. Posterior propodeal projection: rounded, extending onto anterior margin of T1. Sculpture of metasomal depression: setose. Plical area: densely setose concealing sculpture. Sculpture of propleuron: anteriorly smooth, posteriorly foveate. Sculpture of lateral pronotal area: smooth. Posterior pronotal sulcus: with irregular cells. Pronotal cervical sulcus: not foveate. Speculum of mesopleuron: transversely carinate, interspersed foveae, densely setose. Postacetabular sulcus: foveate. Prespecular sulcus: foveate. Mesepimeral sulcus: foveate. Posterior mesepimeral area: smooth, narrower than mesepimeral sulcus. Mesopleural carina: indicated as a short carina anteriorly with foveae on dorsal margin. Sculpture of femoral depression: smooth. Mesopleural pit: not distinct. Sculpture of ventral mesopleuron: anteriorly with polygonal cells and dense setae, remainder smooth with setigerous foveae and punctae. Sculpture of metapleuron: dorsal metapleural area smooth with dense setae on anterior margin; ventral metapleural area dorsally smooth and ventrally with a row of foveae bearing brown setae followed by dense polygonal cells bearing white setae. Metapleural sulcus: foveate, medially indicated as a furrow. Paracoxal sulcus: with irregular cells. Metapleural epicoxal sulcus: dense setae concealing the sculpture.

#### Fore wing

L: W: 344.7:125. Transparency: strongly infuscate. Lengths of R: R1: r-rs in ratio of 159:95:38. R: gradually distant from anterior margin of wing. Anterior margin of fore wing: with a slight downcurve prior to R1.

#### Metasoma

L: W of metasoma: 257.6:107.6 Ratio of length of T1: T2: T3: T4: T5: 40.4:47.8:48.9:50.5:42.6. Anterior margin of T1: weakly convex. Sculpture of T1: longitudinally ribbed costae, laterally with foveae bearing setae, posteriorly smooth. Sculpture of T2: basal foveae present, followed by longitudinally ribbed costae medially; laterally with shallow foveae with setae, posteriorly smooth. Sculpture of T3: same as T2. Sculpture of T4: same as T2 except for medial smooth area with setigerous punctae. Sculpture of T5: small basal foveae present, remainder smooth with setigerous punctae except for weak longitudinal costae submedially on anterior margin. Sculpture of T6: smooth with setigerous punctae.

#### Male

Unknown.

#### Etymology

This species is named ‘Syamalangi’ after a *ragam* or melodic structure in South Indian (Carnatic) classical music meaning ‘she whose body is dark’.

### *Sparasion todi* Veenakumari sp. n. (Figs. 14A–F, 44I, 46E, 47B)

urn:lsid:zoobank.org:act:C6160AB4-18C7-40AB-943F-CF937A854B61

#### Diagnosis

*Sparasion todi* sp. n. is close to *S. shulini* sp. n. The distinguishing characters are given above under the latter species.

#### Material examined

Holotype: Female, (ICAR/NBAIR/P4611), **INDIA**: Tamil Nadu: Lower Pulney Hills, Thadiyankudisai, HRS, 10°17′58ʺN 77°42′42ʺE, 990 m, YPT, 07.XI.2014. Paratypes: 1 female, (ICAR/NBAIR/P4612), Tamil Nadu: Lower Pulney Hills, Thadiyankudisai, HRS, 10°17′58ʺN 77°42′42ʺE, 990 m, YPT, 07.XI.2014; 1 female (ICAR/NBAIR/P4613), Tamil Nadu: Lower Pulney Hills, Thadiyankudisai, HRS, 10°17′58ʺN 77°42′42ʺE, 990 m, YPT, 26.XI.2016.

### Description

Female body length = 5.39–5.54 mm (*n* = 3).

#### Colour

Head, mesosoma and metasoma steel blue; tegula black with brown patches; fore coxa brown, meso and meta coxae steel blue, remainder of all legs black-brown; radicle, basal and apical A1 and apical A2 yellow–brown; remainder of A1 and A2 and other antennomeres black-brown; mandibles entirely black-brown.

#### Head

1.2 × as wide as high, as high as long. Setation on head: dense. IOS: 0.5 × head width, subequal to eye length. POL > LOL > OOL: 27.7:20.4:6.5. OOL: 0.5 × MOD. Compound eye: (L: W = 61.3:54.3). Setation of compound eye: glabrous. Anterior margin of frons: arcuate without medial indentation. Distance from level of anterior margin of compound eyes to anterior extension of frons: 1.1 × MOD. Number of transverse ledges on upper frons: one. Sculpture of upper frons: with polygonal cells bearing setae. Sculpture of lower frons: with polygonal cells dorsally and laterally bearing setae, medially smooth with transverse carinae. Interantennal process: 1.3 × as long as wide, smooth with a medial longitudinal furrow. Transverse carina above interantennal process: with an acute medial notch. Area ventral to transverse carina above interantennal process: with setigerous punctae except for smooth area on inner margin. Sculpture on vertex: anteriorly with polygonal cells bearing setae, followed by an uneven transverse carina, posteriorly smooth with setigerous punctae; a narrow smooth area present around anterior ocellus; smooth area present on anterior and posterior margins of lateral ocellus. Sculpture of posterior orbital furrow: dorsally foveate ventrally with depressions. Genal carina: absent. Sculpture of gena: anteriorly with setigerous foveae and punctae, posteriorly smooth, setae dense. Sculpture on A1: densely setigerous punctate. A1: 3.8 × as long as wide. Length of A3: 0.6 × A1 and 2.2 × A2.

#### Mesosoma

Sculpture of dorsal pronotum: with setigerous depressions. L: W of mesoscutum: 80.1:87.8. Sculpture of mesoscutum: smooth with setigerous punctae and foveae. Notaulus: present. Sculpture of notaulus: foveate. Mesoscutal humeral sulcus: foveate. Mesoscutal suprahumeral sulcus: foveate. Parapsidal line: indicated as furrow. Scutoscutellar sulcus: foveate. L: W of mesoscutellum: 41.8:61.2. Sculpture of mesoscutellum: with compact polygonal cells bearing setae and an incomplete transverse furrow anteriorly. Sculpture of dorsellum: anteriorly foveate, posteriorly smooth with sparse foveae, posterior margin straight. Sculpture of outer lateral propodeal area: anteriorly densely setose, posteriorly smooth. Sculpture of inner lateral propodeal area: anteriorly densely setose, posteriorly smooth with shallow depressions. Lateral propodeal carina: anteriorly oblique, posteriorly sinuous. Posterior propodeal projection: pointed, not extending to anterior margin of T1. Sculpture of metasomal depression: densely setose. Plical area: anteriorly densely setose, posteriorly smooth with weak foveae. Sculpture of propleuron: anteriorly smooth, posteriorly weakly carinate. Sculpture of lateral pronotal area: smooth. Posterior pronotal sulcus: with irregular cells. Pronotal cervical sulcus: not foveate. Speculum of mesopleuron: with foveae bearing setae interspersed with sparse transverse carinae. Postacetabular sulcus: foveate. Prespecular sulcus: foveate. Mesepimeral sulcus: foveate. Posterior mesepimeral area: smooth, subequal in width of mesepimeral sulcus. Mesopleural carina: not distinct. Sculpture of femoral depression: smooth. Mesopleural pit: present. Sculpture of ventral mesopleuron: anteriorly with polygonal cells with dense setae, remainder smooth with setigerous foveae and punctae. Sculpture of metapleuron: dorsal metapleural area smooth with dense setae on anterior margin; ventral metapleural area dorsally smooth and ventrally with a row of foveae bearing brown setae followed by dense polygonal cells bearing white setae. Metapleural sulcus: foveate, medially indicated by a furrow. Paracoxal sulcus: indicated by polygonal cells. Metapleural epicoxal sulcus: densely setose.

#### Fore wing

L: W: 331.0:119.7. Transparency: strongly infuscate. Lengths of R: R1: r-rs in ratio of 135:80:41. R: gradually distant from anterior margin of wing. Anterior margin of fore wing: no downcurve prior to R1.

#### Metasoma

L: W of metasoma: 248.9:86.7. Ratio of length of T1: T2: T3: T4: T5: 42.2:44.4:44.4:46.7:41.1. Anterior margin of T1: weakly convex. Sculpture of T1: longitudinally ribbed costate medially, laterally smooth with setae. Sculpture of T2: basal foveae present, followed by longitudinal ribbed costae medially; laterally smooth with setigerous punctae, posteriorly smooth. Sculpture of T3: same as T2 except for a smooth patch medially with setigerous punctae. Sculpture of T4: same as T3 except for larger smooth patch medially. Sculpture of T5: same asT4. Sculpture of T6: smooth with setigerous punctae.

#### Male

Unknown.

#### Etymology

This species is named ‘Todi’, after the melodic structure or *raga* in Hindustani (North Indian classical) music which is to be sung in the morning.

### *Sparasion travancoricus* Mani & Sharma (Figs. 15A–D, 19, 46F, 47C)

*Sparasion travancoricus* Mani & Sharma, 1981: 443.

*Sparasion travncoricus*: Mani & Sharma, 1982: 157. Description, spelling error.

#### Diagnosis

*Sparasion travancoricus* is close to *S. meghmalhari* sp. n. The distinguishing characters are given above under the latter species.

#### Material examined

Holotype, female (USNMENT 01109920), **INDIA**: Kerala: Kottur, 06.IV.1980, leg. M. S. Mani & party, Teak-Sal Eco. Survey, School of Entomology, St. John’s College, Agra -282002, India.

### Description

Female body length = 6.0–6.2 mm.

#### Colour

Head, mesosoma and metasoma steel green-blue; tegula black; all coxae steel blue-green, remainder of legs brown-black with extremities brown-red; radicle, basal and apical A1, A2 red-brown, remainder of A1 and A2 and other antennomeres black-brown; mandible black-brown except for red-brown patch in apical 1/3.

#### Head

Setation on head: dense. Anterior margin of frons: arcuate with a medial indentation. Distance from level of anterior margin of compound eyes to anterior extension of frons in dorsal view: < MOD. Number of transverse ledges on upper frons: one. Sculpture of upper frons: with polygonal cells bearing setae. Sculpture of lower frons: with polygonal cells bearing setae. Sculpture on vertex: anteriorly smooth with small polygonal cells and foveae followed by larger polygonal cells with setae and posteriorly smooth with setigerous punctae; a smooth area present around anterior ocellus; a smooth area present posterior to lateral ocellus. Sculpture of posterior orbital furrow: dorsally foveate and ventrally with depressions. Genal carina: absent. Sculpture of gena: smooth with setigerous foveae and punctae, densely setose. Sculpture on A1: densely setigerous punctate.

#### Mesosoma

Sculpture of dorsal pronotum: with setigerous depressions with smooth interstices. L: W of mesoscutum: 85.0:113. Sculpture of mesoscutum: smooth with sparse foveae and punctae. Notaulus: present. Sculpture of notaulus: foveate. Mesoscutal humeral sulcus: foveate. Mesoscutal suprahumeral sulcus: foveate. Parapsidal line: indicated as furrow. Scutoscutellar sulcus: foveate. L: W of mesoscutellum: 57:84. Sculpture of mesoscutellum: with compact polygonal cells, and an incomplete transverse furrow anteriorly. Sculpture of dorsellum: anteriorly foveate, posteriorly smooth with small uneven foveae, posterior margin almost straight. Posterior propodeal projection: rounded, not extending on to anterior margin of T1. Sculpture of metasomal depression: setose. Plical area: anteriorly densely setose and posteriorly with large shallow depressions. Sculpture of propleuron: weakly foveate. Sculpture of lateral pronotal area: smooth with sparse setigerous punctae posteriorly. Posterior pronotal sulcus: foveate. Pronotal cervical sulcus: not foveate. Speculum of mesopleuron: setigerous punctate with sparse effaced transverse carinae, densely setose. Postacetabular sulcus: foveate. Prespecular sulcus: with wide foveae. Mesepimeral sulcus: foveate. Mesepimeral area: smooth, narrower than mesepimeral sulcus. Mesopleural carina: present with foveae on dorsal margin. Sculpture of femoral depression: smooth. Mesopleural pit: present. Sculpture of ventral mesopleuron: with dense setigerous foveae. Sculpture of metapleuron: dorsal metapleural area smooth with dense long setae on anterior margin; ventral metapleural area dorsally smooth and ventrally with polygonal cells bearing brown and white setae. Metapleural sulcus: foveate, medially indicated as a furrow. Paracoxal sulcus: foveate. Metapleural epicoxal sulcus: densely setose.

#### Fore wing

L: W: 400:130. Transparency: strongly infuscate. Lengths of R: R1: r-rs in ratio of 166:100:44. R: basally closer and progressively diverging from anterior margin of wing. Anterior margin of wing: with a weak downcurve prior to R1.

#### Metasoma

L: W of metasoma: 260:104. Sculpture of T1–T4: longitudinally costate, laterally smooth with setigerous punctae. Sculpture of T2–T4: basal foveae present, followed by longitudinal costae, laterally smooth with setigerous punctae. Sculpture of T5: basal foveae present, smooth with setigerous punctae and short longitudinal costae sublaterally on anterior margin. Sculpture of T6: smooth with setigerous punctae.

#### Male

Unknown.

#### Remarks

The holotype is present in USNM. Specimen is in good condition.

### *Sparasion vanaspati* Veenakumari sp. n. (Figs. 44J, 46G, 47D, 48A–F)

**Fig. 48:**
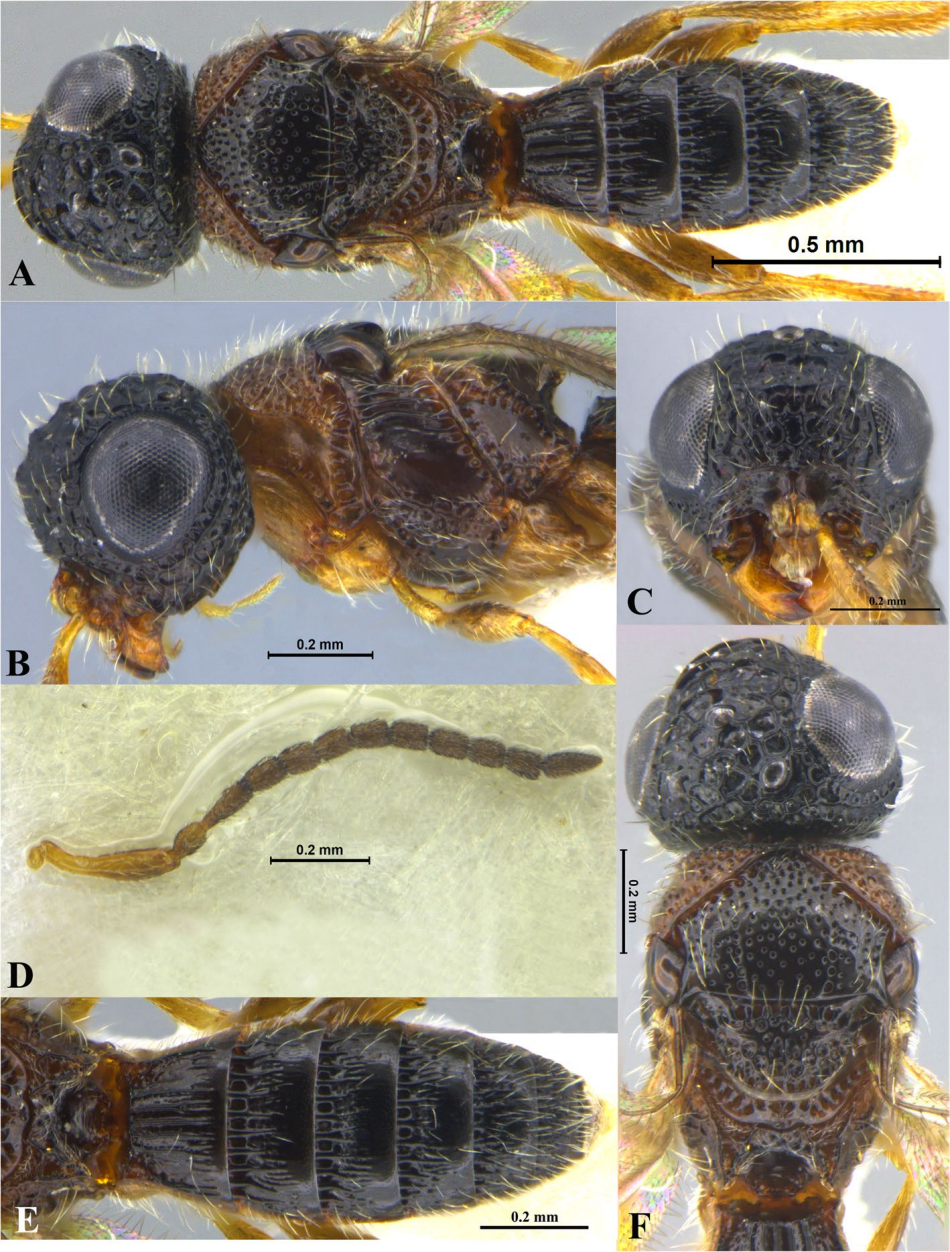
*Sparasion vanaspati ***sp. n**., male holotype. **A** Habitus, dorsal view. **B** Head and pleuron. **C** Frons. **D** Antenna. **E** Metasoma. **F** Head and mesonotum

urn:lsid:zoobank.org:act:E5D6B213-858F-4581-8CC8-B14D9FB69D9F

#### Diagnosis

This is the only species in the Oriental fauna of *Sparasion* with ovoid fore wing.

#### Material examined

Holotype: Female, (ICAR/NBAIR/P4628), **INDIA**: Arunachal Pradesh: Pasighat, CHF, YPT, 28°04′28ʺN 95°19′28ʺE, 173 m, YPT, 05.V.2014.

### Description

Male body length = 2.15 mm (*n* = 1).

#### Colour

Head black, mesoscutum, mesoscutellum and metasoma black-brown; pronotum, dorsellum, metanotal trough, lateral propodeal area brown-black; tegula dark brown with uneven light brown patches; legs orange-brown; lateral pronotal area brown, mesopleuron and metapleuron brown-black; radicle, A1–A2 brown-yellow, remaining antennomeres brown-black; mandibles orange-yellow with teeth dark brown.

#### Head

1.1 × as wide as high, 1.2 × as high as long. Setation on head: sparse. IOS: 0.5 × head width, 0.9 × eye length. POL > LOL > OOL: 18.8:8.2:5.2. OOL: 0.9 × MOD. Compound eye: (L: W = 29.7:25.4). Setation of compound eye: glabrous. Anterior margin of frons: arcuate. Distance from level of anterior margin of compound eyes to anterior extension of frons: 1.3 × MOD. Number of transverse ledges on upper frons: three. Sculpture of upper frons: anteriorly with a row of shallow polygonal cells followed by two rows of large polygonal cells. Sculpture of lower frons: with polygonal cells bearing setae. Interantennal process: 1.3 × as long as wide, smooth with medial furrow. Transverse carina above interantennal process: with a medial notch. Area ventral to transverse carina above interantennal process: setigerous punctae except smooth area on inner margin. Sculpture on vertex: anteriorly with large polygonal cells followed by smaller polygonal cells bearing setae, posteriorly smooth with setigerous punctae; a narrow smooth area present around anterior ocellus; a semicircular smooth area present posterior to lateral ocellus. Sculpture of posterior orbital furrow: dorsally foveate and ventrally with polygonal cells. Genal carina: present. Sculpture of gena: anteriorly with setigerous polygonal cells, posteriorly smooth with sparse white setigerous punctae. Sculpture on A1: smooth with sparse setae. A1: 4.7 × as long as wide. Length of A3: 0.4 × A1 and 1.6 × A2.

#### Mesosoma

Sculpture of dorsal pronotum: with depressions bearing setae. L: W of mesoscutum: 30.1:41.6. Sculpture of mesoscutum: smooth with setigerous foveae. Notaulus: present. Sculpture of notaulus: with circular cells. Mesoscutal humeral sulcus: foveate. Mesoscutal suprahumeral sulcus: with circular cells. Parapsidal line: indicated as a furrow. Scutoscutellar sulcus: foveate. L: W of mesoscutellum: 19.8:28.9. Sculpture of mesoscutellum: anteriorly smooth, remainder with compact irregular cells with several setae, a weak discontinuous furrow present anterolaterally. Sculpture of dorsellum: anteriorly foveate, posteriorly smooth, posterior margin sinuous. Sculpture of outer lateral propodeal area: anteriorly with depressions and posteriorly almost smooth, sparsely setose. Sculpture of inner lateral propodeal area: anteriorly sparsely setose, posteriorly smooth with depression. Lateral propodeal carina: sinuous. Posterior propodeal projection: rounded, not extending to anterior margin of T1. Sculpture of metasomal depression: with uneven depressions and sparse pilosity anteriorly. Plical area: anteriorly densely setose, posteriorly smooth with sparse pilosity. Sculpture of propleuron: transversely striate. Sculpture of lateral pronotal area: smooth. Posterior pronotal sulcus: with ovoid cells and two transverse carinae medially. Pronotal cervical sulcus: weakly foveate. Speculum of mesopleuron: transversely carinate with sparse setae. Postacetabular sulcus: foveate. Prespecular sulcus: foveate. Mesepimeral sulcus: foveate. Posterior mesepimeral area: smooth, narrower than mesepimeral sulcus. Mesopleural carina: percurrent, with shallow depressions dorsally. Sculpture of femoral depression: smooth. Mesopleural pit: present. Sculpture of ventral mesopleuron: anteriorly and dorsally with depressions, remainder smooth, sparsely setose. Sculpture of metapleuron: dorsal metapleural area narrow and smooth with sparse setae on anterior margin; ventral metapleural area dorsally smooth and ventrally with a row of foveae followed by sparse depressions, sparsely setose. Metapleural sulcus: foveate. Paracoxal sulcus: with depressions. Metapleural epicoxal sulcus: foveate.

#### Fore wing

L: W: 130.5:59.1 (almost ovoid). Transparency: weakly infuscate. Lengths of R: R1: r-rs in ratio of 52:27:15. R: distant from anterior margin of wing. Anterior margin of fore wing: no downcurve prior to R1.

#### Metasoma

L: W of metasoma: 91.8:38.3. Ratio of length of T1: T2: T3: T4: T5: 18.3:16.6:15.3:14.2:11.9. Anterior margin of T1: straight. Sculpture of T1: medially longitudinally ribbed costate, posteriorly smooth, laterally with depressions anteriorly, medially smooth and posteriorly with short longitudinal costae. Sculpture of T2: basal foveae present, followed by longitudinal costae extending half the length of tergite, remainder smooth; laterally setose. Sculpture of T3: same as T2. Sculpture of T4: same as T2. Sculpture of T5: same as T2, except in posterior half with dense setigerous punctae. Sculpture of T6: with small basal foveae, smooth with dense setigerous punctae.

#### Female

Unknown.

#### Etymology

This species is named ‘Vanaspati’ after a *ragam* or melodic structure in South Indian classical (Carnatic) music which means ‘lord of the forest’.

### *Sparasion visvambari* Veenakumari sp. n. (Figs. 44K, 46H, 47E, 49A–F)

**Fig. 49:**
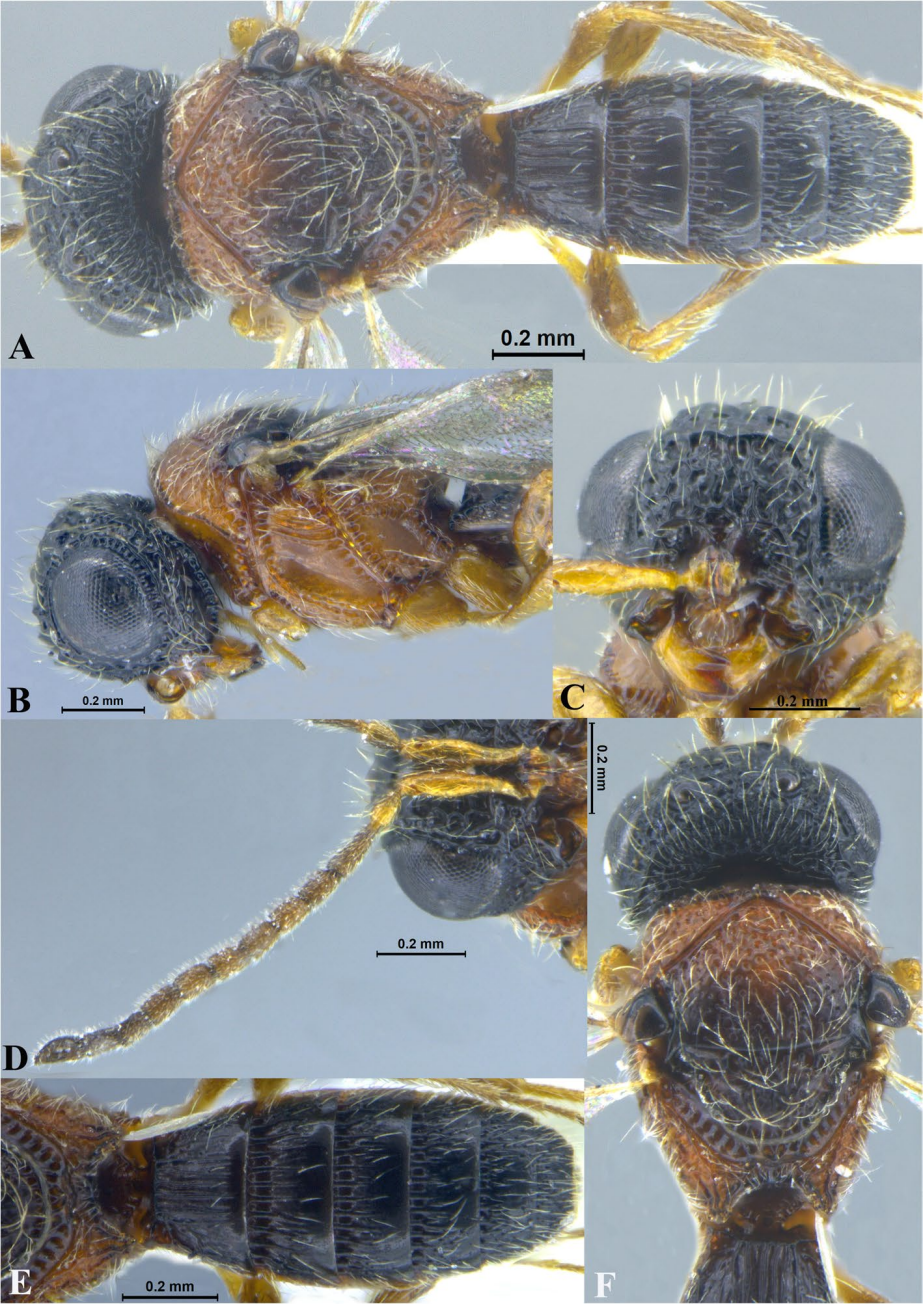
*Sparasion visvambari ***sp. n**., male holotype. **A** Habitus, dorsal view. **B** Head and pleuron. **C** Frons. **D** Antenna. **E** Metasoma. **F** Head and mesonotum

urn:lsid:zoobank.org:act:9A5F4849-02C2-490C-A049-015D55154FCE

#### Diagnosis

*Sparasion visvambari* sp. n. is close to *S. micromerus.* The distinguishing characters are given above under the latter species.

#### Material examined

Holotype: Male, (ICAR/NBAIR/P4743), INDIA: Tripura: Dhalai: Ambassa, 23°52′29ʺN 91°50′47ʺE, 73 m, light trap, 28.VI.2016.

### Description

Male body length = 2.30 mm (*n* = 1).

#### Colour

Head black; dorsal pronotum, anterior half of mesoscutum, mesopleuron and lateral propodeal area red-brown; posterior half of mesoscutum and mesoscutellum brown-black; metasoma black; legs yellow-brown; tegula black with sparse brown patches; radicle, A1 and A2 yellow, remaining antennomeres yellow-brown; mandibles yellow-brown with dark brown teeth.

#### Head

1.3 × as wide as high, 1.1 × as high as long. Setation on head: sparse. IOS: 0.5 × head width, 1.2 × eye length. POL > LOL > OOL: 16.6:11.3:4.6. OOL: 0.6 × MOD. Compound eye: (L: W = 25.8:20.8). Setation of compound eye: sparsely setose. Anterior margin of frons: arcuate with a weak medial indentation. Distance from level of anterior margin of compound eyes to anterior extension of frons in dorsal view: 1.1 × MOD. Number of transverse ledges on upper frons: three. Sculpture of upper frons: anteriorly smooth with sparse setae and posteriorly with two rows of shallow polygonal cells bearing a seta each. Sculpture of lower frons: entirely with shallow polygonal cells bearing setae. Interantennal process: 1.2 × as long as wide, smooth. Transverse carina above interantennal process: with a medial notch. Area ventral to transverse carina above interantennal process: smooth, with sparse setose foveae dorsally and laterally. Sculpture on vertex: with shallow polygonal cells followed by a transverse carina and dense setigerous punctae; anterior ocellus without encircling smooth area; posterior ocellus with smooth depression posteriorly. Sculpture of posterior orbital furrow: dorsally foveate and ventrally with polygonal cells. Genal carina: present. Sculpture of gena: anteriorly with shallow polygonal cells, posteriorly smooth, sparsely setose. Sculpture on A1: smooth with sparse setae. A1: 4.3 × as long as wide. Length of A3: 0.5 × A1 and 1.7 × A2.

#### Mesosoma

Sculpture of dorsal pronotum: setigerous punctate. L: W of mesoscutum: 33.5:46.4. Sculpture of mesoscutum: smooth with dense setigerous foveae. Notaulus: present. Sculpture of notaulus: with circular cells. Mesoscutal humeral sulcus: foveate. Mesoscutal suprahumeral sulcus: with circular cells. Parapsidal line: not indicated. Scutoscutellar sulcus: foveate. L: W of mesoscutellum: 20.7:35.7. Sculpture of mesoscutellum: setigerous foveate with anterior incomplete carina. Sculpture of dorsellum: anteriorly foveate, posteriorly smooth, posterior margin weakly upcurved medially. Sculpture of outer lateral propodeal area: almost smooth with sparse setae. Sculpture of inner lateral propodeal area: anteriorly sparsely setose, posteriorly smooth, with a transverse medial carina. Lateral propodeal carina: anteriorly arched and posteriorly oblique. Posterior propodeal projection: rounded, not extending to anterior margin of T1. Sculpture of metasomal depression: smooth, sparsely setose. Plical area: anteriorly densely setose, posteriorly smooth with sparse pilosity. Sculpture of lateral pronotal area: smooth. Posterior pronotal sulcus: with irregular cells. Pronotal cervical sulcus: foveate. Speculum of mesopleuron: transversely carinate, sparsely setose. Postacetabular sulcus: foveate. Prespecular sulcus: foveate. Mesepimeral sulcus: foveate, foveae incomplete. Posterior mesepimeral area: smooth, narrower than mesepimeral sulcus. Mesopleural carina: percurrent, with a row of irregular foveae dorsally. Sculpture of femoral depression: smooth. Mesopleural pit: present. Sculpture of ventral mesopleuron: with a row of shallow foveae dorsally remainder with sparse setigerous foveae. Sculpture of metapleuron: dorsal metapleural area narrow and smooth with setae on anterior margin; ventral metapleural area dorsally smooth and ventrally with a row of shallow polygonal cells. Metapleural sulcus: foveate, not indicated medially as furrow. Paracoxal sulcus: foveate. Metapleural epicoxal sulcus: indicated as a weak furrow.

#### Fore wing

L: W: 165.0:68.6. Transparency: weakly infuscate. Lengths of R: R1: r-rs in ratio of 71.4:30.0:21.4. R: parallel to anterior margin of wing. Anterior margin of fore wing: with no downcurve prior to R1.

#### Metasoma

L: W of metasoma: 85.1:41.9. Ratio of length of T1: T2: T3: T4: T5: 19.3:18.3:16.1:14.8:7.7. Anterior margin of T1: straight. Sculpture of T1: longitudinally ribbed costate medially, laterally smooth with sparse setae, posteriorly smooth. Sculpture of T2: basal foveae present, followed by longitudinal costae extending half the length of tergite; laterally and posteriorly smooth with setae. Sculpture of T3: same as T2, anteromedially with costae shorter. Sculpture of T4: same as T3. Sculpture of T5: smooth with setigerous punctae and short longitudinal carinae sublaterally on anterior margin; Sculpture of T6: smooth with setigerous punctae.

#### Female

Unknown.

#### Etymology

This species is named ‘Visvambari’ after a *ragam* or melodic structure in South Indian classical (Carnatic) music named after the Hindu goddess who is ‘the creator of the entire universe’.

### *Sparasion zeelafi* Veenakumari sp. n. (Figs. 44L, 46I, 47F, 50A–F)

**Fig. 50:**
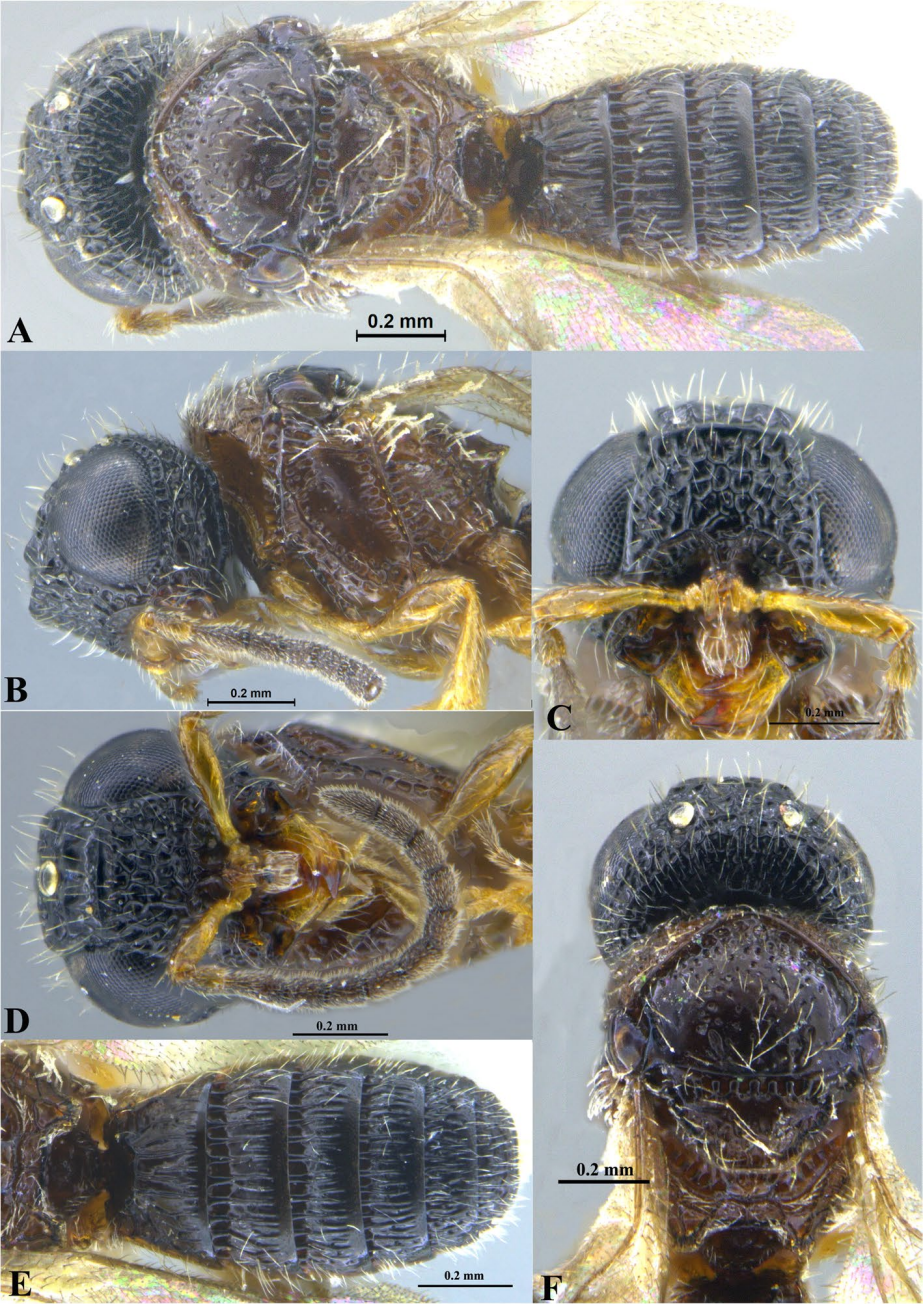
*Sparasion zeelafi ***sp. n**., male holotype. **A** Habitus, dorsal view. **B** Head and pleuron. **C** Frons. **D** Frons and antenna. **E** Metasoma. **F** Head and mesonotum

urn:lsid:zoobank.org:act:1774AA5A-4FB9-4B27-A440-7D537C668FED

#### Diagnosis

*Sparasion zeelafi* sp. n. is close to *S. micromerus* and *S. visvambari* sp. n. but differs from them in the following characters: in *S. zeelafi* sp. n. mesoscutum is sparsely foveate, anterior margin of frons is without medial indentation and notaulus is indicated as an irregular depression. Conversely, in the latter two species mesoscutum is densely foveate, anterior margin of frons is medially indented and notaulus is indicated as ovoid cells.

#### Material examined

Holotype: Male, (ICAR/NBAIR/P4744), **INDIA**: Karnataka: Bengaluru, Hebbal, 13°02′08ʺN 77°35′49ʺE, 906 m, SN, 06.X.2009. Paratypes: 1 male (ICAR/NBAIR/P4745), Karnataka: Bengaluru, Hebbal, 13°02′08ʺN 77°35′49ʺE, 906 m, SN, 06.X.2009; 1 male (ICAR/NBAIR/P4746), Karnataka: Bengaluru, Hebbal, 13°02′08ʺN 77°35′49ʺE, 906 m, SN, 07.X.2009.

### Description

Male body length = 2.31–2.42 mm (*n* = 3).

#### Colour

Head black; mesoscutum and mesoscutellum brown-black; pronotum, pleuron, metascutellum, metanotal trough and lateral propodeal area red-brown; metasoma black-brown; legs yellow-brown; tegula black with sparse brown patches; radicle, A1–A2 yellow-brown, remaining antennomeres brown; mandibles yellow-brown with teeth dark brown.

#### Head

1.3 × as wide as high, as high as long. Setation on head: sparse. IOS: 0.4 × head width, 0.9 × eye length. POL > LOL > OOL: 18.5:12.2:5.4. OOL: 0.7 × MOD. Compound eye: (L: W = 32.5:28.1). Setation of compound eye: glabrous. Anterior margin of frons: arcuate. Distance from level of anterior margin of compound eyes to anterior extension of frons in dorsal view: 1.3 × MOD. Number of transverse ledges on upper frons: three. Sculpture of upper frons: anteriorly with a row of large polygonal cells, followed by a row of narrow polygonal cells and posteriorly with a row of large polygonal cells, all bearing setae. Sculpture of lower frons: entirely with polygonal cells and with sparse short vertical carinae on top of transverse carina above interantennal process. Interantennal process: 1.3 × as long as wide, smooth. Transverse carina above interantennal process: with a medial notch. Area ventral to transverse carina above interantennal process: smooth, with setigerous foveae dorsally and laterally. Sculpture on vertex: anteriorly with large polygonal cells followed by an irregular transverse carina and posteriorly smooth with setigerous punctae; anterior ocellus without smooth area around; lateral ocellus with smooth area anteriorly and posteriorly. Sculpture of posterior orbital furrow: with rectangular cells. Genal carina: present. Sculpture of gena: anteriorly with setigerous polygonal cells and posteriorly smooth with very sparse setae. Sculpture on A1: smooth with sparse setae. A1: 4.8 × as long as wide. Length of A3: 0.5 × A1 and 1.3 × A2.

#### Mesosoma

Sculpture of dorsal pronotum: with setigerous foveae and polygonal cells. L: W of mesoscutum: 37.3:50.6. Sculpture of mesoscutum: smooth with sparse setigerous foveae. Notaulus: present. Sculpture of notaulus: indicated as a furrow. Mesoscutal humeral sulcus: foveate. Mesoscutal suprahumeral sulcus: with circular cells. Parapsidal line: indicated as furrow. Scutoscutellar sulcus: foveate. L: W of mesoscutellum: 21.3:34.6. Sculpture of mesoscutellum: anteriorly smooth with an incomplete furrow, posteriorly with polygonal cells. Sculpture of dorsellum: anteriorly foveate, posteriorly smooth, posterior margin almost straight. Sculpture of outer lateral propodeal area: with shallow depressions, sparsely setose. Sculpture of inner lateral propodeal area: anteriorly with sparse setae, posteriorly smooth, with a transverse medial carina. Lateral propodeal carina: oblique. Posterior propodeal projection: rounded, not extending to anterior margin of T1. Sculpture of metasomal depression: smooth with a medial vertical carina and sparse pilosity. Plical area: anteriorly densely setose, posteriorly with shallow foveae and sparse pilosity. Sculpture of propleuron: smooth. Sculpture of lateral pronotal area: smooth. Posterior pronotal sulcus: with oblong cells. Pronotal cervical sulcus: foveate. Speculum of mesopleuron: transversely carinate, sparsely setose. Postacetabular sulcus: with rectangular depressions. Prespecular sulcus: with wide foveae. Mesepimeral sulcus: with incomplete foveae. Posterior mesepimeral area: smooth, narrower than mesepimeral sulcus. Mesopleural carina: percurrent, with a row of polygonal cells dorsally. Sculpture of femoral depression: smooth. Mesopleural pit: present. Sculpture of ventral mesopleuron: dorsally with a row of rectangular depressions, remainder smooth with sparse setae. Sculpture of metapleuron: dorsal metapleural area not distinct and ventral metapleural area dorsally weakly rugose and ventrally with shallow polygonal cells. Metapleural sulcus: foveate, indicated medially as a furrow. Paracoxal sulcus: with wide foveae. Metapleural epicoxal sulcus: indicated as a furrow with setae.

#### Fore wing

L: W: 161.7:76.6. Transparency: weakly infuscate. Lengths of R: R1: r-rs in ratio of 69.8:38.3:18.7. R: basally closer, gradually distant from anterior margin. Anterior margin of fore wing: with slight downcurve prior to R1.

#### Metasoma

L: W of metasoma: 81.6:47.1. Ratio of length of T1: T2: T3: T4: T5: 15.1:17.3:14.9:13.7:11.1. Anterior margin of T1: convex. Sculpture of T1: medially longitudinally ribbed costate, sublaterally and laterally smooth with sparse punctae, posteriorly smooth. Sculpture of T2: basal foveae present, followed by longitudinal costae; laterally smooth with dense setae, posteriorly smooth with punctae. Sculpture of T3: same as T2. Sculpture of T4: same as T2. Sculpture of T5: longitudinally costate interspersed with setigerous punctae, posteriorly smooth. Sculpture of T6: smooth with setigerous punctae.

#### Female

Unknown.

#### Etymology

This species is named after ‘Zeelaf’, a rarely performed *raga* or melodic structure in North Indian classical (Hindustani) music.

## Key to females of species of *Sparasion* of the Oriental region

1. Upper frons with 2–3 transverse ledges (e.g. Figs. 2B, C and 3B, C); A1 smooth with sparse setae; A3 at most 1.4 × the length of A2 (e.g. Figs. 2D, 3D and 4D); genal carina present (e.g. Figs. 2B, 3B and 4B); mesopleural carina percurrent (e.g. Figs. 31B, 34B and 35B); body colour yellow, brown or black (e.g. Figs. 2A, 3A, 4A, 28A and 29A); mandibles (e.g. Figs. 2C, 3C, 4C, 28C and 29C), A1–A3 (e.g. Figs. 2D, 4D, 29D and 35D) and legs xanthic (e.g. Figs. 2B, 3B, 4B, 28B and 29B); radialis curving upwards distally; fore wing weakly infuscate (e.g. Fig. 18B-E); body short, < 3.5 mm (with the exception of *S. deepaki* sp. n. where body length > 4.5 mm)…………….... 2 **(*Sparasion bilahari *species group)**
  Upper frons with a single transverse ledge (e.g. Figs. 38B, C and 41B, C); A1 densely setigerous punctate (with the exception of *S. karivadana* sp. n. where it is sparely foveate (Fig. 38D)); A3 at least 2.2 × the length of A2 (e.g. Figs. 7D, 9D and 43D); genal carina absent (e.g. Figs. 7B, 9B and 10B); mesopleural carina generally indicated anteriorly (e.g. Figs. 7B, 9B and 10B); body colour steel blue or green, sometimes with black metasoma (e.g. Figs. 5A, 6A, 38A, 41A and 43A); mandibles (e.g. Figs. 5C, 38C, 41C and 43C), A1–A3 (e.g. Figs. 5D, 38D, 41D and 43D) and legs black-brown (e.g. Figs. 5B, 38B, 41B and 43B); radialis almost straight (except in *S. karivadana* sp. n. where it is curving upwards distally); fore wing generally strongly infuscate (e.g. Fig. 33G-I, K); body elongate > 4.5 mm…………….…………………………….12 **(*Sparasion manavati* species group**)
2. Lower frons with several rows of polygonal cells (Figs. 24C and 29C); mesoscutum with dense large polygonal cells posteriorly; mesoscutellum predominantly with compact polygonal cells (Figs. 24G and 29F); metasoma narrow and elongate, at least 2.5 × as long as wide (Figs. 24D and 29E); head, mesosoma and metasoma black (Figs. 24A and 29A); body size > 3.4 mm.…………………………………………………………………………………….…3
  Lower frons medially smooth with polygonal cells on borders (Figs. 1C, 2C, 3C, 4C, 28C, 31C, 34C, 35C and 37C); mesoscutum foveate-punctate or longitudinally carinate posteriorly; mesoscutellum predominantly smooth or at least with a smooth patch anteriorly (Figs. 1F, 2F, 3F, 4F, 28F, 31F, 34F, 35F and 37F); Metasoma short and wide, at most 2 × as wide as long (Figs. 1E, 2E, 3E, 4E, 28E, 31E, 34E, 35E and 37E); head, mesosoma and metasoma either entirely or party xanthic or brown; body size < 2.7 mm……………………………………………………………………………………….4
3. Distance from level of anterior margin of compound eyes to anterior extension of frons is at least 1.8 × MOD (Fig. 17K); R1 > 2.0 × the length of r-rs; R gradually curving towards anterior margin of wing up to 0.6 × its length and subsequently deflecting away from margin towards bulla; anterior margin of fore wing with a downcurve prior to R1 (Fig. 18K); frons with several transverse carinae with rows of polygonal cells between them (Fig. 29C); posterior pronotal sulcus indicated as large ovoid cells (Fig. 29B); T1 anteromedially with a short spine; body size > 4.5 mm (Fig. 29B)………………………………………………………………………………...***Sparasion deepaki *sp. n.**
  Distance from level of anterior margin of compound eyes to anterior extension of frons is at most 1.2 × MOD (Fig. 17G); R1 < 1.8 × the length of r-rs; R gradually curving away from anterior margin towards bulla; anterior margin of fore wing without a downcurve prior to R1 (Fig. 18G); frons with several polygonal cells and with two short transverse carinae medially (Fig. 24C); posterior pronotal sulcus indicated as narrow transverse cells (Fig. 24B); T1 anteromedially without a short spine; body size 3.5 mm (Fig. 24B)……………………………………………….… ***Sparasion coconcu*s Kozlov and Lê**
4. Anterior frons with two transverse ledges (Fig. 2B); mesoscutum with longitudinal carinae interspersed with sparse setigerous foveae (Fig. 2F)……………………………….…***Sparasion bhupali*** **sp. n.**
  Anterior frons with three transverse ledges (Figs. 1B, 3B, 4B, 28B, 31B, 34B, 35B and 37B); mesoscutum predominantly smooth with either setigerous foveae or punctae or both (Figs. 1F, 3F, 4F, 28F, 31F, 34F, 35F and 37F)……………………………………….…………….5
5. R1 > 1.8 × the length of r-rs (Fig. 18D, E, J); transverse carinae on speculum of mesopleuron well defined (Figs. 3B, 4B and 28B)……………………………………….…...6
  R1 < 1.3 × the length of r-rs (Figs. 18B, L and 33B-D); transverse carinae on speculum of mesopleuron feeble (Figs. 1B, 31B, 34B, 35B and 37B) ………………….……8
6. Vertex smooth except for three shallow polygonal cells on either side anterior ocellus; transverse carina present between lateral ocelli (Fig. 17D); first frontal ledge almost straight and placed lower, at 2/3 level along ventral margin of eye (Fig. 3C); ventral mesopleuron smooth (Fig. 3B)……………………………….…***Sparasion bihagi* sp. n.**
  Vertex with polygonal cells; transverse carina between lateral ocelli absent (Fig. 17E, J); first frontal ledge curving downwards at either end and placed higher, almost level with anterior margin of eye (Figs. 4C and 28C); ventral mesopleuron with rectangular cells anteriorly (Figs. 4B and 28B) …………………………………………………………………7
7. Mesoscutum smooth with sparse setigerous punctae; mesoscutellum smooth with sparse setigerous foveae posteriorly (Fig. 4F); anterior margin of frons arcuate (Fig. 17E); posterior propodeal projection rounded (Fig. 4F); T4 > 1.3 × longer than T5 (Fig. 4E); head, mesosoma and metasoma brown-black to brown except for yellow–brown pleuron and propodeum (Fig. 4A) ……………………………………… ***Sparasion bilahari* sp. n.**
  Mesoscutum weakly rugose with sparse setigerous punctae; mesoscutellum anteriorly smooth, posteriorly longitudinally carinate with shallow foveae between costae (Fig. 28F); anterior margin of frons weakly sinuate (Fig. 17J); posterior propodeal projection angular (Fig. 28F); T4 and T5 subequal in length (Fig. 28E); head brown to black, mesosoma and metasoma yellow–brown (Fig. 28A) ………… ***Sparasion darbari* sp. n.**
8. T2 and T3 medially smooth (Figs. 1E and 34E)……………………………………….………9
  T2 and T3 anteromedially longitudinally costate and posteromedially smooth (Figs. 31E, 35E and 37E)………………………………………………………………………………...10
9. Metasoma narrow and elongate, at least 2 × as long as wide (Fig. 1E); genal carina not percurrent, curving towards orbital carina basally (Fig. 1B); first frontal ledge wide and curving downwards at either end (Fig. 1C); outer lateral propodeal area densely foveate; foveae of scutoscutellar sulcus incomplete (Fig. 1F); basal foveae on T1–T4 narrow and elongate (Fig. 1E)……………………………………………… ***Sparasion bhairavi* sp. n.**
  Metasoma short and wide, at most 1.6 × as long as wide (Fig. 34E); genal carina percurrent (Fig. 34B); first frontal ledge short and straight (Fig. 34C); outer lateral propodeal area smooth; foveae of scutoscutellar sulcus complete (Fig. 34F); basal foveae on T1–T4 short and oval (Fig. 34E) …………………………... ***Sparasion hindoli* sp. n.**
10. Outer lateral propodeal area almost smooth; mesoscutellum setigerous foveate except for a smooth patch anteromedially (Fig. 31F); foveae of occipital carina wider than long (Fig. 31B); T1 without basal foveae; basal foveae on T2–T4 short and almost round; basal foveae on T5 absent (Fig. 31E); A1 < 3 × as long as wide (Fig. 31D) ……………………………………………………………………………………….... ***Sparasion elbakyanae* sp. n.**
  Outer lateral propodeal area either sparsely foveate or densely punctate-foveate; mesoscutellum predominantly smooth (Figs. 35F and 37F); foveae of occipital carina subequal in length and width (Figs. 35B and 37B); T1 with narrow and elongate basal foveae; basal foveae on T2–T4 narrow and elongate; basal foveae on T5 present (Figs. 35E and 37E); A1 > 4 × as long as wide (Figs. 35D and 37D) …………………………………………….....11
11. Lateral propodeal area sparsely foveate; posterior lateral propodeal area present as a wide lamella (Fig. 35F); T1–T3 posteromedially weakly rugose; T2–T5 anteriorly with a narrow smooth area anterior to basal foveae (Fig. 35E)……………………………………………………...… ***Sparasion kalyani* sp. n.**
  Lateral propodeal area densely punctate-foveate; posterior lateral propodeal area narrow and notched (Fig. 37F); T1–T3 posteromedially smooth; T2–T5 anteriorly with only basal foveae (Fig. 37E) ………………………………………………………… ***Sparasion kanakangi* sp. n.**
12. Head smooth with sparse punctae……………….…..*Sparasion parcepunctatus* Kieffer*
  Head densely sculptured, either foveate or with polygonal cells (e. g. Figs. 5F, 6A, 7F, 38F, 41F and 43F)…………………………………………………….……………………….13
13. Anterior width of metasomal depression at least half as wide as its posterior margin (Fig. 38F); A1 smooth with sparse setigerous foveae; A3 at most 1.2 × length of A2 (Fig. 38D); T1–T3 medially costate, remainder smooth with sparse setigerous punctae (Fig. 38E); radialis curving upwards distally (Fig. 33E); body length < 4.7 mm; head and mesosoma metallic green, metasoma black (Fig. 38A, E)………….. ***Sparasion karivadana* sp. n.**
  Anterior width of metasomal depression narrow, at most ¼ the width of posterior margin (e.g. Figs. 26D, 27C and 41F); A1 with dense setigerous punctae; A3 at least 2.2 × length of A2 (e.g. Figs. 7D, 8D, 9D, 41D and 43D); T1–T3 costate except laterally and sometimes medially (e.g. Figs. 5E, 7E, 8E, 26E and 43E); radialis straight (e.g. Fig. 33G, H, K, M); body length > 5.3 mm; body generally metallic blue or green, sometimes black-blue (e.g. Figs. 5A, 22A, 27A, 41A and 43A)…………………………….14
14. Mesoscutum with deep punctae and with longitudinal blunt carinae (Fig. 26D); longitudinal costae on T3–T5 closely spaced; T5 medially densely punctate and laterally with closely spaced longitudinal costae; metasoma 3.6 × as long as wide (Fig. 26E)……………………………………………………….. ***Sparasion coeruleus* Kieffer**
  Mesoscutum foveate-punctate (e.g. Figs. 5F, 6A, 27G, 41F and 43F); longitudinal costae on T3–T5 spaced apart, medially with smooth area and short setae; T5 with sparse longitudinal costae except for smooth area medially and posteriorly with setae; metasoma at most 3.2 × as long as wide (e.g. Figs. 5E, 7E, 8E, 9E and 43E)…………………..….15
15. Lower frons entirely with polygonal cells or at most with a narrow smooth patch medially (e.g. Figs. 11C, 15C, 22F, 41C and 43C) …………………………………………………...16
  Lower frons with transverse carinae medially, remainder with polygonal cells (e.g. Figs. 5C, 7C, 8C, 9C, 10C and 12C) …………………………….…………………………..21
16. T3 medially with effaced longitudinal costae and sparse setae, T4 with a wide smooth area medially; metasoma short and stout, < 2.4 × as long as wide (Fig. 41E)…………………………………………………………… ***Sparasion manavati* sp. n.**
  T3–T4 costate at most with a narrow smooth area medially with sparse setae; metasoma narrow and elongate, > 2.7 × as long as wide (Figs. 11D, 15A, 22G, 27A, F and 43E)……..17
17. Anterior mesoscutum smooth with sparse punctae (Fig. 11F); ventral mesopleuron sparsely setose; ventral metapleuron with shallow depressions (Fig. 11B); body length 8.8 mm……………………………………………………………………………………………***Sparasion sinensis* Walker**
  Anterior mesoscutum with dense punctae or foveae (Figs. 15A, 22H, 27G and 43F); ventral mesopleuron densely setose; ventral metapleuron with dense polygonal cells (Figs. 15B, 22E, 27B and 43B); body length < 6.2 mm…………………………………………………..18
18. Notaulus absent (Fig. 22H); gena smooth with sparse foveae; speculum of mesopleuron with setigerous foveae; mesoscutum flat when viewed laterally; dorsal metapleural area large, almost half the size of dorsal smooth area of ventral metapleural area (Fig. 22E)………………………………………………….………...***Sparasion cellularis* Strand**
  Notaulus present (Figs. 15A, 27G and 43F); gena unevenly sculptured either with polygonal cells or punctae; speculum of mesopleuron with setigerous foveae interspersed with either prominent or weak transverse carinae; mesoscutum convex when viewed laterally; dorsal metapleural area narrow, at most 1/3 the size of dorsal smooth area of ventral metapleural area (Figs. 15B, 27B and 43B)………………………………………………….19
19. Speculum of mesopleuron with prominent transverse carinae and sparsely setose; ventral mesopleuron foveate with interspersed transverse carinae (Fig. 27B); metasomal depression with two pairs of transverse carinae; dorsellum entirely foveate with small smooth areas posterolaterally (Fig. 27C); parapsidal line indicated as a deep furrow (Fig. 27G)………………………………………………… ***Sparasion cullaris* Kozlov and Lê**
  Speculum of mesopleuron either with sparse foveae or punctae interspersed with sparse transverse carinae and densely setose; ventral mesopleuron anteriorly with polygonal cells and posteriorly either smooth or foveate (Figs. 15B and 43B); metasomal depression with or without transverse furrow; dorsellum anteriorly foveate and posteriorly smooth; parapsidal line indicated as a shallow furrow (Figs. 15A and 43F)………….………………20
20. Vertex around posterior ocelli predominantly smooth with sparse foveae (Fig. 47C); lower frons with a network of polygonal cells (Fig. 15C); posterior propodeal projection not reaching anterior margin of metasoma; posterior mesepimeral area narrower than the width of foveae of mesepimeral sulcus; gena with setigerous punctae (Fig. 15B)………….………………***Sparasion travancoricus* Mani and Sharma**
  Vertex around posterior ocelli with polygonal cells and foveae (Fig. 36F); lower frons with circular and polygonal cells with smooth interstices (Fig. 43C); posterior propodeal projection extending on to anterior margin of metasoma (Fig. 43F); posterior mesepimeral area wider than the width of foveae of mesepimeral sulcus; gena with setigerous polygonal cells (Fig. 43B)……………………………………………………..…***Sparasion meghmalhari* sp. n.**
21. T4 with longitudinal costae extending 0.6 × the length of tergite, remainder smooth with punctae; T5 narrow and elongate, < 1.4 × as wide as long; T6 narrow and elongate < 1.5 × as wide as long (Fig. 6A); R distant and parallel to the anterior margin of wing along its entire length (Fig. 33J)……………………………………………………………………………***Sparasion philippinensis* Kieffer**
  T4 with longitudinal costae extending almost the entire length except for a narrow smooth posterior margin; T5 short and wide, > 1.7 × as wide as long; T6 short and wide, > 2.5 × as wide as long (Figs. 5E, 7E, 8E, 9E, 10E, 12E, 13E and 14E); R closer to anterior margin of wing at least proximally (Figs. 33K, I, M, L and 46A, C-E)……………………………………………………………………………….……..22
22. Anterior projection of frons (in dorsal view) short, distance from anterior margin of compound eyes to anterior extension of frons < MOD (Fig. 36H, J, K, O)...................................................................................................................................23
  Anterior projection of frons (in dorsal view) elongate, distance from anterior margin of compound eyes to anterior extension of frons > MOD (Figs. 36L, M and 47A, B)………………………………………………………………………………….…..26
23. Anterior margin of frons almost straight (Fig. 36H); lower frons medially with wide transverse carinae progressively increasing in width anteriad (Fig. 5C); lateral propodeal area elongate and narrow posteriorly (Fig. 5F); ventral mesopleuron smooth with setigerous punctae (Fig. 5B)…………………………………… ***Sparasion pahadi* sp. n.**
  Anterior margin of frons medially indented (Fig. 36J, K, O); lower frons medially with either short subequal transverse carinae or with carinae progressively reducing in width anteriad forming horizontal cells (Figs. 7C, 8C and 12C); lateral propodeal area wide and not tapering posteriorly (Figs. 7F, 8F and 12F); ventral mesopleuron smooth with setigerous foveae (Figs. 7B, 8B and 12B)……………………24
24. T5 entirely punctate without longitudinal costae (Fig. 7E); lateral propodeal area posteriorly not extending onto anterior margin of T1 (Fig. 7F); transverse ledge on frons extending up to orbits, curving downwards at either end (Fig. 7C); R gradually diverging *from* anterior margin of fore wing (Fig. 33L)…….… ***Sparasion ratnangi* sp. n.**
  T5 with longitudinal costae interspersed with punctae (Figs. 8E and 12E); lateral propodeal area posteriorly extending onto anterior margin of T1 (Figs. 8F and 12F); transverse ledge on frons short and straight, culminating well before the orbits (Figs. 8C and 12C); R closer to anterior margin of fore wing proximally (Figs. 33K and 46C)………………………...….25
25. Sculpture on upper frons with polygonal cells without smooth interstices (Fig. 36K); basal gena with large polygonal cells anteriorly (Fig. 8B); OOL at least 0.7 × MOD (Fig. 36K); metasoma elongate > 2.8 × as long as wide; T4 medially smooth with setae; (Fig. 8E); lateral propodeal carina almost straight in posterior half (Fig. 8F); anterior margin of fore wing with a distinct downcurve prior to R1; posterior margin of fore wing weakly pointed (Fig. 33K)………………………………………….….. ***Sparasion rupavati* sp. n.**
  Sculpture on upper frons with circular cells, with smooth interstices (Fig. 36O); basal gena punctate anteriorly (Fig. 12B); OOL at most 0.3 × MOD (Fig. 36O); metasoma short and wide, < 2.3 × as long as wide; T4 medially longitudinally costate with sparse setae posteriorly (Fig. 12E); lateral propodeal carina sinuous in posterior half (Fig. 12F); anterior margin of fore wing with no downcurve prior to R1; posterior margin of fore wing rounded (Fig. 46C)…………………………………………………………. ***Sparasion sivaranjini* sp. n.**
26. Anterior margin of frons with a medial indentation (Figs. 36L and 47A); R closer to anterior margin of fore wing basally (Figs. 33M and 46D) …………………………………………. 27
  Anterior margin of frons almost straight (Figs. 36H and 47B); R distant from anterior margin of fore wing basally (Fig. 46A, E)…………………………………………………….28
27. Mesoscutum densely foveate-punctate; mesoscutal humeral sulcus and suprahumeral sulcus indicated as small foveae; scutoscutellar sulcus not foveate medially; posterior half of lateral propodeal area abbreviate; outer lateral propodeal area densely setose (Fig. 13F) basal foveae present on T5 (Fig. 13E); A3 < 2.4 × as long as A2 (Fig. 13D); head and mesosoma steel green (Fig. 13A)………………………..…. ***Sparasion syamalangi* sp. n.**
  Mesoscutum sparsely foveate-punctate; mesoscutal humeral sulcus and suprahumeral sulcus indicated as large depressions; scutoscutellar sulcus foveate medially; posterior half of lateral propodeal area elongate; outer lateral propodeal area sparsely setose (Fig. 9F); basal foveae absent on T5 (Fig. 9E); A3 > 3.5 × as long as A2 (Fig. 9D); head and mesosoma steel blue (Fig. 9A)…………………………….….***Sparasion salagami* sp. n.**
28. Mesoscutum sparsely foveate-punctate (Fig. 10F); A1 > 4.6 × as long as wide (Fig. 10D); lower gena smooth with setigerous punctae; paracoxal sulcus indicated as wide foveae; plical area sparsely setose; setae on speculum of mesopleuron short and sparse (Fig. 10B); longitudinal costae on T2–T3 closely spaced (Fig. 10E)….***Sparasion shulini* sp. n.**
  Mesoscutum densely foveate (Fig. 14F); A1 < 4.0 × as long as wide (Fig. 14D); lower gena with setigerous foveae; paracoxal sulcus indicated as polygonal cells; plical area densely setose; setae on speculum of mesopleuron dense and elongate (Fig. 14B); longitudinal costae on T2–T3 spaced apart (Fig. 14E)…………… ***Sparasion todi* sp. n.**

* Based on literature

## Key to males of species of *Sparasion* of the Oriental region

1. Fore wing ovoid (Fig. 46G)………………………………………………….. ***Sparasion vanaspati* sp. n.**
  Fore wing angular (e. g. Figs. 18A, 33A, F and 46H, I)………………..…………………………..………2
2. Transverse ledge on upper frons absent (Fig. 40B); ventral metapleural area entirely with polygonal cells except for a narrow smooth patch dorsally (Fig. 40B); vertex smooth with sparse punctae (Fig. 36D)………... ***Sparasion lividus* Johnson et al.**
  Upper frons with 1–3 transverse ledges (e. g. Figs. 16B, 23G, 30B, 49C and 50C); ventral metapleural area dorsally smooth and ventrally sculptured (e. g. Figs. 30B, 32B, 45B, 49B and 50B); vertex densely sculptured (e. g. Figs. 17L, N, 36G and 47E, F)………………………………………………….. 3
3. Upper frons with 2–3 transverse ledges (e. g. Figs. 30B, 45B, 49B and 50B); A1 smooth with sparse setae (e. g. Fig. 20B, D, F, H); radialis curving upwards distally (e. g. Fig. 46H, I); body colour brown or black, mandibles, A1–A3, legs xanthic (e. g. Fig. 20) ………………………………………………..…. 4 (***Sparasion bilahari* species group)**
  Upper frons with a single transverse ledge (e. g. Figs. 23G and 25B); A1 densely setigerous punctate (e. g. Figs. 23G, 32C and 39C); radialis almost straight (e. g. Figs. 23D and 33A); body colour steel blue or green, mandibles, A1–A3, legs black-brown (e. g. Fig. 42)……………………………………………..14 **(*Sparasion manavati* species group)**
4. Upper frons with oblique carinae anterior to lateral ocellus and a median transverse carina between lateral ocelli; distance from the level of anterior margin of compound eyes to anterior extension of frons in dorsal view 1.8 × MOD (Fig. 16D); gena predominantly smooth with sparse punctae and carinae; orbital carina absent (Fig. 16B); dorsal and ventral metapleural area transversely carinate (Fig. 16B)…………………………………………...……… ***Sparasion albopilosellus* Cameron**
  Upper frons without carinae anterior to lateral ocellus; distance from the level of anterior margin of compound eyes to anterior extension of frons in dorsal view generally < 1.3 × MOD (e. g. Fig. 20A, C, G, K); gena either with polygonal cells or punctae or foveae; orbital carina present; dorsal metapleural area generally smooth and ventral metapleural area either foveate or with polygonal cells (e. g. Figs. 1B, 3B, 4B and 28B)……5
5. Mesoscutum either with polygonal cells or with polygonal cells and longitudinal carinae (Figs. 20C and 30F)……………………………………………………………………..…….6
  Mesoscutum either punctate or foveate (e. g. Figs. 20A, E, G, I, K and 45D)....…..7
6. Mesoscutum anteromedially with polygonal cells and posteromedially with longitudinal carinae; notaulus absent; mesoscutellum anteriorly smooth and posteriorly with sparse foveae interspersed with longitudinal carinae; metasoma short and wide, < 1.6 × as long as wide; posterior propodeal projections short and wide (Fig. 20C). ……………………………… ***Sparasion bhupali* sp. n.**
  Mesoscutum anteriorly with polygonal cells and posteriorly smooth with sparse punctae; notaulus present; mesoscutellum with network of round foveae (Fig. 30F); metasoma elongate and narrow, 2.7 × as long as wide; posterior propodeal projections narrow (Fig. 30D)………………………………………………….…***Sparasion domes* Kozlov and Lê**
7. Notaulus present (Figs. 45D, 49F and 50F)……………………………………………..…….8
  Notaulus absent (Figs. 20A, E, G, I, K)…………………………………..……10
8. Mesoscutum sparsely foveate; anterior half of mesoscutellum smooth and posterior half with polygonal cells; notaulus indicated as an irregular depression (Fig. 50F); anterior margin of frons without medial indentation (Fig. 47F); speculum of mesopleuron with two transverse carinae (Fig. 50B)……………………………………………...…….***Sparasion zeelafi* sp. n.**
  Mesoscutum densely foveate; mesoscutellum entirely sculptured, at most with a small smooth patch anteromedially; notaulus indicated as ovoid cells (Figs. 45D and 49F); anterior margin of frons medially indented (Figs. 45D and 49C); speculum of mesopleuron with four transverse carinae (Figs. 45B and 49B)…………………………………………………….…9
9. T2–T4 with sparse longitudinal costae in anterior half and posterior half smooth; T5 smooth with sparse setigerous punctae (Fig. 49E); longitudinal carinae absent anterior to medial occipital carina; mesoscutal flange elongate, > 2 × as long as wide; foveae of mesoscutum widely spaced; dorsal pronotum sparsely punctate (Fig. 49F)………………………………………………………………………………………….…… ***Sparasion visvambari* sp. n.**
  T2–T4 entirely densely longitudinally costate except for a narrow smooth posterior margin; T5 costate in anterior half and smooth in posterior half (Fig. 45C); several longitudinal carinae present anterior to medial occipital carina; mesoscutal flange short, < 2 × as long as wide; foveae of mesoscutum closely spaced; dorsal pronotum densely foveate-punctate (Fig. 45D)……………………………...…….***Sparasion micromerus* Kozlov and Lê**
10. Lateral propodeal area predominantly smooth with sparse carinae (Fig. 20E, G); R1 elongate, > 1.8 × the length of r-rs (Fig. 18D, E); metasoma short and wide < 1.4 × as wide as long (Fig. 20E, G)………………………………………………………...….11
  Lateral propodeal area foveate-punctate (Fig. 20A, I, K); R1 short, < 1.3 × the length of r-rs (Figs. 18B and 33B, D); metasoma narrow and elongate > 1.8 × as wide as long (Fig. 20A, I, K) …………………………………………………………………………..12
11. Lateral margin of mesoscutum discontinuous anteromedially; posterior propodeal projection narrow and angular; dorsellum entirely foveate except for small smooth area posterolaterally; longitudinal costae on T2–T4 medially short with a wide smooth area posteriorly (Fig. 20E); fore wing with a downcurve prior to R1 (Fig. 18D)…………………………………………… ***Sparasion bihagi* sp. n.**
  Lateral margin of mesoscutum continuous anteromedially; posterior propodeal projection wide and rounded; dorsellum with anterior half foveate and posterior half smooth; T2–T4 entirely longitudinally costate except for a narrow posterior margin with punctae (Fig. 20G); fore wing straight, without a downcurve prior to R1 (Fig. 18E)……………………………………………………………………………….. ***Sparasion bilahari* sp. n.**
12. Mesoscutum sparsely punctate; basal foveae on T1 short; basal foveae on T3–T4 subequal in length and width; costae on T3–T4 extending almost the entire length of tergite, except for a narrow smooth patch with punctae posteriorly (Fig. 20I) ………………………………………………………………………..……. ***Sparasion hindoli* sp. n.**
  Mesoscutum densely punctate; basal foveae in T1 elongate; basal foveae on T3–T4 longer than wide; T3–T4 with short costae anteromedially followed by a large smooth area (Fig. 20A, K) ………………………………………………………..………….13
13. Metasoma > 2.1 × as long as wide; metasomal depression smooth; space between costae on metasomal tergites weakly punctate; basal foveae on T2–T3 with rounded anterior margin; T6 with basal foveae (Fig. 20A)…………………………………….... ***Sparasion bhairavi* sp. n.**
  Metasoma < 1.8 × as long as wide; metasomal depression transversely carinate; space between costae on metasomal tergites longitudinally striate without punctae; basal foveae on T2–T3 with truncate anterior margin; T6 without basal foveae (Fig. 20K) ………………………………………………………...…….. ***Sparasion kanakangi* sp. n.**
14. Mesoscutum with dense foveae and punctae (Figs. 25C and 42A-D)……...…15
  Mesoscutum smooth with sparse punctae (Figs. 23A, 32F and 39B)…………………….….19
15. Oblique carinae present on temples (Fig. 25F); transverse pronotal carina laterally with spine-like projection (in dorsal view) (Fig. 25C)……………..***Sparasion coeruleus* Kieffer**
  Oblique carinae absent on temples; transverse pronotal carina without lateral projections (in dorsal view) (Fig. 42A-D)…………………………………………….16
16. Anterior projection of frons (in dorsal view) without medial indentation (Fig. 36H); ligula on anteromedial mesoscutum elongate, at least 2 × as long as wide (Fig. 42B)………………………………………………………………***Sparasion pahadi* sp. n.**
  Anterior projection of frons (in dorsal view) with medial indentation (Fig. 36E, J, K); ligula on anteromedial mesoscutum short, at most as long as wide (Fig. 42A, C, D)…. …. ……………………………………………………………………..………....17
17. Width of anterior margin of T1 shorter than its length; metasoma elongate > 3.1 × as long as wide (Fig. 42C)…………………………………………………………***Sparasion rupavati* sp. n.**
  Width of anterior margin of T1 longer than its length; metasoma short and wide, < 2.8 × as long as wide (Fig. 42A, D)…………………………………………………18
18. Mesoscutum smooth with foveae and punctae; costae on T1 not extending entire length of tergite, T1 posteromedially smooth; mesoscutal flange wide (Fig. 42D) …………………………………………………………………..***Sparasion ratnangi* sp. n.**
  Mesoscutum weakly rugose with foveae and punctae; costae on T1 extending entire length of tergite; mesoscutal flange elongate and narrow (Fig. 42A)………………………………………………………...….***Sparasion manavati* sp. n.**
19. Mesoscutellum predominantly smooth; inner lateral propodeal area unevenly foveate-punctate; anterior width of metasomal depression ¾ width of posterior margin; lateral propodeal carina almost vertical (Fig. 39B)……………………………………………….…….. ***Sparasion karivadana* sp. n.**
  Mesoscutellum with dense foveae or with polygonal cells, at most with a small smooth area anteromedially; inner lateral propodeal area either entirely smooth or smooth with sparse rugae; anterior width of metasomal depression at most 1/3 width of posterior margin; lateral propodeal carina oblique (Figs. 23B and 32F)……………………………….20
20. Metasoma and ventral metapleuron densely setose (Fig. 32B, D); lateral propodeal area narrow and smooth; lateral propodeal carina sinuate; mesoscutellum anteromedially smooth with two rows of foveae on posterior margin (Fig. 32F); mesepimeral area wider than mesepimeral sulcus (Fig. 32B); fore wing 3.5 × as long as wide; fore wing angular and tapering distally (Fig. 33A) ………………………………………………………….…***Sparasion formosus* Kieffer**
  Metasoma and ventral metapleuron sparsely setose (Fig. 23A, F); lateral propodeal area wide and rugose, rugae thickened; lateral propodeal carina almost straight; mesoscutellum densely packed polygonal cells (Fig. 23B); mesepimeral area narrower than mesepimeral sulcus (Fig. 23F); fore wing 3 × as long as wide; fore wing rounded distally (Fig. 18F)………………………………………………………..…***Sparasion cellularis* Strand**

## Checklist and species-group placement of Oriental *Sparasion*

### *Sparasion bilahari* species group

*Sparasion albopilosellus* Cameron

*Sparasion bhairavi* Veenakumari sp. n.

*Sparasion bhupali* Veenakumari sp. n.

*Sparasion bihagi* Veenakumari sp. n.

*Sparasion bilahari* Veenakumari sp. n.

*Sparasion coconcu*s Kozlov and Lê

*Sparasion darbari* Veenakumari sp. n.

*Sparasion deepaki* Veenakumari sp. n.

*Sparasion domes* Kozlov and Lê

*Sparasion elbakyanae* Veenakumari sp. n.

*Sparasion hindoli* Veenakumari sp. n.

*Sparasion kalyani* Veenakumari sp. n.

*Sparasion kanakangi* Veenakumari sp. n.

*Sparasion micromerus* Kozlov and Lê

*Sparasion vanaspati* Veenakumari sp. n.

*Sparasion visvambari* Veenakumari sp. n.

*Sparasion zeelafi* Veenakumari sp. n.

### *Sparasion manavati* species group

*Sparasion cellularis* Strand

*Sparasion coeruleus* Kieffer

*Sparasion cullaris* Kozlov and Lê

*Sparasion formosus* Kieffer

*Sparasion karivadana* Veenakumari sp. n.

*Sparasion manavati* Veenakumari sp. n.

*Sparasion meghmalhari* Veenakumari sp. n.

*Sparasion pahadi* Veenakumari sp. n.

*Sparasion parcepunctatus* Kieffer*

*Sparasion philippinensis* Kieffer

*Sparasion ratnangi* Veenakumari sp. n.

*Sparasion rupavati* Veenakumari sp. n.

*Sparasion salagami* Veenakumari sp. n.

*Sparasion shulini* Veenakumari sp. n.

*Sparasion sinensis* Walker

*Sparasion sivaranjini* Veenakumari sp. n.

*Sparasion syamalangi* Veenakumari sp. n.

*Sparasion todi* Veenakumari sp. n.

*Sparasion travancoricus* Mani and Sharma

### Other species of *Sparasion*

*Sparasion lividus* Johnson, Masner & Musetti

*not dealt with here as the museum where the type specimen was deposited remains unknown [7].

## Discussion

All Platygastroidea (except Platygastridae) are idiobiont endoparasitoids of insects (belonging to the orders Hemiptera, Odonata, Orthoptera, Mantodea, Embidiina, Coleoptera, Neuroptera, Lepidoptera and Diptera), and spiders (Arachnida: Araneae). On the other hand, the majority of Platygastridae are koinobiont endoparasitoids of immature stages of Auchenorrhyncha, Sternorrhyncha and Cecidomyiidae (Diptera) with some being egg parasitoids of Coleoptera and Hemiptera (Auchenorrhyncha). Trichogrammatidae and Mymaridae are the other families of insects known to be parasitoids of the eggs of insects [1, 5].

### Hosts

Hosts of most species of Platygastroidea remain unknown. The hosts of *Sparasion* are however known. They parasitize the eggs of *Anabrus simplex* Haldeman, 1852*, A. longipes* Caudell, 1907 and *Atlanticus gibbosus* Scudder, 1894 (Orthoptera: Tettigoniidae)*.* The first recorded host for *Sparasion* was *Anabrus simplex* - the Mormon cricket (a misnomer, as it is a long horned grasshopper) - a serious pest of several crops in North America in the 1920s. The eggs of these grasshoppers collected in 1925 and 1926 from Charlo, Lake County, Montana, USA were found to be heavily parasitized by *S. pilosus* Ashmead, 1893 resulting in significant reductions in populations of the pest in 1927 [24]. Later, based on his observations in British Columbia, Spencer [25] surmised that a species of *Sparasion* parasitized the eggs of *Anabrus longipes*. Later still, Grissell [26] observed both females and males of an unidentified species of *Sparasion* flying low over the surface of the soil where *Atlanticus gibbossus* had laid eggs in Alachua County, Florida. He inferred that these females were ovipositing in eggs of the grasshopper, though attempts at rearing them from these eggs were futile.

All species of *Sparasion* are thought to parasitize eggs of Tettigoniidae and Gryllacrididae, all of which in turn lay their eggs either in the stems or leaves of plants, or in the soil [10]. Records of Gryllacrididae (Stenopelmatinae) as hosts of Sparasionidae are doubtful [4].

### Prospective candidates for biological control

Platygastroidea are significant in that they kill the host insects either at the egg or early immature stages, before the hosts are capable of inflicting significant damage to crops. This gives them an edge over other parasitoids that attack their hosts in their later stages of development. Consequently they have been used as biological control agents either solely or in concert with other methods in Integrated Pest Management (IPM) programmes against pests in agricultural and forest ecosystems as well as against insect pests affecting human and animal health [1].

Prior taxonomic awareness of the native fauna of natural enemies and their hosts is a prerequisite of vital importance for the successful deployment of these insects as bio-agents in biological control / IPM programmes. There would then be far ‘fewer cases of such programmes being classified as failures in biological control’ [27] by helping circumvent the use of misidentified, and therefore wrong natural enemy species in these pest management programmes. This paper seeks to redress the lacuna on this front with respect to the *Sparasion* of India in particular. Further studies could reveal the presence of more species particularly from areas where collections are yet to be made in India and elsewhere.

## Conclusions

With the addition of twenty-four new species in this study, the fauna of *Sparasion* has been tripled in the Oriental region. As previous descriptions of Oriental *Sparasion* were scanty they have been redescribed and illustrated with photographs (most for the first time). Keys have been provided to the females and males of all these species to enable their easy identification by those taking up studies on the group in future. Colour photographs will further aid their easy identification. Known to be egg parasitoids of grasshoppers that have been observed to efficiently suppress populations of these pests in varied ecosystems, they have the potential to be incorporated in biological control and IPM programmes for the management of grasshopper pests once successful mass rearing techniques for the parasitoid are developed.

## Material and methods

All specimens were collected using the following methods: yellow pan trap (YPT), Malaise trap (MT), sweep net (SN) and light trap. They were sorted under a stereo binocular light microscope (Carl Zeiss, Stemi 305) and preserved in ethyl alcohol (70 per cent) at 4 °C in a refrigerator. Later these specimens were cleaned, spread on paper, dried and mounted on card-point tips.

The descriptions, measurements and imaging were carried out with a Leica M205A stereomicroscope, with 1 × objective and Leica DFC-500 digital camera with LED ring light illuminator. The images were stacked using Leica Application Suite (LAS) software. All measurements were taken as per Mikó et al. [28].

Variations in surface sculpture, relative proportions and sizes of body parts along with differences in colour, both within and between putative species, were assessed to distinguish between species and species groups (Table 1). Unique combinations of these characters were utilized to delimit species and species groups. Species were described by comparison with species descriptions in the literature and by examination of the types of Oriental species from various depositories worldwide following conventional terminology used in current literature for Platygastroidea. Diagnostic characters are specified for each species enabling their differentiation from all other species. Finally, keys were formulated to enable their identification.

**Table 1:** Character matrix distinguishing between *bilahari* species group and *manavati* species group of *Sparasion*

**Serial Number**	**Species**	**Sex**	**Genal carina [present (-) / absent(+)]**	**A1**	**A3/A2**	**No. of Transverse ledge/s on frons**	**Radialis [upcurved / straight]**	**Body colour**	**Leg colour**
***Sparasion bilahari* species group**									
*1.*	*S. albopilosellus* (Figs. 16 and 18A)	M	-	smooth	--	3	upcurved	black	orange (except mid and hind coxae)
*2.*	*S. bhairavi* (Figs. 1 and 18B)	F	+	smooth	1	3	upcurved	brown	xanthic
*3.*	*S. bhupali* (Figs. 2 and 18C)	F	+	smooth	0.9	2	upcurved	yellow	xanthic
*4.*	*S. bihagi* (Figs. 3 and 18D)	F	+	smooth	1	3	upcurved	yellow brown	xanthic
*5.*	*S. bilahari* (Figs. 4 and 18E)	F	+	smooth	0.7	3	upcurved	brown and yellow	xanthic
*6.*	*S. coconcu*s (Figs. 18G and 24)	F	+	smooth	1.1 based on original paper	3	upcurved	brown	xanthic
*7.*	*S. darbari* (Figs. 18J and 28)	F	+	smooth	1	3	upcurved	brown	xanthic
*8.*	*S. deepaki* (Figs. 18K and 29)	F	+	smooth	1.4	3	upcurved	black	xanthic
*9.*	*S. domes* (Fig. 30)	M	+	smooth		2	not observable^b^	black	xanthic
*10.*	*S. elbakyanae* (Figs. 31 and 18L)	F	+	smooth	1	3	upcurved	orange brown	xanthic
*11.*	*S. hindoli* (Figs. 34 and 33B)	F	+	smooth	1.2	3	upcurved	black and brown	xanthic
*12.*	*S. kalyani* (Figs. 33C and 35)	F	+	smooth	1.1	3	upcurved	black and brown	xanthic
*13.*	*S. kanakangi* (Figs. 33D and 37)	F	+	smooth	1.4	3	upcurved	black and brown	xanthic
*14.*	*S. micromerus* (Fig. 45)	M	+	smooth	NA^c^	3	not observable^b^	brown	xanthic
*15.*	*S. vanaspati* (Figs. 46G and 48)	M	+	smooth	NA^c^	3	upcurved	brown-black	xanthic
*16.*	*S. visvambari* (Figs. 46H and 49)	M	+	smooth	NA^c^	3	upcurved	brown-black	xanthic
*17.*	*S. zeelafi* (Figs. 46I and 50)	M	+	smooth	NA^c^	3	upcurved	brown-black	xanthic
**Sparasion manavati species group**									
*18.*	*S. cellularis* (Figs. 18F and 22)	F	-	dsp^e^	2.4	1	straight	steel blue	brown-black
*19.*	*S. coeruleus* (Figs. 18H, 25 and 26)	M, F	-	dsp^e^	4.5	1	straight	steel blue	brown-black
*20.*	*S. cullaris* (Figs. 18I and 27)	F	Not visible in image^d^	dsp^e^	2.5 text	1	straight	steel blue	brown-black
*21.*	*S. formosus* (Figs. 32 and 33A)	M	-	dsp^e^	NA^c^	1	straight	steel blue	brown-black
*22.*	*S. karivadana* (Figs. 33E and 38)	F	-	ssp^f^	1.2	1	upcurved	steel green blue	brown-black
*23.*	*S. manavati* (Figs. 33G and 41)	F	-	dsp^e^	3.7	1	straight	steel green blue	brown-black
*24.*	*S. meghmalhari* (Figs. 33H and 43)	F	-	dsp^e^	2.9	1	straight	steel blue	brown-black
*25.*	*S. pahadi* (Figs. 5 and 33I)	F	-	dsp^e^	2.2	1	straight	steel blue	brown-black
*26.*	*S. philippinensis* (Figs. 6 and 33J)	F	-	dsp^e^	3.2	1	straight	steel blue	brown-black
*27.*	*S. ratnangi* (Figs. 7 and 33L)	F	-	dsp^e^	3.3	1	straight	steel blue	brown-black
*28.*	*S. rupavati* (Figs. 8 and 33K)	F	-	dsp^e^	2.6	1	straight	steel blue	brown-black
*29.*	*S. salagami* (Figs. 9 and 33M)	F	-	dsp^e^	3.4	1	straight	steel blue	brown-black
*30.*	*S. shulini* (Figs. 10 and 46A)	F	-	dsp^e^	2.2	1	straight	steel blue	brown-black
*31.*	*S. sinensis* (Figs. 11 and 46B)	F	-	dsp^e^	2.8	1	straight	steel blue	brown-black
*32.*	*S. sivaranjini* (Figs. 12 and 46C)	F	-	dsp^e^	2.8	1	straight	steel blue-green	brown-black
*33.*	*S. syamalangi* (Figs. 13 and 46D)	F	-	dsp^e^	2.5	1	straight	steel green-blue	brown-black
*34.*	*S. todi* (Figs. 14 and 46E)	F	-	dsp^e^	2.2	1	straight	steel blue	brown-black
*35.*	*S. travancoricus* (Figs. 15 and 46F)	F	-	dsp^e^		1	straight	steel green-blue	brown-black
**Other**									
*36.*	*S. lividus* (Figs. 33F and 40)	M	-	dsp^e^	NA	0	straight	steel green-blue	Orange

*M* Male, *F* Female

^a^As given in the original description

^b^Species represented by type specimen only in which the wings are damaged

^c^Not applicable for males

^d^Species represented by type specimen only, the wing could not be moved to study veins as specimen brittle and the veins are not clear in the image

^e^Densely setigerous punctate

^f^Sparsely setigerous punctate

Morphological terms follow Masner [29], Mikó et al. [28, 30], Johnson et al. [4] and Yoder et al. [31], and for macro- and microsculpture Harris [32].

Images of the primary types of species of *Sparasion* were accessed at the Ohio State University’s Museum of Biological Diversity database [10], as well as from the museums where the types are deposited. When it was not possible to discern the character states from the images of previously described species, the original descriptions of the respective species were relied upon for the redescriptions as well as for the formulation of the keys.

The holotypes and paratypes of all the new species described are deposited in the National Insect Museum (NIM), ICAR-National Bureau of Agricultural Insect Resources (ICAR-NBAIR), Bengaluru, India.

### Distribution mapping

The distribution maps of Oriental and Indian species were made using open source software DIVA-GIS based on topographic grids retrieved from the DIVA-GIS database (http://www.diva-gis.org/). The collection localities of all specimens used in this study are from literature and from collections made by the authors [KV, PM, KS, FRK].

### Nomenclatural acts

This electronic version of this research paper is in conformity with the requirements of the International Code of Zoological Nomenclature (ICZN). The new species described in this paper have been assigned Zoobank registration numbers and mentioned under the respective species.

## Data Availability

All data available in the main script. Type material at the National Insect Museum, ICAR – NBAIR, Bengaluru, India.

## Abbreviations

A1–A12: Antennomeres 1–12 (A1 = Scape, A2 = Pedicel)
L: Length
W: Width
H: Height
OD: Ocellar diameter
OOL: Ocular ocellar line
POL: Posterior ocellar line
LOL: Lateral ocellar line
IOS: Interorbital space
R: Radius (submarginalis)
R1: First abscissa of radius (postmarginalis)
r-rs: Radial-radial sector cross vein (stigmalis)
T1–T6: Metasomal tergites 1–6
IEBR: Institute of Ecology and Biological Resources, Vietnam
MCSN: Museo Civico di Storia Naturale “Giacomo Doria”, Genova
NHMUK: Natural History Museum, London, UK (formerly BMNH)
MNHN: Muséum National d’Histoire Naturelle, Paris, France
NBAIR: National Bureau of Agricultural Insect Resources, Bengaluru, India
SDEI: Senckenberg Deutsches Entomologisches Institut, Müncheberg, Germany
USNM: United States National Museum of Natural History, Washington DC, USA
